# The germline mutational process in rhesus macaque and its implications for phylogenetic dating

**DOI:** 10.1093/gigascience/giab029

**Published:** 2021-05-05

**Authors:** Lucie A Bergeron, Søren Besenbacher, Jaco Bakker, Jiao Zheng, Panyi Li, George Pacheco, Mikkel-Holger S Sinding, Maria Kamilari, M Thomas P Gilbert, Mikkel H Schierup, Guojie Zhang

**Affiliations:** Section for Ecology and Evolution, Department of Biology, University of Copenhagen, Universitetsparken 15, 2100 Copenhagen Ø, Denmark; Department of Molecular Medicine, Aarhus University, Brendstrupgårdsvej 21A, 8200 Aarhus N, Denmark; Animal Science Department, Biomedical Primate Research Centre, Lange Kleiweg 161, 2288 GJ Rijswijk, Netherlands; BGI-Shenzhen, Shenzhen 518083, Guangdong, China; BGI Education Center, University of Chinese Academy of Sciences, Shenzhen 518083, Guangdong, China; BGI-Shenzhen, Shenzhen 518083, Guangdong, China; Section for Evolutionary Genomics, The GLOBE Institute, University of Copenhagen, Oester Voldgade 5-7, 1350 Copenhagen K, Denmark; Department of genetics, Trinity College Dublin, 2 college green, D02 VF25, Dublin, Ireland; Greenland Institute of Natural Resources, Kivioq 2, 3900 Nuuk, Greenland; Section for Ecology and Evolution, Department of Biology, University of Copenhagen, Universitetsparken 15, 2100 Copenhagen Ø, Denmark; Section for Evolutionary Genomics, The GLOBE Institute, University of Copenhagen, Oester Voldgade 5-7, 1350 Copenhagen K, Denmark; Department of Natural History, NTNU University Museum, Norwegian University of Science and Technology (NTNU), NO-7491 Trondheim, Norway; Bioinformatics Research Centre, Aarhus University, C.F.Møllers Allé 8, 8000, Aarhus C, Denmark; Section for Ecology and Evolution, Department of Biology, University of Copenhagen, Universitetsparken 15, 2100 Copenhagen Ø, Denmark; BGI-Shenzhen, Shenzhen 518083, Guangdong, China; State Key Laboratory of Genetic Resources and Evolution, Kunming Institute of Zoology, Chinese Academy of Sciences, Kunming 650223, China; Center for Excellence in Animal Evolution and Genetics, Chinese Academy of Sciences, Kunming 650223, China

**Keywords:** Evolution, mutation rate, primates, phylogeny

## Abstract

**Background:**

Understanding the rate and pattern of germline mutations is of fundamental importance for understanding evolutionary processes.

**Results:**

Here we analyzed 19 parent-offspring trios of rhesus macaques (*Macaca mulatta*) at high sequencing coverage of ∼76× per individual and estimated a mean rate of 0.77 × 10^−8^  *de novo* mutations per site per generation (95% CI: 0.69 × 10^−8^ to 0.85 × 10^−8^). By phasing 50% of the mutations to parental origins, we found that the mutation rate is positively correlated with the paternal age. The paternal lineage contributed a mean of 81% of the *de novo* mutations, with a trend of an increasing male contribution for older fathers. Approximately 3.5% of *de novo* mutations were shared between siblings, with no parental bias, suggesting that they arose from early development (postzygotic) stages. Finally, the divergence times between closely related primates calculated on the basis of the yearly mutation rate of rhesus macaque generally reconcile with divergence estimated with molecular clock methods, except for the Cercopithecoidea/Hominoidea molecular divergence dated at 58 Mya using our new estimate of the yearly mutation rate.

**Conclusions:**

When compared to the traditional molecular clock methods, new estimated rates from pedigree samples can provide insights into the evolution of well-studied groups such as primates.

## Background

Germline mutations are the source of heritable disease and evolutionary adaptation. Thus, having precise estimates of germline mutation rates is of fundamental importance for many fields in biology, including searching for *de novo* disease mutations [1,2], inferring demographic events [3,4], and accurate dating of species divergence times [5–7]. Over the past 10 years, new sequencing techniques have allowed deep sequencing of individuals from the same pedigree, enabling direct estimation of the *de novo* mutation rate for each generation, and precise estimation of the individual parental contributions to germline mutations across the whole genome. Most such studies have been conducted on humans, using large pedigrees with up to 3,000 trios [8,9], leading to a consensus estimate of ∼1.25 × 10^−8^  *de novo* mutations per site per generation, with a mean parental age of ∼29 years, leading to a yearly rate of 0.43 × 10^−9^  *de novo* mutations per site per year and most variation between trios explained by the age of the parents [8,10–17].

The observed increases in the mutation rate with paternal age in humans and other primates [8,18,19] have generally been attributed to errors during replication [20,21]. In mammalian spermatogenesis, primordial germ cells go through meiotic divisions, to produce stem cells by the time of puberty. After this time, stem cell divisions occur continuously throughout the male lifetime. Thus, human spermatogonial stem cells have undergone 100–150 mitoses in a 20-year-old male and ∼610 mitoses in a 40-year-old male [1], leading to an additional 1.51 *de novo* mutations per year increase in the father's age [8]. Female age also seems to affect the mutation rate in humans, with 0.37 mutations added per year [8]. This maternal effect cannot be attributed to replication errors because, different from spermatogenesis, female oocytogenesis occurs during the embryogenesis process and is already finished before birth [22]. Moreover, there seems to be a bias towards males in contribution to *de novo* mutations, as the paternal to maternal contribution is 4:1 in humans and chimpanzees [8,18]. One recent study proposed that damage-induced mutations might be a potential explanation for the observation of both the maternal age effect and the male-bias also present in parents reproducing right after puberty when replication mutations should not have accumulated yet in the male germline [23]. Parent-offspring analyses can also be used to distinguish mutations that are caused by gametogenesis from mutations that emerge in postzygotic stages [24,25]. While germline mutations in humans are relatively well studied, it remains unknown how much variability exists among primates in the contribution of replication errors to *de novo* mutations, the parental effects, and the developmental stages at which these mutations are established (postzygotic or gametogenesis).

Up until now, the germline mutation rate has only been estimated using pedigrees in a few non-human primate species, including chimpanzee (*Pan troglodytes*) [18,26,27], gorilla (*Gorilla gorilla*) [27], orangutan (*Pongo abelii*) [27], African green monkey (*Chlorocebus sabaeus*) [28], owl monkey (*Aotus nancymaae*) [19], the baboon (*Papio anubis*) [29], and recently rhesus macaque (*Macaca mulatta*) [30]. The mutation rate of grey mouse lemur (*Microcebus murinus*) [31] has also been estimated in preprinted studies. To precisely call *de novo* mutations in the offspring, collecting and comparing the genomic information of the pedigrees is the first essential step for detecting mutations only present in offspring but not in either parent. Next, the *de novo* mutations need to be separated from sequencing errors or somatic mutations, which cause false-positive calls. Because mutations are rare events, detecting *de novo* mutations that occur within a single generation requires high sequencing coverage to cover a majority of genomic regions and identify the false-positive calls. Furthermore, the algorithms used to estimate the mutation rate should take false-negative calls into account. However, a considerable range of sequencing depth (ranging from 18× [28] to 120× [26]) has been applied in many studies for estimation of mutation rate. Different filtering methods have been introduced to reduce false-positive and false-negative calls, but the lack of standardized methodology makes it difficult to assess whether differences in mutation rate estimates are caused by technical or biological variability. In addition, most studies on non-human primates used small pedigrees with <10 trios, which made it difficult to detect any statistically significant patterns over *de novo* mutation spectra.

Studying non-human primates could help us understand whether the mutation rate is affected by life history traits such as mating strategies or the age of reproduction. The variation in mutation rate among primates will also be useful for re-calibrating the speciation times across lineages. The sister group of Hominoidea is Cercopithecoidea, including the important biomedical model species, rhesus macaque (*M. mulatta*), which is 93% identical to the genome of humans [32]. This species has a generation time estimate of ∼11 years [33], and their sexual maturity is much earlier than in humans, with females reaching maturity at ∼3 years old, while males mature at ∼4 years [34]. While female macaques generally start reproducing right after maturation, males rarely reproduce in the wild until they reach their adult body size, at ∼8 years old [35]. They are also a promiscuous species and do not form pair bonds but reproduce with multiple individuals. These life history traits, as a member of the closest related outgroup species of the hominoid group, make the rhesus macaque an interesting species for investigating the differences and common features in mutation rate processes across primates.

In this study, we produced high-depth sequencing data for 33 rhesus macaque individuals (76× per individual) representing 19 trios. This particular dataset consists of a large number of trios, each with high coverage sequencing, and allowed us to test different filter criteria and choose the most appropriate ones to estimate the species mutation rate with high confidence. With a large number of *de novo* mutations phased to their parents of origin, we can statistically assess the parental contribution and the effect of the parental age. We characterize the type of mutations and their location on the genome to detect clusters and shared mutations between siblings. Finally, we use our new estimate to infer the effective population size and date their divergence time from closely related primate species.

## Results

### Estimation of mutation rate for 19 trios of rhesus macaques

**Figure 1:**
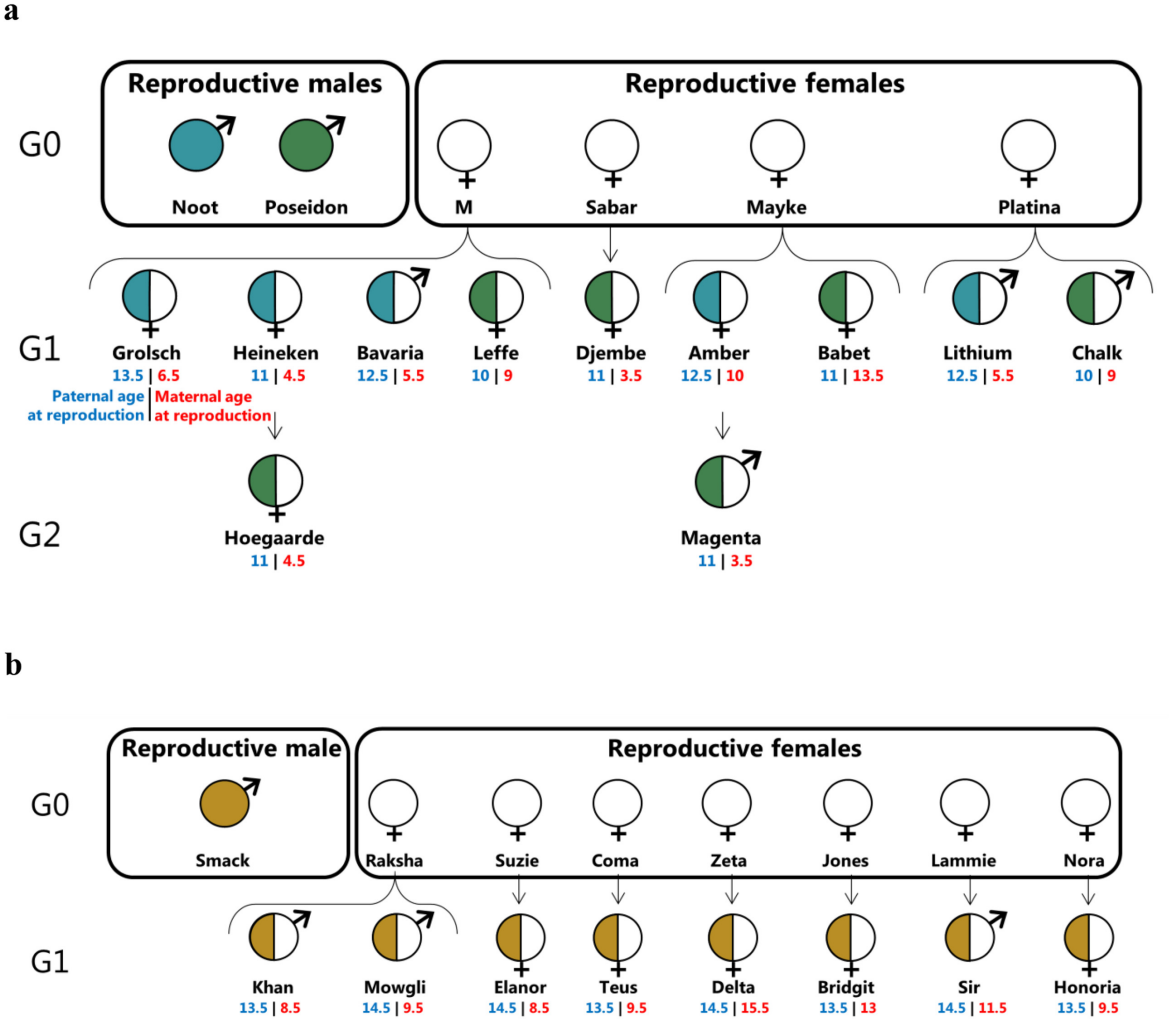
Pedigree of the 19 trios used for the direct estimation of mutation rate. (a) The first group is composed of 2 reproductive males and 4 reproductive females. (b) The second group contained 1 reproductive male and 7 reproductive females. In each offspring, the color on the left corresponds to the paternal lineage and under the name are the age of the father (in blue) and mother (in red) at the time of reproduction. The reproductive ranges are 4.5 years for males and 12.2 years for females.

To produce an estimate for the germline mutation rate of rhesus macaques, we generated high-coverage (76× per individual after mapping, minimum 64×, maximum 86×) genome sequencing data for 19 trios of 2 unrelated families (Fig. 1). The first family consisted of 2 reproductive males and 4 reproductive females, and the second family had 1 reproductive male and 7 reproductive females. In the first family, the pedigree extended over a third generation in 2 cases. The promiscuous mating habits of rhesus macaques allowed us to follow the mutation rates in various ages of reproduction and compare numerous full siblings and half-siblings.

We developed a pipeline for single-nucleotide polymorphism (SNP) calling with multiple quality control steps involving the filtering of reads and sites (see Methods). For each trio, we considered candidate sites as *de novo* mutations when (i) both parents were homozygotes for the reference allele, while the offspring was heterozygous with 30–70% of its reads supporting the alternative allele; and (ii) the 3 individuals passed the depth and genotype quality filters (see Methods). These filters were calibrated to ensure a low rate of false-positive results among the candidate *de novo* mutations. To validate our method, we applied our pipeline to a published trio of chimpanzee [27], for which the prior published mutation rate was estimated at 1.27 × 10^−8^ mutations per site per generation (95% CI: 0.95–1.7 × 10^−8^) and obtained a very similar rate of 1.25 × 10^−8^ de novo mutations per site per generation.

We obtained an unfiltered set of 12,785,386 mean candidate autosomal SNPs per trio (se = 26,196), of which a total of 177,227 were potential Mendelian violations (mean of 9,328 per trio; se = 106). Of these, 744 SNPs passed the filters as *de novo* mutations, ranging from 25 to 59 for each trio and a mean of 39 *de novo* mutations per trio (se = 2) (see Supplementary Table S1). We manually curated all mutations using IGV on bam files and found that 663 mutations convincingly displayed as true-positive calls. This leaves a maximum of 10.9% (81 sites) that could be false-positive results due to the incorrect absence of a call of the variant in the parents or the incorrect presence of a called variant in the offspring (see Supplementary Fig. S1 and the 81 curated mutations in Supplementary Appendix 2). Most of those sites were in dinucleotide repeat regions or short tandem repeats (56 sites), while others were in non-repetitive regions of the genome (25 sites).

To confirm the authenticity of the *de novo* mutations, we performed PCR experiments for all candidate *de novo* mutations from 1 trio before manual correction. We designed primers to a set of 39 *de novo* candidates among which 3 *de novo* mutations were assigned as spurious from the manual inspection. Of these, 24 sites were successfully amplified and sequenced for all 3 individuals, i.e., mother, father, and offspring, including 1 of the spurious sites. Among those sequenced sites, 23 were correct and only 1 was wrong (Supplementary Fig. S2). This invalidated candidate was the spurious candidate removed by manual curation, therefore supporting our manual curation method. The PCR validation results suggested a lower false-positive rate of 4.2% before manual curation. Becaus the PCR validation was done only on 24 candidates we decided to keep the strict false-positive rate of 10.9% found by manual curation.

We then estimated the mutation rate, per site per generation, as the number of mutations observed, and corrected for false-positive calls, divided by the number of callable sites. The number of callable sites for each trio ranged from 2,334,764,487 to 2,359,040,186, covering on average 88% of the autosomal sites of the rhesus macaque genome. A site was defined as callable when both parents were homozygotes for the reference allele and all individuals passed the depth and genotype quality filters at that site. Because callability is determined using the base-pair resolution vcf file, containing every single site of the genome, all filters used during calling were taken into account during the estimation of callability, except for the site filters and the allelic balance filter, only applicable to variant sites. We then corrected for false-negative rates, calculated as the number of “good” sites that could be filtered away by both the site filters and allelic balance filters—estimated at 4.02% (see equation 1 in Methods section). Thus, the final estimated mean mutation rate of the rhesus macaques was 0.77 × 10^−8^  *de novo* mutations per site per generation (95% CI: 0.69 × 10^−8^–0.85 × 10^−8^). This rate is higher than the 0.58 × 10^−8^  *de novo* mutations per site per generation found by Wang et al. [30], yet, this difference can be explained by the older age of the parents at the time of reproduction in our study (mean 10.4 years) than in Wang et al. [30] (mean parental age of 7.5 years). After normalization with the parental age, the estimated yearly rates in these 2 studies are very close, with our study only 5% lower. We removed the 81 sites that, based on manual curation, could represent false-positive calls from the following analyses (see the 663 *de novo* mutations in Supplementary Table S2).

### Parental contribution and age effect on the *de novo* mutation rate

**Figure 2:**
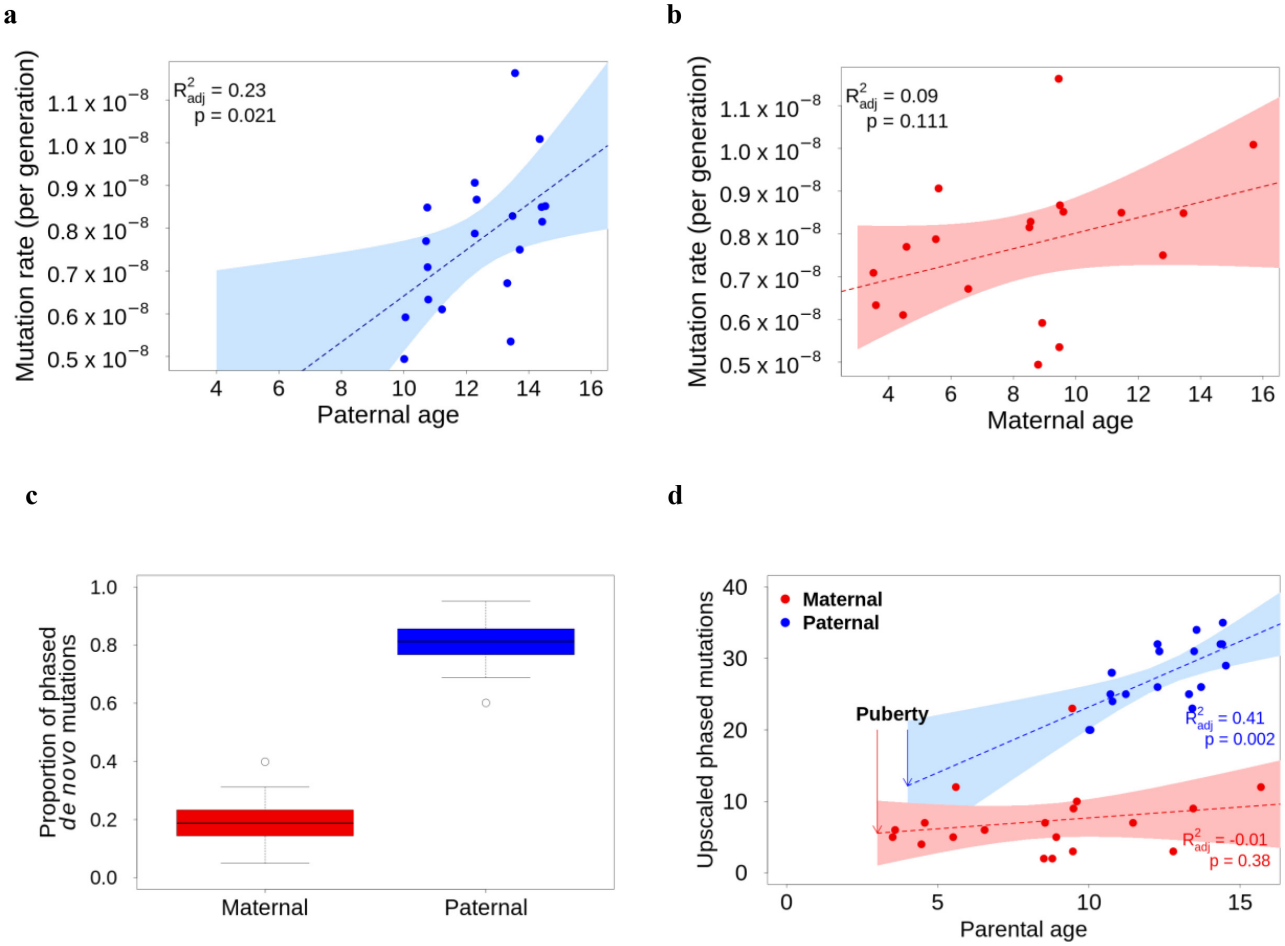
Parental contribution and age effect on the *de novo* mutation rate. (a) There is a positive correlation between the mutation rate and the paternal age shown by the linear regression (dotted lines) and 95% CI (shading). (b) The correlation between maternal age and mutation rate is not significant. (c) Boxplot of the maternal and paternal contribution in *de novo* mutations, with minimum, maximum and outlier values (error bars and dots), values within the first and third quartiles (colored block) and median (horizontal lines). (d) Upscaled number of *de novo* mutations given by each parent shows a similar contribution at the age of sexual maturation and a substantial increase with male age.

We observed a positive correlation between the paternal age and the mutation rate in the offspring (adjusted *R*^2^ = 0.23; *P* = 0.021; regression: µ = 1.022 × 10^−9^ + 5.393 × 10^−10^ × age_paternal_; *P* = 0.021; Fig. 2a). We also detected a slight positive correlation with the maternal age, though not significant (adjusted *R*^2^ = 0.09; *P* = 0.111; regression: µ = 6.200 × 10^−9^ + 1.818 × 10^−10^ × age_maternal_; *P* = 0.111; Fig. 2b). A multiple regression of the mutation rate on paternal and maternal age resulted in this formula: µ_Rhesus_ = 1.355 × 10^−9^ + 7.936 × 10^−11^ × age_maternal_ + 4.588 × 10^−10^ × age_paternal_ (*P* = 0.06), where µ_Rhesus_ is the mutation rate for the species.

We were able to phase 337 mutations to their parent of origin, which accounted for more than half of the total number of *de novo* mutations (663). There is a significant male bias in the contribution of *de novo* mutations, with a mean of 80.6% paternal *de novo* mutations (95% CI: 76.6%–84.6%; *T* = 22.62, df = 36, *P* < 2.2 × 10^−16^; Fig. 2c). Moreover, with more than half of the *de novo* mutations phased to their parent of origin, we were able to disentangle the effect of the age of each parent on mutation rate independently (Fig. 2d). By assuming that the ratio of mutations phased to a particular parent was the same in the phased mutations as in the unphased ones, we could predict the total number of mutations given by each parent. For instance, if an offspring had 40 *de novo* mutations and only half were phased, with 80% given from its father, we would apply this ratio to the total number of mutations in this offspring, ending up with 32 *de novo* mutations from its father and 8 from its mother. This analysis suggested a stronger male age effect on the number of mutations (adjusted *R*^2^ = 0.41, *P* = 0.002), and a similar, non-significant maternal age effect (adjusted *R*^2^ = −0.01, *P* = 0.38). The 2 regression lines meet around the age of sexual maturity (3 years for females and 4 years for males), which is consistent with a similar accumulation of *de novo* mutations during the developmental process from birth to sexual maturity in both sexes, but the variances on the regression line slopes are large (see Fig. 2c and Supplementary Fig. S3 for the same analysis with a Poisson regression). Using these 2 linear regressions, we can predict the number of *de novo* mutations in the offspring based on the age of each parent at the time of reproduction: No. of mutations_Rhesus_ = 4.6497+ 0.3042 × age_maternal_ + 4.8399 + 1.8364 × age_paternal_, where No. of mutations_Rhesus_ is the number of *de novo* mutations for the given trio. The expected mutation rates calculated using the 2 different regression models show similar correlations with the observed mutation rate (*R*^2^ = 0.54, *P* = 0.016 for the first regression and *R*^2^ = 0.54, *P* = 0.016 for the upscaled one, see Supplementary Fig. S4). However, on the first regression on the mutation rate, the maternal age effect may be confounded by the paternal age, as maternal and paternal age are correlated in our dataset, yet, non-significantly (*R*^2^ = 0.38, *P* = 0.106; see Supplementary Fig. S5). The upscaled regression unravels the effect of the parental age independently from each other. This regression can also be used to infer the contribution of each parent at different reproductive ages. For instance, if both parents reproduce at 5 years old, based on the upscaled regression, the father is estimated to give ∼14 *de novo* mutations (95% CI: 6–22) and the mother ∼6 *de novo* mutations (95% CI: 3–10), corresponding to a contribution ratio from father to mother of 2.3:1 at 5 years old. If they reproduce at 15 years old, this ratio would be 3.6:1 with males giving ∼32 *de novo* mutations (95% CI: 29–36) and females ∼9 *de novo* mutations (95% CI: 4–14). It seems that the male bias increases with the parental age, yet, our model was based on too few data points in early male reproductive ages to reach a firm conclusion. For the 2 extended trios for which a second generation is available, we looked at the proportion of *de novo* mutations in the first offspring that were passed on to the third generation—the third generation inherited a heterozygote genotype with the alternative allele being the *de novo* mutation. In 1 case, 66% of the *de novo* mutations in the female (Heineken) were passed to her daughter (Hoegaarde), while in another case, 40% of the *de novo* mutations in the female (Amber) were passed to her son (Magenta). These deviations from the expected 50% inheritance rate are not statistically significant (binomial test; *P*_Hoegaarde_ = 0.14 and *P*_Magenta_ = 0.27).

### Characterizations of *de novo* mutations

**Figure 3:**
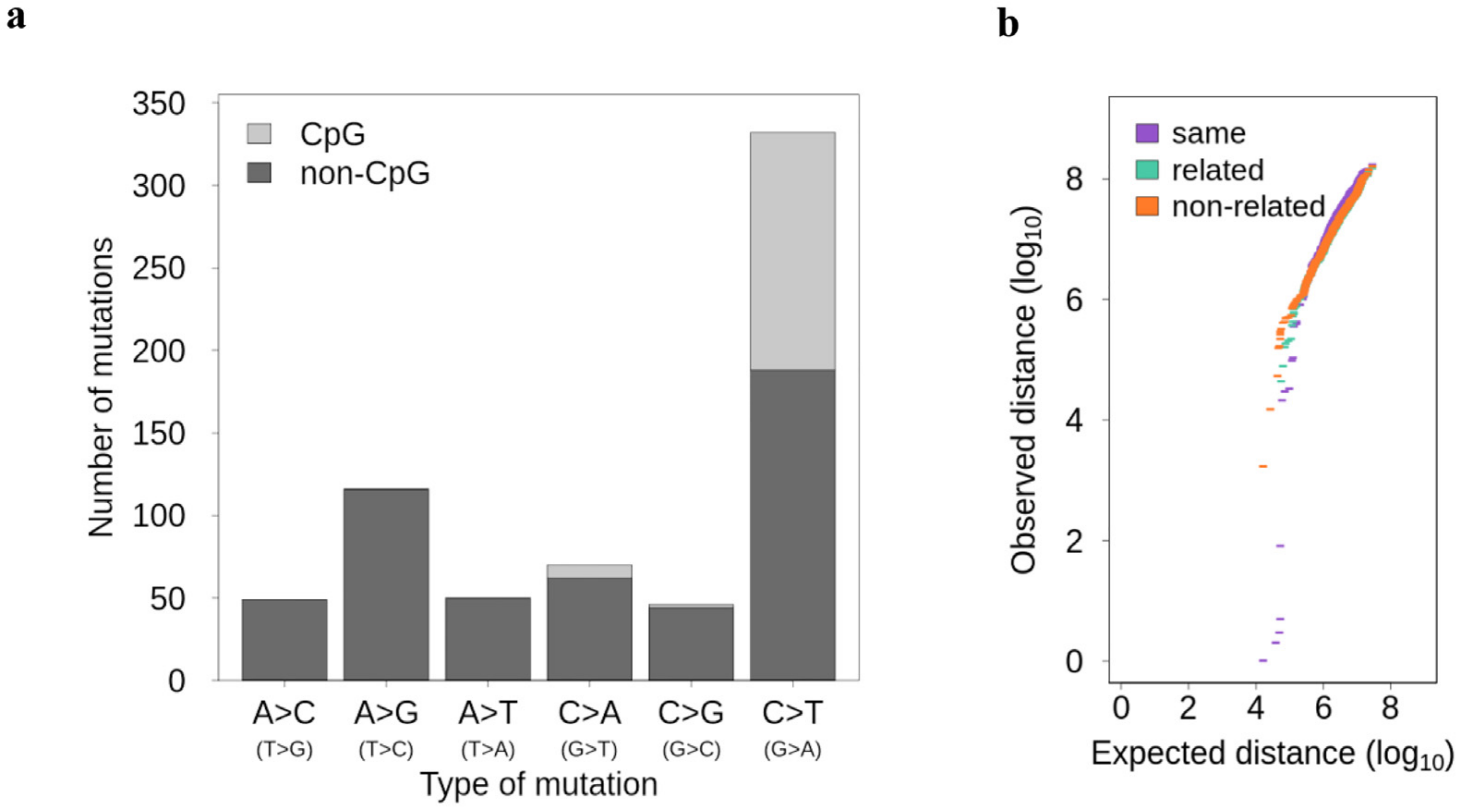
Characterizations of the *de novo* mutations. (a) The type of *de novo* mutations in CpG and non-CpG sites. (b) QQ-plot of the distance between *de novo* mutations compared to a uniform distribution within individuals (purple), between related individuals (green), and between non-related individuals (orange).

We characterized the type of *de novo* mutations and found that transitions from a strong base to weak base (G > A and C > T) were most common (332 of 663), and similar to what was already reported for rhesus macaque [30], we found 43% of those mutations located in CpG sites (Fig. 3a). In total, 23.2% (154 of 663) of the *de novo* mutations were located in CpG sites. This is slightly higher than what has been found in humans, for which 19% of the *de novo* mutations are in CpG sites [11], but not significantly (human: χ^2^ = 2.774, df = 1, *P* = 0.096) and similar to the 24% reported for rhesus macaque [30]. Moreover, 32.1% (144 of 448) of the transition mutations (A > G and C > T) were in CpG sites, higher than what has been found in chimpanzees, with 24% of the transition *de novo* mutations in CpG sites [18]. The transition to transversion ratio (ti/tv) was 2.08, which is similar to the ratio observed in other species (human: ti/tv ∼ 2.16 [36]; human ti/tv ∼ 2.2 [17]; chimpanzee: ti/tv ∼ 1.98 [26]. The 663 *de novo* mutations showed some clustering in the genome (Fig. 3b and Supplementary Fig. S6). Across all trios, we observed 11 clusters, defined as windows of 20,000 bp where >1 mutation occurred in any individual, involving 23 mutations. Four clusters were made of mutations from a single individual, accounting for 8 mutations (Fig. 3b). Overall, 3.47% of the *de novo* mutations were located in clusters, and 1.21% were mutations within the same individual located in a cluster, which is significantly lower than the 3.1% reported in humans [37] (χ^2^ = 7.35, df = 1, *P* = 0.007; Supplementary Fig. S7, Supplementary Table S3). We observed 23 mutations occurring recurrently in >1 related individual (Table 1), which accounted for 3.5% of the total number of *de novo* mutations (23 of 663) and 1.5% of sites (10 of 650 unique sites). Four *de novo* mutations (2 sites) were shared between half-siblings on the maternal side, and 19 (8 sites) were shared between half-siblings on the paternal side. However, there was no significant difference between the proportion of mutations shared between pairs of individuals related on the maternal side (9 pairs, 0.70% shared) and pairs related on their paternal side (53 pairs, 0.80% shared; Fisher exact test *P* = 1). In 6 sites, the phasing to the parent of origin confirmed that the mutation was coming from the common parent for ≥1 individual (Table 1). Moreover, the phasing was never inconsistent by attributing a shared *de novo* mutation to the other parent than the parent in common. However, 5 shared sites did appear as mosaic in the common parent, with a maximum of 5% of the reads of the father supporting the alternative allele (4 of 80 reads). Nine of the *de novo* mutations (1.4% of the total *de novo* mutations) were located in coding sequences (CDS regions), which is close to the overall proportion of coding sequences region (1.2%) in the whole macaque genome. Eight of those 9 mutations were non-synonymous.

**Table 1:** 6 mutations shared between related individuals

Chromosome	Position	Ref	Alt	Sibling 1		Sibling 2		Sibling 3		Sibling 4		Common parent	Name parent
				Name	Phasing ^a^	Name	Phasing^a^	Name	Phasing^a^	Name	Phasing^a^		
chr2	101,979,137	G	A	Khan	P	Delta	U					Father	Smack
chr6	132,663,101	A	T	Amber	U	Babet	M					Mother	Mayke
chr7	60,635,102	G	T	Sir	U	Honoria	U					Father	Smack
chr7	116,648,579	G	A	Amber	M	Babet	U					Mother	Mayke
chr9	32,544,257	C	T	Hoegaarde	U	Djembe	U	Babet	U	Magenta	U	Father	Poseidon
chr10	65,163,492	G	A	Khan	P	Delta	P					Father	Smack
chr15	35,463,257	C	T	Leffe	U	Babet	U	Magenta	U			Father	Poseidon
chr17	88,174,686	C	A	Hoegaarde	P	Babet	P					Father	Poseidon
chr19	7,047,030	C	T	Leffe	U	Djembe	U					Father	Poseidon
chr19	15,861,061	C	T	Bavaria	P	Lithium	U					Father	Noot

a M: maternal; P: paternal; U: unphased.

### Molecular dating with trio-based mutation rate

On the basis of our inferred mutation rate and the genetic diversity of Indian rhesus macaques (π = 0.00247) estimated using whole-genome sequencing data from >120 unrelated wild individuals [33], we calculated the effective population size (*N_e_*) of rhesus macaques to be 79,874. This is similar to the *N_e_* = 80,000 estimated previously using µ*=* 0.59 × 10^−8^ from hippocampal transcriptome and H3K4me3-marked DNA regions from 14 individuals [38], yet higher than *N_e_* = 61,800 estimated using µ*=* 1 × 10^−8^ with 120 individuals' full-genome data [33]. Because captive animals usually reproduce later than in the wild, which could affect the mean mutation rate per generation, we used the regression instead of the mutation rate per generation to correct for this possible bias. Assuming a generation time of 11 years and a mean reproduction age of 10 years for females and 12 years for males, the yearly mutation rate of rhesus macaques was calculated on the basis of both regression models. Using the regression estimating the per generation rate given both parental ages, we estimated a yearly rate of 0.7 × 10^−9^ mutations per site per year. Yet, as both parental age effects may be confounded in this regression we choose to use the regression yearly rate of the number of mutations given by males and females independently, and the mean callability (see equation 2 in the Methods section). The yearly mutation rate of rhesus macaques with this calculation was 0.62 × 10^−9^ per site per year, almost 1.5 times that of humans [8].

**Figure 4:**
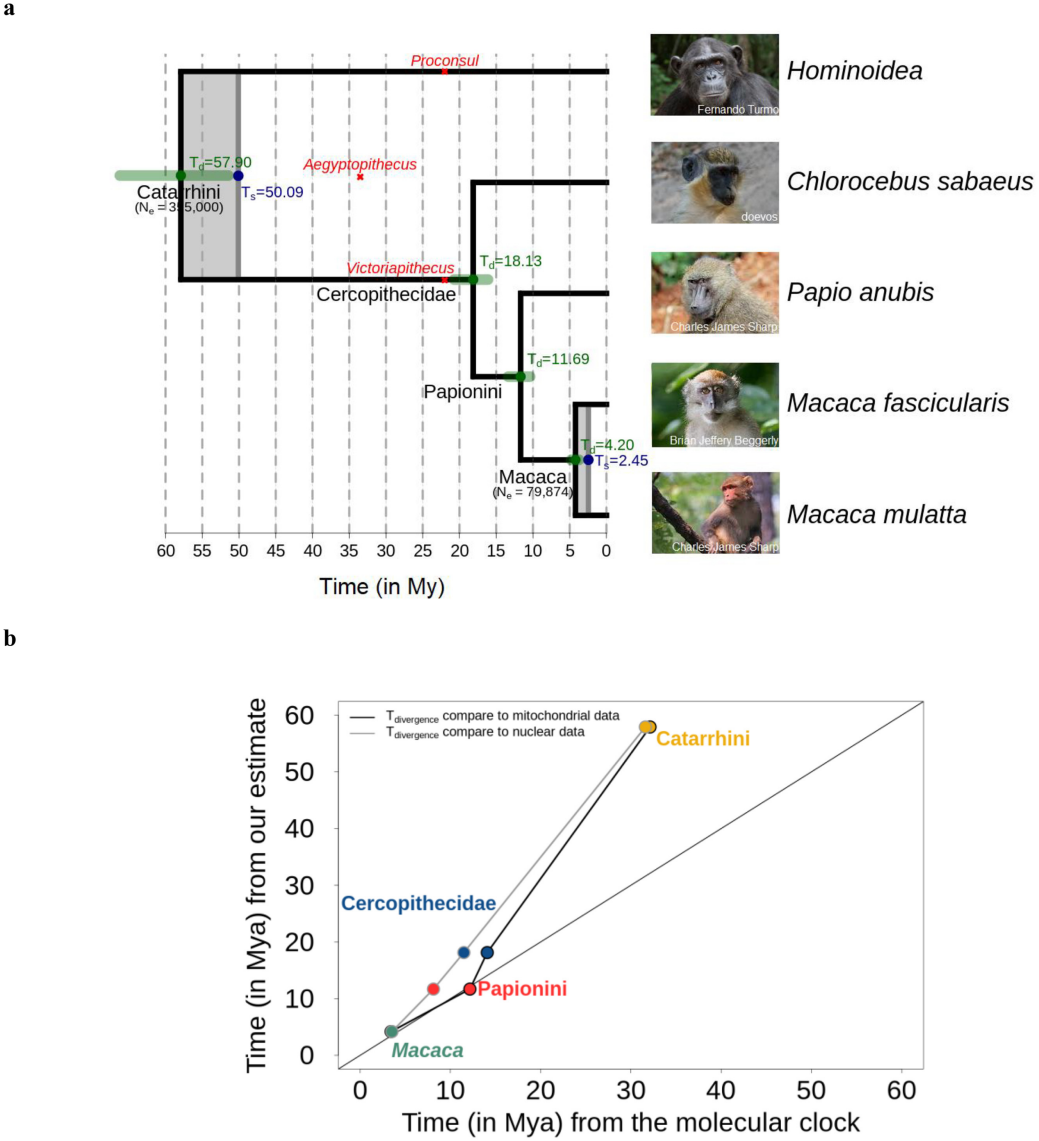
Molecular dating with pedigree-based mutation rate. (a) Primate phylogeny based on the yearly mutation rate (0.62 × 10*^−^*^9^ per site per year). In green are the confidence intervals of our divergence time estimates (*T*_d_), and grey shades represent the time of speciation (*T*_s_). The effective population sizes are indicated under the nodes (*N_e_* Macaca ancestor is our estimate of *N_e_*  *Macaca mulatta* and *N_e_* Catarrhini from the literature [48]). (b) Comparison of our divergence time and speciation time with the previous estimation using the molecular clock from mitochondrial [43] and nuclear data [44] calibrated with fossil records.

Given that a precise evolutionary mutation rate is essential for accurate calibration of molecular divergence events between species, we used the mutation rate we inferred for rhesus macaques to re-date the phylogeny of closely related primate species with full genome alignment available [39] (Fig. 4a). The molecular divergence time (*T*_d_) is the time since an ancestral lineage started to split into 2 descendant lineages and can be inferred from the genetic divergence between the 2 descendant lineages and the mutation rate. The speciation time (*T*_s_) is a younger event that implies no more gene flow between lineages [40]. In the backward direction, the alleles of 2 descendant lineages are randomly sampled from their parents until going back to the most recent common ancestor [41]. This stochastic event, known as the coalescent, depends on the population sizes, being slower in a large population [42]. Thus, from the divergence time, the speciation time can be inferred given the rate of coalescence (see equation 3 in the Methods section). We also compared our results to those of previous dating attempts based on molecular phylogenetic trees calibrated with fossil records (Fig. 4b). We found that the 2 methods concur for the most recent events. Specifically, we estimated that the *M. mulatta* and *Macaca fascicularis* genomes had already diverged ∼4.20 million years ago (Mya) (95% CI: 3.74–4.81), which is slightly older than previous estimates using the molecular clock calibrated with fossils, as the molecular divergence of the 2 species has been estimated at 3.44 Mya (95% CI: 2.75–4.21) with mitochondrial data [43] and 3.53 Mya from nuclear data [44]. We estimated a speciation event between the 2 species 2.45 Mya after the coalescent time, also consistent with previous findings of a most common recent ancestor to the 2 populations of the rhesus macaque, the Chinese and the Indian population, ∼1.94 Mya based on coalescent simulations [45]. For the next node, the molecular clock seems to differ between mitochondrial and nuclear data, as the divergence time for the Papionini group into the *Papio* and *Macaca* genera has been estimated to 8.13 Mya using nuclear data [44], and 12.17 Mya (95% CI: 10.51–13.64) with mitochondrial data [43]. We estimated a divergence time between these 2 genera of 11.69 Mya (95% CI: 10.39–13.37). The effective population size of this ancestral node is yet unknown, limiting the estimation of the speciation time. However, using the baboon yearly mutation rate of 0.55 × 10^−9^ per site per year [29] and the baboon branch, the divergence time of this node was also estimated at ∼12.5 Mya. For earlier divergence events, our estimated divergence times are more ancient than previous reports. For instance, we estimated that the Cercopithecini and Papionini diverged 18.13 Mya (95% CI: 16.11–20.74), while other studies had calculated 11.55 Mya using nuclear data [44] and 14.09 Mya (95% CI: 12.24–15.82) using mitochondrial data [43]. Moreover, using the green monkey rate  (1.1 × 10^−9^ per site per year [28]) and branch length led to a divergence time of this node 10.1 Mya. There is high uncertainty on this yearly rate as the age of the parents was unknown and the generation is used to calculate the yearly rate. Finally, the divergence between Cercopithecoidea and Hominoidea has been reported between 25 and 30 Mya [39,46], with an estimation of 31.6 Mya using the nuclear molecular clock [44] and 32.12 Mya  (95% CI: 29.44–33.82) using the mitochondrial one [43]. Our dating of the divergence time between the Cercopithecoidea and Hominoidea of 57.90 Mya (95% CI: 51.43–66.22) is substantially older than previous estimates. However, the estimated speciation time inferred on the basis of the ancestral population size suggested a speciation of the Catarrhini group into 2 lineages 50.09 Mya (Fig. 4b). Using the human rate (0.43 × 10^−9^ per site per year) to estimate this divergence time led to an even older divergence time ∼61 Mya. Yet, with the chimpanzee yearly rate (0.64 × 10^−9^ per site per year) and branch length, the Cercopithecoidea/Hominoidea divergence time would decrease to ∼41.6 Mya, stressing the bias that can be brought by using a single rate to date such an old speciation event. Instead, the mutation rate could have changed over time. As estimating the divergence time of the *Papio*/*Macaca* node from both the macaque and the baboon rates conciliate, we could infer that the rate only changed before this divergence event. Back then the mutation rate could have been higher, for instance, similar to the green monkey 1.1 × 10^−9^ per site per year [28], leading to a divergence of the Cercopithecoidea/Hominoidea ∼37.5 Mya and a speciation 29.7 Mya. The yearly mutation rate of the crown Catarrhini could even have been higher considering the rate estimated in New World monkeys that are smaller primates with shorter generation time (e.g., 2.7 × 10^−9^ per site per year in owl monkeys [19]). Another possible cause of this discrepancy between our estimation and the literature can be due to different genetic divergence between species than the one used in this study. However, by using another whole-genome alignment [47], we estimated similar divergence time with the *M. mulatta*/*M. fascicularis* ∼3.9 Mya, *Papio*/*Macaca* ∼12.2 Mya, Cercopithecini/Papionini ∼18.9 Mya, and Cercopithecoidea/Hominoidea ∼60.1 Mya.

## Discussion

Despite many efforts to accurately estimate direct *de novo* mutation rates, it is still a challenging task owing to the rare occurrence of *de novo* mutations and the small sample size that is often available. Sequencing coverage is known to be a significant factor in affecting false-positive and false-negative calls when detecting *de novo* mutations [1,26]. A minimal sequencing coverage at 15× was recommended for SNP calling [49]. However, such coverage cannot provide sufficient power to reduce false-positive calls because the lower depth threshold cannot preclude Mendelian violations due to sequencing errors. Moreover, a larger portion of the genome would be removed in the denominator at low depth in order to reduce the false-negative rate. While most studies on direct estimation of mutation rate use 35–40× coverage [8,19,27], their methods to reduce the false-positive and false-negative rate differ. Some studies use the deviation from 50% of the *de novo* mutation pass to the next generation to infer the false-positive rate [8,19]. Others use probabilistic methods to access the callability [27], or simulation of known mutation to control the pipeline quality [28]. Differences in methods likely affect the calculated rate. Here, we produced sequences at 76× coverage, which allows us to apply conservative filtering processes, while still obtaining high coverage (88%) of the autosomal genome region when inferring *de novo* mutations. To our knowledge, only 1 other study has used very high coverage (120× per individual), on a single trio of chimpanzees [26].

Our estimated rate is higher than the 0.58 × 10^−8^  *de novo* mutations per site per generation estimated in a recent study [30]. The difference should be mainly attributed to the fact that they sequenced the offspring of younger parents (mean parental age of 7.1 years for females and 7.8 years for males compared to 8.4 years for females and 12.4 years for males in the present study). Using our regression from the phased mutation, we estimated a mutation rate of 0.51 × 10^−8^ per site per generation, when males reproduce at 7.8 years and females reproduce at 7.1 years old. Moreover, using their regression based on the age of puberty and the increase of paternal mutation per year, Wang and collaborators estimated a per generation rate of 0.71 × 10^−8^ mutations when males reproduce at 11 years, and a yearly rate of 0.65 × 10^−9^ mutations per site per year, which is ∼5% higher than our estimate of 0.62 × 10^−9^ [30]. This difference may be due to any combination of stochasticity, differences in *de novo* mutation rate pipelines (callability estimate, false-negative rate, and false-positive rate estimate), and different models for converting pedigree estimates to yearly rates. Our combination of high-coverage data and a large number of trios allowed us to estimate the germline mutation rate of rhesus macaques at ∼0.77 × 10^−8^  *de novo* mutation per site per generation, ranging from 0.49 × 10^−8^ to 1.16 × 10^−8^. This is similar to the mutation rate estimated for other non-Hominidae primates—0.81 × 10^−8^ for the owl monkey (*A. nancymaae*) [19] and 0.94 × 10^−8^ for the African green monkey (*C. sabaeus*) [28]—while all Hominidae seem to have a mutation rate that is higher than 1 × 10^−8^  *de novo* mutations per site per generation [8,27]. However, if we calculate the *de novo* mutation rate per site per year, the rate of rhesus macaque (0.62 × 10^−9^) is almost 1.5-fold the human one of 0.43 × 10^−9^ mutations per site per year [8].

One of the main factors affecting the mutation rate within the species is the paternal age at the time of reproduction, which has been attributed to the accumulation of replication-driven mutations during spermatogenesis [20,21, 50] and has been observed in many other primates [8,13, 18, 19, 27]. In rhesus macaques, the rate at which germline mutation increases with paternal age seems faster than in humans; we inferred 1.84 mutations more per year for the rhesus macaque father (95% CI: 0.77–2.90 for a mean callable genome of 2.35 Mb), compared to 1.51 in humans (95% CI: 1.45–1.57 for a mean callable genome of 2.72 Mb) [8]. For females, there is less difference, with 0.30 more mutations per year for the mother in rhesus macaque (95% CI: −0.41 to 1.02), and 0.37 more per year in human mothers (95% CI: 0.32–0.43) [8]. In rhesus macaques, males produce a larger number of sperm cells per unit of time (23 × 10^6^ sperm cells per gram of testis per day [51]) than humans (4.4 × 10^6^ sperm cells per gram of testis per day [52]). This could imply a higher number of cell division per unit of time in rhesus macaques and thus more replication error during spermatogenesis. This is also consistent with the generation time effect, which stipulates that an increase in generation time would decrease the number of cell divisions per unit of time, as well as the yearly mutation rate assuming that most mutations arise from replication errors [21,24, 53–56]. Indeed, humans have a generation time of 29 years, while it is 11 years for rhesus macaques. Another explanation for a higher increase of mutation rate with paternal age could be differences in the replication machinery itself. Due to higher sperm competition in rhesus macaque, the replication might be under selective pressure for fast production at the expense of replication fidelity, leading to fewer DNA repair mechanisms. As in other primates, we found a male bias in the contribution of *de novo* mutations, as the paternal to maternal ratio is 4.2:1. This ratio is higher than the 2.7:1 ratio observed in mice [57] and slightly higher than the 4:1 ratio observed in humans [57–59]. Similarly to the wild, the males of our dataset reproduced from 10 years old, which did not allow us to examine whether the contribution bias was also present just after maturation. Moreover, the promiscuous behavior of the rhesus macaque leads to fathers reproducing with younger females. Using our model to compare the contribution of each parent reproducing at similar ages, it seems that the male bias increases with the parental age, with a lower difference in contribution at the time of sexual maturation (2.3:1 for parents aged 5 years) and an increase in male to female contribution with older parents (3.6:1 for parents aged 15 years). This result differs from humans, where the male bias seems constant over time [23], but more time points in macaque would be needed to interpret the contribution over time. In rhesus macaques, the ratio of paternal to maternal contribution to the shared mutations between related individuals is 1:1, similarly to what has been shown in mice [57], highlighting that those mutations probably occur during primordial germ cell divisions in postzygotic stages. Our study shows many shared patterns in the *de novo* mutations among non-hominid primates. More estimation of mammals could help elucidate whether these features are conserved across a broad phylogenetic scale. Moreover, further work would be needed to understand whether some gamete production stages are more mutagenic in some species than others.

An accurate estimation of the mutation rate is essential for the precise dating of species divergence events. We used the rhesus macaque mutation rate to estimate its divergence time with related species for which whole-genome alignments are already available and their molecular divergence times have been investigated before with other methods [39]. The results of our direct dating method, based on molecular distances between species and *de novo* mutation rate, matched those of traditional molecular clock approaches for speciation events within 10–15 million years. However, it often produced earlier divergence times for more ancient nodes than the molecular clock method. This incongruence might be attributed to the fossils that were used for calibration with the clock method, which has many limitations [7,40, 60]. A fossil used for calibrating a node is usually selected to represent the oldest known specimen of a lineage. Still, it cannot be known whether even older specimens existed [60]. Thus, a fossil is usually assumed to be younger than the real divergence time of the species [61]. Moreover, despite the error associated with the dating of a fossil itself, determining its position on a tree can be challenging and have effects on the inferred ages across the whole tree [7,40]. For instance, the Catarrhini node, marking the divergence between the Cercopithecoidea and the Hominoidea, is often calibrated in primate phylogenies [60]. This node has been calibrated to ∼25 Mya using the oldest known Cercopithecoidea fossil (*Victoriapithecus*) and the oldest known Hominoidea fossil (*Proconsul*), both ∼22 My old [62]. However, if the oldest Catarrhini fossil (*Aegyptopithecus*) of 33–34 My age is used, this node could also be calibrated to 35 Mya [46]. Finally, instead of being an ancestral specimen of the Catarrhini, *Aegyptopithecus* has been suggested as a sister taxon to Catarrhini, which would lead to an even older calibration time for this node [46]. Moreover, this time is particularly known to have poor fossil records, and dating of the Catarrhini crown group has been difficult [63].

On the other hand, the direct mutation rate estimation could have produced overestimated divergence times for the Catarrhini node age compared to previous estimates [43,44] because the mutation rate and generation time might change cross-species and over time. It is possible that the Catarrhini ancestor could have had a faster yearly mutation rate and/or a shorter generation time than the recent macaques. Because fossil calibration could underestimate real divergence times, molecular-based methods could overestimate it, especially by assuming a unique mutation rate to an entire clade [40]. Allowing an increase in mutation rate back in time can reconcile the different methods to estimate divergence time between species.

To obtain more confidence in the estimation of divergence time, it would be necessary to have an accurate estimation of the mutation rate for various species. The estimates available today for primates vary from 0.81 × 10^−8^ per site per generation for the owl monkey (*A. nancymaae*) to 1.66 × 10^−8^ per site per generation for orangutan (*P. abelii*). However, the different methods and sequencing depth make it difficult to compare between species and attribute differences to biological causes or methodological ones. Therefore, more standardized methods in future studies would be needed to allow for cross-species comparison.

## Methods

### Samples

Whole-blood samples (2 mL) in EDTA (ethylenediaminetetraacetic acid) were collected from Indian rhesus macaques (*Macaca mulatta*) during routine health checks at the Biomedical Primate Research Centre (BPRC, Rijswijk, Netherlands). Individuals originated from 2 groups, with 1 or 2 reproductive males per group. After ensuring relatedness with a test based on individual genotypes [64], we ended up with 19 trios formed by 33 individuals and 2 extended trios (for which a second generation was available). In our dataset males reproduced from age 10.0 to 14.5 years (male reproductive range: 4.5 years), and females from 3.5 to 15.7 years (female reproductive range: 12.2 years). Genomic DNA was extracted using the DNeasy Blood and Tissue Kit (Qiagen, Valencia, CA, USA) following the manufacturer's instructions. BGIseq libraries were built in China National GeneBank (CNGB), Shenzhen, China. The mean insert size of the samples was 230 bp. Whole-genome pair-end sequencing was performed on BGISEQ500 platform, with a read length of 2 × 100 bp. The mean coverage of the raw sequences before trimming was 81× per sample (se = 1.35). Whole-genome sequences have been deposited in NCBI with BioProject No. PRJNA588178 and SRA submission SUB6522592.

### Read mapping, SNP calling, and filtering pipeline

Adaptors, low-quality reads, and N-reads were removed with SOAPnuke filter [65]. Trimmed reads were mapped to the reference genome of rhesus macaque Mmul 8.0.1 using BWA-MEM version 0.7.15 with the estimated insert size option [66]. Only reads mapping uniquely were kept, and duplicates were removed using Picard MarkDuplicates 2.7.1. The mean coverage after mapping was 76× per individual (se = 1.16). Variants were called using GATK 4.0.7.0 [67]; calling variants for each individual was performed with HaplotypeCaller in BP-RESOLUTION mode; all gVCF files per sample were combined into a single one per trio using CombineGVCFs per autosomal chromosomes; and finally joint genotyping was applied with GenotypeGVCF. Because *de novo* mutations are rare events, variant quality score recalibration (VQSR) is not a suitable tool to filter the sites as *de novo* mutations are more likely to be filtered out as low-quality variants. Instead we used a site filtering with the following parameters: QD < 2.0, FS > 20.0, MQ < 40.0, MQRankSum < –2.0, MQRankSum > 4.0, ReadPosRankSum < –3.0, ReadPosRankSum > 3.0, and SOR > 3.0. These filters were chosen by first running the pipeline with the site filters recommended by GATK (QD < 2.0; FS > 60.0; MQ < 40.0; MQRankSum < −12.5; ReadPosRankSum < −8.0; SOR > 3.0) and then doing a manual curation of the candidate *de novo* mutations on IGV [68]. Finally, we identified the common parameters within the apparent false-positive calls and decided to adjust the site filter to remove as many false-positive calls as possible without losing many true-positive calls (see the pipeline Supplementary Fig. S8 and the scripts on GitHub [69] and Zenodo [70]).

### Detection of *de novo* mutations

The combination of high coverage (76×) and stringent filters reduced false-positive results (calling a *de novo* mutation while it is not there). Thus, for each trio, we applied the following filters using R 3.5.1:

- Mendelian violations were selected using GATK SelectVariant (–mendelian-violation) and refined to only keep sites where both parents were homozygote reference (HomRef) and their offspring was heterozygote (Het).
- In the case of a *de novo*mutation, the number of alternative alleles seen in the offspring should account for ∼50% of the reads. Our allelic balance filter allowed the alternative allele to be present in 30%–70% of the total number of reads (applying the same 30% cut-off as in other studies [11,15,71]; Supplementary Fig. S9).
- The depth of the 3 individuals was filtered to be between 0.5 × *m*_depth_ and 2 × *m*_depth_, with *m*_depth_ being the mean depth of the trio. Most of the Mendelian violations are due to sequencing errors in regions of low sequencing depth; therefore, we applied a stricter threshold on the minimum depth to avoid the peak of Mendelian violations around 20× (Supplementary Fig. S10).
- Finally, after analyzing each trio with different genotype quality (GQ) cut-off (from 10 to 90), we set up a filter on the GQ of 60 to ensure the genotypes of the HomRef parents and the Het offspring (Supplementary Fig. S11).

From 242,922,329 autosomal SNPs (mean of 12,785,386 per trio), 2,251,363 were potential Mendelian violations found by GATK (mean of 118,493 per trio), 177,227 were filtered Mendelian violations with parents HomRef and offspring Het (mean of 9,328 per trio) (a), 78,339 passed the allelic balance filter (mean of 4,123 per trio) (b), 13,251 passed the depth filter (mean of 697 per trio) (c), and 744 the genotype quality filter (mean of 39 per trio) (d) (see Supplementary Table S4 for details on each individual). We also remove sites where a *de novo* mutation was shared among non-related individuals (1 site shared among 4 unrelated individuals). This allowed us to detect the number of *de novo* mutations observed per trio called *m*. We manually checked the read mapping quality for all *de novo* mutation sites in IGV, and we found possible false-positive calls in 10.89% of the sites for which the variant was absent from the offspring or also present in a parent (see Fig. S1). We compared the manual curation methods on the reads before realignment with a manual curation on the realigned reads outputted by GATK HaplotypeCaller. The manual curation on the realigned reads led to a lower false-positive rate of 6.72% instead of 10.89% and a 5% higher per generation rate than the rate estimated with manual curation before realignment. This difference is rather small and within the confidence interval of our estimated rate. Moreover, 47 of the 50 false-positive candidates found with the manual curation after realignment were also detected in the manual curation method before realignment. However, the latter had a larger set of potential false-positive candidates. Thus, in the absence of objective filters, we decided to use a conservative strategy and keep all sites but corrected the number of mutations for each trio with a false-positive rate (*β* = 0.1089) according to the manual curation before alignment (see equation 1). The 81 false-positive candidates were removed for downstream pattern analysis. We experimentally validated the *de novo* candidates from the trio Noot (father), Platina (mother), and Lithium (offspring). Primers were designed for 39 candidates (Supplementary Table S5). PCR amplification and Sanger sequencing were conducted on each individual (protocol in Supplementary Appendix 1 materials). On 24 sites the PCR amplification and sequencing returned high-quality results for all 3 individuals. A candidate was considered validated when both parents showed homozygosity for the reference allele and the offspring showed heterozygosity (Supplementary Fig. S2). All sequences generated for the PCR validation have been deposited in Genbank with accession Nos. MT426016–MT426087 (Supplementary Table S5).

### Estimation of the mutation rate per site per generation

From the number of *de novo* mutations to an estimate of the mutation rate per site per generation, it is necessary to also correct for false-negative calls (not calling a true *de novo* mutation as such). To do so, we estimated 2 parameters: the false-negative rate and the number of callable sites, *C*, i.e., the number of sites in the genome where we would be able to call a *de novo* mutation if it was there. We used the BP_RESOLUTION option in GATK to call variants for each position and thus get the exact genotype quality for each site in each individual—also sites that are not polymorphic. Thus, we do not have to rely on sequencing depth as a proxy for genotype quality at those sites. Instead, we can apply the same genotype quality threshold to the non-polymorphic sites as we do for *de novo* mutation candidate sites. This should lead to a more accurate estimate of the number of callable sites. For each trio, *C* is the sum of all sites where both parents are HomRef and the 3 individuals passed the depth filter (b) and the genotype quality filter (d). To correct for our last filter, the allelic balance (c), we estimated the false-negative rate *α*, defined as the proportion of true heterozygote sites (1 parent HomRef, the other parent HomAlt, and their offspring Het) outside the allelic balance threshold (Supplementary Fig. S9). We also implemented in this parameter the false-negative rate of the site filters following a normal distribution (FS, MQRankSum, and ReadPosRankSum). For all trios combined, the rate of false-negative calls caused by the allele balance filter and the site filters was 0.0402. The mutation rate per site per generation can then be estimated per trio with the following equation:

(1) \begin{eqnarray*}
\mu = {m \times (1 - \beta) \over (1 - \alpha) \times 2 \times C}
\end{eqnarray*}

To validate our pipeline we analyzed a trio of chimpanzees with a previously published estimated rate at 1.27 × 10^−8^  *de novo* mutations per site per generation [27]. We applied the exact same pipeline and found 54 *de novo* candidate mutations for this trio, a callable genome of 1,966,477,569 bp, and a false-negative rate of 4.6%. The callability represented only 64% of the total genome, which was lower than the rhesus macaques' callability (∼88% of the total genome). This is mainly due to the difference in depth between the parents (∼35× coverage) and the offspring (∼45× coverage) in the chimpanzee trio, leading to more filtering when using the mean depth of all individuals as a depth filter. When exploring the bam file for manual curation we identified 7 candidates as possible false-positive candidates. Removing these candidates to calculate a rate led to 1.25 × 10^−8^  *de novo* mutations per site per generation. On the other hand, keeping those candidates and applying the same false-positive rate as for the macaque trio of *β* = 0.1089 led to an estimated rate of 1.28 × 10^−8^  *de novo* mutations per site per generation. In either case, our analysis resulted in a rate similar to that previously estimated [27].

### Sex bias, ages, and relatedness

*De novo* mutations were phased to their parental origin using the read-backed phasing method described by Maretty et al. 2017 (script available on GitHub: [72]) [13]. The method uses read-pairs that contain both a *de novo* mutation and another heterozygous variant, the latter of which was used to determine the parental origin of the mutation if it is present in both offspring and 1 of the parents. The phasing allowed us to identify any parental bias in the contribution of the *de novo* mutations. A Pearson correlation test was performed between the mutation rate and the ages of each parent, as well as a linear regression model for father and mother independently. A multiple linear regression model was performed to predict the mutation rate from both parental ages as predictor variables. The phased mutations were used to dissociate the effect of the parental age from one another. Because the total number of SNPs phased to the mother or the father may differ, we divided the phased *de novo* mutations found in a parent by the total SNPs phased to this parent. Only a subset of the *de novo* mutations in an offspring was phased. Thus, we applied the paternal to maternal ratio to the total number of mutations in a trio, referred to as “upscaled” number of mutations, to predict the number of total mutations given by each parent at different ages. The 2 extended trios, analyzed as independent trios, also allowed us to determine whether ∼50% of the *de novo* mutations observed in the first trio were passed on to the next generation.

### Characterization of *de novo* mutations

From all the *de novo* mutations found, the type of mutations and their frequencies were estimated. For the mutations from a C to any base we determined whether they were followed by a G to detect the CpG sites (similarly if G mutations were preceded by a C). We defined a cluster as a window of 20,000 bp, similarly to Besenbacher et al. [37], and qualify how many mutations were clustered together: over all individuals, looking at related individuals, and within individuals. We simulated 663 mutations following a uniform distribution (runif function in R) to compare with our dataset. We investigated the mutations that are shared between related individuals. Finally, we looked at the location of mutations in the coding region using the annotation of the Mmul_8.0.1 reference genome from Ensembl.

### Molecular dating using the new mutation rate

We calculated the effective population size using the Watterson estimator θ = 4*N_e_*µ [73]. We estimated *θ* with the nucleotide diversity π = 0.00247 according to a recent population study [33]. Thus, we calculated the effective population size as *N_e_* = π/(4µ), with µ the mutation rate per site per generation estimated in our study. To calculate divergence time, we converted the mutation rate to a yearly rate based on the regression model of the number of mutations given by each parent regarding their ages and the mean callability C = 2,351,302,179. Given the maturation time and the high mortality due to predation, we assumed a mean age of reproduction in the wild at 10 years for females and 12 years for males and a generation time of 11 years, also reported in another study [33]. Thus, the yearly mutation rate was:

(2) \begin{eqnarray*}
&{\mu} _{\mathrm{yearly}} = \frac{4.6497 + 0.3042 \times \mathrm{age_{maternal}} + 4.8399 + 1.8364 \times \mathrm{age_{paternal}}\,\, \times \,\,\left( {1\,\, - \,\,\beta } \right)}{{\left( {1\,\, - \,\,\alpha } \right)\,\, \times \,\,2\,\, \times \,\,C}}
\end{eqnarray*}

The divergence time between species was then calculated using *T*_divergence_ = branch length macaque/µ_yearly_, with the branch length calculated from the whole-genome comparison [39] and µ_yearly_ the yearly mutation rate of rhesus macaques. We also used the confidence interval at 95% of our mutation rate regression to compute the confidence interval on divergence time. Based on the coalescent theory [42], the time to coalescence is 2*N_e_G* with *G* the generation time and *N_e_* the ancestral effective population size, assumed constant over time, as shown in a previous study [33]. Thus, we dated the speciation event as previously done by Besenbacher et al. [27] with:

(3) \begin{equation*} {T_{\mathrm{speciation}}} = {T_{\mathrm{divergence}}} - {\mathrm{ }}2{\mathrm{ }} \times {N_{e{\mathrm{\ }}\mathrm{ancestor}}} \times G.\,\, \end{equation*}

## Availability of Source Code and Requirements

Project name: Germline mutation rate

Project home page: https://github.com/lucieabergeron/germline_mutation_rate

Programming language: Python and Bash

Licence: MIT

## Data Availability

Whole-genome sequences underlying this article are available in NCBI and can be accessed with BioProject No. PRJNA588178 and SRA submission SUB6522592. All sequences generated for the PCR validation are available in Genbank and can be accessed with accession Nos. MT426016–MT426087. The analysis pipeline and scripts are available via Github [69] and Zenodo [70]. Other supporting data are available via the *GigaScience* database GigaDB [74].

## Additional Files

**Supplementary Figure S1**.Manual curation of the *de novo* mutations. (a) An example of *de novo* mutation that passed the manual curation and (b) an example of *de novo* mutation that did not pass the manual curation.

**Supplementary Figure S2**.PCR-sequencing chromatograms for the 24 *de novo* candidates that were successfully amplified for all 3 individuals, i.e., father (Noot), mother (Platina), and offspring (Lithium). For each alignment, the candidate *de novo* position on the reference genome of rheMac8 is indicated with an underscore and highlighted in black background at the F, M, O sequences. The order of the colored letters (forward or reverse) in each chromatogram indicates the primer used for sequencing. The *de novo* candidate that was not validated is presented in the bottom grey box. Due to the repetitive bases we provide both forward and reverse sequencing results for the mother and father.

**Supplementary Figure S3**.Poisson regression on the proportion of *de novo* mutation given by each parent applied to the total number of mutations phased (upscaled phased mutations). nb_paternal = *e*^2.48 + 0.07 × age paternal^ and nb_maternal = *e*^1.62 + 0.04 ^×^ age maternal^.

**Supplementary Figure S4**.Regression comparison. (a) Correlation between the expected mutation rate calculated with the first regression with the age of the parents for each trio and the observed rate (*r* = 0.66, *P* = 0.002). (b) Correlation between the expected mutation rate based on the second regression and the observed rate (*r* = 0.65, *P* = 0.002). The expected rates were calculated on the same dataset that served to build the regressions.

**Supplementary Figure S5**.Correlation between parental ages.

**Supplementary Figure S6**.Location of the 685 *de novo* mutations along the genome.

**Supplementary Figure S7**.Distance between mutations. (a) Number of mutations per cluster (<20,000 bp) within individuals (purple), between related individuals (green), and between non-related individuals (orange). (b) Distribution of the distance between mutations in a cluster; clusters involving non-related individuals are mainly observed in larger distances (> 10,000 bp) (Fisher exact test between non-related and other *P* = 2.6 × 10^−5^).

**Supplementary Figure S8. P**ipeline from fastq file to mutation rate estimation. The major steps are (1) mapping, (2) post-mapping processing, (3) variant calling, (4) *de novo* mutation detection, and (5) mutation rate estimation. All the scripts are available on Github: https://github.com/lucieabergeron/germline_mutation_rate.

**Supplementary Figure S9**.Allelic balances. (a) Distribution of allelic balance (number of reads supporting the alternative allele/total number of reads) for all true heterozygotes and (b) all candidate *de novo* mutations with all filters except the allelic balance, showing a large portion of somatic mutation or sequencing errors around 0.2. (c) The *de novo* mutations after all filters shows a normal distribution around 0.5.

**Supplementary Figure S10**. Mean depth distribution of Mendelian violations for each trio. Dark grey shade corresponds to the range of mean depth for the 19 trios and light grey shade corresponds to the minimum 0.5*m*_depth_ and maximum 2*m*_depth_ range of the depth filter.

**Supplementary Figure S11**. Variation of the number of *de novo* mutations, number of callable sites, and mutation rate with different genotype quality threshold. Red indicates the mean of the 19 trios.

**Supplementary Table S1**. Information for each trio on pedigrees, parental ages, and *de novo* mutations.

**Supplementary Table S2**. Position of the 663 *de novo* mutations used for all analyses.

**Supplementary Table S3**. Position of the clustered mutations.

**Supplementary Table S4**. Number of candidates after each filter.

**Supplementary Table S5**.Primers used for PCR validation and sequencing of *de novo* candidates for each individual, i.e., F: father (Noot), M: mother (Platina), and O: offspring (Lithium), along with sequences’ ID and corresponding Genbank accession numbers.

**Supplementary Appendix 1**.PCR amplification and sequencing validation of *de novo* candidates.

**Supplementary Appendix 2**.Bam files of the 81 manually curated *de novo* candidates (in the following order and for each panel with the father on the top, the mother in the middle, and the offspring in the bottom).

## Abbreviations

bp: base pairs; BWA: Burrows-Wheeler Aligner; GATK: Genomic Analysis Toolkit; GQ: genotype quality; IGV: Integrative Genome Viewer; Mb: megabase pairs; NCBI: National Center for Biotechnology Information; SNP: single-nucleotide polymorphism; SRA: Sequence Read Archive.

## Ethics Statement

Samples were provided from collaborators for research that was undertaken at the Natural History Museum of Denmark, permit 2020–12-7186–00733 from the Danish Ministry of Environment and Food.

## Competing Interests

The authors declare that they have no competing interests.

## Funding

This project was supported by a Carlsberg Foundation Grant to G.Z. (CF16–0663), Strategic Priority Research Program of the Chinese Academy of Sciences (XDB13000000), and ERC Consolidator grant 681396 Extinction Genomics. L.A.B. was supported by the Carlsberg Foundation.

## Authors’ Contributions

G.Z., M.H.S., S.B., and L.A.B. conceived this work. J.B. provided the samples. L.A.B., J.Z., P.L., G.P., M.H.S.S., and M.T.P.G. participated in extraction, library preparation, and sequencing. M.K. planned and executed the experimental validation. L.A.B. and S.B. built the analysis pipelines and conducted all the analyses. L.A.B., G.Z., S.B., and M.H.S. wrote the manuscript with the input of all co-authors. G.Z. supervised this project.

## Supplementary Material

giab029_GIGA-D-20-00280_Original_Submission

giab029_GIGA-D-20-00280_Revision_1

giab029_GIGA-D-20-00280_Revision_2

giab029_Response_to_Reviewer_Comments_Original_Submission

giab029_Response_to_Reviewer_Comments_Revision_1

giab029_Reviewer_1_Report_Original_Submission Susanne Pfeifer -- 11/23/2020 Reviewed

giab029_Reviewer_1_Report_Revision_1 Susanne Pfeifer -- 2/1/2021 Reviewed

giab029_Reviewer_2_Report_Original_Submission Jeffrey Rogers, Ph.D. -- 11/30/2020 Reviewed

giab029_Reviewer_2_Report_Revision_1 Jeffrey Rogers, Ph.D. -- 1/23/2021 Reviewed

giab029_Supplemental_File
