# Supplementary material for: The germline mutational process in rhesus macaque and its implications for phylogenetic dating
Source: Gigascience. 2021 May 5;10(5):giab029. doi: 10.1093/gigascience/giab029 (PMC8099771; doi:10.1093/gigascience/giab029)
Supplement: giab029_GIGA-D-20-00280_Original_Submission [file giab029_giga-d-20-00280_original_submission.pdf]

GigaScience

The germline mutational process in rhesus macaque and its implications for phylogenetic dating

--Manuscript Draft--

**Manuscript Number:**	GIGA-D-20-00280
**Full Title:**	The germline mutational process in rhesus macaque and its implications for phylogenetic dating
**Article Type:**	Research
**Funding Information:**	Carlsbergfondet (CF16-0663) — Dr. Guojie Zhang<br>Strategic Priority Research Program of the Chinese Academy of Sciences (XDB13000000) — Dr. Guojie Zhang<br>European Research Council (Consolidator grant) (681396 Extinction Genomics) — Dr. M. Thomas P. Gilbert
**Abstract:**	Background<br>Understanding the rate and pattern of germline mutations is of fundamental importance for understanding evolutionary processes.<br>Results<br>Here we analyzed 19 parent-offspring trios of rhesus macaques (*Macaca mulatta*) at high sequencing coverage of ca. 76X per individual, and estimated an average rate of 0.77×10^{-8} *de novo* mutations per site per generation (95 % CI: 0.69×10^{-8} - 0.85×10^{-8}). . By phasing 50 % of the mutations to parental origins, we found that the mutation rate is positively correlated with the paternal age. The paternal lineage contributed an average of 81 % of the *de novo* mutations, with a trend of an increasing male contribution for older fathers. About 3.5 % of *de novo* mutations were shared between siblings, with no parental bias, suggesting that they arose from early development (postzygotic) stages. Finally, the divergence times between closely related primates calculated based on the yearly mutation rate of rhesus macaque generally reconcile with divergence estimated with molecular clock methods, except for the Cercopithecidae/Hominoidea molecular divergence dated at 52 Mya using our new estimate of the yearly mutation rate.<br>Conclusions<br>When compared to the traditional molecular clock methods, new estimated rates from pedigree samples can provide insights into the evolution of well-studied groups such as primates.
**Corresponding Author:**	Lucie Adrienne Bergeron<br>Københavns Universitet<br>COPENHAGEN, Copenhagen DENMARK
**Corresponding Author Secondary Information:**	
**Corresponding Author's Institution:**	Københavns Universitet
**Corresponding Author's Secondary Institution:**	
**First Author:**	Lucie A. Bergeron
**First Author Secondary Information:**	
**Order of Authors:**	Lucie A. Bergeron
	Søren Besenbacher
	Jaco Bakker
	Jiao Zheng
	Panyi Li

Powered by Editorial Manager® and ProduXion Manager® from Aries Systems Corporation

	George Pacheco
	Mikkel-Holger S. Sinding
	Maria Kamilari
	M. Thomas P. Gilbert
	Mikkel H. Schierup
	Guojie Zhang
**Order of Authors Secondary Information:**	
**Additional Information:**	
**Question**	**Response**
Are you submitting this manuscript to a special series or article collection?	No
**Experimental design and statistics**<br><br>Full details of the experimental design and statistical methods used should be given in the Methods section, as detailed in our Minimum Standards Reporting Checklist. Information essential to interpreting the data presented should be made available in the figure legends.<br><br>Have you included all the information requested in your manuscript?	Yes
**Resources**<br><br>A description of all resources used, including antibodies, cell lines, animals and software tools, with enough information to allow them to be uniquely identified, should be included in the Methods section. Authors are strongly encouraged to cite Research Resource Identifiers (RRIDs) for antibodies, model organisms and tools, where possible.<br><br>Have you included the information requested as detailed in our Minimum Standards Reporting Checklist?	Yes
**Availability of data and materials**<br><br>All datasets and code on which the	Yes

conclusions of the paper rely must be either included in your submission or deposited in publicly available repositories (where available and ethically appropriate), referencing such data using a unique identifier in the references and in the "Availability of Data and Materials" section of your manuscript.<br><br>Have you have met the above requirement as detailed in our Minimum Standards Reporting Checklist?	

Powered by Editorial Manager® and ProduXion Manager® from Aries Systems Corporation

Manuscript

Click here to access/download;Manuscript;Rhesus_mutation_rate_manuscri

The germline mutational process in rhesus macaque and its implications for phylogenetic dating

Lucie A. Bergeron [1*], Søren Besenbacher [2], Jaco Bakker [3], Jiao Zheng [4,5], Panyi Li [4], George Pacheco [6], Mikkel-Holger S. Sinding [7,8], Maria Kamilari [1], M. Thomas P. Gilbert [6,9], Mikkel H. Schierup [10] and Guojie Zhang [1,4,11,12*]

[1] Section for Ecology and Evolution, Department of Biology, University of Copenhagen, Copenhagen, Denmark

[2] Department of Molecular Medicine, Aarhus University, Aarhus, Denmark

[3] Animal Science Department, Biomedical Primate Research Centre, Rijswijk, Netherlands

[4] BGI-Shenzhen, Shenzhen 518083, Guangdong, China

[5] BGI Education Center, University of Chinese Academy of Sciences, Shenzhen 518083, Guangdong, China

[6] Section for Evolutionary Genomics, The GLOBE Institute, University of Copenhagen, Copenhagen, Denmark

[7] Trinity College Dublin, Dublin, Ireland

[8] Greenland Institute of Natural Resources, Nuuk, Greenland

[9] Department of Natural History, NTNU University Museum, Norwegian University of Science and Technology (NTNU), NO-7491 Trondheim, Norway

[10] Bioinformatics Research Centre, Aarhus University, Aarhus, Denmark

[11] State Key Laboratory of Genetic Resources and Evolution, Kunming Institute of Zoology, Chinese Academy of Sciences, Kunming 650223, China

[12] Center for Excellence in Animal Evolution and Genetics, Chinese Academy of Sciences, Kunming 650223, China

[*] Corresponding author

E-mail: guojie.zhang@bio.ku.dk or lucie.a.bergeron@gmail.com

Abstract

Background

Understanding the rate and pattern of germline mutations is of fundamental importance for understanding evolutionary processes.

Results

Here we analyzed 19 parent-offspring trios of rhesus macaques (*Macaca mulatta*) at high sequencing coverage of ca. 76X per individual, and estimated an average rate of 0.77×10^{-8} *de novo* mutations per site per generation (95 % CI: 0.69×10^{-8} - 0.85×10^{-8}). By phasing 50 % of the mutations to parental origins, we found that the mutation rate is positively correlated with the paternal age. The paternal lineage contributed an average of 81 % of the *de novo* mutations, with a trend of an increasing male contribution for older fathers. About 3.5 % of *de novo* mutations were shared between siblings, with no parental bias, suggesting that they arose from early development (postzygotic) stages. Finally, the divergence times between closely related primates calculated based on the yearly mutation rate of rhesus macaque generally reconcile with divergence estimated with molecular clock methods, except for the Cercopithecidae/Hominoidea molecular divergence dated at 52 Mya using our new estimate of the yearly mutation rate.

Conclusions

When compared to the traditional molecular clock methods, new estimated rates from pedigree samples can provide insights into the evolution of well-studied groups such as primates.

Keywords

Evolution, mutation rate, primates, phylogeny

Background

Germline mutations are the source of heritable disease and evolutionary adaptation. Thus, having precise estimates of germline mutation rates is of fundamental importance for many fields in biology, including searching for *de novo* disease mutations [1,2], inferring demographic events [3,4], and accurate dating of species divergence times [5–7]. Over the past ten years, new

sequencing techniques have allowed deep sequencing of individuals from the same pedigree, enabling direct estimation of the *de novo* mutation rate for each generation, and precise estimation of the individual parental contributions to germline mutations across the whole genome. Most such studies have been conducted on humans, using large pedigrees with up to 3000 trios [8,9], leading to a consensus estimate of ~1.25 x 10^{-8} *de novo* mutation per site per generation, with an average parental age of ~ 29 years, leading to a yearly rate of 0.43 x 10^{-9} *de novo* mutation per site per year and most variation between trios explained by the age of the parents [8,10–17].

The observed increases in the mutation rate with paternal age in humans and other primates [8,18,19] has generally been attributed to errors during replication [20,21]. In mammalian spermatogenesis, primordial germ cells go through meiotic divisions, to produce stem cells by the time of puberty. After this time, stem cell divisions occur continuously throughout the male lifetime. Thus, human spermatogonial stem cells have undergone 100 to 150 mitoses in a 20 years old male, and ~ 610 mitoses in a 40 years old male [1], leading to an additional 1.51 *de novo* mutations per year increase in the father's age [8]. Female age also seems to affect the mutation rate in humans, with 0.37 mutations added per year [8]. This maternal effect cannot be attributed to replication errors, as different from spermatogenesis, female oocytogenesis occurs during embryogenesis process and is already finished before birth [22]. Moreover, there seems to be a bias towards males in contribution to *de novo* mutations, as the paternal to maternal contribution is 4:1 in human and chimpanzee [8,18]. One recent study proposed that damage-induced mutations might be a potential explanation for the observation of both the maternal age effect and the male-bias also present in parents reproducing right after puberty when replication mutations should not have accumulated yet in the male germline [23]. Parent-offspring analyses can also be used to distinguish mutations that are caused by gametogenesis from mutations that emerge in postzygotic stages [24,25]. While germline mutations in humans are relatively well studied, it remains unknown how much variability exists among primates on the contribution of replication errors to *de novo* mutations, the parental effects, and the developmental stages at which these mutations are established (postzygotic or gametogenesis).

Up until now, the germline mutation rate has only been estimated using pedigrees in few non-

human primate species, including chimpanzee (*Pan troglodytes*) [18,26,27], gorilla (*Gorilla gorilla*) [27], orangutan (*Pongo abelii*) [27], African green monkey (*Chlorocebus sabaeus*) [28], owl monkey (*Aotus nancymaae*) [19] and recently rhesus macaque (*Macaca mulatta*)[29]. The mutation rate of baboon (*Papio anubis*) [30] and grey mouse lemur (*Microcebus murinus*) [31] have also been estimated in preprinted studies. To precisely call *de novo* mutations in the offspring, collecting and comparing the genomic information of the pedigrees is the first essential step for detecting mutations only present in offspring but not in either parent. Next, the *de novo* mutations need to be separated from sequencing errors or somatic mutations, which cause false-positive calls. Because mutations are rare events, detecting *de novo* mutations that occur within a single generation requires high sequencing coverage to cover a majority of genomic regions and identify the false-positives. Furthermore, the algorithms used to estimate the mutation rate should take false-negative calls into account. However, a considerable range of sequencing depth (ranging from 18X [28] to 120X [26]) has been applied in many studies for estimation of mutation rate. Different filtering methods have been introduced to reduce false-positives and false-negatives but the lack of standardized methodology makes it difficult to assess whether differences in mutation rate estimates are caused by technical or biological variability. In addition, most studies on non-human primates used small pedigrees with less than ten trios, which made it difficult to detect any statistically significant patterns over *de novo* mutation spectra.

Studying non-human primates could help us understanding whether the mutation rate is affected by life-history traits such as mating strategies or the age of reproduction. The variation in mutation rate among primates will also be useful for re-calibrating the speciation times across lineages. The sister group of Hominoidea is Cercopithecidae, including the important biomedical model species, rhesus macaque (*Macaca mulatta*), which share 93 % of its genome with humans [32]. This species has a generation time estimate of ~ 11 years [33], and their sexual maturity is much earlier than in humans with females reaching maturity around three years old, while males mature around the age of 4 years [34]. While female macaques generally start reproducing right after maturation, males rarely reproduce in the wild until they reach their adult body size, at approximately eight years old [35]. They are also a promiscuous species, and

do not form pair bonds, but reproduce with multiple individuals. These life-history traits, along with their status as the closest related outgroup species of the hominoid group, make the rhesus macaque an interesting species for investigating the differences and common features in mutation rate processes across primates.

In this study, we produced high depth sequencing data for 33 rhesus macaque individuals (76X per individual) representing 19 trios. This particular dataset consists of a large number of trios, each with high coverage sequencing, and allowed us to test different filter criteria and choose the most appropriate ones to estimate the species mutation rate with high confidence. With a large number of *de novo* mutations phased to their parents of origins, we can statistically assess the parental contribution and the effect of the parental age. We characterize the type of mutations and their location on the genome to detect clusters and shared mutations between siblings. Finally, we use our new estimate to infer the effective population size and date their divergence time from closely related primate species.

Results

Estimation of mutation rate for 19 trios of rhesus macaques

To produce an accurate estimate for the germline mutation rate of rhesus macaques, we generated high coverage (76 X per individual after mapping, min 64 X, max 86 X) genome sequencing data for 19 trios of two unrelated families (Fig. 1). The first family consisted of two reproductive males and four reproductive females, and the second family had one reproductive male and seven reproductive females. In the first family, the pedigree extended over a third generation in two cases. The promiscuous mating system of rhesus macaques allowed us to follow the mutation rates in various ages of reproduction, and compare numerous full siblings and half-siblings.

We developed a pipeline for single nucleotide polymorphisms (SNP) calling with multiple quality control steps involving the filtering of reads and sites (see Methods). For each trio, we considered candidate sites as *de novo* mutations when i) both parents were homozygotes for the reference allele, while the offspring was heterozygous with 30 % to 70 % of its reads supporting

the alternative allele, and ii) the three individuals passed the depth and genotype quality filters (see Methods). These filters were calibrated to ensure a low rate of false-positives among the candidate *de novo* mutations.

We obtained an unfiltered set of 12,785,386 average candidate autosomal SNPs per trio (se = 26,196), of which a total of 177,227 were potential Mendelian violations (average of 9,328 per trio; se = 106). Of these, 744 SNPs passed the filters as *de novo* mutations, ranging from 25 to 59 for each trio and an average of 39 *de novo* mutations per trio (se = 2) (see Supplementary Table S1). We manually curated all mutations using IGV on bam files and found that 663 mutations convincingly displayed as true positives. This leaves a maximum of 10.89 % (81 sites) that could be false-positives due to the absence of the variant in the offspring or presence of the variant in the parents (see Supplementary Fig. S1 and the 81 curated mutations in supplementary). Most of those sites were in dinucleotide repeat regions or short tandem repeats (56 sites), while others were in non-repetitive regions of the genome (25 sites). The manual curation may have missed the realignment executed during variant calling. Thus, in the absence of objective filters, we decided to keep these regions in the estimate of mutation rate but corrected the number of mutations for each trio with a false-positive rate (see equation 1 in Methods section).

To confirm the authenticity of the *de novo* mutations, we performed PCR experiments for all candidate *de novo* mutations from one trio before manual correction. We designed primers to a set of 39 *de novo* candidates among which 3 *de novo* mutations assigned as spurious from the manual inspection. Of these, 24 sites were successfully amplified and sequenced for all three individuals i.e mother, father, and offspring, including 1 of the spurious sites. Among those sequenced sites, 23 were correct, only one was wrong (Supplementary Fig. S2). This invalidated candidate was the spurious candidate removed by manual curation, therefore supporting our manual curation method. The PCR validation results suggested a lower false-positive rate of 4.2 % before manual curation. As the PCR validation was done only on 24 candidates we decided to keep a strict false-positive rate of 10.89 % found by manual curation.

We then estimated the mutation rate, per site per generation, as the number of mutations

observed, and corrected for false-positive calls, divided by the number of callable sites. The number if callable sites for each trio ranged from 2,334,764,487 to 2,359,040,186, covering on average 88 % of the autosomal sites of the rhesus macaque genome. A site was defined as callable when both parents were homozygotes for the reference allele, and all individuals passed the depth and genotype quality filters at that site. As callability is determined using the base-pair resolution vcf file, containing every single site of the genome, all filters used during calling were taken into account during the estimation of callability, except for the site filters and the allelic balance filter. We then corrected for false-negative rates, calculated as the number of "good" sites that could be filtered away by both the site filters and allelic balance filters - estimated at 4.02 % (see equation 1 in Methods section). Another method to estimate the false-negative rate is to simulate mutations on the bam files and evaluate the detection rate after passing through all filters. On 552 randomly simulated mutations among the 19 offsprings, 545 were detected as *de novo* mutations, resulting in a false-negative rate of 1.27 %. The 7 remaining mutations were filtered away by the allelic balance filter only, which can be explained by the reads filtering in the variant calling step. This result might be underestimated due to the methodological limitation of simulating *de novo* mutations, yet, it ensures that a false-negative rate of 4.02 % is not out of range. Thus, the final estimated average mutation rate of the rhesus macaques was 0.77×10^{-8} *de novo* mutations per site per generation (95 % CI 0.69×10^{-8} - 0.85×10^{-8}). We removed the 81 sites that, based on manual curation, could represent false-positive calls from the following analyses (see the 663 *de novo* mutations in Supplementary Table S2).

Parental contribution and age impact to the *de novo* mutation rate

We observed a positive correlation between the paternal age and the mutation rate in the offspring (adjusted $R^2 = 0.23$; $P = 0.021$; regression: $\mu = 1.022 \times 10^{-9} + 5.393 \times 10^{-10} \times age_{paternal}$; $P = 0.021$; Fig. 2a). We also detected a slight positive correlation with the maternal age, though not significant (adjusted $R^2 = 0.09$; $P = 0.111$; regression: $\mu = 6.200 \times 10^{-9} + 1.818 \times 10^{-10} \times age_{maternal}$; P = 0.111; Fig. 2b). A multiple regression of the mutation rate on paternal and

maternal age resulted in this formula: $\mu_{Rhesus} = 1.355\times10^{-9} + 7.936\times10^{-11} \times age_{maternal} + 4.588\times 10^{-10} \times age_{paternal}$ (P = 0.06), where μ_{Rhesus} is the mutation rate for the species.

We were able to phase 337 mutations to their parent of origin, which accounted for more than half of the total number of *de novo* mutations (663). There is a significant male bias in the contribution of *de novo* mutations, with an average of 80.6 % paternal *de novo* mutations (95 % CI 76.6 % - 84.6 %; T = 22.62, DF = 36, $P < 2.2 \times 10^{-16}$; Fig. 2c). Moreover, with more than half of the *de novo* mutations phased to their parent of origin, we were able to disentangle the effect of the age of each parent on mutation rate independently (Fig. 2d). By assuming that the ratio of mutations phased to a particular parent was the same in the phased mutations than in the unphased ones, we could predict the total number of mutations given by each parent. For instance, if an offspring had 40 *de novo* mutations and only half were phased, with 80 % given from its father, we would apply this ratio to the total number of mutations in this offspring, ending up with 32 *de novo* mutations from its father and eight from its mother. This analysis suggested a stronger male age effect to the number of mutations (adjusted $R^2 = 0.41$, P = 0.002), and a similar, non significant maternal age effect (adjusted $R^2 = -0.01$, P = 0.38). The two regression lines meet around the age of sexual maturity (3 years for females and 4 years for males), which is consistent with a similar accumulation of *de novo* mutations during the developmental process from birth to sexual maturity in both sexes, but the variances on the regression line slopes are large (see Fig. 2c and Supplementary Fig. S3 for the same analysis with a Poisson regression). Using these two linear regressions, we can predict the number of *de novo* mutations in the offspring based on the age of each parent at the time of reproduction: $nb\ of\ mutations_{Rhesus} = 4.6497 + 0.3042\times age_{maternal} + 4.8399 + 1.8364 \times age_{paternal}$, where $nb\ of\ mutations_{Rhesus}$ is the number of *de novo* mutations for the given trio. The expected mutation rates calculated using the two different regression models show similar correlations with the observed mutation rate ($R^2 = 0.54$, P = 0.016 for the first regression and $R^2 = 0.54$, P = 0.016 for the upscaled one, see Supplementary Fig. S4). However, on the first regression on the mutation rate, the maternal age effect may be confounded by the paternal age, as maternal and paternal age are correlated in our dataset, yet, non-significantly ($R^2 = 0.38$, P = 0.106, see Supplementary Fig. S5). The upscaled regression unravels the effect of the parental age independently from

each other. This regression can also be used to infer the contribution of each parent at different reproductive age. For instance, if both parents reproduce at 5 years old, based on the upscaled regression, the father is estimated to give ~ 14 *de novo* mutations (95 % CI:6 - 22) and the mother ~ 6 *de novo* mutations (95 % CI:3 - 10), corresponding to a contribution ratio from father to mother of 2.3:1 at 5 years old. If they reproduce at 15 years old, this ratio would be 3.6:1 with males giving ~ 32 *de novo* mutations (95 % CI: 29 – 36) and females ~ 9 *de novo* mutations (95 % CI: 4 – 14). It seems that the male bias increases with the parental age, yet, our model was based on too few data points in early male reproductive ages to reach a firm conclusion. For the two extended trios for which a second generation is available, we looked at the proportion of *de novo* mutations in the first offspring that were passed on to the third generation - the third generation inherited a heterozygote genotype with the alternative allele being the *de novo* mutation. In one case, 66 % of the *de novo* mutations in the female (Heineken) were passed to her daughter (Hoegaarde), while in another case, 40 % of the *de novo* mutations in the female (Amber) were passed to her son (Magenta). These deviations from the expected 50 % inheritance rate are not statistically significant (Binomial test; $P_{Hoegaarde} = 0.14$ and $P_{Magenta} = 0.27$).

Characterizations of *de novo* mutations

We characterized the type of *de novo* mutations and found that transition from a strong base to weak base ($G > A$ and $C > T$) were most common (332/663), with 43 % of those mutations located in CpG sites (Fig. 3a). In total, 23.2 % (154/663) of the *de novo* mutations were located in CpG sites. This is slightly higher than what has been found in humans, for which 19 % of the *de novo* mutations are in CpG sites [11], but not significantly (human: $X^2 = 2.774$, df = 1, P = 0.096). Moreover, 32.1 % (144/448) of the transition mutations ($A > G$ and $C > T$) were in CpG sites, higher than what has been found in chimpanzee, with 24 % of the transition *de novo* mutations in CpG sites [18]. The transition to transversion ratio (ti/tv) was 2.08, which is similar to the ratio observed in other species (human: ti/tv ~ 2.16 [36]; human ti/tv ~ 2.2 [17]; chimpanzee: ti/tv ~ 1.98 [26]. The 663 *de novo* mutations showed some clustering in the genome (Fig. 3b and Supplementary Fig. S6). Across all trios, we observed 11 clusters, defined as

windows of 20,000 bp where more than one mutation occurred in any individual, involving 23 mutations. Four clusters were made of mutations from a single individual, accounting for eight mutations (Fig. 3b). Overall, 3.47 % of the *de novo* mutations were located in clusters, and 1.21 % were mutations within the same individual located in a cluster, which is significantly lower than the 3.1 % reported in humans [37] ($X^2 = 7.35$, DF = 1, P = 0.007; Supplementary Fig. S7, Supplementary Table S3). We observed 23 mutations occurring recurrently in more than one related individual (Table 1), which accounted for 3.5 % of the total number of *de novo* mutations (23/663) and 1.5 % of sites (10/650 unique sites). Four *de novo* mutations (2 sites) were shared between half-siblings on the maternal side, and 19 (8 sites) were shared between half-siblings on the paternal side. However, there was no significant difference between the proportion of mutations shared between pairs of individuals related on the maternal side (9 pairs, 0.70 % shared), and pairs related on their paternal side (53 pairs, 0.80 % shared; Fisher's exact test P = 1). In 6 sites, the phasing to the parent of origin confirmed that the mutation was coming from the common parent for at least one individual (Table 1). Moreover, the phasing was never inconsistent by attributing a shared *de novo* mutation to the other parent than the parent in common. However, 5 shared sites did appear as mosaic in the common parent, with a maximum of 5 % of the reads of the father supporting the alternative allele (4 out of 80 reads). Nine of the *de novo* mutations (1.4 % of the total *de novo* mutations) were located in coding sequences (CDS regions), which is close to the overall proportion of coding sequences region (1.2%) in the whole macaque genome. Eigth out of those eight mutations were non-synonymous.

Table 1 – Six mutations shared between related individuals.

Chrom	Position	Ref	Alt	Sibling 1	Phasing [a]	Sibling 2	Phasing	Sibling 3	Phasing	Sibling 4	Phasing	Common parent	Name parent
chr2	101979137	G	A	Khan	P	Delta	U					father	Smack
chr6	132663101	A	T	Amber	U	Babet	M					mother	Mayke
chr7	60635102	G	T	Sir	U	Honoria	U					father	Smack
chr7	116648579	G	A	Amber	M	Babet	U					mother	Mayke
chr9	32544257	C	T	Hoegaarde	U	Djembe	U	Babet	U	Magenta	U	father	Poseidon
chr10	65163492	G	A	Khan	P	Delta	P					father	Smack
chr15	35463257	C	T	Leffe	U	Babet	U	Magenta	U			father	Poseidon
chr17	88174686	C	A	Hoegaarde	P	Babet	P					father	Poseidon
chr19	7047030	C	T	Leffe	U	Djembe	U					father	Poseidon
chr19	15861061	C	T	Bavaria	P	Lithium	U					father	Noot

a: P: paternal; M: maternal; U: unphased

Molecular dating with trio-based mutation rate

Based on our inferred mutation rate and the genetic diversity of Indian rhesus macaques (π = 0.00247) estimated using whole genomic sequencing data from more than 120 unrelated wild individuals [33], we calculated the effective population size (N_e) of rhesus macaques to be 79,874. This is similar to the N_e = 80,000 estimated previously using $\mu = 0.59\times10^{-8}$ from hippocampal transcriptome and H3K4me3-marked DNA regions from 14 individuals [38], yet higher than N_e = 61,800 estimated using $\mu = 1\times10^{-8}$ with 120 individual full genome data [33]. Assuming a generation time of 11 years and an average reproduction age of 10 years for females and 12 years for males, the yearly mutation rate of rhesus macaques was calculated based on our regression model of the number of mutations given by males and females independently, and the average callability (see equation 2 in the Methods section). As captive animals usually reproduce later than in the wild, which could impact the average mutation rate per generation, we used the regression instead of the mutation rate per generation to correct for this possible bias. The yearly mutation rate of rhesus macaques in our calculation was 0.62×10^{-9} per site per year, almost 1.5 times that of humans [8].

Given a precise evolutionary mutation rate is essential for accurate calibration of molecular divergence events between species, we used the mutation rate we inferred for rhesus macaques to re-date the phylogeny of closely-related primate species with full genome alignment available [39] (Fig. 4a). The molecular divergence time (T_D) is the time since an ancestral lineage started to split into two descendant lineages, and can be inferred from the genetic divergence between the two descendant lineages and the mutation rate. The speciation time (T_S) is a younger event that implies no more gene flow between lineages [40]. On the backward direction, the alleles of two descendant lineages are randomly sampled from their parents until going back to the most recent common ancestor [41]. This stochastic event, known as the coalescent, depends on the population sizes, being slower in a large population [42]. Thus, from the divergence time, the speciation time can be inferred given the rate of coalescence (see equation 3 in the Method section). We also compared our results to those of previous dating attempts based on molecular phylogenetic trees calibrated with fossils records (Fig. 4b). We found that the two methods concur for the most recent events. Specifically, we estimated that the *Macaca mulatta* and *Macaca*

fascicularis genomes had already diverged around 3.90 million years ago (Mya) (95 % CI: 3.46 – 4.46), which is slightly older than previous estimates using the molecular clock calibrated with fossils, as the molecular divergence of the two species has been estimated at 3.44 Mya with mitochondrial data [43] and 3.53 Mya from nuclear data [44]. We estimated a speciation event between the two species 2.14 Mya after the coalescent time, also consistent with previous findings of a most common recent ancestor to the two populations of the rhesus macaque, the Chinese and the Indian population, around 1.94 Mya based on coalescent simulations [45]. For the next node, the molecular clock seems to differ between mitochondrial and nuclear data, as the divergence time for the Papionini group into the *Papio* and *Macaca* genera has been estimated to 8.13 Mya using nuclear data [44], and 12.17 Mya with mitochondrial data [43]. We estimated a divergence time between these two genera of 13.17 Mya (95 % CI: 11.70 – 15.07). For earlier divergence events, our estimated divergence times are more ancient than previous reports. For instance, we estimated that the Cercopithecini and Papionini diverged 19.86 Mya (95 % CI: 17.64 – 22.71), while other studies had calculated 11.55 Mya using nuclear data [44], and 14.09 Mya using mitochondrial data [43]. Finally, the divergence between Cercopithecidae and Hominoidea has been reported between 25 and 30 Mya [39,46], with an estimation of 31.6 Mya using the nuclear molecular clock [44] and 32.12 Mya using the mitochondrial one [43]. Our dating of the divergence time between the Cercopithecidae and Hominoidea of 52.31 Mya (95 % CI: 46.47 – 59.83) is substantially older than previous estimates. However, the estimated speciation time inferred based on the ancestral population size, suggested a speciation of the Catarrhini group into two lineages 44.50 Mya (Fig. 4b).

Discussion

Despite many efforts to accurately estimate direct *de novo* mutation rates, it is still a challenging task due to the rare occurrence of *de novo* mutations, and the small sample size that is often available. Sequencing coverage is known to be a significant factor in affecting false-positive (FP), and false-negative (FN) calls when detecting *de novo* mutation [1,26]. A minimal sequencing coverage at 15X was recommended for SNPs calling [47]. However, such coverage cannot provide sufficient power to reduce FPs because the lower depth threshold

cannot preclude Mendelian violations due to sequencing errors. Moreover, a larger portion of the genome would be removed in the denominator at low depth in order to reduce the FN. While most studies on direct estimation of mutation rate use 35-40X coverage [8,19,27], their methods to reduce FP and FN differ. Some studies use the deviation from 50 % of the *de novo* mutation pass to the next generation to infer the false-positive rate [8,19]. Others use probabilistic methods to access the callability [27], or simulation of known mutation to control the pipeline quality [28]. Differences in methods likely impact the calculated rate. Here, we produced sequences at 76X coverage, which allows us to apply conservative filtering processes, while still obtaining high coverage (88 %) of the autosomal genome region when inferring *de novo* mutations. To our knowledge, only one other study has used very high coverage (120X per individuals), on a single trio of chimpanzees [26]. Such high coverage allowed us to achieve a false-positive rate below 10.89 % and within the regions we deemed callable, we calculated a low false-negative rate of 4.02 %.

Our estimated rate is higher than the 0.58×10^{-8} *de novo* mutations per site per generation estimated in a recent study [29]. The difference should be mainly attributed to the fact that they sequenced the offspring of younger parents (average parental age of 7.1 years for females and 7.8 years for males compared to 8.4 years for females and 12.4 years for males in this study). Using our regression from the phased mutation, we estimated a mutation rate of 0.51×10^{-8} per site per generation, when males reproduce at 7.8 years and females reproduce at 7.1 years old. Moreover, using their regression based on the age of puberty and the increase of paternal mutation per year, Wang and collaborators estimated a per generation rate of 0.71×10^{-8} mutations when males reproduce at 11 years, and a yearly rate of 0.65×10^{-9} mutations per site per year, which is approx 5 % higher than our estimate of 0.62×10^{-9} [29]. This difference may be due to any combination of stochasticity, differences in *de novo* mutation rate pipelines (callability estimate, false-negative rate, and false-positive rate estimate) and different models for converting pedigree estimates to yearly rates. Our combination of high coverage data and a large number of trios allowed us to gain high confidence estimates of the germline mutation rate of rhesus macaques at around 0.77×10^{-8} *de novo* mutation per site per generation, ranging from 0.49×10^{-8} to 1.16×10^{-8}. This is similar to the mutation rate

estimated for other non-Hominidae primates; 0.81×10^{-8} for the owl monkey (*Aotus nancymaae*) [19] and 0.94×10^{-8} for the African green monkey (*Chlorocebus sabaeus*) [28], while all Hominidae seem to have a mutation rate that is higher than 1×10^{-8} *de novo* mutation per site per generation [8,27]. However, if we count for the *de novo* mutation per site per year, the rate of rhesus macaque (0.62×10^{-9}) is almost 1.5-fold the human one of 0.43×10^{-9} mutation per sites per year [8].

One of the main factors affecting the mutation rate within the species is the paternal age at the time of reproduction, which was attributed to the accumulation of replication-driven mutations during spermatogenesis [20,21,48], and has been observed in many other primates [8,13,18,19,27]. In rhesus macaques, the rate at which germline mutation increases with paternal age seems faster than in humans; we inferred 1.84 mutations more per year for the rhesus macaque father (95% CI 0.77 – 2.90 for an average callable genome of 2.35 Mb), compared to 1.51 in humans (95% CI 1.45–1.57 for an average callable genome of 2.72 Mb) [8]. For females, there is less difference, with 0.30 more mutations per year for the mother in rhesus macaque (95% CI -0.41 – 1.02), and 0.37 more per year in human mothers (95% CI 0.32–0.43) [8]. In rhesus macaques, males produce a larger number of sperm cells per unit of time (23×10^{6} sperm cells per gram of testis per day [49]) than humans (4.4×10^{6} sperm cells per gram of testis per day [50]). This could imply a higher number of cell division per unit of time in rhesus macaques and thus more replication error during spermatogenesis. This is also consistent with the generation time effect which stipulates that an increase in generation time would decrease the number of cell division per unit of time as well as the yearly mutation rate assuming that most mutations arise from replication errors [21,24,51–54]. Indeed, humans have a generation time of 29 years, while it is 11 years for rhesus macaques. Another explanation for a higher increase of mutation rate with paternal age could be differences in the replication machinery itself. Due to higher sperm competition in rhesus macaque, the replication might be under selective pressure for fast production at the expense of replication fidelity, leading to less DNA repair mechanisms. As in other primates, we found a male bias in the contribution of *de novo* mutations, as the paternal to maternal ratio is 4.2:1.This ratio is higher than the 2.7:1 ratio observed in mice [55] and slightly higher than the 4:1 ratio observed

in humans [55–57]. Similarly to the wild, the males of our dataset reproduced from 10 years old, which did not allow us to examine if the contribution bias was also present just after maturation. Moreover, the promiscuous behavior of rhesus macaque leads to father reproducing with younger females. Using our model to compare the contribution of each parent reproducing at the similar age, it seems that the male bias increases with the parental age, with a lower difference in contribution at the time of sexual maturation (2.3:1 for parents of 5 years old) and an increase in male to female contribution with older parents (3.6:1 for parents of 15 years old). This result differs from humans, where the male bias seems constant over time [23], but more time points in macaque would be needed to interpret the contribution over time. In rhesus macaques, the ratio of paternal to maternal contribution to the shared mutations between related individuals is 1:1, similarly to what has been shown in mice [55], highlighting that those mutations probably occur during primordial germ cell divisions in postzygotic stages. Our study shows many shared patterns in the *de novo* mutations among non-Hominid primates. More estimation of mammals could help understanding if these features are conserved across a broad phylogenetic scale. Moreover, further work would be needed to understand if some gamete production stages are more mutagenic in some species than others.

An accurate estimation of the mutation rate is essential for the precise dating of species divergence events. We used the rhesus macaque mutation rate to estimate its divergence time with related species for which whole-genome alignments are already available and their molecular divergence times have been investigated before with other methods [39]. The results of our direct dating method, based on molecular distances between species and *de novo* mutation rate, matched those of traditional molecular clock approaches for speciation events within 10 to 15 million years. However, it often produced earlier divergence times for more ancient nodes than the molecular clock method. This incongruence might be attributed to the fossils that were used for calibration with the clock method, which has many limitations [7,40,58]. A fossil used for calibrating a node is usually selected to represent the oldest known specimen of a lineage. Still, it cannot be known if real even older specimens existed [58]. Thus, a fossil is usually assumed to be younger than the real divergence time of the species [59]. Moreover, despite the

error associated with the dating of a fossil itself, determining its position on a tree can be challenging and have effects on the inferred ages across the whole tree [7,40]. For instance, the Catarrhini node, marking the divergence between the Cercopithecidae and the Hominoidea, is often calibrated in primate phylogenies [58]. This node has been calibrated to approx. 25 Mya using the oldest known Cercopithecidae fossil (*Victoriapithecus*), and the oldest known Hominoidea fossil (*Proconsul*), both around 22 My old [60]. However, if the oldest Catarrhini fossil (*Aegyptopithecus*) of 33 to 34 My age is used, this node could also be calibrated to 35 Mya [46]. Finally, instead of being an ancestral specimen of the Catarrhini, *Aegyptopithecus* has been suggested as a sister taxon to Catarrhini, which would lead to an even older calibration time for this node [46].

On the other hand, the direct mutation rate estimation could have produced overestimated divergence times for the Catarrhini node age compared to previous estimates [43,44], because the mutation rate and generation time might change cross-species and over time. It is possible that the Catarrhini ancestor would have had a faster yearly mutation rate, and/or a shorter generation time than the recent macaques. Since fossil calibration could underestimate real divergence times, molecular-based methods could overestimate it, especially by assuming a unique mutation rate to an entire clade [40].

To obtain more confidence in the estimation of divergence time, it would be necessary to have an accurate estimation of the mutation rate for various species. The estimates available today for primates vary from 0.81×10^{-8} per site per generation for the Owl monkey (*Aotus nancymaae*) to 1.66×10^{-8} per site per generation for Orangutan (*Pongo abelii*). However, the different methods and sequencing depth make it difficult to compare between species and attribute differences to biological causes or methodological ones. Therefore, more standardized methods in further studies would be needed to allow for cross-species comparison.

Methods

Samples

Whole blood samples (2 mL) in EDTA (Ethylenediaminetetraacetic acid) were collected from 53 Indian rhesus macaques (*Macaca mulatta*) during routine health checks at the Biomedical

Primate Research Centre (BPRC, Rijswijk, Netherlands). Individuals originated from two groups, with one or two reproductive males per group. After ensuring the relatedness with a test based on individual genotypes [61], we ended up with 19 trios formed by 33 individuals and two extended trios (for which a second generation was available). In our dataset males reproduced from 10 years old to 14.5 years old (♂ reproductive range: 4.5 years), and females from 3.5 years old to 15.7 years old (♀ reproductive range: 12.2 years). Genomic DNA was extracted using DNeasy Blood and Tissue Kit (Qiagen, Valencia-CA, USA) following the manufacturer's instructions. BGIseq libraries were built in China National GeneBank (CNGB), Shenzhen, China. The average insert size of the samples was 230 base pairs. Whole-genome pair-ended sequencing was performed on BGISEQ500 platform, with a read length of 2x100 bp. The average coverage of the raw sequences before trimming was 81X per sample (se = 1.35). Whole-genome sequences have been deposited in NCBI (National Center for Biotechnology Information) with BioProject number PRJNA588178 and SRA submission SUB6522592.

Reads mapping, SNPs calling, and filtering pipeline

Adaptors, low-quality reads, and N-reads were removed with SOAPnuke filter [62]. Trimmed reads were mapped to the reference genome of rhesus macaque Mmul 8.0.1 using BWA-MEM version 0.7.15 with the estimated insert size option. Only reads mapping uniquely were kept and duplicates were removed using Picard MarkDuplicates. The average coverage after mapping was 76X per individuals (se = 1.16). Variants were called using GATK 4.0.7.0 [63]; calling variants for each individual with HaplotypeCaller in BP-RESOLUTION mode; all gVCF files per sample were combined into a single one per trio using CombineGVCFs per autosomal chromosomes; finally joint genotyping was applied with GenotypeGVCF. Because *de novo* mutations are rare events, variant quality score recalibration (VQSR) is not a suitable tool to filter the sites as *de novo* mutations are more likely to be filtered out as low-quality variants. Instead we used a site filtering with the following parameters: QD < 2.0, FS > 20.0, MQ < 40.0, MQRankSum < - 2.0, MQRankSum > 4.0, ReadPosRankSum < - 3.0, ReadPosRankSum > 3.0 and SOR > 3.0. These filters were chosen by first, running the pipeline with the site filters recommended by GATK (QD < 2.0; FS > 60.0; MQ < 40.0; MQRankSum < -12.5;

ReadPosRankSum < -8.0 ; SOR > 3.0), then, doing a manual curation of the candidates *de novo* mutations on the Integrative Genome Viewer (IGV). Finally, we identified the common parameters within the apparent false-positive calls and decided to adjust the site filter to remove as many false-positives without losing much true positive calls (see the pipeline Supplementary Fig. S8 and the scripts on GitHub: https://github.com/lucieabergeron/germline_mutation_rate).

Detection of *de novo* mutations

The combination of high coverage (76X) and stringent filters reduced false-positive - calling a *de novo* mutation while it is not there. Thus, for each trio, we applied the following filters:

(a) Mendelian violations were selected using GATK SelectVariant and refined to only keep sites where both parents were homozygote reference (HomRef), and their offspring was heterozygote (Het).

(b) In the case of a *de novo* mutation, the number of alternative alleles seen in the offspring should account for ~ 50 % of the reads. Our allelic balance filter allowed the alternative allele to be present in 30 % to 70 % of the total number of reads (applying the same 30% cutoff as in other studies [11,15,64] (Supplementary Fig. S9).

(c) The depth of the three individuals was filtered to be between $0.5\times m_{depth}$ and $2\times m_{depth}$, with m_{depth} being the average depth of the trio. Most of the Mendelian violations are due to sequencing errors in regions of low sequencing depth; therefore, we applied a stricter threshold on the minimum depth to avoid the peak of Mendelian violations around 20X (Supplementary Fig. S10).

(d) Finally, after analyzing each trio with different genotype quality GQ cutoff (from 10 to 90), we set up a filter on the genotype quality of 60 to ensure the genotypes of the HomRef parents and the Het offspring (Supplementary Fig. S11).

From 242,922,329 autosomal SNPs (average of 12,785,386 per trio), 2,251,363 were potential Mendelian violations found by GATK (average of 118,493 per trio), 177,227 were filtered

Mendelian violations with parents HomRef and offspring Het (average of 9,328 per trio) (a), 78,339 passed the allelic balance filter (average of 4,123 per trio) (b), 13,251 passed the depth filter (average of 697 per trio) (c) and 744 the genotype quality filter (average of 39 per trio) (d) (see Supplementary Table S4 for details on each individual). We also remove sites where a *de novo* mutation was shared among non-related individuals (1 site shared between 4 unrelated individuals). This allowed us to detect the number of *de novo* mutations observed per trio called m. We manually checked the reads mapping quality for all *de novo* mutations sites in the Integrative Genome Viewer (IGV). And we found possible false-positive calls in 10.89 % of the sites for which the variant was absent from the offspring or also present in a parent (see Fig. S1). We kept those sites for the estimation of the mutation rate, and corrected for false-positive (β = 0.1089), but removed them for downstream pattern analysis. We experimentally validated the *de novo* candidates from the trio Noot (father), Platina (mother), and Lithium (offspring). Primers were designed for 39 candidates (Supplementary Table S5). PCR amplification and Sanger sequencing were conducted on each individual (protocol in Supplementary materials). On 24 sites the PCR amplification and sequencing returned high-quality results for all three individuals. A candidate was considered validated when both parents showed homozygosity for the reference allele and the offspring showed heterozygosity (Supplementary Fig. S2). All sequences generated for the PCR validation have been deposited in Genbank with accession numbers MT426016 - MT426087 (Supplementary Table S4).

Estimation of the mutation rate per site per generation

From the number of *de novo* mutations to an estimate of the mutation rate per site per generation, it is necessary to also correct for false-negatives - not calling a true *de novo* mutation as such. To do so, we estimated two parameters: the false-negative rate and the number of callable sites, *C*, ie. the number of sites in the genome where we would be able to call a *de novo* mutation if it was there. We used the BP_RESOLUTION option in GATK to call variants for each position and thus get the exact genotype quality for each site in each individual - also sites that are not polymorphic. So unlike other studies, we do not have to rely on sequencing depth as a proxy for genotype quality at those sites. Instead, we can apply the

same genotype quality threshold to the non-polymorphic sites as we do for *de novo* mutation candidate sites. This should lead to a more accurate estimate of the number of callable sites. For each trio, C is the sum of all sites where: both parents are HomRef, and the three individuals passed the depth filter (b) and the genotype quality filter (d). To correct for our last filter, the allelic balance (c), we estimated the false-negative rate α, defined as the proportion of true heterozygotes sites (one parent HomRef, the other parent HomAlt and their offspring Het) outside the allelic balance threshold (Supplementary Fig. S9). We also implemented in this parameter the false-negative rate of the site filters following a normal distribution (FS, MQRankSum, and ReadPosRankSum). For all trios combined, the rate of false-negatives caused by the allele balance filter and the site filters was 0.0402. To validate this false-negative rate estimation we also used a simulation method, used in other studies [28,65]. With BAMSurgeon [66], 552 mutations were simulated across the 19 trios at random callable sites. The false-negative rate was calculated as 1 – (number of detected mutations/number of simulated mutations), after running the pipeline from variant calling. The mutation rate per sites per generation can then be estimated per trio with the following equation:

$$\mu = \frac{m \times (1 - \beta)}{(1 - \alpha) \times 2 \times C} \tag{1}$$

Sex bias, ages, and relatedness

De novo mutations were phased to their parental origin using the read-backed phasing method described in Maretty et al. 2017 (script available on GitHub: https://github.com/besenbacher/POOHA) [13]. The method uses read-pairs that contain both a *de novo* mutation and another heterozygous variant, the latter of which was used to determine the parental origin of the mutation if it is present in both offspring and one of the parents. The phasing allowed us to identify any parental bias in the contribution of the *de novo* mutations. Pearson’s correlation test was performed between the mutation rate and ages of each parent, as well as a linear regression model for father and mother independently. A multiple linear

regression model was performed to predict the mutation rate from both parental ages as predictor variables. The phased mutations were used to dissociate the effect of the parental age from one another. Because the total number of SNPs phased to the mother or the father may differ, we divided the phased *de novo* mutations found in a parent by the total SNPs phased to this parent. Only a subset of the *de novo* mutations in an offspring was phased. Thus, we applied the paternal to maternal ratio to the total number of mutations in a trio, referred to as 'upscaled' number of mutations, to predict the number of total mutations given by each parent at different ages. The two extended trios, analyzed as independent trios, also allowed us to determine if ~ 50 % of the *de novo* mutations observed in the first trio were passed on to the next generation.

Characterization of *de novo* mutations

From all the *de novo* mutations found, the type of mutations and their frequencies were estimated. For the mutations from a C to any base we determined if they were followed by a G to detect the CpG sites (similarly if G mutations were preceded by a C). We defined a cluster as a window of 20,000 bp to qualify how many mutations were clustered together; over all individuals, looking at related individuals, and within individuals. We simulated 663 mutations following a uniform distribution to compare with our dataset. We investigated the mutations that are shared between related individuals. Finally, we looked at the location of mutations in the coding region using the annotation of the reference genome.

Molecular dating using the new mutation rate

We calculated the effective population size using Watterson's estimator $\theta = 4N_e\mu$ [67]. We estimated θ with the nucleotide diversity $\pi = 0.00247$ according to a recent population study [33]. Thus, we calculated the effective population size as $N_e = \frac{\pi}{4\mu}$ with μ the mutation rate per site per generation estimated in our study. To calculate divergence time, we converted the mutation rate to a yearly rate based on the regression model of the number of mutations given by each parent regarding their ages and the average callability C = 2,351,302,179. Given the maturation time and the high mortality due to predation, we assumed an average age of reproduction in the wild at 10 years old for females and 12 years old for males and a generation

time of 11 years, also reported in another study [33]. Thus, the yearly mutation rate was:

$$\mu = \frac{4.6497 + 0.3042 \times agematernal + 4.8399 + 1.8364 \times agepaternal \times (1 - \beta)}{(1 - \alpha) \times 2 \times C} \qquad (2)$$

The divergence time between species was then calculated using $T_{divergence} = \frac{d}{2\mu}$ with d the genetic distance between species which were calculated from the whole-genome comparison [39] and μ the yearly mutation rate of rhesus macaques. We also used the confidence interval at 95% of our mutation rate regression to compute the confidence interval on divergence time. Based on the coalescent theory [42], the time to coalescence is 2NeG with G the generation time and Ne the ancestral effective population size, assumed constant over time, as shown in a previous study [33]. Thus, we dated the speciation event as previously done by Besenbacher et al. 2019 [27] with:

$$T_{speciation} = T_{divergence} - 2 \times N_{e\ ancestor} \times G \qquad (3)$$

Figures

Figure 1. Pedigree of the 19 trios used for the direct estimation of mutation rate. a: The first group is composed of two reproductive males and four reproductive females. b: The second group contained one reproductive male and seven reproductive females. In each offspring, the color on the left corresponds to the paternal lineage and under the name are the age of the father (in blue) and mother (in red) at the time of reproduction. The reproductive ranges are 4.5 years for males and 12.2 years for females.

Figure 2. Parental contribution and age effect to the *de novo* mutation rate. a: There is a positive correlation between the mutation rate and the paternal age. b: The correlation between maternal age and mutation rate is not significant. c: Males contribute to 80.6 % of the *de novo* mutations while females contribute to 19.4 % of them. d: Upscaled number of *de novo* mutations given by each parent shows a similar contribution at the age of sexual maturation and a substantial increase with male age.

Figure 3. Characterizations of the *de novo* mutations. a: The type of *de novo* mutations in CpG and non-CpG sites. b: QQ-plot of the distance between *de novo* mutations compared to a uniform distribution within individuals (purple), between related individuals (green), and between non-related individuals

(orange).

Figure 4. Molecular dating with pedigree-based mutation rate. a: Primates phylogeny based on the yearly mutation rate (0.62×10^{-9} per site per year). In green are the confidence interval of our divergence time estimates (Td) and grey shades represent the time of speciation (Ts). The effective population sizes are indicated under the nodes (N_e Macaca ancestor is our estimate of N_e *Macaca mulatta* and N_e Catarrhini from the literature [68]). b: Comparison of our divergence time and speciation time with the previous estimation using the molecular clock from mitochondrial [43] and nuclear data [44] calibrated with fossils records.

Availability of source code and requirements

Project name: Germline mutation rate

Project home page: https://github.com/lucieabergeron/germline_mutation_rate

Programming language: Python and Bash

Availability of supporting data and materials

Whole-genome sequences have been deposited in NCBI (National Center for Biotechnology Information) with BioProject number PRJNA588178 and SRA submission SUB6522592 and will be available upon publication. All sequences generated for the PCR validation have been deposited in Genbank with accession numbers MT426016 - MT426087 and will be available upon publication.

Ethics Statement

Samples were provided from collaborators for research that was undertaken at the Natural History Museum of Denmark, permit 2020-12-7186-00733 from the Danish Ministry of Environment and Food.

Competing Interests

The authors declare that they have no competing interests.

Fundings

This project was supported by a Carlsberg Foundation Grant to GZ (CF16-0663), Strategic Priority Research Program of the Chinese Academy of Sciences (XDB13000000), and ERC Consolidator grant 681396 Extinction Genomics. LB was supported by Carlsberg Foundation.

Authors' contributions

G.Z., M.H.S., S.B. and L.B. conceived this work. J.B. provided the samples. L.B., J. Z., P.L., G.P., M.H.S.S, and M.T.P.G. participated in extraction, library preparation, and sequencing. MK planned and executed the experimental validation. L.B. and S.B. built the analyses pipelines and conducted all the analyses. L.B, G.Z, S.B, and M.H.S wrote this manuscript with the input of all co-authors. G.Z. supervised this project.

Acknowledgments

We would like to thank GenomeDK at Aarhus University for providing computational resources and supports to this study. We also thank Josefin Stiller for helpful comments on the manuscript.

Additional Files

Supplementary Figure S1 - Manual curation of the *de novo* mutations. a: an example of *de novo* mutation that passed the manual curation and b: an example of *de novo* mutation that did not pass the manual curation.

Supplementary Figure S2. PCR-Sequencing Chromatograms for the 24 *de novo* candidates that were successfully amplified for all three individuals ie. father (Noot), mother (Platina), and offspring (Lithium). For each alignment, the candidate *de novo* position on the reference genome of rheMac8 is indicated with an underscore and highlighted in black background at the F,M,O sequences. The order of the colored letters (forward or reverse) in each chromatogram indicates the primer used for sequencing. The *de novo* candidate that was not validated is presented in the bottom grey box. Due to the repetitive bases we provide both

forward and reverse sequencing results for the mother and father.

Supplementary Figure S3. Poisson regression on the proportion of *de novo* mutation given by each parent apply to the total number of mutation phased (upscaled phased mutation). $nb\_paternal = e^{2.48 + 0.07 \times age\_father}$ and $nb\_maternal = e^{1.62 + 0.04 \times age\_mother}$.

Supplementary Figure S4. Regression comparison. a: Correlation between the expected mutation rate calculated with the first regression with the age of the parents for each trio and the observed rate (r=0.66, p=0.002). b: Correlation between the expected mutation rate based on the second regression and the observed rate (r=0.65, p=0.002). The expected rates were calculated on the same dataset that served to build the regressions.

Supplementary Figure S5. Correlation between parental ages.

Supplementary Figure S6. Location of the 685 *de novo* mutations along the genome.

Supplementary Figure S7. Distance between mutation. a: Number of mutations per cluster (< 20000 bp) within individuals (purple), between related individuals (green), and between non-related individuals (orange). b: Distribution of the distance between mutations in a cluster, clusters involving non-related individuals are mainly observed in larger distances (> 10,000 bp) (Fisher's exact test between non-related and other $P = 2.6 \times 10^{-5}$).

Supplementary Figure S8 - Pipeline from fastq file to mutation rate estimation. The major steps are (1) mapping (2) post mapping processing (3) variant calling (4) *de novo* mutations detection and (5) mutation rate estimation. All the scripts are available on Github: https://github.com/lucieabergeron/germline_mutation_rate.

Supplementary Figure S9 – Allelic balances. a: Distribution of allelic balance (number of reads supporting the alternative allele/ total number of reads) for all true heterozygotes and b: all candidate *de novo* mutation with all filter except the allelic balance, showing a large portion of somatic mutation or sequencing errors around 0.2. c: The *de novo* mutation after all filter shows a normal distribution around 0.5.

Supplementary Figure S10 - Average depth distribution of Mendelian violations for each trio. Dark grey shade corresponds to the range of average depth for the 19 trios and light grey shade corresponds to the minimum $0.5m_{depth}$ and maximum $2m_{depth}$ range of the depth filter.

Supplementary Figure S11 - Variation of the number of *de novo* mutations, number of

callable sites, and mutation rate with different genotype quality threshold. In red the average of the 19 trios.

Supplementary Table S1 – Information for each trio on pedigrees, parental ages, and *de novo* mutations.

Supplementary Table S2 – Position of the 663 *de novo* mutations used for all analyses.

Supplementary Table S3 – Position of the clustered mutations.

Supplementary Table S4 – Number of candidates after each filter.

Supplementary Table S5: Primers used for PCR validation and sequencing of *de novo* candidates for each individual ie F: father (Noot), M: mother (Platina) and O: offspring (Lithium) along with sequences' ID and corresponding Genbank accession numbers.

Supplementary Appendix 1. PCR amplification and sequencing validation of *de novo* candidates.

Supplementary Appendix 2. Bam files of the 81 manually curated *de novo* candidates (in the following order and for each panel with the father on the top, the mother in the middle and the offspring in the bottom)

References

1. Acuna-Hidalgo R, Veltman JA, Hoischen A. New insights into the generation and role of de novo mutations in health and disease. Genome Biol. BioMed Central Ltd.;

2. Oliveira S, Cooper DN, Azevedo L. De Novo Mutations in Human Inherited Disease. *eLS*. John Wiley & Sons, Ltd;

3. Lapierre M, Lambert A, Achaz G. Accuracy of demographic inferences from the site frequency spectrum: The case of the yoruba population. *Genetics*. Genetics; 2017; doi: 10.1534/genetics.116.192708.

4. Zeng K, Jackson BC, Barton HJ. Methods for estimating demography and detecting between-locus differences in the effective population size and mutation rate. *Mol Biol Evol*. Oxford University Press; 2018; doi: 10.1093/molbev/msy212.

5. Teeling EC, Springer MS, Madsen O, Bates P, O'Brien SJ, Murphy WJ. A molecular phylogeny for bats illuminates biogeography and the fossil record. *Science (80-)*. American Association for the Advancement of Science; 2005; doi: 10.1126/science.1105113.

6. Ho SYW, Larson G. Molecular clocks: When timesare a-changin'. *Trends Genet*. Elsevier Ltd; 2006; doi: 10.1016/j.tig.2005.11.006.

7. Pulquério MJF, Nichols RA. Dates from the molecular clock: how wrong can we be? *Trends Ecol Evol*. 2007; doi: 10.1016/j.tree.2006.11.013.

8. Jónsson H, Sulem P, Kehr B, Kristmundsdottir S, Zink F, Hjartarson E, et al.. Parental influence on human germline de novo mutations in 1,548 trios from Iceland. *Nature*. Nature Publishing Group; 2017; doi: 10.1038/nature24018.

9. Halldorsson B V., Palsson G, Stefansson OA, Jonsson H, Hardarson MT, Eggertsson HP, et al.. Characterizing mutagenic effects of recombination through a sequence-level genetic map. *Science (80-)*. American Association for the Advancement of Science; 2019; doi: 10.1126/science.aau1043.

10. Awadalla P, Gauthier J, Myers RA, Casals F, Hamdan FF, Griffing AR, et al.. Direct measure of the de novo mutation rate in autism and schizophrenia cohorts. *Am J Hum Genet*. Cell Press; 2010; doi: 10.1016/j.ajhg.2010.07.019.

11. Besenbacher S, Liu S, Izarzugaza JMG, Grove J, Belling K, Bork-Jensen J, et al.. Novel variation and de novo mutation rates in population-wide de novo assembled Danish trios. *Nat Commun*. Nature Publishing Group; 2015; doi: 10.1038/ncomms6969.

12. Rahbari R, Wuster A, Lindsay SJ, Hardwick RJ, Alexandrov LB, Turki S Al, et al.. Timing, rates and spectra of human germline mutation. *Nat Genet*. 2016; doi: 10.1038/ng.3469.

13. Maretty L, Jensen JM, Petersen B, Sibbesen JA, Liu S, Villesen P, et al.. Sequencing and de novo assembly of 150 genomes from Denmark as a population reference. *Nature*. Nature Publishing Group; 2017; doi: 10.1038/nature23264.

14. Roach JC, Glusman G, Smit AFA, Huff CD, Hubley R, Shannon PT, et al.. Analysis of Genetic Inheritance in a Family Quartet by Whole Genome Sequencing. *Science (80-)*. 2010; doi: 10.1126/science.1186802.

15. Kong A, Frigge ML, Masson G, Besenbacher S, Sulem P, Magnusson G, et al.. Rate of de novo mutations and the importance of father's age to disease risk. *Nature*. 2012; doi: 10.1038/nature11396.

16. Neale BM, Devlin B, Boone BE, Levy SE, Lihm J, Buxbaum JD, et al.. Patterns and rates of exonic de novo mutations in autism spectrum disorders. *Nature*. 2012; doi: 10.1038/nature11011.

17. Wang H, Zhu X. De novo mutations discovered in 8 Mexican American families through whole genome sequencing. *BMC Proc*. 2014; doi: 10.1186/1753-6561-8-S1-S24.

18. Venn O, Turner I, Mathieson I, De Groot N, Bontrop R, McVean G. Strong male bias drives germline mutation in chimpanzees. *Science (80-)*. 2014; doi: 10.1126/science.344.6189.1272.

19. Thomas GWC, Wang RJ, Puri A, Rogers J, Radivojac P, Hahn MW, et al.. Reproductive Longevity Predicts Mutation Rates in Primates. *Curr Biol*. Elsevier Ltd.; 2018; doi: 10.1016/j.cub.2018.08.050.

20. Crow JF. The origins, patterns and implications of human spontaneous mutation. Nat. Rev. Genet. European Association for Cardio-Thoracic Surgery;

21. Li WH, Ellsworth DL, Krushkal J, Chang BHJ, Hewett-Emmett D. Rates of nucleotide substitution in primates and rodents and the generation-time effect hypothesis. *Mol Phylogenet Evol*. Academic Press

Inc.; 1996; doi: 10.1006/mpev.1996.0012.

22. Byskov AG. Differential of mammalian embryonic gonad. *Physiol Rev*. 1986; doi: 10.1152/physrev.1986.66.1.71.

23. Gao Z, Moorjani P, Sasani TA, Pedersen BS, Quinlan AR, Jorde LB, et al.. Overlooked roles of DNA damage and maternal age in generating human germline mutations. *Proc Natl Acad Sci U S A*. National Academy of Sciences; 2019; doi: 10.1073/pnas.1901259116.

24. Scally A. Mutation rates and the evolution of germline structure. *Philos Trans R Soc B Biol Sci*. 2016; doi: 10.1098/rstb.2015.0137.

25. Acuna-Hidalgo R, Bo T, Kwint MP, Van De Vorst M, Pinelli M, Veltman JA, et al.. Post-zygotic Point Mutations Are an Underrecognized Source of de Novo Genomic Variation. *Am J Hum Genet*. Cell Press; 2015; doi: 10.1016/j.ajhg.2015.05.008.

26. Tatsumoto S, Go Y, Fukuta K, Noguchi H, Hayakawa T, Tomonaga M, et al.. Direct estimation of de novo mutation rates in a chimpanzee parent-offspring trio by ultra-deep whole genome sequencing. *Sci Rep*. Nature Publishing Group; 2017; doi: 10.1038/s41598-017-13919-7.

27. Besenbacher S, Hvilsom C, Marques-Bonet T, Mailund T, Schierup MH. Direct estimation of mutations in great apes reconciles phylogenetic dating. *Nat Ecol Evol*. Nature Publishing Group; 2019; doi: 10.1038/s41559-018-0778-x.

28. Pfeifer SP. Direct estimate of the spontaneous germ line mutation rate in African green monkeys. *Evolution (N Y)*. Wiley/Blackwell (10.1111); 2017; doi: 10.1111/evo.13383.

29. Wang RJ, Thomas GWC, Raveendran M, Harris RA, Doddapaneni H, Muzny DM, et al.. Paternal age in rhesus macaques is positively associated with germline mutation accumulation but not with measures of offspring sociability. *Genome Res*. Cold Spring Harbor Laboratory Press; 2020; doi: 10.1101/gr.255174.119.

30. Wu FL, Strand A, Ober C, Wall JD, Moorjani P, Przeworski M. A comparison of humans and baboons suggests germline mutation rates do not track cell divisions. *bioRxiv*. Cold Spring Harbor Laboratory; 2019; doi: 10.1101/844910.

31. Campbell CR, Tiley GP, Poelstra JW, Hunnicutt KE, Larsen PA, dos Reis M, et al.. Pedigree-based measurement of the de novo mutation rate in the gray mouse lemur reveals a high mutation rate, few mutations in CpG sites, and a weak sex bias. *bioRxiv*. 2019; doi: 10.1101/724880.

32. Gibbs RA, Rogers J, Katze MG, Bumgarner R, Weinstock GM, Mardis ER, et al.. Evolutionary and biomedical insights from the rhesus macaque genome. *Science (80-)*. 316:222–342007;

33. Xue C, Raveendran M, Harris RA, Fawcett GL, Liu X, White S, et al.. The population genomics of rhesus macaques (Macaca mulatta) based on whole-genome sequences. *Genome Res*. Cold Spring Harbor Laboratory Press; 2016; doi: 10.1101/gr.204255.116.

34. Rawlins RG, Kessler MJ. The Cayo Santiago Macaques: History, Behavior, and Biology.

35. Bercovitch FB, Widdig A, Trefilov A, Kessler MJ, Berard JD, Schmidtke J, et al.. A longitudinal

study of age-specific reproductive output and body condition among male rhesus macaques, Macaca mulatta. *Naturwissenschaften*. 2003; doi: 10.1007/s00114-003-0436-1.

36. Yuen RKC, Merico D, Cao H, Pellecchia G, Alipanahi B, Thiruvahindrapuram B, et al.. Genome-wide characteristics of de novo mutations in autism. *npj Genomic Med*. Nature Publishing Group; 2016; doi: 10.1038/npjgenmed.2016.27.

37. Besenbacher S, Sulem P, Helgason A, Helgason H, Kristjansson H, Jonasdottir A, et al.. Multi-nucleotide de novo Mutations in Humans. Petrov DA, editor. *PLOS Genet*. Public Library of Science; 2016; doi: 10.1371/journal.pgen.1006315.

38. Yuan Q, Zhou Z, Lindell SG, Higley JD, Ferguson B, Thompson RC, et al.. The rhesus macaque is three times as diverse but more closely equivalent in damaging coding variation as compared to the human. *BMC Genet*. BioMed Central; 2012; doi: 10.1186/1471-2156-13-52.

39. Moorjani P, Amorim CEG, Arndt PF, Przeworski M. Variation in the molecular clock of primates. *Proc Natl Acad Sci U S A*. National Academy of Sciences; 2016; doi: 10.1073/pnas.1600374113.

40. Steiper ME, Young NM. Timing primate evolution: Lessons from the discordance between molecular and paleontological estimates. *Evol Anthropol*. 2008; doi: 10.1002/evan.20177.

41. Rosenberg NA, Nordborg M. Genealogical trees, coalescent theory and the analysis of genetic polymorphisms. Nat. Rev. Genet. Nature Publishing Group;

42. Kingman JFC. The coalescent. *Stoch Process their Appl*. North-Holland; 1982; doi: 10.1016/0304-4149(82)90011-4.

43. Pozzi L, Hodgson JA, Burrell AS, Sterner KN, Raaum RL, Disotell TR. Primate phylogenetic relationships and divergence dates inferred from complete mitochondrial genomes. *Mol Phylogenet Evol*. Academic Press Inc.; 2014; doi: 10.1016/j.ympev.2014.02.023.

44. Perelman P, Johnson WE, Roos C, Seuánez HN, Horvath JE, Moreira MAM, et al.. A Molecular Phylogeny of Living Primates. Brosius J, editor. *PLoS Genet*. Public Library of Science; 2011; doi: 10.1371/journal.pgen.1001342.

45. Hernandez RD, Hubisz MJ, Wheeler DA, Smith DG, Ferguson B, Rogers J, et al.. Demographic histories and patterns of linkage disequilibrium in Chinese and Indian rhesus macaques. *Science*. American Association for the Advancement of Science; 2007; doi: 10.1126/science.1140462.

46. Stewart C-B, Disotell TR. Primate evolution – in and out of Africa. *Curr Biol*. 1998; doi: 10.1016/S0960-9822(07)00367-3.

47. Song K, Li L, Zhang G. Coverage recommendation for genotyping analysis of highly heterologous species using next-generation sequencing technology. *Sci Rep*. Nature Publishing Group; 2016; doi: 10.1038/srep35736.

48. Drost JB, Lee WR. Biological basis of germline mutation: Comparisons of spontaneous germline mutation rates among drosophila, mouse, and human. *Environ Mol Mutagen*. Wiley-Blackwell; 1995; doi: 10.1002/em.2850250609.

49. Amann RP, Johnson L, Thompson DL, Pickett BW. Daily Spermatozoal Production, Epididymal Spermatozoal Reserves and Transit Time of Spermatozoa Through the Epididymis of the Rhesus Monkey. *Biol Reprod*. 1976; doi: 10.1095/biolreprod15.5.586.

50. Amann RP, Howards SS. Daily spermatozoal production and epididymal spermatozoal reserves of the human male. *J Urol*. 1980; doi: 10.1016/S0022-5347(17)55377-X.

51. Wu C-I, Lit W-H. Evolution evidence for higher rates of nucleotide substitution in rodents than in man. *Proc Nati Acad Sci USA*. 82:1741–51985;

52. Goodman MF, Creighton S, Bloom LB, Petruska J, Kunkel TA. Biochemical Basis of DNA Replication Fidelity. *Crit Rev Biochem Mol Biol*. 1993; doi: 10.3109/10409239309086792.

53. Ohta T. An examination of the generation-time effect on molecular evolution. *Proc Natl Acad Sci USA*. 90:10676–801993;

54. Ségurel L, Wyman MJ, Przeworski M. Determinants of Mutation Rate Variation in the Human Germline. *Annu Rev Genomics Hum Genet*. 2014; doi: 10.1146/annurev-genom-031714-125740.

55. Lindsay SJ, Rahbari R, Kaplanis J, Keane T, Hurles ME. Similarities and differences in patterns of germline mutation between mice and humans. *Nat Commun*. Springer Science and Business Media LLC; 2019; doi: 10.1038/s41467-019-12023-w.

56. Jónsson H, Sulem P, Arnadottir GA, Pálsson G, Eggertsson HP, Kristmundsdottir S, et al.. Multiple transmissions of de novo mutations in families. *Nat Genet*. Nature Publishing Group; 2018; doi: 10.1038/s41588-018-0259-9.

57. Goldmann JM, Wong WSW, Pinelli M, Farrah T, Bodian D, Stittrich AB, et al.. Parent-of-origin-specific signatures of de novo mutations. *Nat Genet*. 2016; doi: 10.1038/ng.3597.

58. Heads M. Dating nodes on molecular phylogenies: A critique of molecular biogeography. *Cladistics*. 2005; doi: 10.1111/j.1096-0031.2005.00052.x.

59. Benton MJ, Donoghue PCJ, Asher RJ, Friedman M, Near TJ, Vinther J. Constraints on the timescale of animal evolutionary history. Palaeontol. Electron. Texas A and M University;

60. Goodman M, Porter CA, Czelusniak J, Page SL, Schneider H, Shoshani J, et al.. Toward a Phylogenetic Classification of Primates Based on DNA Evidence Complemented by Fossil Evidence. *Mol Phylogenet Evol*. 1998; doi: 10.1006/mpev.1998.0495.

61. Manichaikul A, Mychaleckyj JC, Rich SS, Daly K, Sale M, Chen WM. Robust relationship inference in genome-wide association studies. *Bioinformatics*. 2010; doi: 10.1093/bioinformatics/btq559.

62. Chen Y, Chen Y, Shi C, Huang Z, Zhang Y, Li S, et al.. SOAPnuke: A MapReduce acceleration-supported software for integrated quality control and preprocessing of high-throughput sequencing data. *Gigascience*. Oxford University Press; 2017; doi: 10.1093/gigascience/gix120.

63. Poplin R, Ruano-Rubio V, DePristo MA, Fennell TJ, Carneiro MO, Auwera GA Van der, et al.. Scaling accurate genetic variant discovery to tens of thousands of samples. *bioRxiv*. 2018; doi: 10.1101/201178.

64. Francioli LC, Polak PP, Koren A, Menelaou A, Chun S, Renkens I. Genome-wide patterns and properties of de novo mutations in humans. *Nat Genet*. 2015; doi: 10.1038/ng.3292.

65. Keightley PD, Pinharanda A, Ness RW, Simpson F, Dasmahapatra KK, Mallet J, et al.. Estimation of the Spontaneous Mutation Rate in Heliconius melpomene. *Mol Biol Evol*. 2014; doi: 10.1093/molbev/msu302.

66. Ewing AD, Houlahan KE, Hu Y, Ellrott K, Caloian C, Yamaguchi TN, et al.. Combining tumor genome simulation with crowdsourcing to benchmark somatic single-nucleotide-variant detection. *Nat Methods*. Nature Publishing Group; 2015; doi: 10.1038/nmeth.3407.

67. Watterson GA. On the number of segregating sites in genetical models without recombination. *Theor Popul Biol*. 1975; doi: 10.1016/0040-5809(75)90020-9.

68. Schrago CG. The effective population sizes of the anthropoid ancestors of the human-chimpanzee lineage provide insights on the historical biogeography of the great apes. *Mol Biol Evol*. 2014; doi: 10.1093/molbev/mst191.

Figure 1

Click here to access/download;Figure;Rhesus_mutation_rate_figures_1.pdf

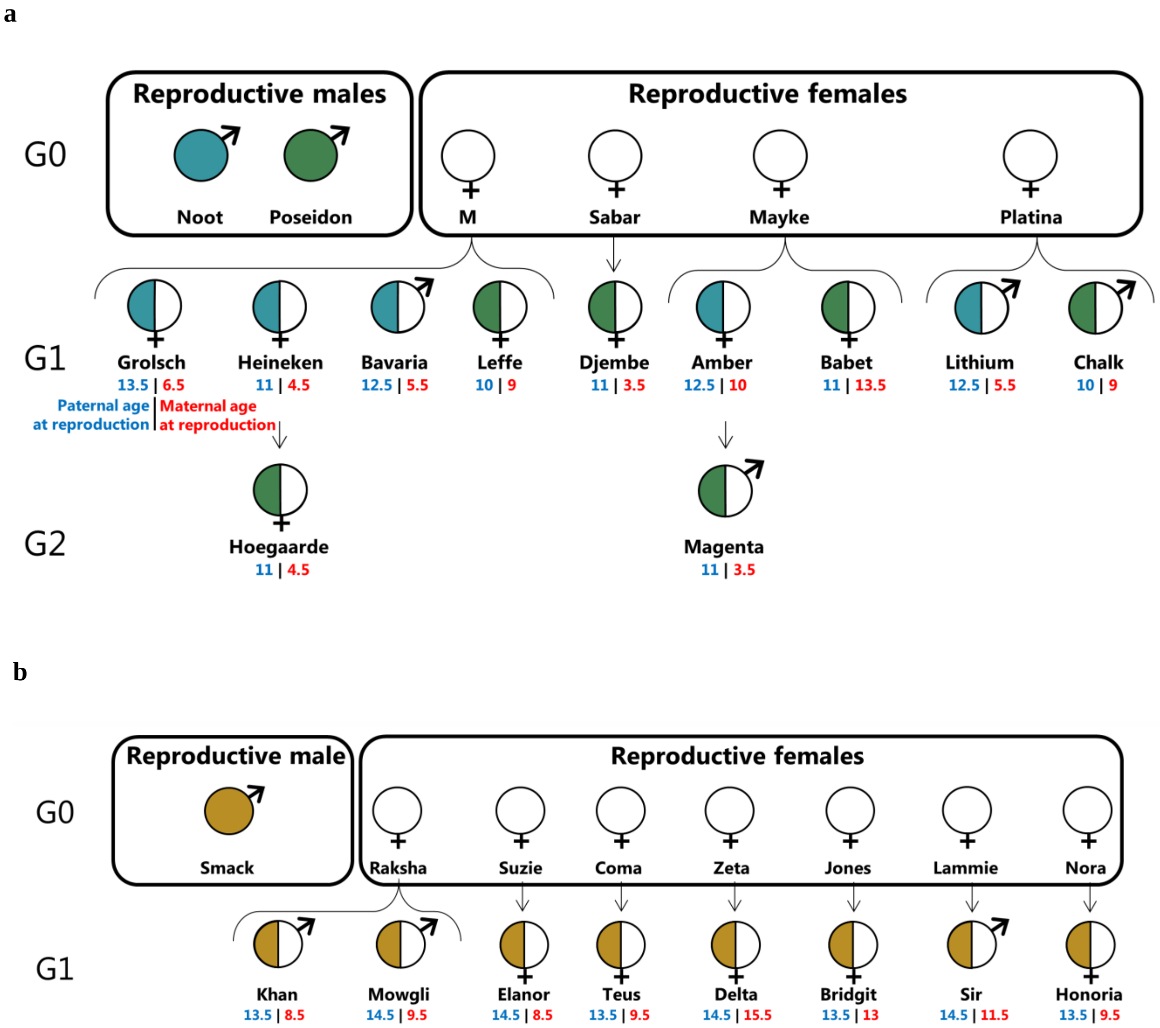

Figure 2

Click here to access/download;Figure;Rhesus_mutation_rate_figures_2.pdf

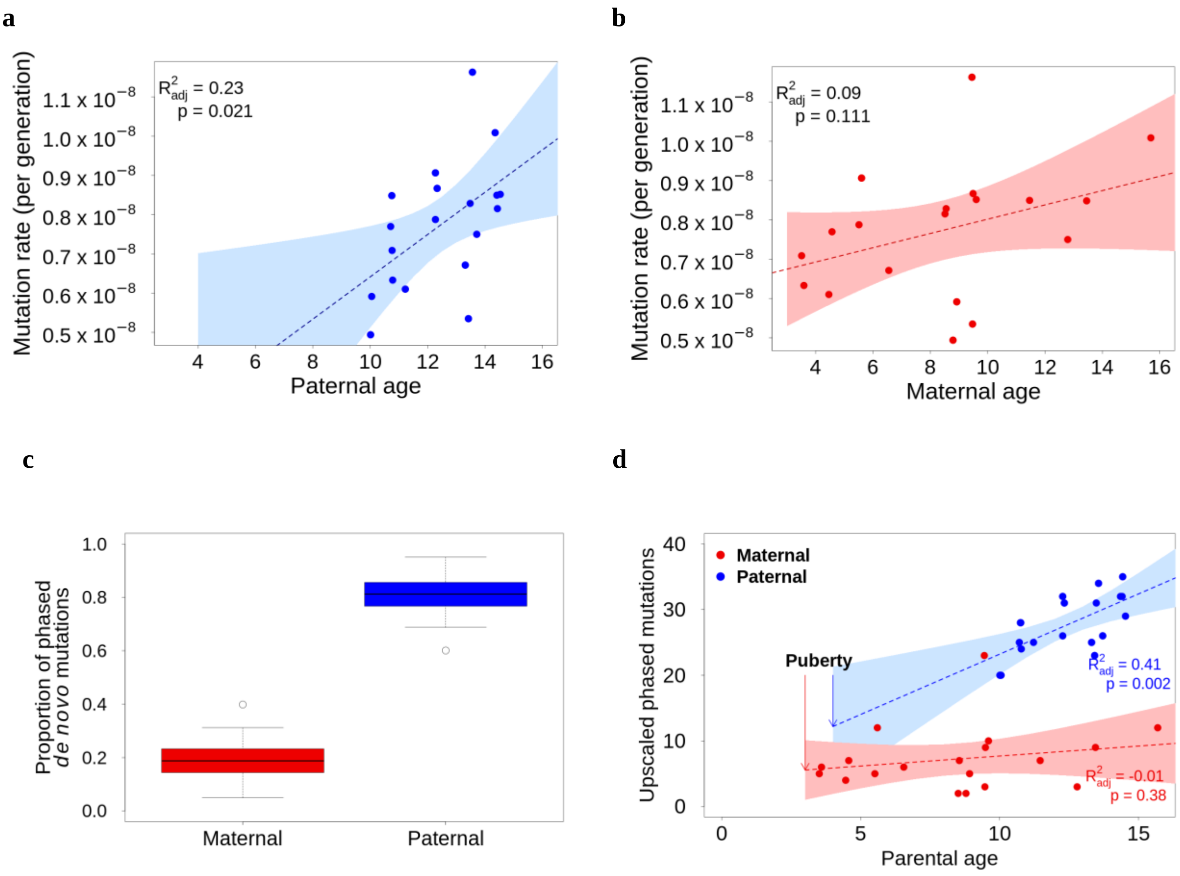

Figure 3

Click here to access/download;Figure;Rhesus_mutation_rate_figures_3.pdf

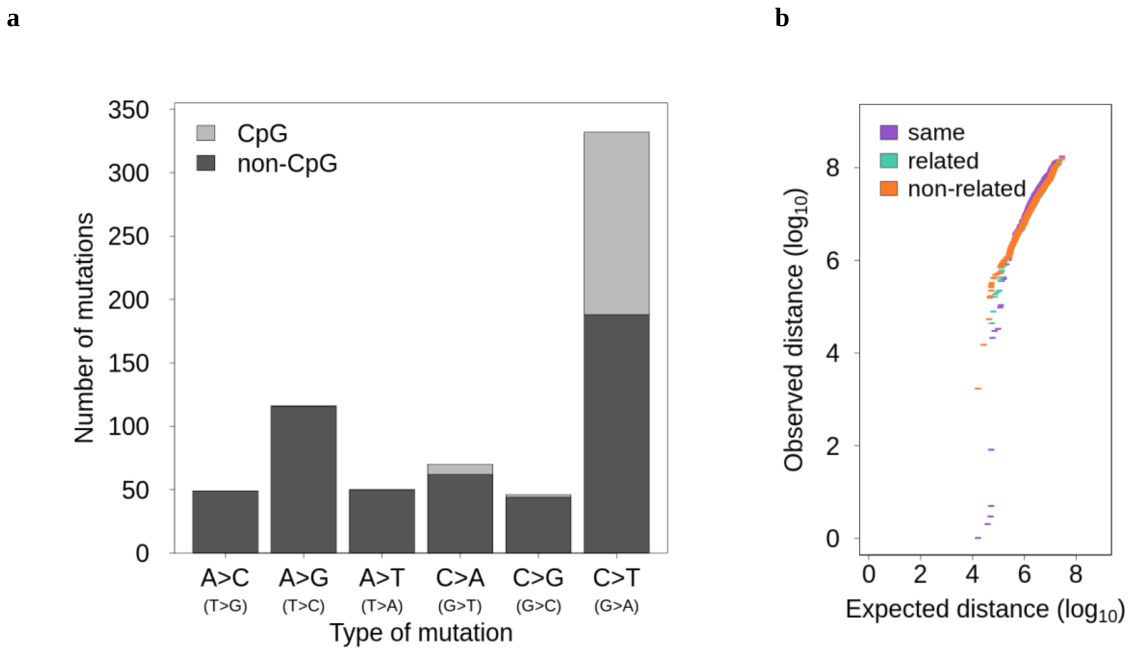

Figure 4

Click here to access/download;Figure;Rhesus_mutation_rate_figures_4.pdf

a

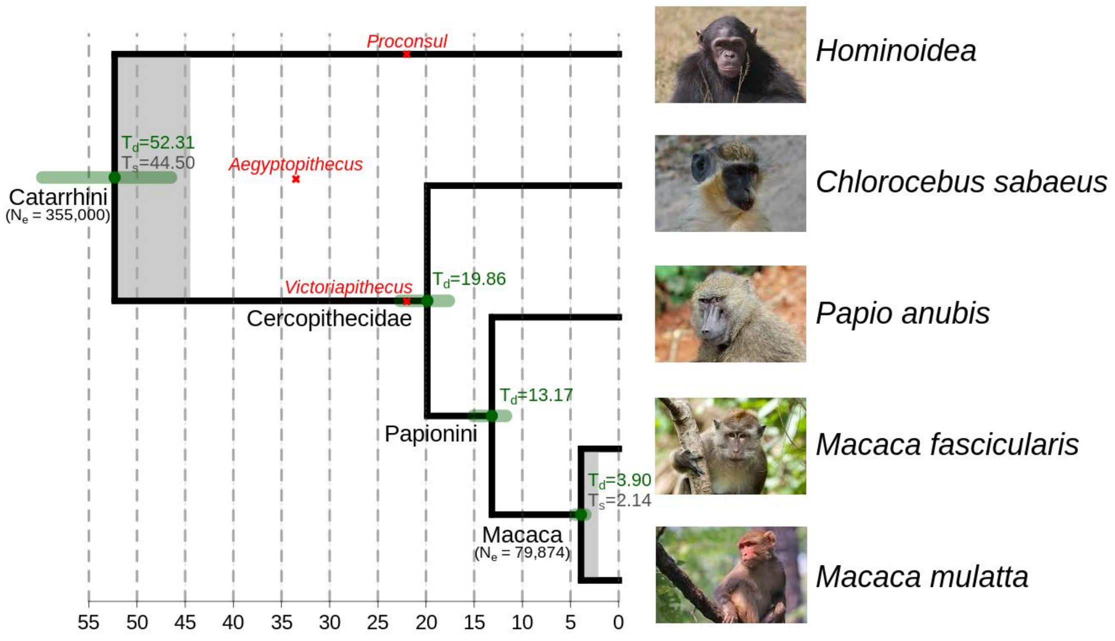

b

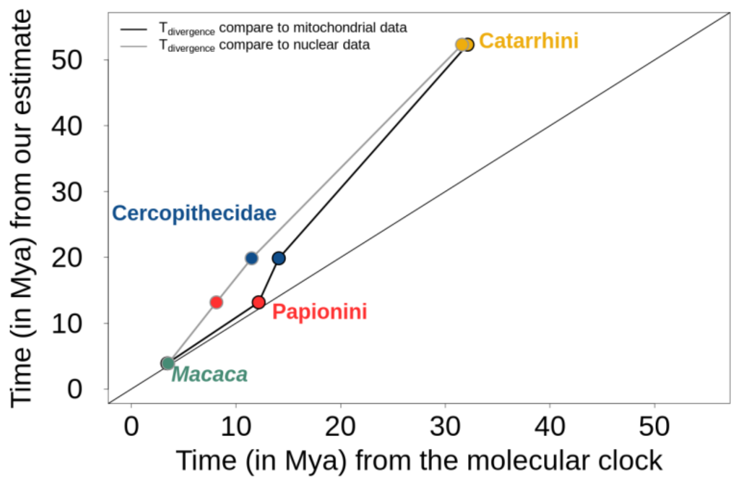

Click here to access/download
Supplementary Material
Rhesus_mutation_rate_sup_material.docx

cover letter Click here to access/download;Personal Cover;Rhesus_mutation_rate_cover_letter.pdf

Dear Editor,

We are pleased to submit our manuscript “The germline mutational process in rhesus macaque and its implications for phylogenetic dating” for consideration as a research article in GigaScience.

Germline mutation rate (GMR) is a key concept in all disciplines of biology, and particularly in evolutionary biology and biomedical sciences. The GMR has been reported from whole genome comparisons of pedigrees in several primate species. However, only humans have been extensively investigated using high coverage sequencing for multiple trios, whereas the GMRs in most other species have only been studied with low coverage sequencing on small numbers of trios. This has limited the possibility to analyze the contributing factors for germline mutation rate variation, both intra-specifically and cross-specifically.

In this work, we produced high depth sequences (76 X after mapping per individual) for a large pedigree of rhesus macaque (19 trios, 33 individuals), in order to explore some of the most striking questions relating to the processes underlying germline mutation.

This dataset allowed us to achieve several major novel findings:

1. The particularly high coverage of our sequences allowed us to improve the pipeline for detecting *de novo* mutations by taking into account all possible causes of false positive and false negative calls.

2. We were able to phase 50 % of the germline mutations to their parents of origins. This allowed us to precisely infer the parental age effect on the mutation rate and further inspect the parental sources of *de novo* mutation. We found a stronger paternal age effect on GMR in macaque than in human but similar non-significant maternal age effect. The male bias to germline mutations in macaque is slightly higher than that in human.

3. The large pedigrees allowed us to detect 3.5 % of the total *de novo* mutations shared between siblings. And we do not find any bias of these shared mutations between parents suggesting they might have occurred during post-zygomatic stage, the stage has been largely underappreciated that can produce *de novo* mutations.

4. Finally, we demonstrated our precise estimation on GMR could provide foundation on the dating of speciation and discussed the limits of the traditional molecular clock method versus dating with GMRs.

We are aware that a parallel effort of germline mutation rate study in the rhesus macaque was published recently (http://www.genome.org/cgi/doi/10.1101/gr.255174.119.). We have used different estimation methods in this study. Yet, we believe our work is complementary to this published study as it represents independent estimations on the mutation rate of rhesus macaques, an important biomedical research model.

A precise estimation for the *de novo* mutation rate in non-human species remains a challenging task. There is no agreed-upon standard and pipeline that can be applied universally. Many of the co-authors in our manuscript have organized an online workshop last June with 30 participants from nine of the major scientific groups in the field to discuss good practices and standards aiming to get comparable results in *de novo* mutation rate studies. The consortium is now preparing a separate review paper to discuss the impacts of the different methods on the mutation rate estimation and propose a standard pipeline for future study. Therefore, a comparison of methods is beyond the scope of our current study.

Given the importance of accurate mutation rate estimation, along with the variety of fundamental question could only be addressed here, we believe our work would be of interest for many fields of biology; Evolutionary Biology, Conservation Biology, Population Biology, Molecular Evolution, Phylogenetics.

This manuscript has not been previously published and is not currently under consideration by any other journal.

I hope you will find our study of interest and look forward to hearing from you.

On behalf of all the authors

Sincerely,

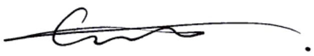

Guojie Zhang, Ph.D.

Professor

Department of Biology

University of Copenhagen

2100 København Ø, Denmark

Phone, office：+45 91 85 54 31

E-mail: guojie.zhang@bio.ku.dk
