# Supplementary material for: The germline mutational process in rhesus macaque and its implications for phylogenetic dating
Source: Gigascience. 2021 May 5;10(5):giab029. doi: 10.1093/gigascience/giab029 (PMC8099771; doi:10.1093/gigascience/giab029)

**Supplementary figures**

**
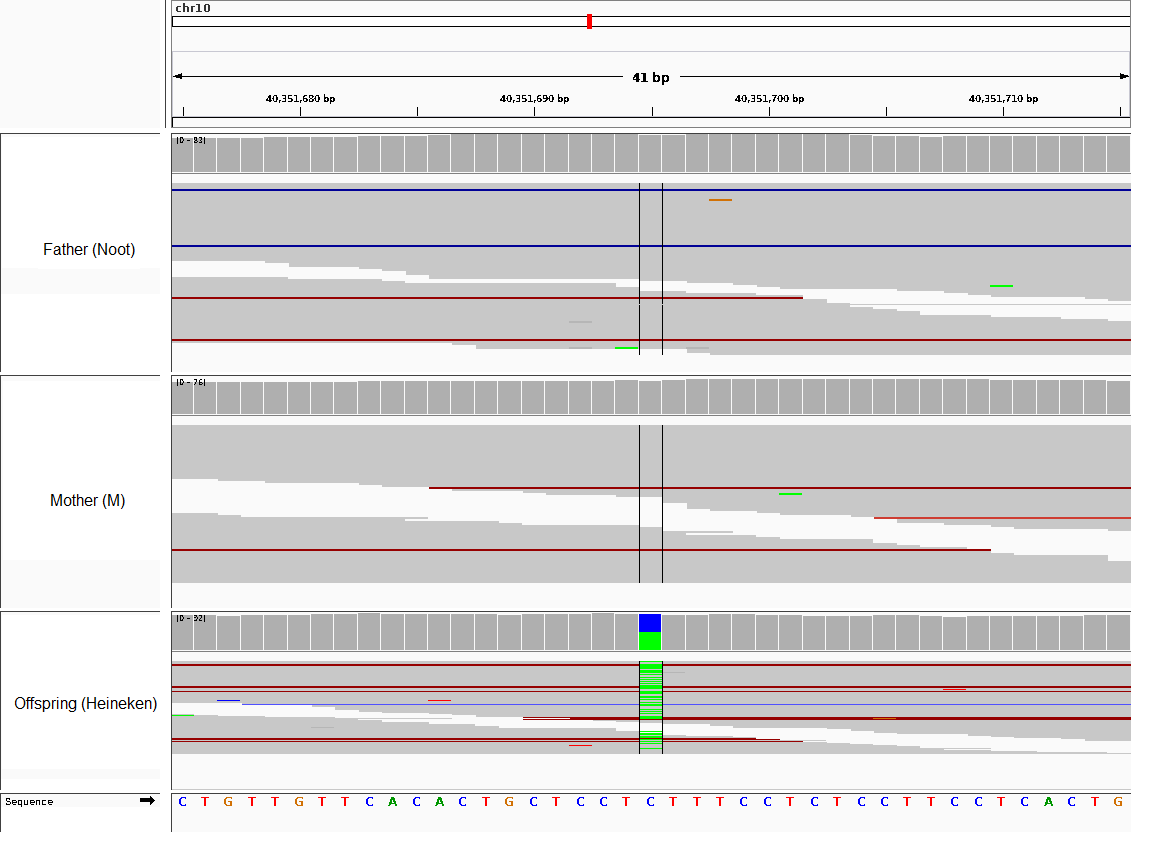
a**

**
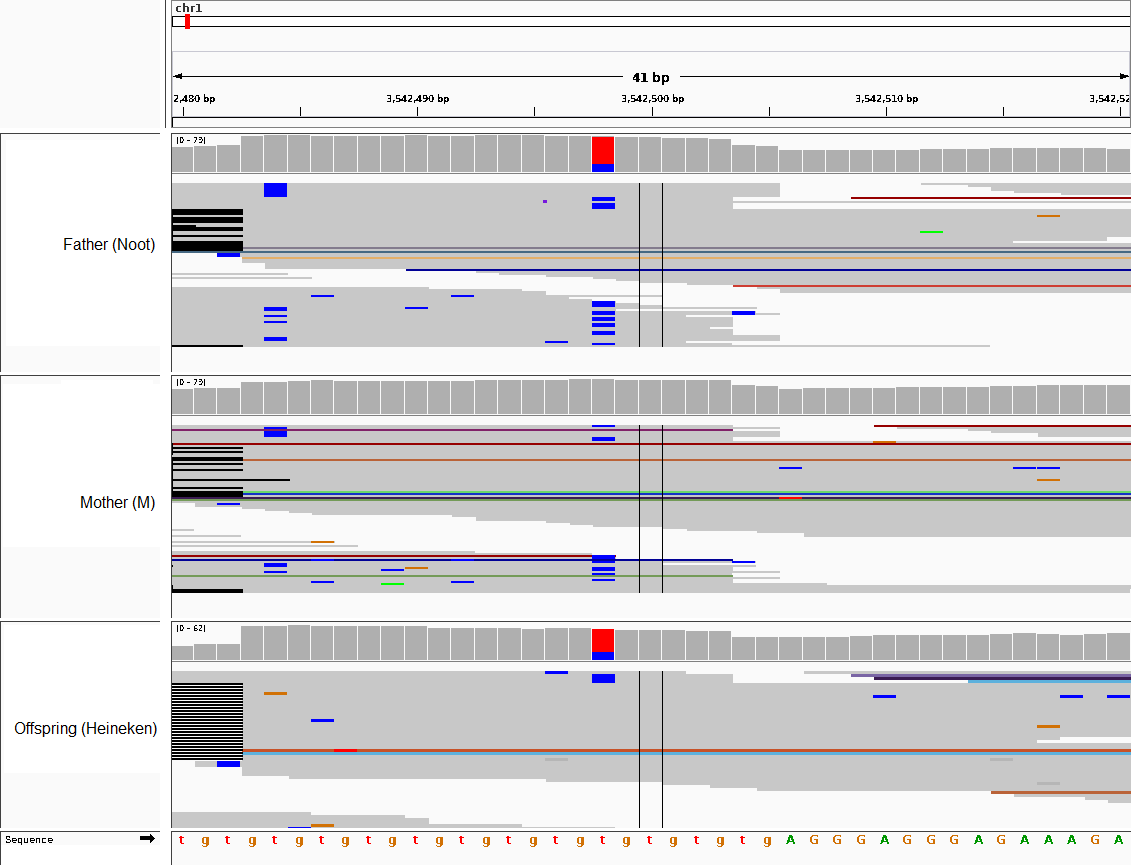
b**

**Figure S1. Manual curation of the *de novo* mutations.** a: an example of *de novo* mutation that passed the manual curation and b: an example of *de novo* mutation that did not pass the manual curation.

**
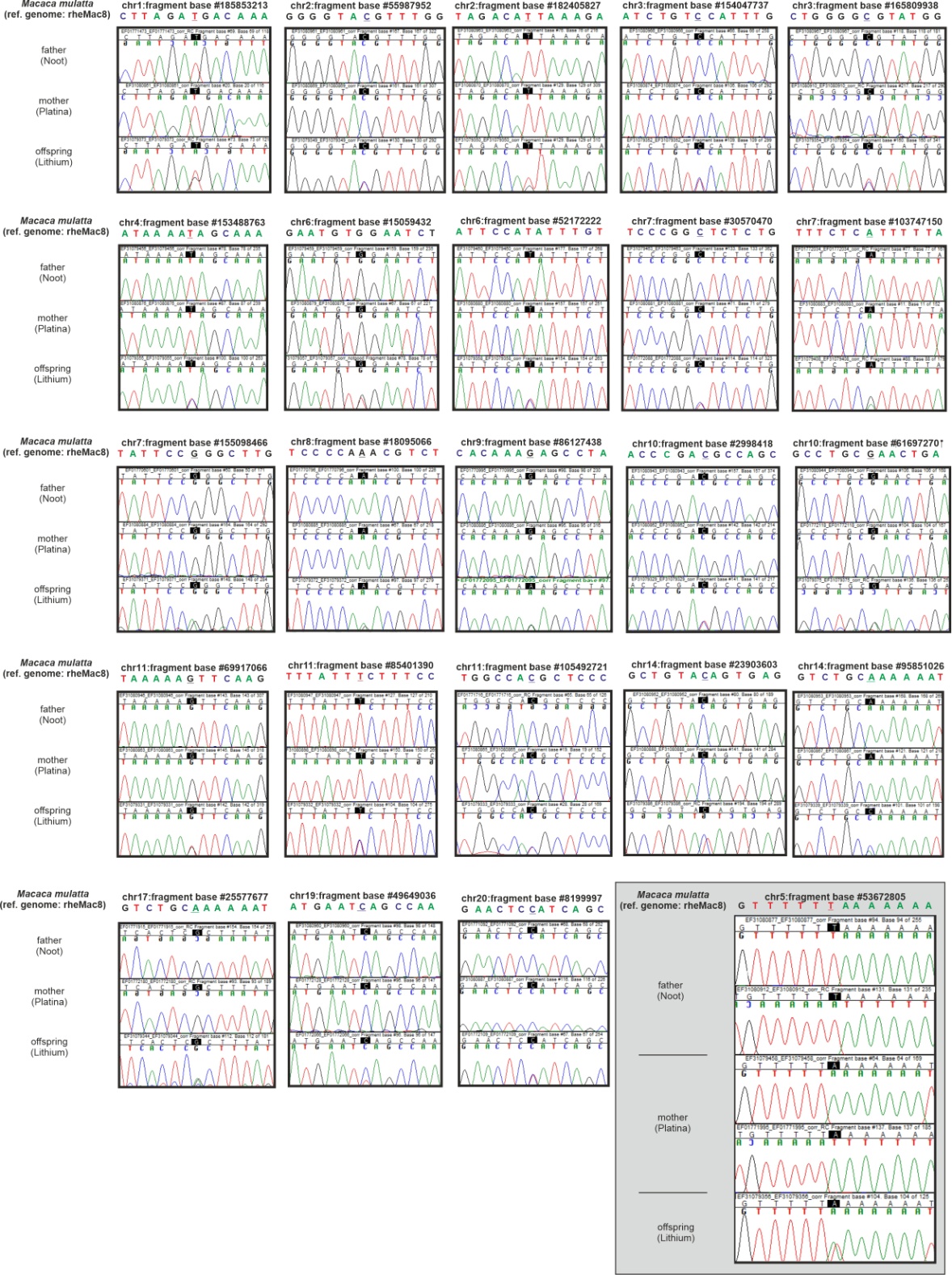
**

**Figure S2. PCR-Sequencing Chromatograms for the 24 *de novo* candidates that were successfully amplified for all three individuals ie. father (Noot), mother (Platina), and offspring (Lithium)**. For each alignment, the candidate *de novo* position on the reference genome of rheMac8 is indicated with an underscore and highlighted in black background at the F,M,O sequences. The order of the colored letters (forward or reverse) in each chromatogram indicates the primer used for sequencing. The *de novo* candidate that was not validated is presented in the bottom grey box. Due to the repetitive bases we provide both forward and reverse sequencing results for the mother and father.


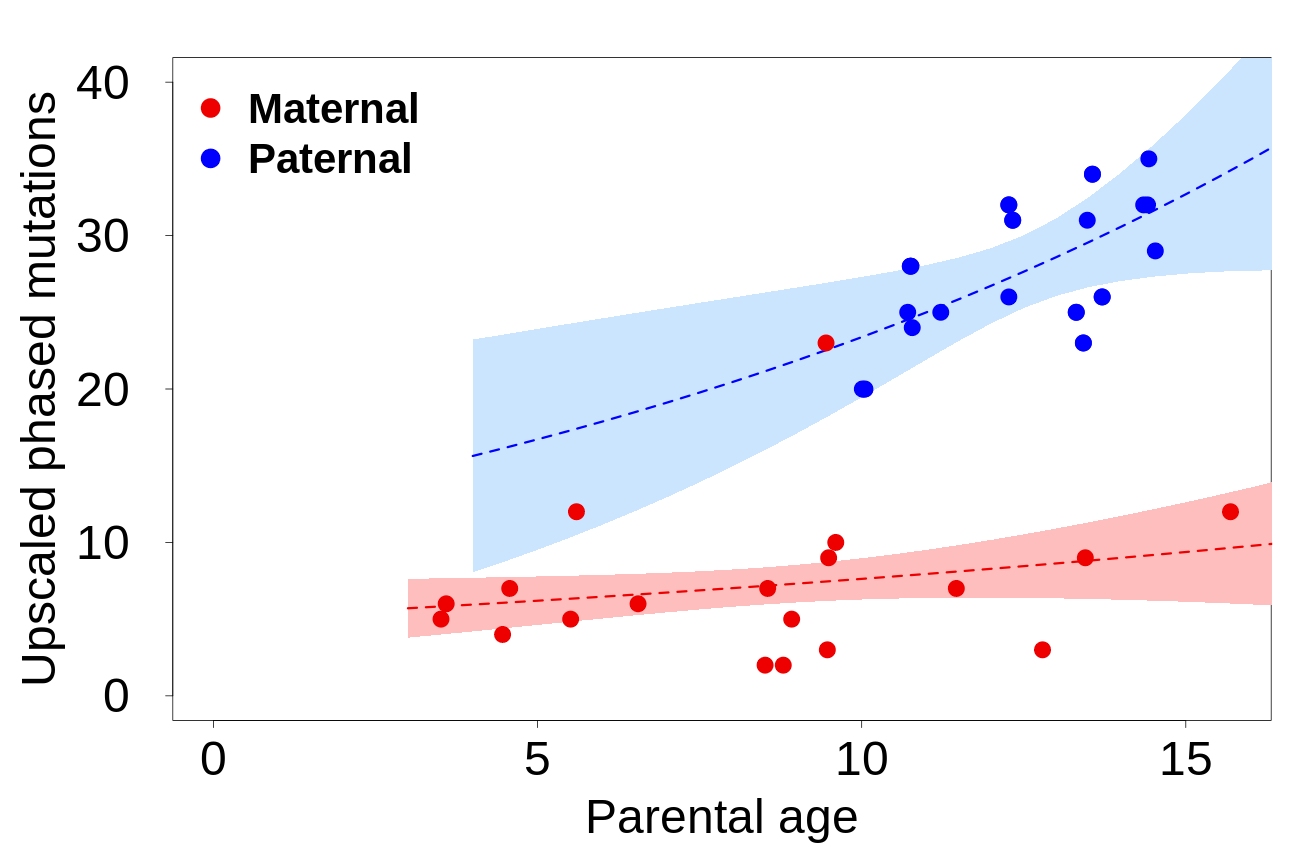


**Figure S3. Poisson regression on the proportion of *de novo* mutation given by each parent apply to the total number of mutation phased (upscaled phased mutation)**. nb_paternal = e ^2.48 + 0.07 x age_father^ and nb_maternal = e ^1.62 + 0.04 x age_mother^.

**
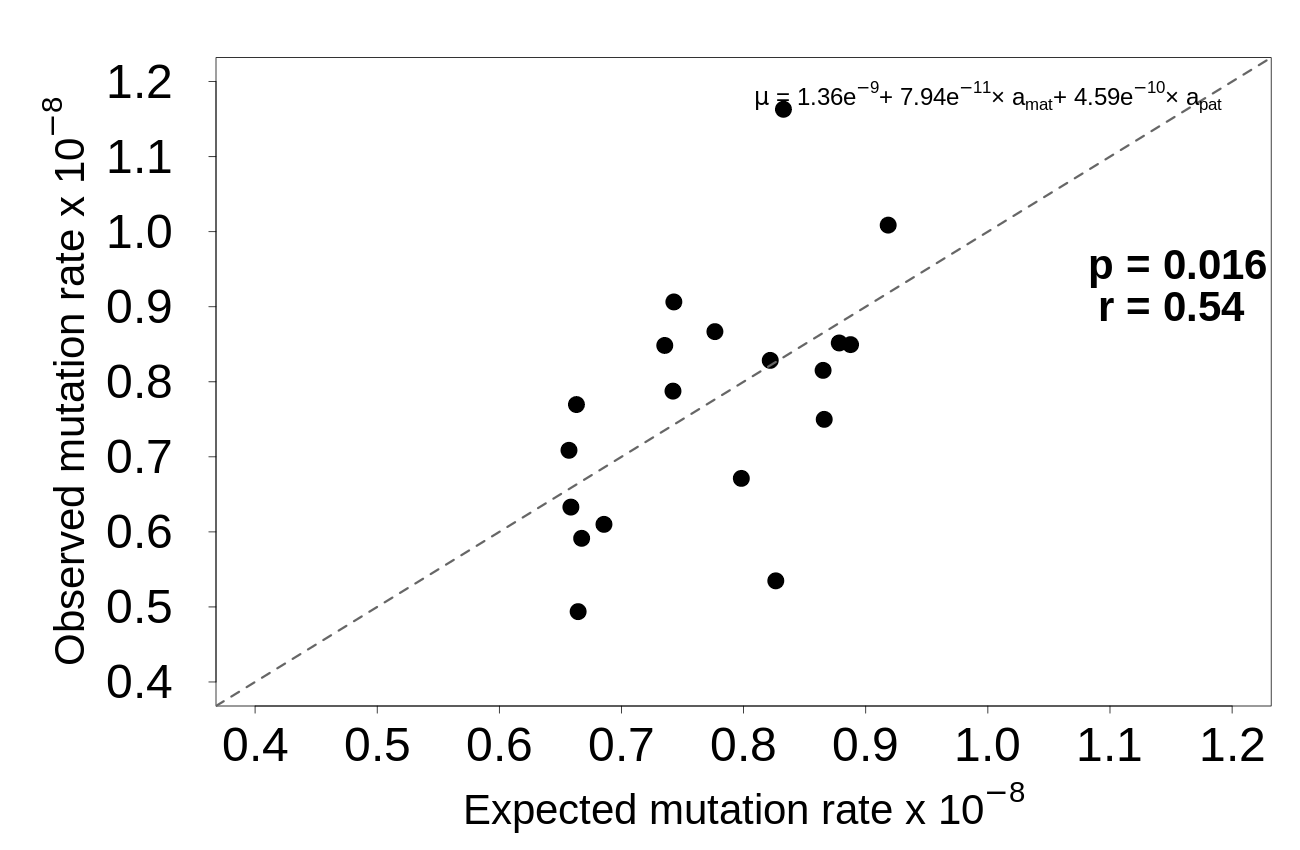
 a**

**b**

**
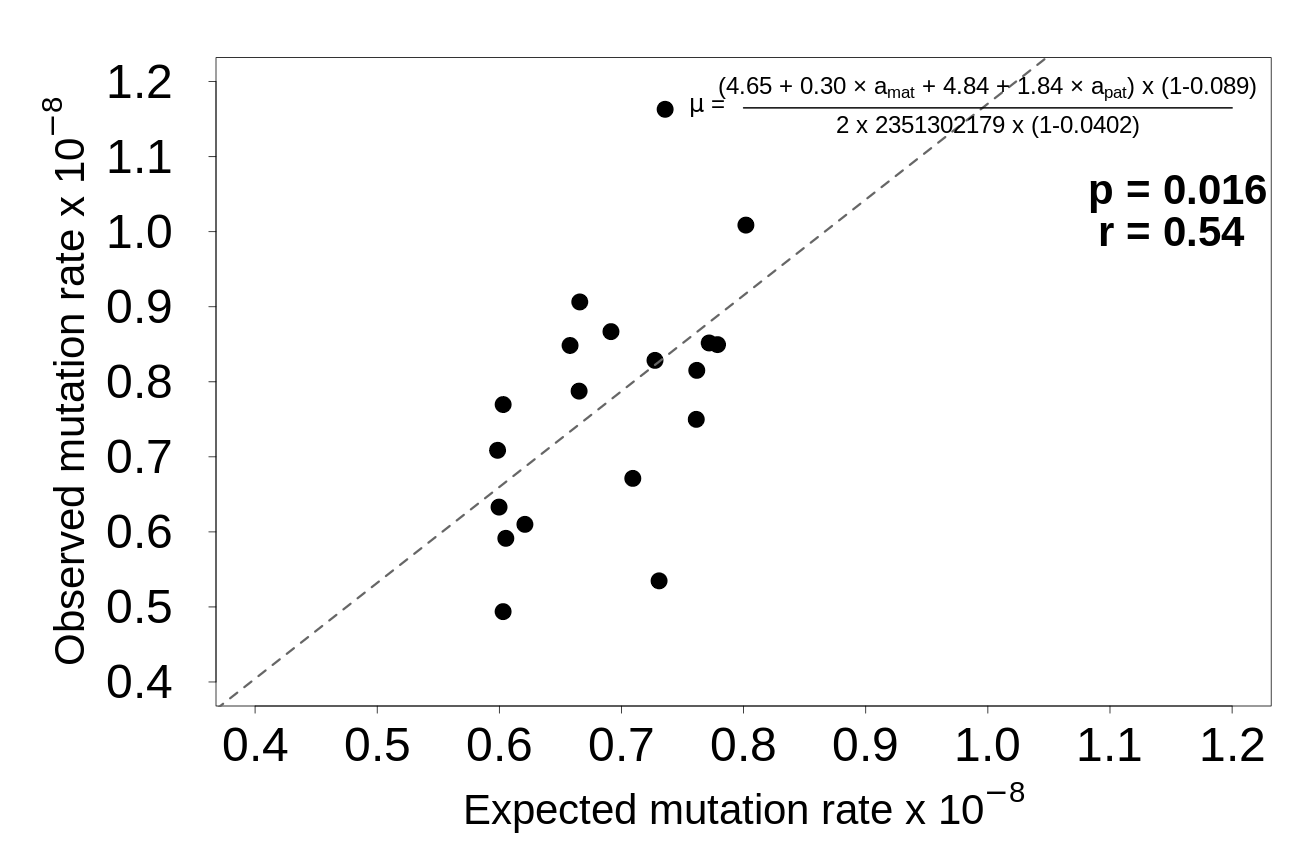
**

**Figure S4.** **Regression comparison**. A: Correlation between the expected mutation rate calculated with the first regression with the age of the parents for each trio and the observed rate (r=0.66, p=0.002). B: Correlation between the expected mutation rate based on the second regression and the observed rate (r=0.65, p=0.002). The expected rates were calculated on the same dataset that served to build the regressions.


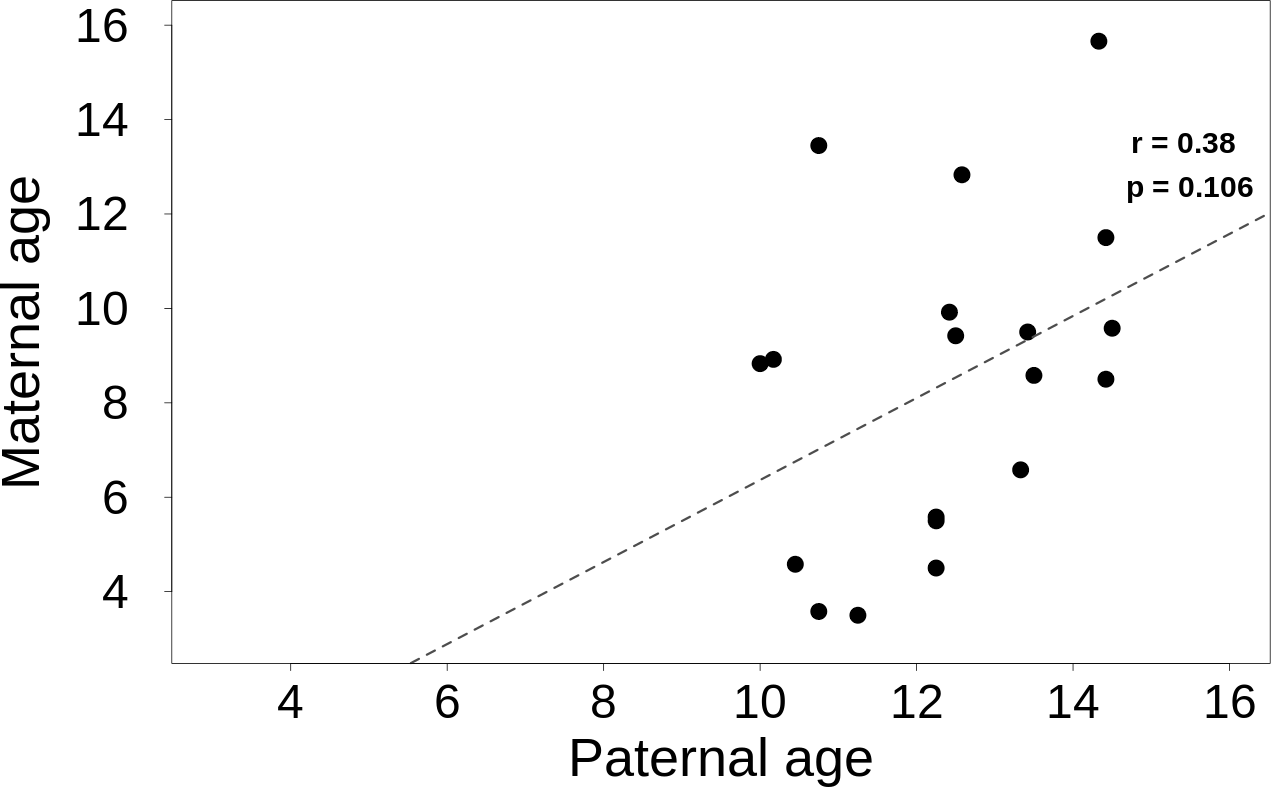


**Figure S5. Correlation between parental ages.**

**
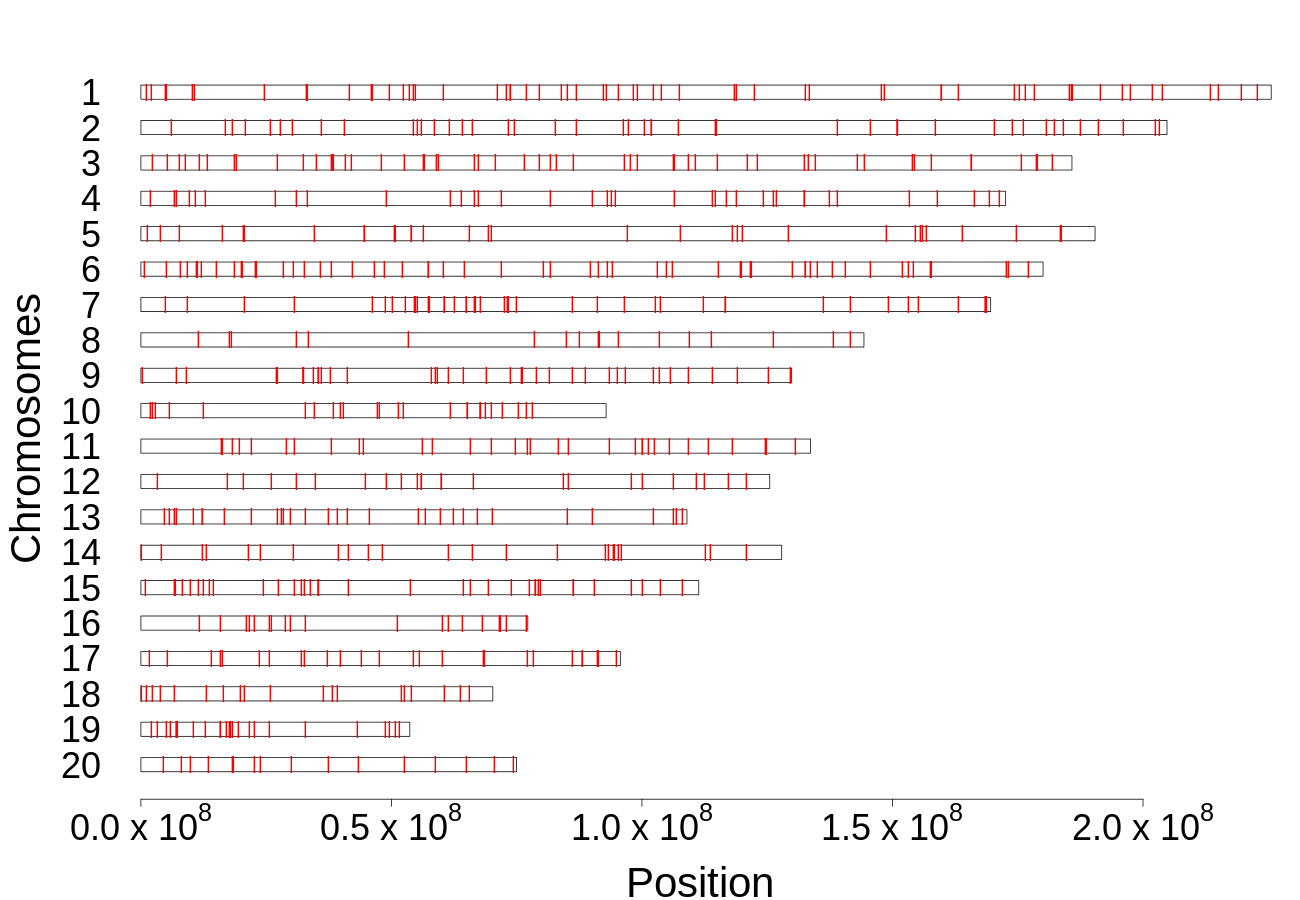
Figure S6. Location of the 685 *de novo* mutations along the genome.**

**
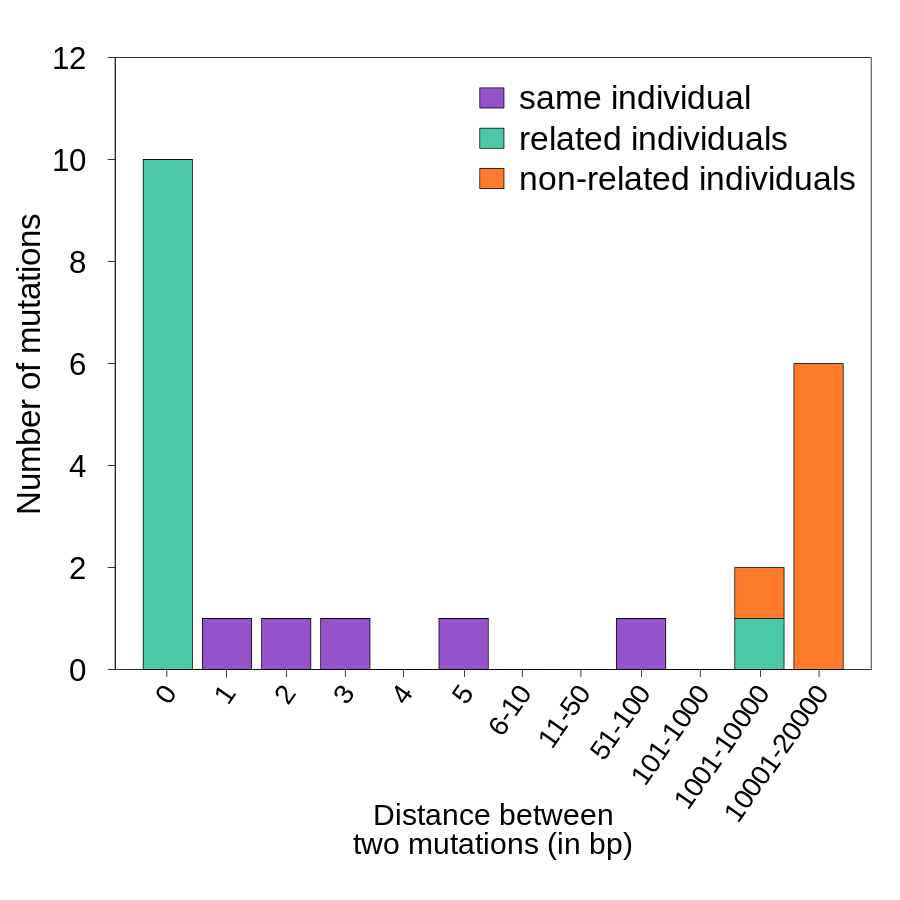

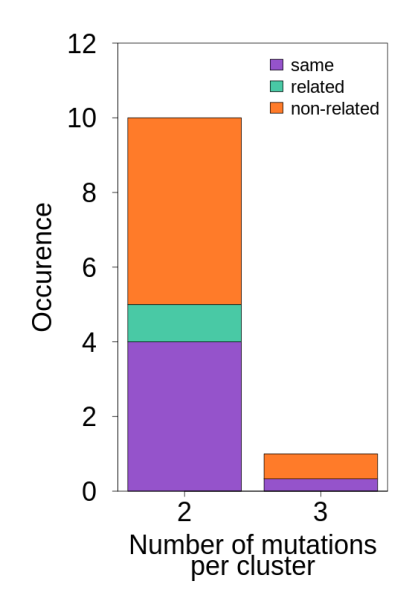
 a b**

**Figure S7. Distance between mutation.** a: Number of mutations per cluster (*<* 20000 bp) within individuals (purple), between related individuals (green), and between non-related individuals (orange). b: Distribution of the distance between mutations in a cluster, clusters involving non-related individuals are mainly observed in larger distances (> 10,000 bp) (Fisher's exact test between non-related and other P = 2.6 × 10^-5^).


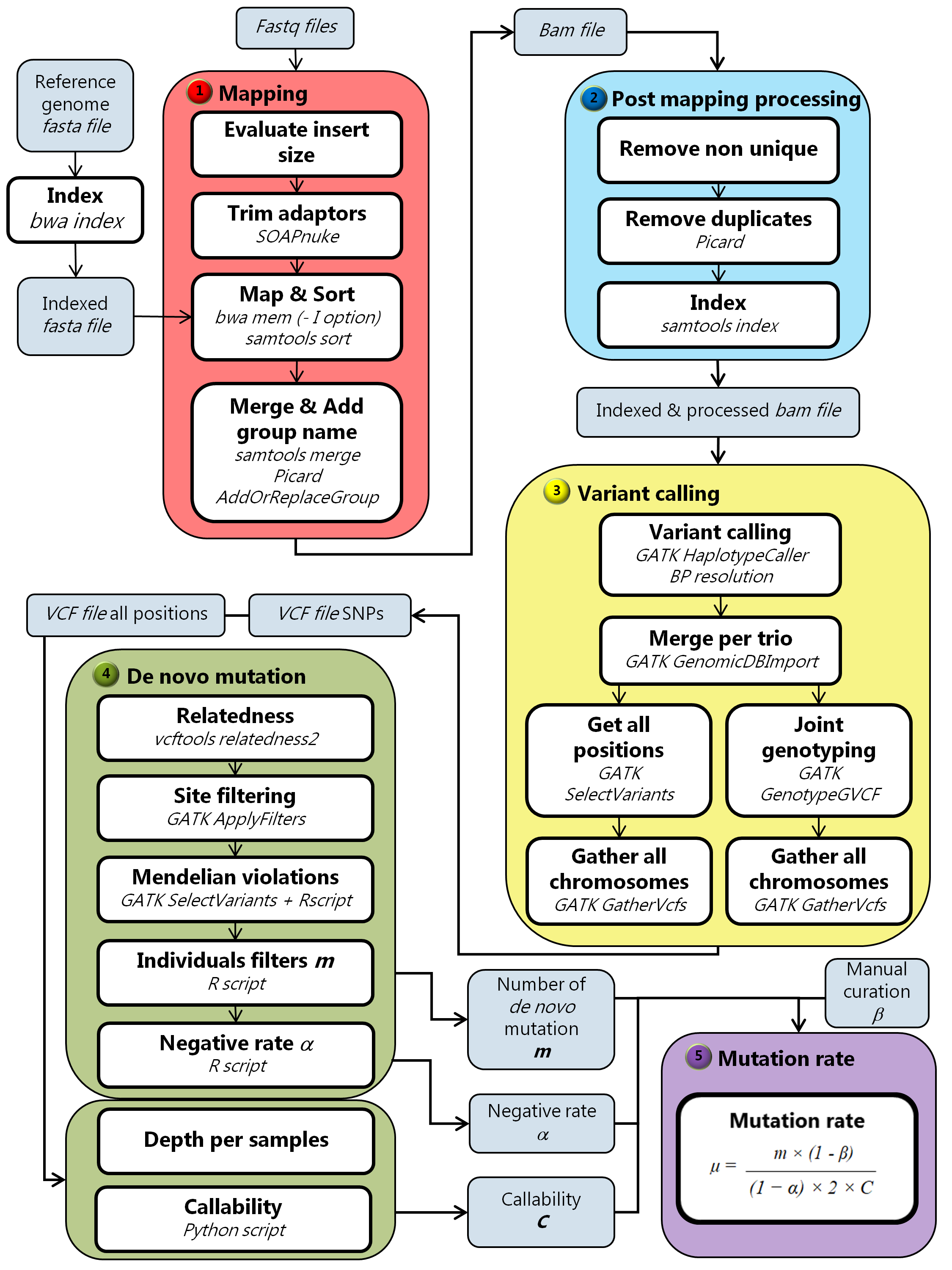


**Figure S8 - Pipeline from fastq file to mutation rate estimation**. The major steps are (1) mapping (2) post mapping processing (3) variant calling (4) *de novo* mutations detection and (5) mutation rate estimation. All the scripts are available on Github: <https://github.com/lucieabergeron/germline_mutation_rate>.

**
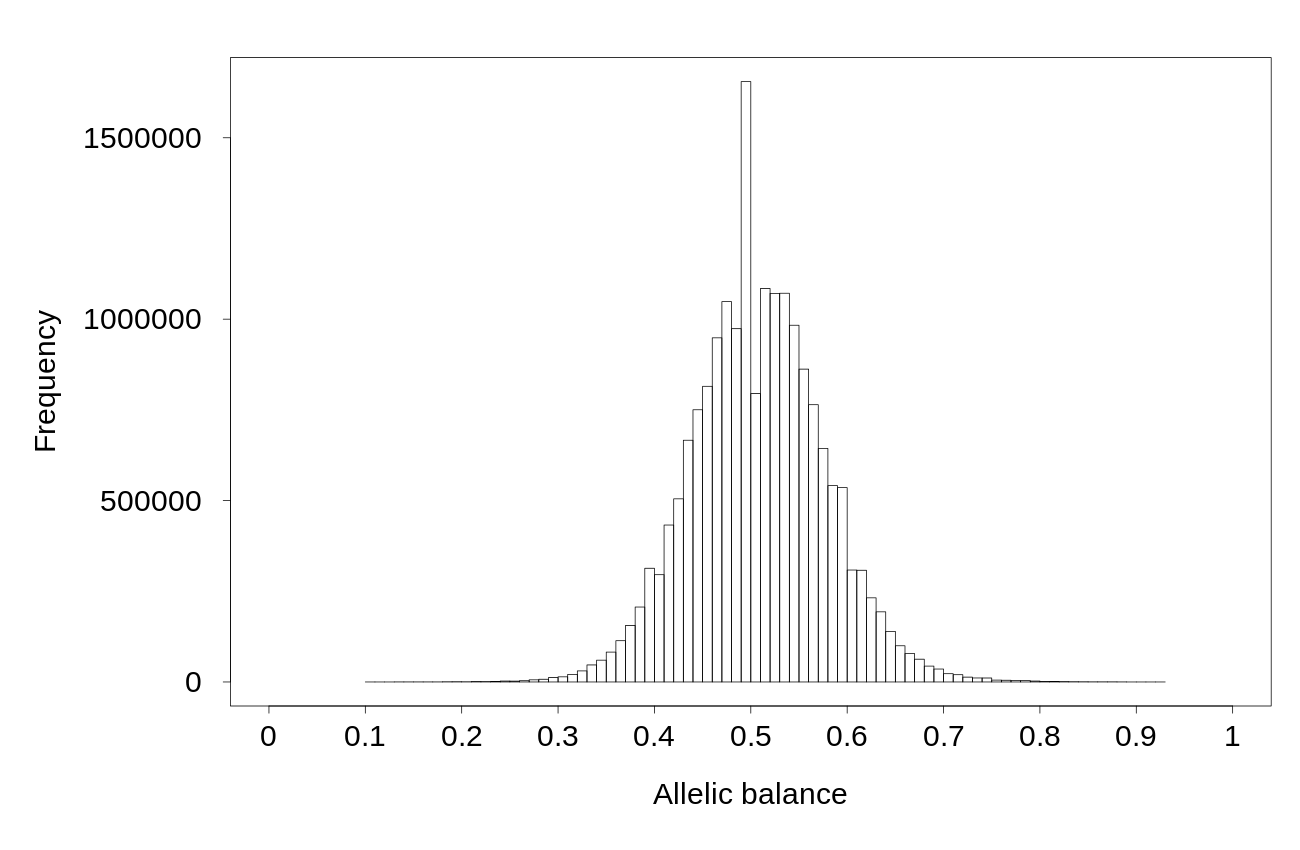
a**

**
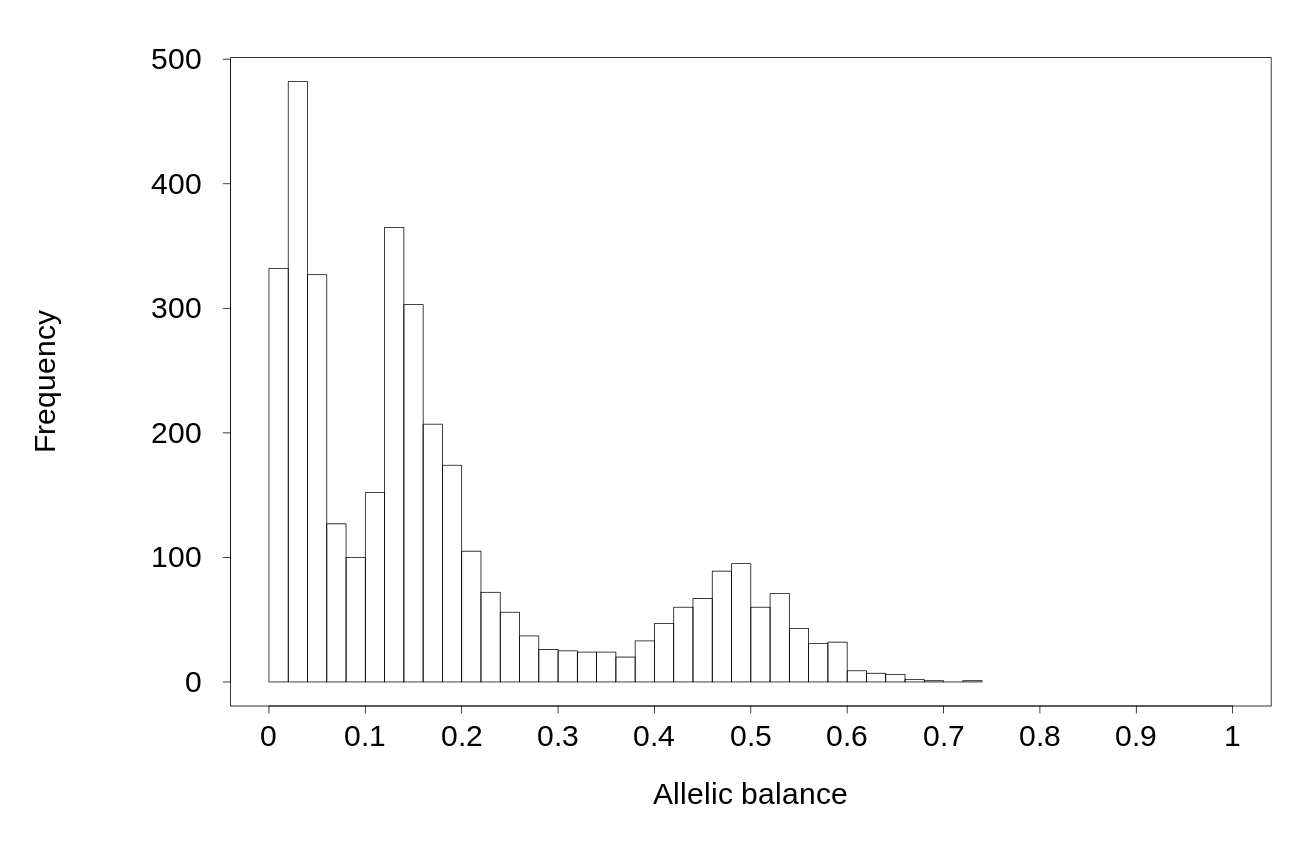
b**


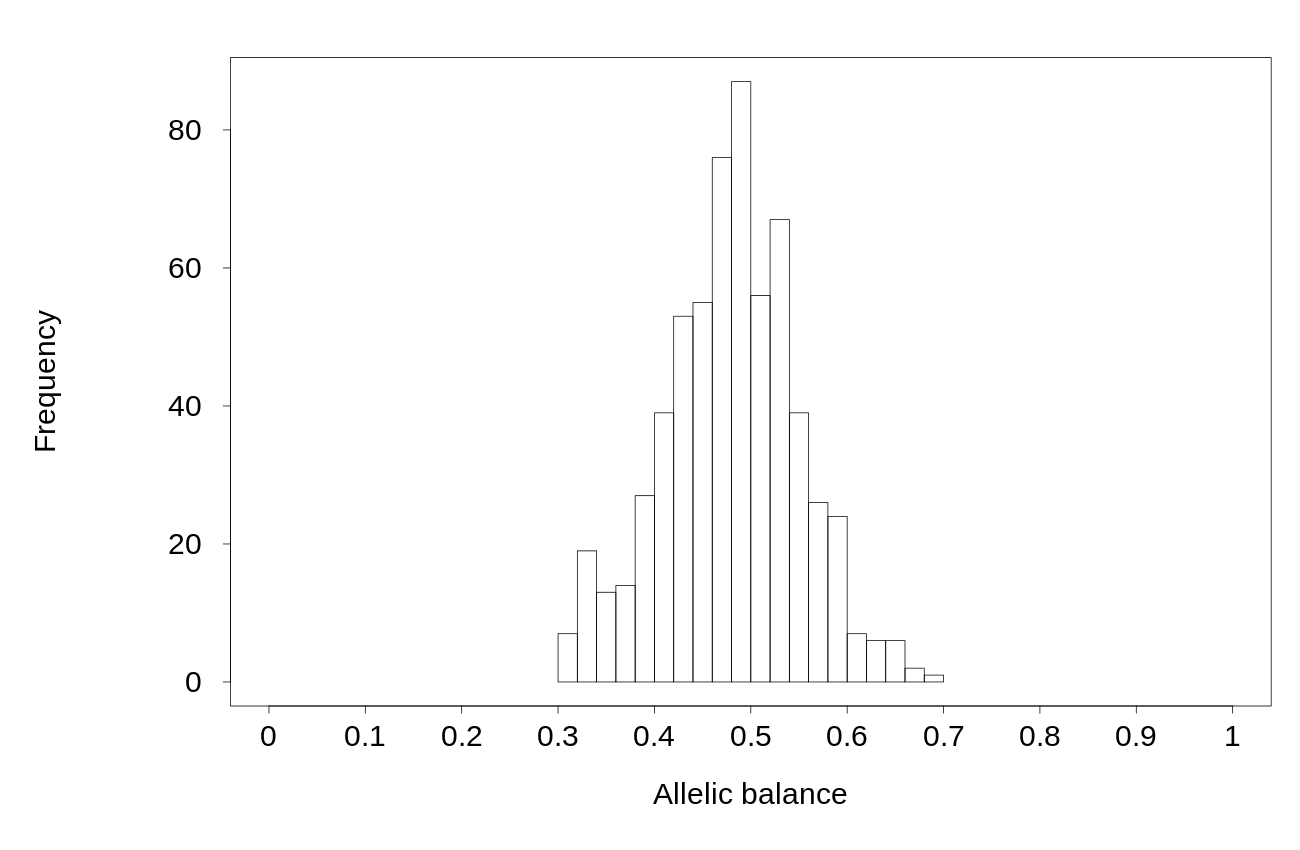
 **c**

**Figure S9 – Allelic balances**. a: Distribution of allelic balance (number of reads supporting the alternative allele/ total number of reads) for all true heterozygotes and b: all candidate *de novo* mutation with all filter except the allelic balance, showing a large portion of somatic mutation or sequencing errors around 0.2. c: The *de novo* mutation after all filter shows a normal distribution around 0.5.

**
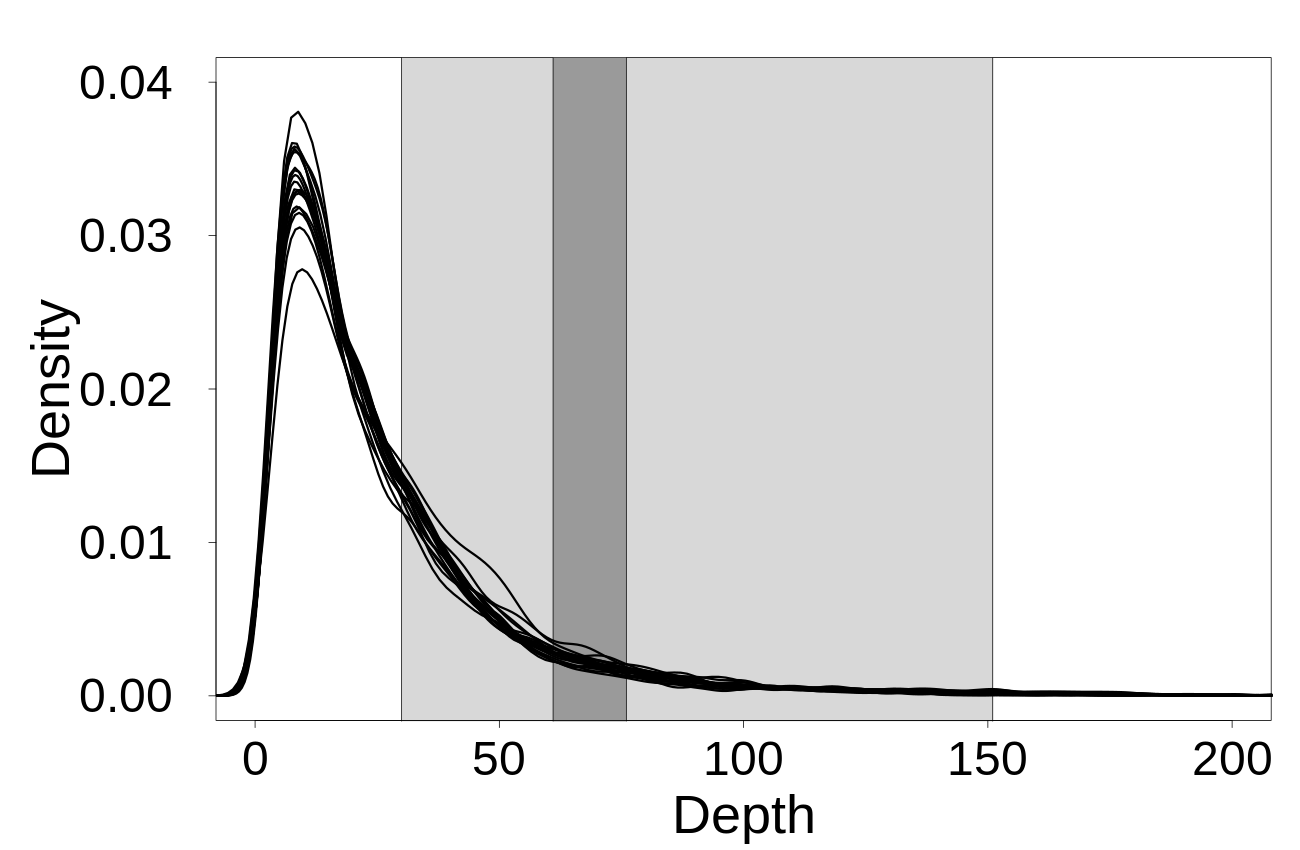
**

**Figure S10 - Average depth distribution of Mendelian violations for each trio**. Dark grey shade corresponds to the range of average depth for the 19 trios and light grey shade corresponds to the minimum 0*.*5*m_depth_* and maximum 2*m_depth_* range of the depth filter.


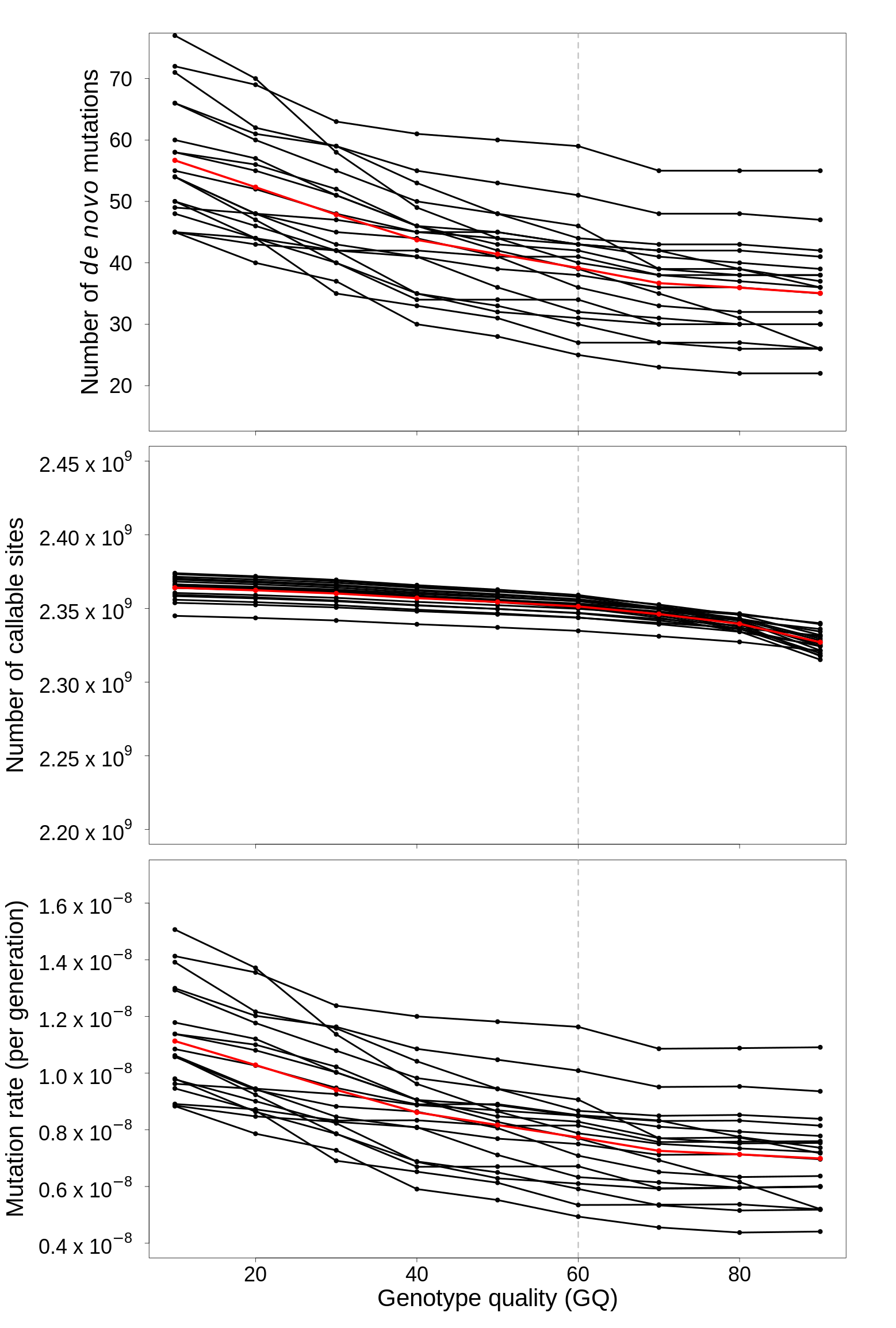


**Figure S11 - Variation of the number of *de novo* mutations, number of callable sites, and mutation rate with different genotype quality threshold. In red the average of the 19 trios.**

**Table S1 – Information for each trio on pedigrees, parental ages, and *de novo* mutations.**

| Offspring | Sex | Father | ♂age | Mother | ♀age | Mutation^a^ | Not phased | Phased | from ♂ | from ♀ | Depth | Callability | Rate µ |
| --- | --- | --- | --- | --- | --- | --- | --- | --- | --- | --- | --- | --- | --- |
| Leffe | F | Poseidon | 10 | M | 8.83 | 25 (-3) | 11 | 11 | 10 | 1 | 63 | 2350483993 | 0.49 × 10−8 |
| Honoria | F | Smack | 13.42 | Nora | 9.5 | 27 (-1) | 10 | 16 | 14 | 2 | 69 | 2343827964 | 0.54 × 10−8 |
| Chalk | M | Poseidon | 10.17 | Platina | 8.92 | 30 (-5) | 14 | 11 | 9 | 2 | 66 | 2354810054 | 0.59 × 10−8 |
| Heineken | F | Noot | 13.25 | M | 4.5 | 31 (-2) | 16 | 13 | 11 | 2 | 63 | 2359040186 | 0.61 × 10−8 |
| Djembe | F | Poseidon | 10.75 | Sabar | 3.58 | 32 (-2) | 15 | 15 | 12 | 3 | 68 | 2346689216 | 0.63 × 10−8 |
| Grolsch | F | Noot | 13.33 | M | 6.58 | 34 (-3) | 16 | 15 | 12 | 3 | 63 | 2351304425 | 0.67 × 10−8 |
| Magenta | M | Poseidon | 11.25 | Amber | 3.5 | 36 (-3) | 13 | 20 | 17 | 3 | 67 | 2358110243 | 0.71 × 10−8 |
| Bridgit | F | Smack | 12.58 | Jones | 12.83 | 38 (-9) | 18 | 11 | 10 | 1 | 71 | 2352269483 | 0.75 × 10−8 |
| Hoegaarde | F | Poseidon | 10.75 | Heineken | 4.58 | 39 (-7) | 9 | 23 | 18 | 5 | 65 | 2352219040 | 0.77 × 10−8 |
| Bavaria | M | Noot | 12.25 | M | 5.5 | 40 (-3) | 17 | 20 | 17 | 3 | 61 | 2357779708 | 0.79 × 10−8 |
| Elanor | F | Smack | 14.42 | Suzie | 8.5 | 41 (-4) | 19 | 18 | 17 | 1 | 73 | 2334764487 | 0.81 × 10−8 |
| Khan | M | Smack | 13.5 | Raksha | 8.58 | 42 (-4) | 17 | 21 | 17 | 4 | 75 | 2353293530 | 0.83 × 10−8 |
| Sir | M | Smack | 14.42 | Lammie | 11.5 | 43 (-4) | 22 | 17 | 14 | 3 | 73 | 2349711303 | 0.85 × 10−8 |
| Babet | F | Poseidon | 10.75 | Mayke | 9.92 | 43 (-6) | 19 | 18 | 14 | 4 | 66 | 2352809332 | 0.85 × 10−8 |
| Mowgli | M | Smack | 14.5 | Raksha | 9.58 | 43 (-4) | 17 | 22 | 16 | 6 | 75 | 2343655111 | 0.85 × 10−8 |
| Amber | F | Noot | 12.42 | Mayke | 9.92 | 44 (-4) | 23 | 17 | 13 | 4 | 64 | 2356253606 | 0.87 × 10−8 |
| Lithium | M | Noot | 12.25 | Platina | 5.58 | 46 (-8) | 19 | 19 | 13 | 6 | 65 | 2355909461 | 0.91 × 10−8 |
| Delta | F | Smack | 14.33 | Zeta | 15.66 | 51 (-7) | 20 | 24 | 17 | 7 | 71 | 2346965047 | 1.01 × 10−8 |
| Teus | F | Smack | 12.5 | Coma | 9.42 | 59 (-2) | 31 | 26 | 16 | 10 | 72 | 2354845218 | 1.16 × 10−8 |

a: observed number of mutations and in brackets the numbers of possible false positives based on manual curation

**Table S2 – Position of the 663 *de novo* mutations used for all analyses.**

| Chromosome | Position | Sample |
| --- | --- | --- |
| chr1 | 1049987 | Babet |
| chr1 | 2149379 | Magenta |
| chr1 | 4833782 | Teus |
| chr1 | 5073694 | Babet |
| chr1 | 10365560 | Djembe |
| chr1 | 10790990 | Lithium |
| chr1 | 24661568 | Bridgit |
| chr1 | 33016325 | Hoegaarde |
| chr1 | 33314720 | Elanor |
| chr1 | 41564951 | Babet |
| chr1 | 46052070 | Khan |
| chr1 | 46155208 | Teus |
| chr1 | 49553664 | Elanor |
| chr1 | 52360106 | Amber |
| chr1 | 53511189 | Hoegaarde |
| chr1 | 54451843 | Bridgit |
| chr1 | 54879132 | Bridgit |
| chr1 | 60361068 | Hoegaarde |
| chr1 | 71165625 | Honoria |
| chr1 | 72885638 | Magenta |
| chr1 | 73665416 | Bridgit |
| chr1 | 73714534 | Honoria |
| chr1 | 76954377 | Delta |
| chr1 | 79476852 | Mowgli |
| chr1 | 83968737 | Grolsch |
| chr1 | 85109446 | Elanor |
| chr1 | 86950122 | Elanor |
| chr1 | 92380997 | Bavaria |
| chr1 | 92931616 | Mowgli |
| chr1 | 95248287 | Khan |
| chr1 | 98286758 | Khan |
| chr1 | 99027621 | Delta |
| chr1 | 102211352 | Mowgli |
| chr1 | 103948799 | Mowgli |
| chr1 | 107563074 | Babet |
| chr1 | 118537483 | Khan |
| chr1 | 118880142 | Djembe |
| chr1 | 122469553 | Bavaria |
| chr1 | 132655513 | Mowgli |
| chr1 | 133476123 | Hoegaarde |
| chr1 | 147834454 | Magenta |
| chr1 | 148365549 | Mowgli |
| chr1 | 159689377 | Babet |
| chr1 | 159723765 | Khan |
| chr1 | 163146386 | Bridgit |
| chr1 | 174263383 | Elanor |
| chr1 | 175386650 | Elanor |
| chr1 | 176612901 | Magenta |
| chr1 | 178309484 | Honoria |
| chr1 | 185322173 | Hoegaarde |
| chr1 | 185681396 | Bavaria |
| chr1 | 185853213 | Lithium |
| chr1 | 191466982 | Teus |
| chr1 | 196005916 | Delta |
| chr1 | 197466940 | Magenta |
| chr1 | 201837541 | Delta |
| chr1 | 203845059 | Sir |
| chr1 | 213449733 | Hoegaarde |
| chr1 | 214980686 | Khan |
| chr1 | 219658319 | Mowgli |
| chr1 | 222812036 | Babet |
| chr10 | 1957159 | Bavaria |
| chr10 | 2385952 | Delta |
| chr10 | 2998418 | Lithium |
| chr10 | 5642136 | Djembe |
| chr10 | 12485775 | Grolsch |
| chr10 | 32935037 | Bavaria |
| chr10 | 34637176 | Amber |
| chr10 | 38384243 | Teus |
| chr10 | 39928071 | Teus |
| chr10 | 40351695 | Heineken |
| chr10 | 47275879 | Magenta |
| chr10 | 47674587 | Djembe |
| chr10 | 51477764 | Djembe |
| chr10 | 52504006 | Bridgit |
| chr10 | 61697270 | Lithium |
| chr10 | 65163492 | Khan |
| chr10 | 65163492 | Delta |
| chr10 | 67704591 | Chalk |
| chr10 | 67704672 | Chalk |
| chr10 | 67720113 | Sir |
| chr10 | 68702798 | Delta |
| chr10 | 70064955 | Bavaria |
| chr10 | 72214764 | Grolsch |
| chr10 | 75270640 | Honoria |
| chr10 | 76865893 | Magenta |
| chr10 | 78098043 | Magenta |
| chr11 | 16175067 | Bridgit |
| chr11 | 16375890 | Elanor |
| chr11 | 18319218 | Chalk |
| chr11 | 19649917 | Grolsch |
| chr11 | 22072488 | Teus |
| chr11 | 29039858 | Chalk |
| chr11 | 30751932 | Djembe |
| chr11 | 38096086 | Sir |
| chr11 | 43699480 | Teus |
| chr11 | 44403758 | Heineken |
| chr11 | 56157142 | Hoegaarde |
| chr11 | 58210826 | Chalk |
| chr11 | 65725777 | Sir |
| chr11 | 69917066 | Lithium |
| chr11 | 74850710 | Djembe |
| chr11 | 77140145 | Teus |
| chr11 | 77806296 | Djembe |
| chr11 | 83412750 | Bavaria |
| chr11 | 85401390 | Lithium |
| chr11 | 93543084 | Grolsch |
| chr11 | 98692026 | Heineken |
| chr11 | 100067183 | Amber |
| chr11 | 100100309 | Amber |
| chr11 | 101271115 | Leffe |
| chr11 | 102550219 | Khan |
| chr11 | 105492721 | Lithium |
| chr11 | 109301631 | Heineken |
| chr11 | 113354526 | Khan |
| chr11 | 118079276 | Delta |
| chr11 | 124696600 | Elanor |
| chr11 | 124923654 | Delta |
| chr11 | 130557830 | Elanor |
| chr12 | 3339257 | Grolsch |
| chr12 | 17192055 | Lithium |
| chr12 | 20490365 | Delta |
| chr12 | 26152394 | Honoria |
| chr12 | 30970268 | Sir |
| chr12 | 34916837 | Khan |
| chr12 | 44805275 | Elanor |
| chr12 | 49113289 | Teus |
| chr12 | 51978831 | Bridgit |
| chr12 | 55149439 | Bavaria |
| chr12 | 55952972 | Teus |
| chr12 | 55952973 | Teus |
| chr12 | 59966197 | Hoegaarde |
| chr12 | 59979880 | Bridgit |
| chr12 | 66302302 | Amber |
| chr12 | 84363716 | Bridgit |
| chr12 | 85346196 | Amber |
| chr12 | 97910997 | Elanor |
| chr12 | 100084478 | Delta |
| chr12 | 106233127 | Bridgit |
| chr12 | 110983332 | Heineken |
| chr12 | 112567785 | Bavaria |
| chr12 | 117335453 | Elanor |
| chr12 | 120840236 | Sir |
| chr13 | 4691517 | Lithium |
| chr13 | 5805698 | Delta |
| chr13 | 6694818 | Sir |
| chr13 | 7187695 | Bridgit |
| chr13 | 10558810 | Amber |
| chr13 | 12194474 | Amber |
| chr13 | 12222097 | Delta |
| chr13 | 16689137 | Amber |
| chr13 | 22006971 | Babet |
| chr13 | 27314551 | Delta |
| chr13 | 28058835 | Magenta |
| chr13 | 28463207 | Heineken |
| chr13 | 29847487 | Djembe |
| chr13 | 32839004 | Lithium |
| chr13 | 37350605 | Elanor |
| chr13 | 39195088 | Magenta |
| chr13 | 41219997 | Chalk |
| chr13 | 45680719 | Teus |
| chr13 | 55458117 | Khan |
| chr13 | 56898396 | Bavaria |
| chr13 | 59832159 | Hoegaarde |
| chr13 | 62463853 | Leffe |
| chr13 | 64398313 | Djembe |
| chr13 | 67091385 | Khan |
| chr13 | 70201543 | Delta |
| chr13 | 85065504 | Babet |
| chr13 | 90054533 | Teus |
| chr13 | 102276801 | Chalk |
| chr13 | 106205783 | Leffe |
| chr13 | 106990069 | Teus |
| chr13 | 108060157 | Khan |
| chr14 | 173341 | Djembe |
| chr14 | 4167186 | Amber |
| chr14 | 12308607 | Babet |
| chr14 | 13164076 | Amber |
| chr14 | 21405576 | Grolsch |
| chr14 | 23903603 | Lithium |
| chr14 | 30493574 | Chalk |
| chr14 | 39504375 | Sir |
| chr14 | 41517101 | Heineken |
| chr14 | 45523096 | Djembe |
| chr14 | 48120086 | Grolsch |
| chr14 | 61334588 | Sir |
| chr14 | 66255584 | Mowgli |
| chr14 | 72981363 | Sir |
| chr14 | 83054360 | Djembe |
| chr14 | 92626902 | Djembe |
| chr14 | 93386743 | Mowgli |
| chr14 | 94404620 | Mowgli |
| chr14 | 94561203 | Babet |
| chr14 | 95258975 | Amber |
| chr14 | 95851026 | Lithium |
| chr14 | 112727023 | Grolsch |
| chr14 | 113669433 | Sir |
| chr14 | 120924099 | Honoria |
| chr15 | 1008166 | Grolsch |
| chr15 | 6803517 | Chalk |
| chr15 | 6833056 | Chalk |
| chr15 | 8377451 | Lithium |
| chr15 | 9826367 | Elanor |
| chr15 | 11480723 | Chalk |
| chr15 | 12448304 | Delta |
| chr15 | 13695493 | Djembe |
| chr15 | 14411657 | Lithium |
| chr15 | 24446545 | Bridgit |
| chr15 | 27373968 | Heineken |
| chr15 | 30630267 | Delta |
| chr15 | 32033161 | Bridgit |
| chr15 | 32634468 | Sir |
| chr15 | 33842106 | Bridgit |
| chr15 | 35463257 | Leffe |
| chr15 | 35463257 | Babet |
| chr15 | 35463257 | Magenta |
| chr15 | 41347648 | Magenta |
| chr15 | 53778344 | Mowgli |
| chr15 | 64372852 | Delta |
| chr15 | 65846192 | Delta |
| chr15 | 69385675 | Amber |
| chr15 | 73864540 | Delta |
| chr15 | 77560766 | Sir |
| chr15 | 78825263 | Hoegaarde |
| chr15 | 78846689 | Hoegaarde |
| chr15 | 79321421 | Bridgit |
| chr15 | 79657103 | Amber |
| chr15 | 86302230 | Elanor |
| chr15 | 86357096 | Teus |
| chr15 | 90460849 | Sir |
| chr15 | 97896828 | Magenta |
| chr15 | 100074218 | Honoria |
| chr15 | 103651265 | Teus |
| chr15 | 108156960 | Sir |
| chr16 | 11662689 | Teus |
| chr16 | 15799407 | Teus |
| chr16 | 20992039 | Amber |
| chr16 | 21754756 | Teus |
| chr16 | 22611461 | Heineken |
| chr16 | 25605581 | Djembe |
| chr16 | 26113090 | Teus |
| chr16 | 28833152 | Magenta |
| chr16 | 29796630 | Amber |
| chr16 | 32932286 | Djembe |
| chr16 | 51120444 | Hoegaarde |
| chr16 | 60191875 | Hoegaarde |
| chr16 | 61354698 | Djembe |
| chr16 | 64148953 | Magenta |
| chr16 | 68218150 | Amber |
| chr16 | 71650256 | Delta |
| chr16 | 71689206 | Lithium |
| chr16 | 72969792 | Elanor |
| chr16 | 77053703 | Leffe |
| chr17 | 1619157 | Delta |
| chr17 | 5378735 | Chalk |
| chr17 | 14126680 | Khan |
| chr17 | 15952233 | Mowgli |
| chr17 | 16313579 | Mowgli |
| chr17 | 23685699 | Babet |
| chr17 | 25577677 | Lithium |
| chr17 | 32093993 | Teus |
| chr17 | 32670805 | Delta |
| chr17 | 37339537 | Elanor |
| chr17 | 39791241 | Leffe |
| chr17 | 44067384 | Hoegaarde |
| chr17 | 47691218 | Grolsch |
| chr17 | 54477919 | Sir |
| chr17 | 55534358 | Elanor |
| chr17 | 60227360 | Sir |
| chr17 | 68380313 | Teus |
| chr17 | 68486644 | Teus |
| chr17 | 77134169 | Bavaria |
| chr17 | 78300571 | Teus |
| chr17 | 86070190 | Khan |
| chr17 | 88174686 | Hoegaarde |
| chr17 | 88174686 | Babet |
| chr17 | 91058285 | Honoria |
| chr17 | 91292845 | Amber |
| chr17 | 94884327 | Mowgli |
| chr18 | 130358 | Grolsch |
| chr18 | 1076194 | Magenta |
| chr18 | 2359170 | Bridgit |
| chr18 | 3984166 | Sir |
| chr18 | 6755131 | Magenta |
| chr18 | 13153323 | Khan |
| chr18 | 16503127 | Sir |
| chr18 | 19912590 | Grolsch |
| chr18 | 20649499 | Bridgit |
| chr18 | 25962135 | Bavaria |
| chr18 | 36364204 | Teus |
| chr18 | 38243917 | Babet |
| chr18 | 39194182 | Mowgli |
| chr18 | 52011483 | Honoria |
| chr18 | 52613966 | Amber |
| chr18 | 54064903 | Bavaria |
| chr18 | 60586708 | Babet |
| chr18 | 63760082 | Amber |
| chr18 | 65587448 | Teus |
| chr19 | 2097806 | Amber |
| chr19 | 3220428 | Bavaria |
| chr19 | 5090522 | Djembe |
| chr19 | 5892999 | Grolsch |
| chr19 | 7047030 | Leffe |
| chr19 | 7047030 | Djembe |
| chr19 | 7210693 | Khan |
| chr19 | 10572417 | Lithium |
| chr19 | 12946646 | Mowgli |
| chr19 | 15861061 | Lithium |
| chr19 | 15861061 | Bavaria |
| chr19 | 17081730 | Sir |
| chr19 | 17591230 | Leffe |
| chr19 | 17819175 | Delta |
| chr19 | 18295501 | Amber |
| chr19 | 19444770 | Sir |
| chr19 | 21628808 | Leffe |
| chr19 | 22738226 | Grolsch |
| chr19 | 25607494 | Elanor |
| chr19 | 32828883 | Amber |
| chr19 | 43263630 | Bridgit |
| chr19 | 48866998 | Khan |
| chr19 | 49649036 | Lithium |
| chr19 | 50740231 | Bridgit |
| chr19 | 51702050 | Leffe |
| chr2 | 6186347 | Babet |
| chr2 | 16907323 | Djembe |
| chr2 | 18311354 | Hoegaarde |
| chr2 | 20904917 | Leffe |
| chr2 | 25883494 | Mowgli |
| chr2 | 27937337 | Mowgli |
| chr2 | 30348976 | Honoria |
| chr2 | 36116961 | Leffe |
| chr2 | 40698737 | Bridgit |
| chr2 | 54469953 | Teus |
| chr2 | 55218358 | Delta |
| chr2 | 55987952 | Lithium |
| chr2 | 58666039 | Khan |
| chr2 | 61574000 | Khan |
| chr2 | 64251799 | Magenta |
| chr2 | 66084232 | Teus |
| chr2 | 73427126 | Grolsch |
| chr2 | 74520227 | Delta |
| chr2 | 82802454 | Grolsch |
| chr2 | 86931223 | Khan |
| chr2 | 96390612 | Elanor |
| chr2 | 97320566 | Honoria |
| chr2 | 100527498 | Leffe |
| chr2 | 101979137 | Khan |
| chr2 | 101979137 | Delta |
| chr2 | 107372570 | Sir |
| chr2 | 114767373 | Bavaria |
| chr2 | 114786987 | Elanor |
| chr2 | 139118703 | Hoegaarde |
| chr2 | 145544102 | Leffe |
| chr2 | 150925215 | Teus |
| chr2 | 150925220 | Teus |
| chr2 | 150987129 | Heineken |
| chr2 | 158605091 | Mowgli |
| chr2 | 170306259 | Sir |
| chr2 | 173906056 | Teus |
| chr2 | 176077475 | Teus |
| chr2 | 180743289 | Sir |
| chr2 | 182405827 | Lithium |
| chr2 | 184229214 | Bavaria |
| chr2 | 187460458 | Khan |
| chr2 | 191144737 | Sir |
| chr2 | 196146158 | Grolsch |
| chr2 | 202555658 | Hoegaarde |
| chr2 | 203383064 | Heineken |
| chr20 | 4553237 | Amber |
| chr20 | 8199997 | Lithium |
| chr20 | 9871372 | Bridgit |
| chr20 | 13401630 | Delta |
| chr20 | 18343669 | Bridgit |
| chr20 | 18467373 | Sir |
| chr20 | 22714487 | Bavaria |
| chr20 | 23937445 | Khan |
| chr20 | 30114102 | Djembe |
| chr20 | 37442645 | Magenta |
| chr20 | 43374745 | Teus |
| chr20 | 52618486 | Leffe |
| chr20 | 58853018 | Babet |
| chr20 | 65072221 | Lithium |
| chr20 | 70622610 | Mowgli |
| chr20 | 74282390 | Elanor |
| chr3 | 2334952 | Bridgit |
| chr3 | 5336254 | Bavaria |
| chr3 | 7658417 | Babet |
| chr3 | 8869914 | Bavaria |
| chr3 | 11702294 | Mowgli |
| chr3 | 13227115 | Delta |
| chr3 | 18612664 | Teus |
| chr3 | 19034268 | Delta |
| chr3 | 27283010 | Teus |
| chr3 | 32459576 | Grolsch |
| chr3 | 35051907 | Honoria |
| chr3 | 37965981 | Hoegaarde |
| chr3 | 38232621 | Bavaria |
| chr3 | 38459571 | Khan |
| chr3 | 40893728 | Khan |
| chr3 | 42002658 | Bavaria |
| chr3 | 48018105 | Elanor |
| chr3 | 52705646 | Khan |
| chr3 | 56407451 | Sir |
| chr3 | 56650994 | Mowgli |
| chr3 | 59093335 | Teus |
| chr3 | 59465257 | Teus |
| chr3 | 66670884 | Babet |
| chr3 | 67351945 | Hoegaarde |
| chr3 | 70724444 | Teus |
| chr3 | 76549728 | Heineken |
| chr3 | 79646348 | Elanor |
| chr3 | 81768551 | Grolsch |
| chr3 | 82954409 | Sir |
| chr3 | 86260197 | Babet |
| chr3 | 96442357 | Sir |
| chr3 | 97705142 | Lithium |
| chr3 | 99101546 | Mowgli |
| chr3 | 106318080 | Teus |
| chr3 | 106536046 | Mowgli |
| chr3 | 109219038 | Honoria |
| chr3 | 110606907 | Bavaria |
| chr3 | 115147400 | Sir |
| chr3 | 121158979 | Khan |
| chr3 | 122985570 | Teus |
| chr3 | 132535106 | Bridgit |
| chr3 | 133314622 | Magenta |
| chr3 | 134667804 | Sir |
| chr3 | 143017307 | Grolsch |
| chr3 | 144393672 | Leffe |
| chr3 | 154047737 | Lithium |
| chr3 | 154330587 | Mowgli |
| chr3 | 157847415 | Honoria |
| chr3 | 165797921 | Honoria |
| chr3 | 165809938 | Lithium |
| chr3 | 175699011 | Babet |
| chr3 | 178705347 | Djembe |
| chr3 | 179004718 | Khan |
| chr3 | 182031589 | Elanor |
| chr4 | 1959550 | Amber |
| chr4 | 6724953 | Babet |
| chr4 | 7146167 | Bavaria |
| chr4 | 9777185 | Delta |
| chr4 | 10867607 | Bavaria |
| chr4 | 12928946 | Grolsch |
| chr4 | 26790870 | Khan |
| chr4 | 30972657 | Teus |
| chr4 | 33344029 | Bavaria |
| chr4 | 48938264 | Delta |
| chr4 | 61793782 | Hoegaarde |
| chr4 | 64077263 | Khan |
| chr4 | 66622299 | Mowgli |
| chr4 | 67377202 | Teus |
| chr4 | 71984472 | Babet |
| chr4 | 81765863 | Amber |
| chr4 | 90177997 | Elanor |
| chr4 | 93063769 | Mowgli |
| chr4 | 93912703 | Leffe |
| chr4 | 94721684 | Bavaria |
| chr4 | 106451604 | Sir |
| chr4 | 114147528 | Bavaria |
| chr4 | 114616258 | Djembe |
| chr4 | 116799040 | Heineken |
| chr4 | 118948518 | Bavaria |
| chr4 | 124314237 | Amber |
| chr4 | 126242052 | Babet |
| chr4 | 126813609 | Lithium |
| chr4 | 132488141 | Honoria |
| chr4 | 132488144 | Honoria |
| chr4 | 137393964 | Teus |
| chr4 | 138934253 | Leffe |
| chr4 | 153488763 | Lithium |
| chr4 | 158959577 | Elanor |
| chr4 | 166270126 | Elanor |
| chr4 | 169401407 | Honoria |
| chr4 | 171311227 | Amber |
| chr5 | 1242411 | Amber |
| chr5 | 3938510 | Hoegaarde |
| chr5 | 7729746 | Hoegaarde |
| chr5 | 16206162 | Teus |
| chr5 | 20565233 | Bavaria |
| chr5 | 20728248 | Magenta |
| chr5 | 34550703 | Amber |
| chr5 | 44640350 | Babet |
| chr5 | 44642022 | Teus |
| chr5 | 50587215 | Bavaria |
| chr5 | 50884615 | Teus |
| chr5 | 53949313 | Delta |
| chr5 | 53959633 | Magenta |
| chr5 | 56378328 | Elanor |
| chr5 | 65679377 | Mowgli |
| chr5 | 69428450 | Elanor |
| chr5 | 69974944 | Teus |
| chr5 | 97020404 | Mowgli |
| chr5 | 107745108 | Grolsch |
| chr5 | 118164310 | Chalk |
| chr5 | 118992462 | Heineken |
| chr5 | 120100420 | Khan |
| chr5 | 129321794 | Magenta |
| chr5 | 148722637 | Hoegaarde |
| chr5 | 154643602 | Sir |
| chr5 | 155505806 | Grolsch |
| chr5 | 155964643 | Bavaria |
| chr5 | 156702035 | Djembe |
| chr5 | 164016598 | Elanor |
| chr5 | 174689577 | Teus |
| chr5 | 183617828 | Djembe |
| chr5 | 183732053 | Bridgit |
| chr6 | 765094 | Bavaria |
| chr6 | 5196487 | Mowgli |
| chr6 | 7813314 | Khan |
| chr6 | 9291440 | Mowgli |
| chr6 | 11071739 | Mowgli |
| chr6 | 11241018 | Heineken |
| chr6 | 12034003 | Delta |
| chr6 | 15059432 | Lithium |
| chr6 | 18650255 | Heineken |
| chr6 | 20126258 | Elanor |
| chr6 | 20320058 | Teus |
| chr6 | 22850227 | Teus |
| chr6 | 23140424 | Babet |
| chr6 | 28542576 | Chalk |
| chr6 | 30402940 | Delta |
| chr6 | 32652183 | Magenta |
| chr6 | 35805918 | Chalk |
| chr6 | 38024023 | Grolsch |
| chr6 | 42315981 | Grolsch |
| chr6 | 46658512 | Babet |
| chr6 | 48632708 | Chalk |
| chr6 | 52172222 | Lithium |
| chr6 | 57410008 | Leffe |
| chr6 | 57451412 | Djembe |
| chr6 | 60360620 | Babet |
| chr6 | 64657185 | Heineken |
| chr6 | 71901241 | Leffe |
| chr6 | 80396610 | Khan |
| chr6 | 81734741 | Magenta |
| chr6 | 89687544 | Chalk |
| chr6 | 91329337 | Magenta |
| chr6 | 93210113 | Magenta |
| chr6 | 94115753 | Bridgit |
| chr6 | 103176391 | Amber |
| chr6 | 104840625 | Djembe |
| chr6 | 106140174 | Sir |
| chr6 | 115324661 | Amber |
| chr6 | 119619116 | Lithium |
| chr6 | 119793997 | Mowgli |
| chr6 | 121630138 | Teus |
| chr6 | 121844825 | Delta |
| chr6 | 129982623 | Mowgli |
| chr6 | 132663101 | Amber |
| chr6 | 132663101 | Babet |
| chr6 | 133656929 | Amber |
| chr6 | 135062402 | Grolsch |
| chr6 | 138085154 | Bavaria |
| chr6 | 140652935 | Chalk |
| chr6 | 145671453 | Sir |
| chr6 | 151956335 | Delta |
| chr6 | 153130648 | Bavaria |
| chr6 | 154103980 | Magenta |
| chr6 | 157547189 | Heineken |
| chr6 | 157873190 | Djembe |
| chr6 | 172688292 | Chalk |
| chr6 | 173195624 | Amber |
| chr6 | 177138869 | Hoegaarde |
| chr7 | 4979922 | Khan |
| chr7 | 9228939 | Magenta |
| chr7 | 20660774 | Grolsch |
| chr7 | 30570470 | Lithium |
| chr7 | 46161032 | Lithium |
| chr7 | 48896157 | Magenta |
| chr7 | 50139568 | Chalk |
| chr7 | 52723770 | Honoria |
| chr7 | 54598313 | Teus |
| chr7 | 54868638 | Babet |
| chr7 | 55183040 | Delta |
| chr7 | 57482274 | Leffe |
| chr7 | 57657681 | Chalk |
| chr7 | 60635102 | Honoria |
| chr7 | 60635102 | Sir |
| chr7 | 62675476 | Hoegaarde |
| chr7 | 64949260 | Teus |
| chr7 | 64958809 | Sir |
| chr7 | 66677690 | Honoria |
| chr7 | 66774766 | Honoria |
| chr7 | 67833523 | Lithium |
| chr7 | 72583415 | Amber |
| chr7 | 73123488 | Delta |
| chr7 | 73310017 | Khan |
| chr7 | 74974684 | Babet |
| chr7 | 86063717 | Leffe |
| chr7 | 91211079 | Honoria |
| chr7 | 96509601 | Elanor |
| chr7 | 102736679 | Delta |
| chr7 | 103747150 | Lithium |
| chr7 | 112353992 | Babet |
| chr7 | 116648579 | Amber |
| chr7 | 116648579 | Babet |
| chr7 | 136289641 | Honoria |
| chr7 | 141679375 | Hoegaarde |
| chr7 | 149216879 | Heineken |
| chr7 | 153210388 | Heineken |
| chr7 | 155098466 | Lithium |
| chr7 | 163141580 | Teus |
| chr7 | 168616166 | Hoegaarde |
| chr7 | 168616168 | Hoegaarde |
| chr7 | 168802031 | Bavaria |
| chr8 | 11458034 | Heineken |
| chr8 | 17723281 | Babet |
| chr8 | 18095066 | Lithium |
| chr8 | 31015066 | Bridgit |
| chr8 | 33516891 | Elanor |
| chr8 | 53326953 | Chalk |
| chr8 | 78486943 | Khan |
| chr8 | 84871205 | Mowgli |
| chr8 | 87439074 | Mowgli |
| chr8 | 91232593 | Elanor |
| chr8 | 91609198 | Sir |
| chr8 | 95416772 | Grolsch |
| chr8 | 103508305 | Teus |
| chr8 | 109477243 | Elanor |
| chr8 | 113872383 | Grolsch |
| chr8 | 126345912 | Delta |
| chr8 | 138243343 | Chalk |
| chr8 | 141524968 | Bridgit |
| chr9 | 332616 | Heineken |
| chr9 | 7035308 | Teus |
| chr9 | 9125709 | Magenta |
| chr9 | 27119221 | Hoegaarde |
| chr9 | 27226909 | Amber |
| chr9 | 32544257 | Hoegaarde |
| chr9 | 32544257 | Babet |
| chr9 | 32544257 | Magenta |
| chr9 | 32544257 | Djembe |
| chr9 | 34507346 | Bavaria |
| chr9 | 35450081 | Chalk |
| chr9 | 35499077 | Mowgli |
| chr9 | 35992733 | Heineken |
| chr9 | 37785254 | Chalk |
| chr9 | 41324399 | Honoria |
| chr9 | 57966486 | Heineken |
| chr9 | 58787884 | Heineken |
| chr9 | 59187032 | Heineken |
| chr9 | 61296657 | Mowgli |
| chr9 | 64372602 | Delta |
| chr9 | 68967618 | Magenta |
| chr9 | 73834301 | Elanor |
| chr9 | 76000046 | Hoegaarde |
| chr9 | 76213490 | Sir |
| chr9 | 78998833 | Khan |
| chr9 | 81526225 | Babet |
| chr9 | 86127438 | Lithium |
| chr9 | 88707756 | Heineken |
| chr9 | 93536046 | Heineken |
| chr9 | 95177521 | Delta |
| chr9 | 96731016 | Honoria |
| chr9 | 102278822 | Amber |
| chr9 | 103409012 | Mowgli |
| chr9 | 105774642 | Babet |
| chr9 | 109215135 | Teus |
| chr9 | 114164171 | Grolsch |
| chr9 | 119148426 | Delta |
| chr9 | 125284007 | Heineken |
| chr9 | 129744621 | Amber |

**Table S3 – Position of the clustered mutations.**

| Chromosome | Position | Reference allele | Alternative allele | Sample |
| --- | --- | --- | --- | --- |
| chr2 | 114767373 | C | G | Bavaria |
| chr2 | 114786987 | C | T | Elanor |
| chr2 | 150925215 | G | A | Teus |
| chr2 | 150925220 | A | T | Teus |
| chr3 | 165797921 | C | T | Honoria |
| chr3 | 165809938 | C | T | Lithium |
| chr4 | 132488141 | G | T | Honoria |
| chr4 | 132488144 | C | T | Honoria |
| chr5 | 44640350 | G | A | Babet |
| chr5 | 44642022 | G | T | Teus |
| chr5 | 53949313 | T | G | Delta |
| chr5 | 53959633 | G | A | Magenta |
| chr7 | 64949260 | G | T | Teus |
| chr7 | 64958809 | C | T | Sir |
| chr7 | 168616166 | C | T | Hoegaarde |
| chr7 | 168616168 | A | T | Hoegaarde |
| chr10 | 67704591 | C | G | Chalk |
| chr10 | 67704672 | A | G | Chalk |
| chr10 | 67720113 | A | G | Sir |
| chr12 | 55952972 | T | C | Teus |
| chr12 | 55952973 | C | A | Teus |
| chr12 | 59966197 | G | C | Hoegaarde |
| chr12 | 59979880 | A | T | Bridgit |

**Table S4 – Number of candidates after each filter.**

| Trio | Mendelian violation (parents homozygote reference Ref and offsprings heterozygote) | Allelic balance filter | Depth filter | Genotype quality filter (and remove shared between non-related individuals ) | Potential false-positive calls |
| --- | --- | --- | --- | --- | --- |
| Amber | 9833 | 4337 | 777 | 44 | 4 |
| Babet | 8928 | 4070 | 702 | 43 | 6 |
| Bavaria | 10045 | 4275 | 813 | 40 | 3 |
| Bridgit | 9079 | 4018 | 661 | 38 | 9 |
| Chalk | 9016 | 3970 | 713 | 30 | 5 |
| Delta | 9492 | 4326 | 716 | 51 | 7 |
| Djembe | 9624 | 4288 | 708 | 32 | 2 |
| Elanor | 9614 | 4272 | 524 | 41 | 4 |
| Grolsch | 9565 | 4112 | 694 | 34 | 3 |
| Heineken | 9623 | 4155 | 727 | 31 | 2 |
| Hoegaarde | 9546 | 4229 | 745 | 39 | 7 |
| Honoria | 9001 | 3982 | 720 | 27 | 1 |
| Khan | 8417 | 3851 | 644 | 42 | 4 |
| Leffe | 10121 | 4306 | 746 | 25 | 3 |
| Lithium | 9426 | 4044 | 712 | 46 | 8 |
| Magenta | 9195 | 4140 | 744 | 36 | 3 |
| Mowgli | 9125 | 4171 | 610 | 43 | 4 |
| Sir | 9085 | 4047 | 583 | 43 | 4 |
| Teus | 8492 | 3746 | 712 | 59 | 2 |

**Table S5: Primers used for PCR validation and sequencing of *de novo* candidates for each individual** ie F: father (Noot), M: mother (Platina) and O: offspring (Lithium) along with sequences’ ID and corresponding Genbank accession numbers.

| **Individual** | ***de novo* candidate position on rheMac8** | **Forward Primer Name** | **Forward Primer sequence (5́-3́)** | **Reverse Primer Name** | **Reverse Primer sequence (5́-3́)** | **SequenceID** | **Accession number** |
| --- | --- | --- | --- | --- | --- | --- | --- |
| M/O | chr1:185853213‬ | Chr1_3_F1 | AGGCCTCTAGAGTTTTGCCAC | Chr1_3_R1 | TGGCTGATACGAAGGCTGTG | EF31080861/ EF31079373 | MT426056/  MT426047 |
| F |  | Chr1_3_F2 | GCCTCTAGAGTTTTGCCACG | Chr1_3_R2 | GGCTGATACGAAGGCTGTGA | EF01771473 | MT426020 |
| M/O | chr2:55987952 | Chr2_21_F1 | GAGCCTGGCTTAGGGAACAG | Chr2_21_R1 | CGAGTCAGAAGAGCCAGCAA | EF31080869/ EF31079349 | MT426061/  MT426037 |
| F |  | Chr2_21_F2 | AGCCAGGAGAGGTTCTCAGT | Chr2_21_R2 | CACTGCGCGAGTCAGAAGAG | EF31080961 | MT426084 |
| M/O | chr2:182405827 | Chr2_22_F1 | ATCAGTTCAGGATCTGCCCG | Chr2_22_R1 | CCACACGAGTGTACACAGCA | EF31080870/ EF31079350 | MT426062/  MT426038 |
| F |  | Chr2_22_F2 | CAATCCCTTGATGCCTCCCT | Chr2_22_R2 | CCACAATGCAAACCACCACC | EF31080963 | MT426085 |
| M/O | chr3:154047737 | Chr3_25_F1 | CTCCCCACCCATTCCACATC | Chr3_25_R1 | GGTGCTATGCGGCATTGTTT | EF31080874/ EF31079352 | MT426063/  MT426039 |
| F |  | Chr3_25_F2 | CCCACCCATTCCACATCAGT | Chr3_25_R2 | TTGATATGGTGCTATGCGGC | EF31080966 | MT426086 |
| M/O | chr3:165809938 | Chr3_26_F1 | GGACCCTGGACAAATGGAGG | Chr3_26_R1 | GTGTTTGGCGTTCACATCCC | EF31080910/ EF31079354 | MT426076/  MT426040 |
| F |  | Chr3_26_F2 | AGAGGAGGGAGAAAAGCCCA | Chr3_26_R2 | GGTGGTTTTGTTCTGCGAGC | EF31080967 | MT426087 |
| M/O | chr4:153488763 | Chr4_27_F1 | TGGGAACTGGCCATGATGTA | Chr4_27_R1 | GCTGCTGTGATAGCAACCCT | EF31080876/ EF31079355 | MT426064/  MT426041 |
| F |  | Chr4_27_F2 | TGGGAACTGGCCATGATGTAA | Chr4_27_R2 | AGCTGCTGTGATAGCAACCC | EF31079456 | MT426051 |
| M/O | chr5:53672805 | Chr5_28_F1 | CACAATAACATTCTCCAGTCTCCTG | Chr5_28_R1 | CCGGCCGATCCTCAGTATTT | EF31080877/ EF31079356 | MT426052/  MT426042 |
| F |  | Chr5_28_F2 | ACAATAACATTCTCCAGTCTCCTG | Chr5_28_R2 | GGCCGATCCTCAGTATTTCCA | EF31079458 | MT426065 |
| M/O | chr6:15059432 | Chr6_29_F1 | GGGGGACTATGCATCCAACT | Chr6_29_R1 | CAGACAATGCATCAGTTTTCAGT | EF31080879/ EF31079357 | MT426066/  MT426043 |
| F |  | Chr6_29_F2 | TGTTAGTGCTGTGGTACTGGG | Chr6_29_R2 | GACAATGCATCAGTTTTCAGTTTCT | EF31079459 | MT426053 |
| M/O | chr6:52172222 | Chr6_30_F1 | TTCCCATGGTGTGGGATGTG | Chr6_30_R1 | TGAATCGGGTGAGAACTCAGG | EF31080880/ EF31079358 | MT426067/  MT426044 |
| F |  | Chr6_30_F2 | CCCATGGTGTGGGATGTGTG | Chr6_30_R2 | GCAAAATAAGATGAATCGGGTGAG | EF31079460 | MT426054 |
| M/O | chr7:30570470 | Chr7_32_F1 | GGGACCTGGTGGGAGGTTAT | Chr7_32_R1 | GGAGGGAAAGGAAGCTGCTC | EF31080881/ EF01772088 | MT426068/  MT426025 |
| F |  | Chr7_32_F2 | AGGTCACTCTTTGTGTTTGGCT | Chr7_32_R2 | ACCAAGTTCTCTGGAGGGAAAG | EF31079463 | MT426055 |
| M/O | chr7:103747150 | Chr7_35_F1 | GTTGCTGACAAAATGTCTCGGT | Chr7_35_R1 | AACGCCACCACTTGGGAAAA | EF31080883/ EF31079408 | MT426069/  MT426050 |
| F |  | Chr7_35_F2 | GGTTGCTGACAAAATGTCTCGG | Chr7_35_R2 | AACGCCACCACTTGGGAAA | EF01772034 | MT426023 |
| M/O | chr7:155098466 | Chr7_36_F1 | CTGGATAAGCCCCTGCATGT | Chr7_36_R1 | GAATTGGATGAGGCCAGGGG | EF31080884/ EF31079371 | MT426070/  MT426045 |
| F |  | Chr7_36_F2 | CAAGGCTCGCAGACACAAAG | Chr7_36_R2 | GGAATTGGATGAGGCCAGGG | EF01770601 | MT426016 |
| M/O | chr8:18095066 | Chr8_37_F1 | ACCTCAAAGAACTAGAAAAGCAGG | Chr8_37_R1 | TCAGTGTGTCATTGTCTGGCT | EF31080885/ EF31079372 | MT426071/  MT426046 |
| F |  | Chr8_37_F2 | ACCTCAAAGAACTAGAAAAGCAGGA | Chr8_37_R2 | TGGCCTTGTAGAATGTGCCTG | EF01770796 | MT426017 |
| M | chr9:86127438 | Chr9_38_F1 | TGATTATTAAGGGAACAGACCAGT | Chr9_38_R1 | AGCTGTGCTGACCTTATGCT | EF31080886 | MT426072 |
| O/F |  | Chr9_38_F2 | TTGATTATTAAGGGAACAGACCAGT | Chr9_38_R2 | AAGGCATGGCAGATGGAAGA | EF01772095/ EF01770995 | MT426026/  MT426018 |
| M/O | chr10:2998418 | Chr10_4_F1 | CCAGCTCTCCCCTATCCCTTA | Chr10_4_R1 | CTGCTACTTCTGCGTTGGTG | EF31080862/ EF31079329 | MT426057/  MT426031 |
| F |  | Chr10_4_F2 | AAGTAGCCAGCTCTCCCCTAT | Chr10_4_R2 | TTGGTCTCATTTCCTGTGGGG | EF31080943 | MT426077 |
| M/O | chr10:61697270‬‬ | Chr10_5_F1 | CTGCTTTCTCTGCAGCACAC | Chr10_5_R1 | TCAAAGCTGCAAGGAGGGTT | EF01772118/ EF31079375 | MT426028/  MT426048 |
| F |  | Chr10_5_F2 | CCGTGTGGTGTCCATACTGT | Chr10_5_R2 | ATCAAAGCTGCAAGGAGGGT | EF31080944 | MT426078 |
| M/O | chr11:69917066 | Chr11_6_F1 | TGCCCAAATAATGTTTCCAACC | Chr11_6_R1 | TTTCCCTTCTGCCTTGCTCA | EF31080863/ EF31079331 | MT426058/  MT426032 |
| F |  | Chr11_6_F2 | TTGCCCAAATAATGTTTCCAACCT | Chr11_6_R2 | TGCCTTGCTCAAAGTCAAGC | EF31080946 | MT426079 |
| M/O | chr11:85401390‬ | Chr11_7_F1 | ATCCCACCTTTCCCATTCTGTC | Chr11_7_R1 | AGTCCTGTAGCTCTCACTGT | EF31080898/ EF31079332 | MT426075/  MT426033 |
| F |  | Chr11_7_F2 | AGCTTGTTCACACGTGCTAATG | Chr11_7_R2 | TGCTGTTTGTGGCTGTCTTTA | EF31080947 | MT426080 |
| M/O | chr11:105492721 | Chr11_8_F1 | CCTTTCTCTGGGTTGGTGGT | Chr11_8_R1 | AAGTCCAGTGCCCCCTTTTC | EF31080865/ EF31079333 | MT426059/  MT426034 |
| F |  | Chr11_8_F2 | CTCCCCCTTCCTGCTTTAGG | Chr11_8_R2 | GAAGTCCAGTGCCCCCTTTT | EF01771716 | MT426021 |
| O | chr14:23903603 | Chr14_12_F1 | AGGGAAATCATGTCTGGGCT | Chr14_12_R1 | TCACAAGGACAGCACCAAGG | EF31079386 | MT426049 |
| M/F |  | Chr14_12_F2 | CCATGGAACAGAGATAGGGCA | Chr14_12_R2 | TGCCCCCACATGATCCAATC | EF31080888/ EF31080952 | MT426074/  MT426081 |
| M/O | chr14:95851026 | Chr14_13_F1 | ACTCACTTTCACCACCAACCA | Chr14_13_R1 | TGCCAATGCTGCTTTCTTGG | EF31080867/ EF31079339 | MT426060/  MT426035 |
| F |  | Chr14_13_F2 | CTGTGCCAAATTGGACTGGTT | Chr14_13_R2 | ACTGCCAATGCTGCTTTCTTG | EF31080953 | MT426082 |
| O | chr17:25577677 | Chr17_17_F1 | GCATGTGCTACTATACCCGACT | Chr17_17_R1 | GGCAGGAAATGCACATCACAC | EF31079344 | MT426036 |
| M/F |  | Chr17_17_F2 | GGCATGTGCTACTATACCCGAC | Chr17_17_R2 | TTGGCCAAAGCCTGTATGCG | EF01772180/ EF01771915 | MT426030/  MT426022 |
| F/M/O | chr19:49649036 | Chr19_20_F2 | TGGGACGCTTAACTCTGCTC | Chr19_20_R2 | TAACACGGTGAAGCCCTGTC | EF31080960/ EF01772128/ EF01772066 | MT426083/  MT426029/  MT426024 |
| M | chr20:8199997 | Chr20_39_F1 | GCCTCCACCAAAGTCTCAAA | Chr20_39_R1 | CTGGGGCAGAATATCATGGG | EF31080887 | MT426073 |
| F/O |  | Chr20_39_F2 | ACGAACTGTCAAGTGTGAGG | Chr20_39_R2 | GGACCAGGAAGGGAGACTTA | EF01771092/ EF01772109 | MT426019/  MT426027 |

**Appendix 1. PCR amplification and sequencing validation of *de novo* candidates.**

PCRs were carried out in 25μL volumes [2.5 units Dream Taq DNA Polymerase (**Thermo Scientific)**, 1X Dream Taq Green Buffer, 0.2mM dNTPs, 3–4.5mM MgCl2, 0.25μL DNA template, filled to 25μL with double-distilled (ddH2O) water]. Thermocycling was performed in a BIORAD PTC-100 thermocycler. The cycle program comprised of an initial denaturation at 95°C for 2min, followed by 35 cycles of 15sec at 95°C, 15sec at 52°C and 30sec at 72°C. Cycling was terminated with a 5min extension at 72°C. PCR products were purified using commercially available spin columns (Invitek). Sanger sequencing was conducted at Eurofins Genomics, Europe using the primers of the amplification procedure using both forward and reverse primers. In Supplementary Fig 2 we provide the chromatograms with the best base quality values.

**Appendix 2. Bam files of the 81 manually curated *de novo* candidates (in the following order and for each panel with the father on the top, the mother in the middle and the offspring in the bottom)**

| Offspring | Chrom | Position |
| --- | --- | --- |
| amber | chr3 | 184747719 |
| amber | chr5 | 41059414 |
| amber | chr11 | 19863816 |
| amber | chr14 | 41732848 |
| babet | chr1 | 67409640 |
| babet | chr1 | 67409644 |
| babet | chr6 | 175439564 |
| babet | chr6 | 175439570 |
| babet | chr12 | 49322712 |
| babet | chr20 | 16318291 |
| bavaria | chr2 | 47299154 |
| bavaria | chr2 | 198249314 |
| bavaria | chr2 | 198249320 |
| bridgit | chr3 | 8994919 |
| bridgit | chr3 | 8994920 |
| bridgit | chr3 | 8994921 |
| bridgit | chr3 | 8994925 |
| bridgit | chr11 | 25851523 |
| bridgit | chr11 | 100469834 |
| bridgit | chr12 | 86404281 |
| bridgit | chr12 | 86404283 |
| bridgit | chr16 | 27496930 |
| chalk | chr4 | 138349903 |
| chalk | chr8 | 80891860 |
| chalk | chr9 | 72557750 |
| chalk | chr15 | 33948114 |
| chalk | chr17 | 17487715 |
| delta | chr5 | 14512753 |
| delta | chr6 | 8707858 |
| delta | chr6 | 8707860 |
| delta | chr12 | 43095987 |
| delta | chr12 | 86404283 |
| delta | chr19 | 53043343 |
| delta | chr19 | 53043350 |
| djembe | chr4 | 33961790 |
| djembe | chr12 | 13775326 |
| elanor | chr3 | 62481887 |
| elanor | chr5 | 14512753 |
| elanor | chr13 | 83261061 |
| elanor | chr19 | 4743401 |
| grolsch | chr2 | 47299154 |
| grolsch | chr10 | 64100223 |
| grolsch | chr15 | 105733276 |
| heineken | chr1 | 3542500 |
| heineken | chr17 | 5262098 |
| hoegaarde | chr2 | 56360306 |
| hoegaarde | chr2 | 56360308 |
| hoegaarde | chr3 | 2331456 |
| hoegaarde | chr4 | 33324777 |
| hoegaarde | chr7 | 32345457 |
| hoegaarde | chr16 | 77061593 |
| hoegaarde | chr18 | 49013245 |
| honoria | chr16 | 65096277 |
| khan | chr3 | 43779682 |
| khan | chr4 | 31711469 |
| khan | chr5 | 189290185 |
| khan | chr16 | 76984110 |
| leffe | chr3 | 46670148 |
| leffe | chr12 | 123801792 |
| leffe | chr17 | 93084961 |
| lithium | chr5 | 53672805 |
| lithium | chr7 | 66081252 |
| lithium | chr10 | 84589564 |
| lithium | chr11 | 57612148 |
| lithium | chr12 | 1837681 |
| lithium | chr15 | 55438949 |
| lithium | chr15 | 55438951 |
| lithium | chr18 | 55382327 |
| magenta | chr1 | 67409635 |
| magenta | chr2 | 132903035 |
| magenta | chr6 | 29757626 |
| mowgli | chr3 | 11702296 |
| mowgli | chr3 | 11702298 |
| mowgli | chr9 | 107503801 |
| mowgli | chr9 | 107503809 |
| sir | chr5 | 14512753 |
| sir | chr14 | 115143833 |
| sir | chr19 | 53043343 |
| sir | chr19 | 53043350 |
| teus | chr7 | 83438313 |
| teus | chr13 | 26422007 |


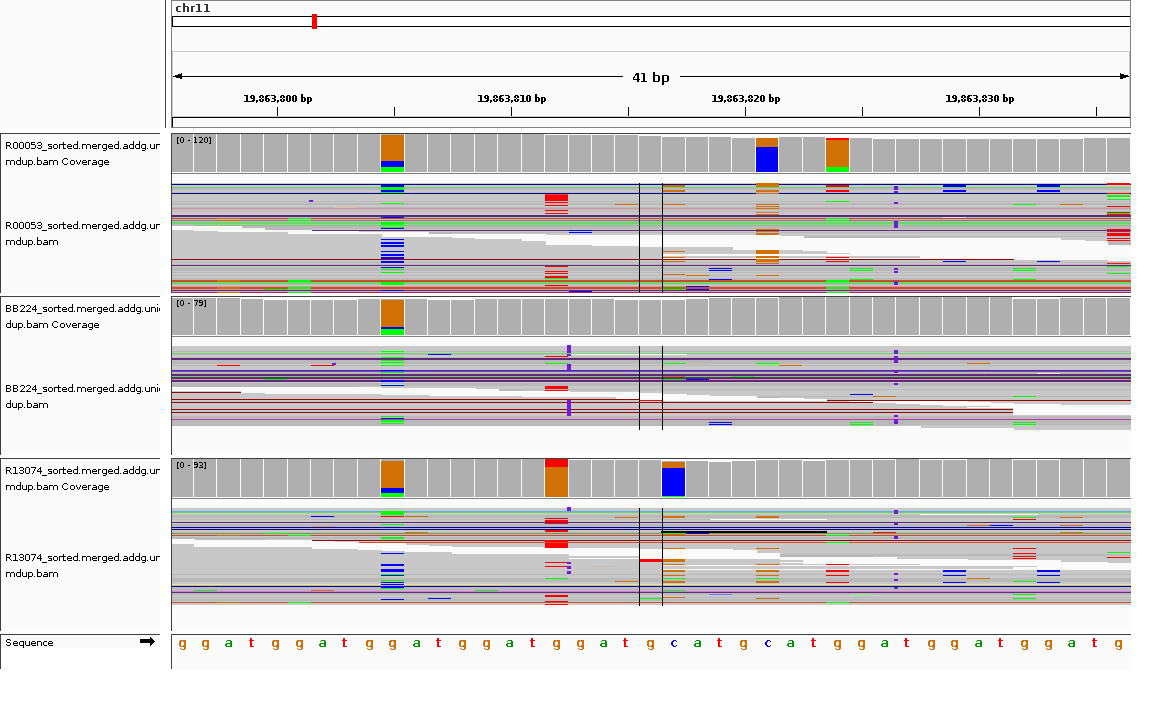

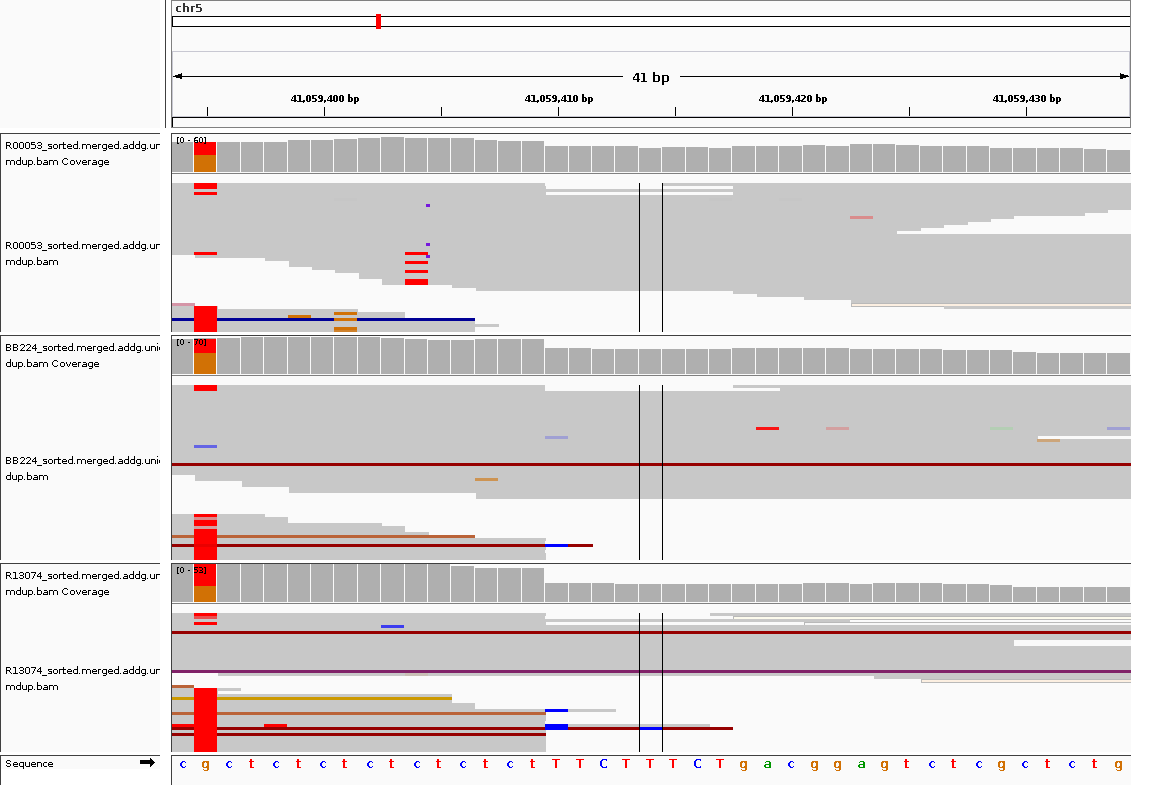

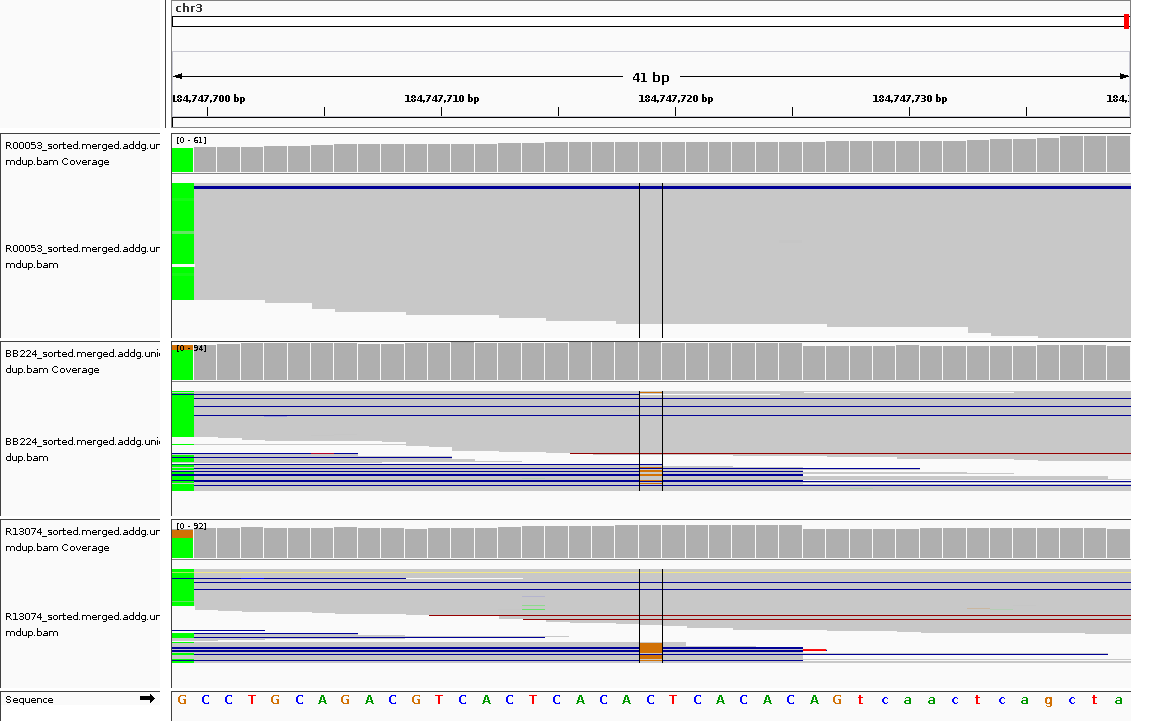

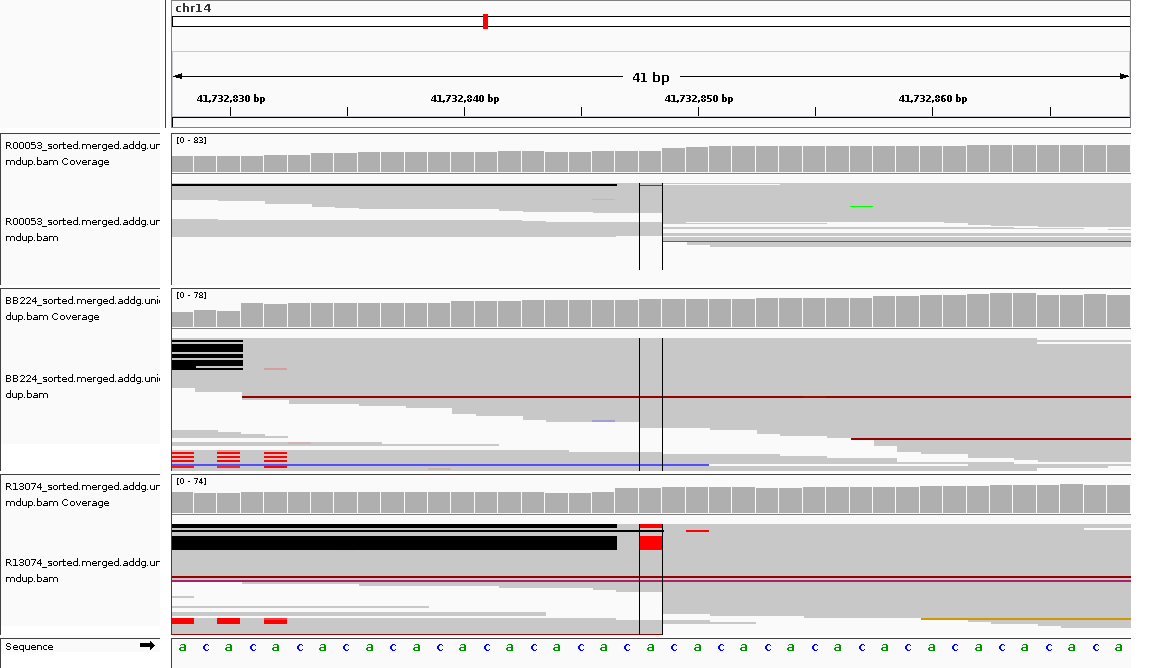


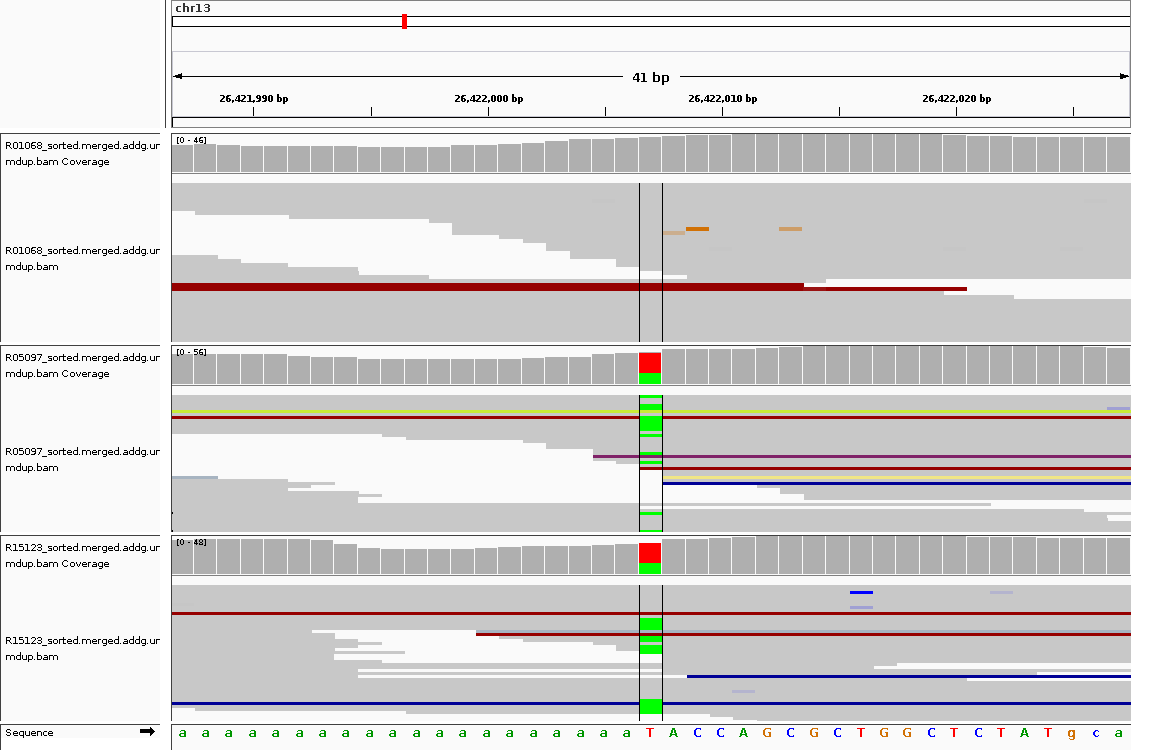

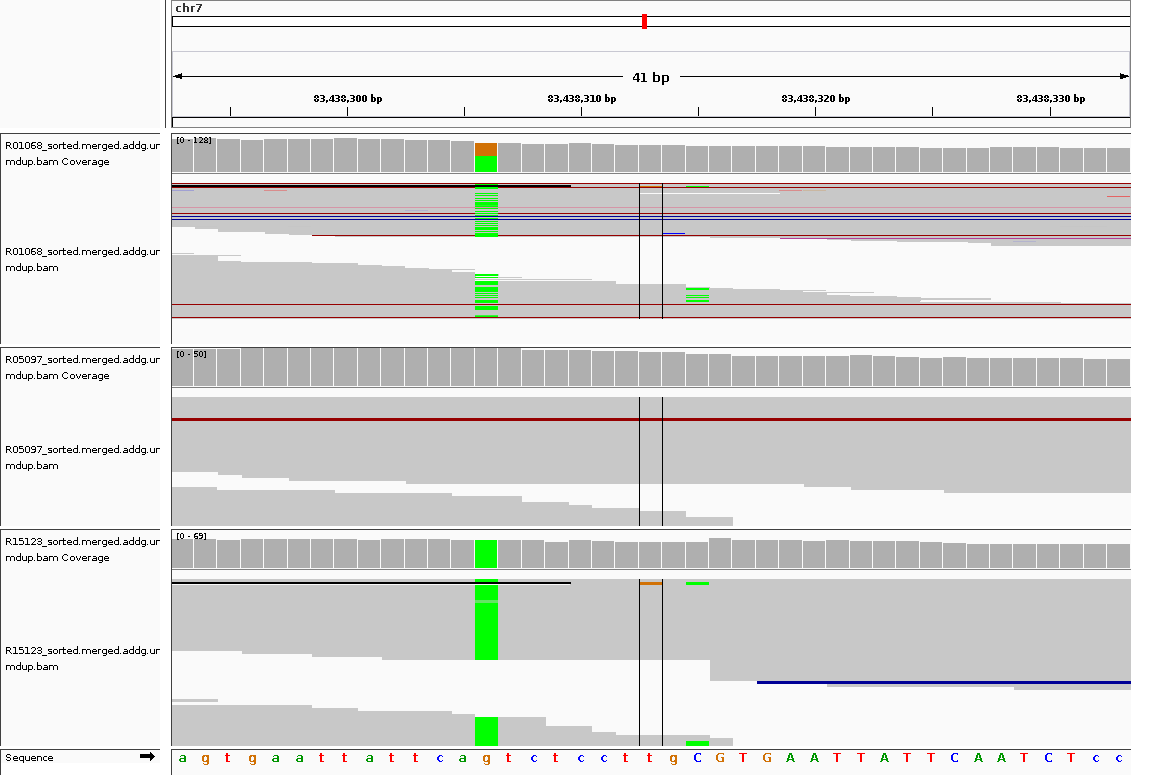

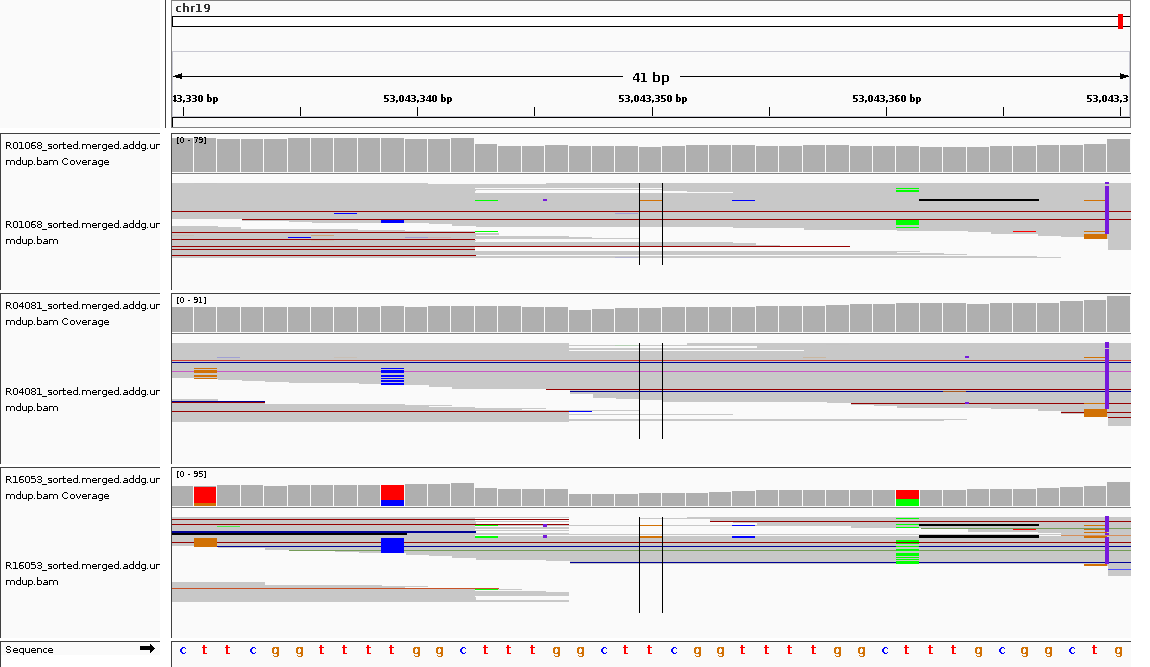

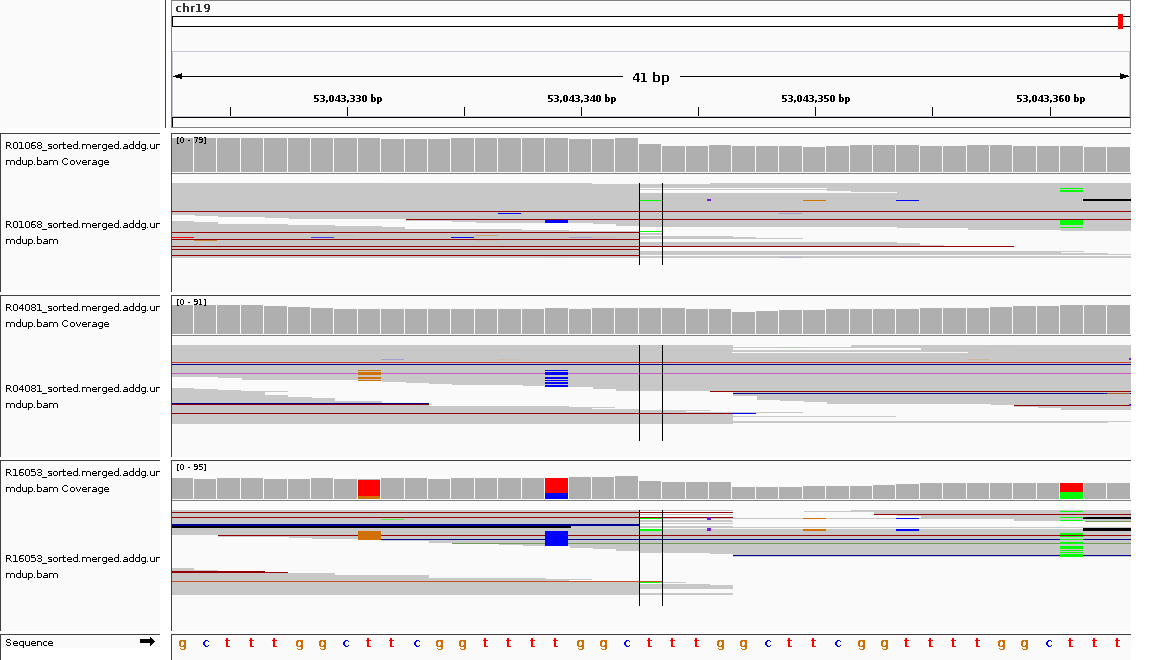

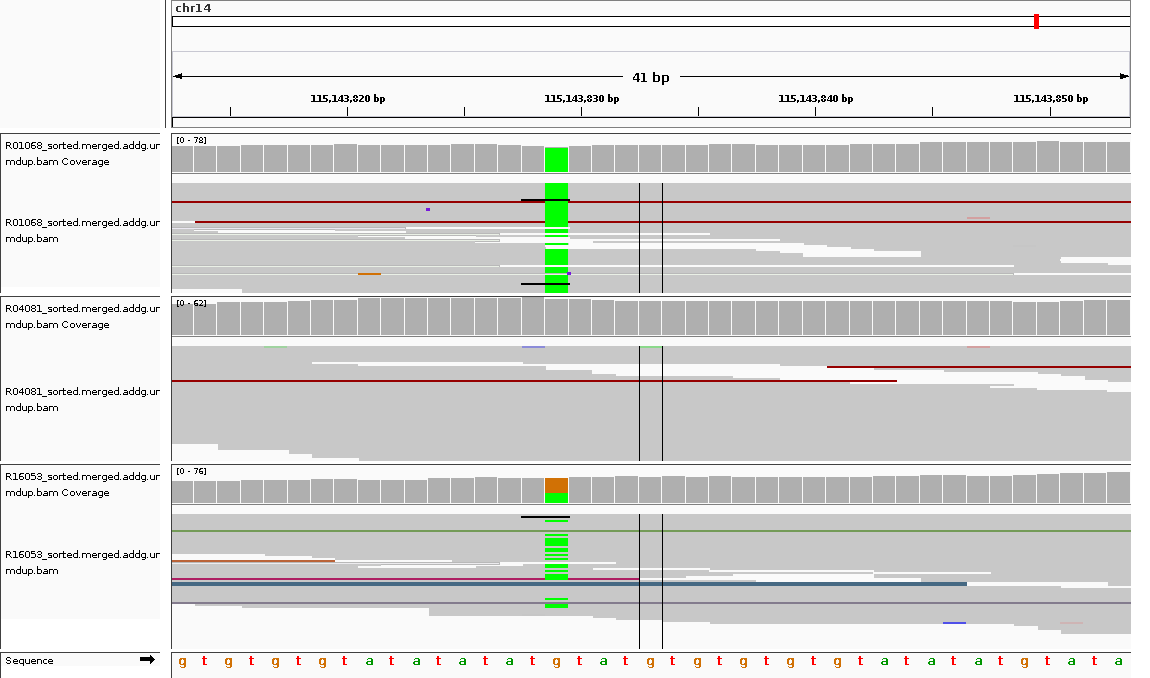

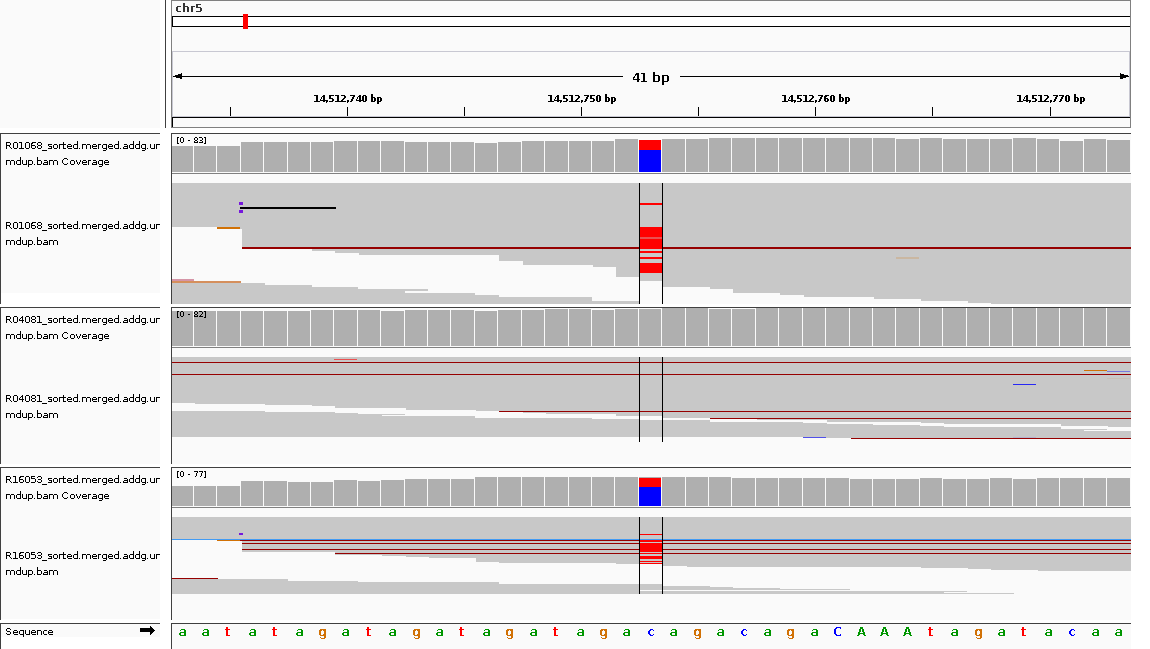

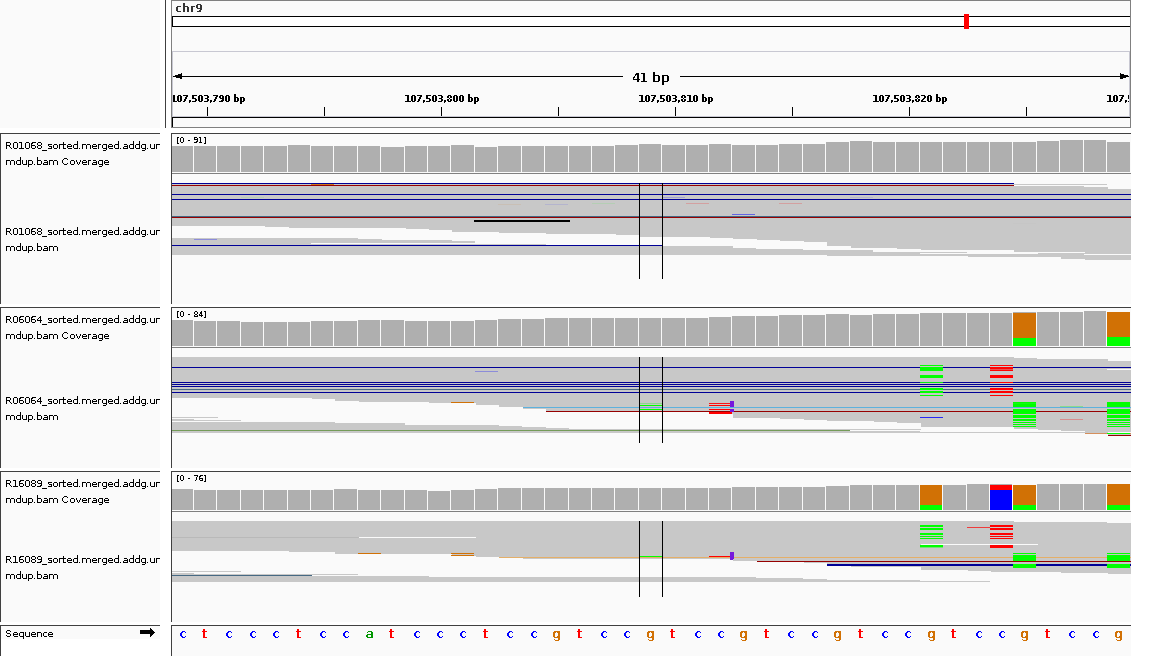

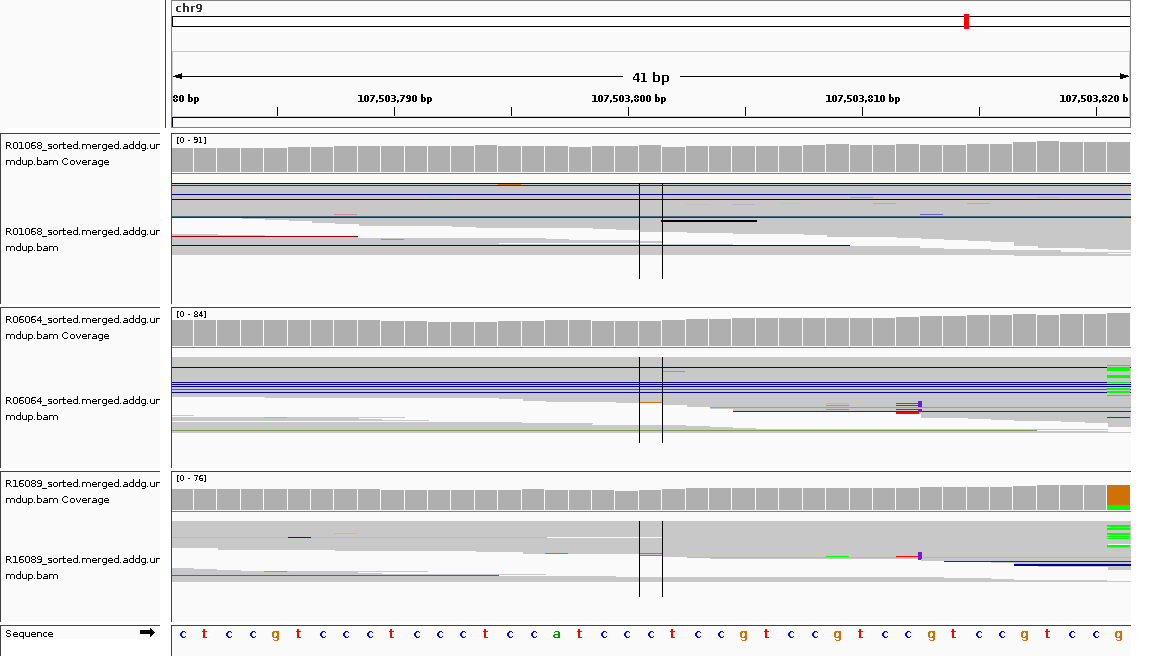

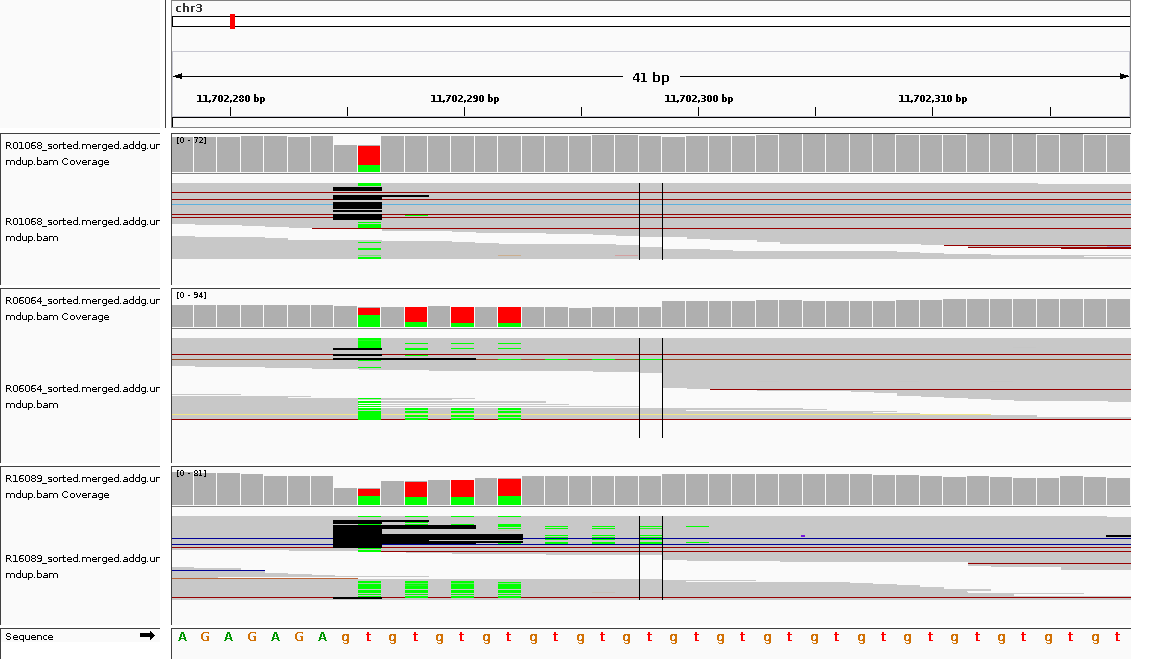

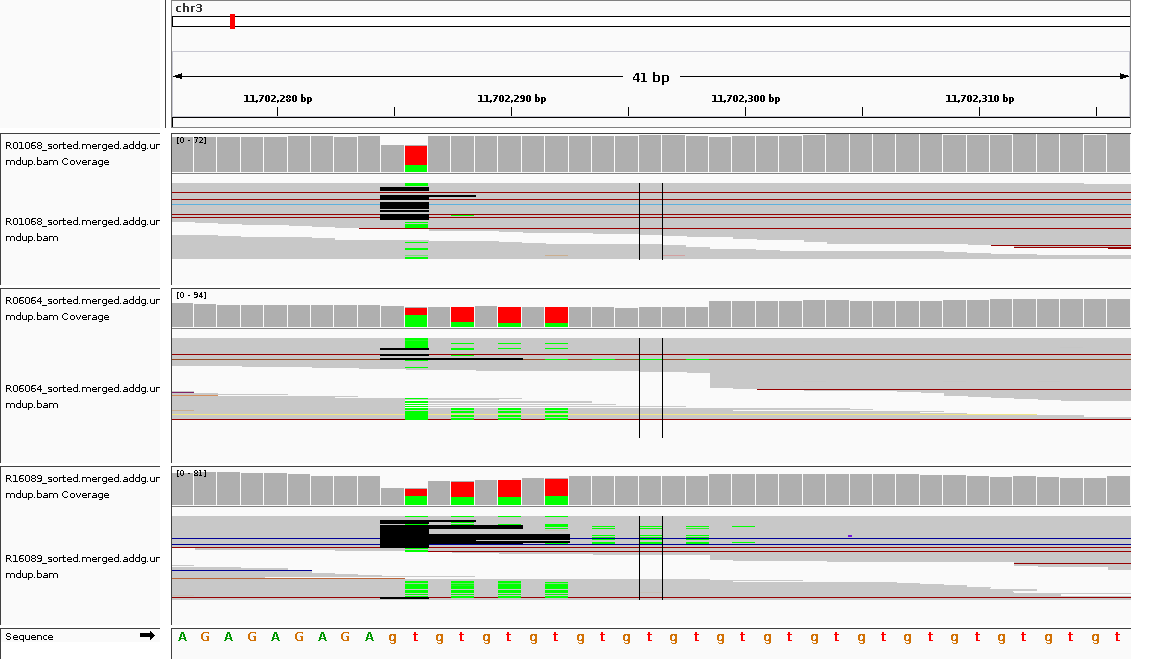

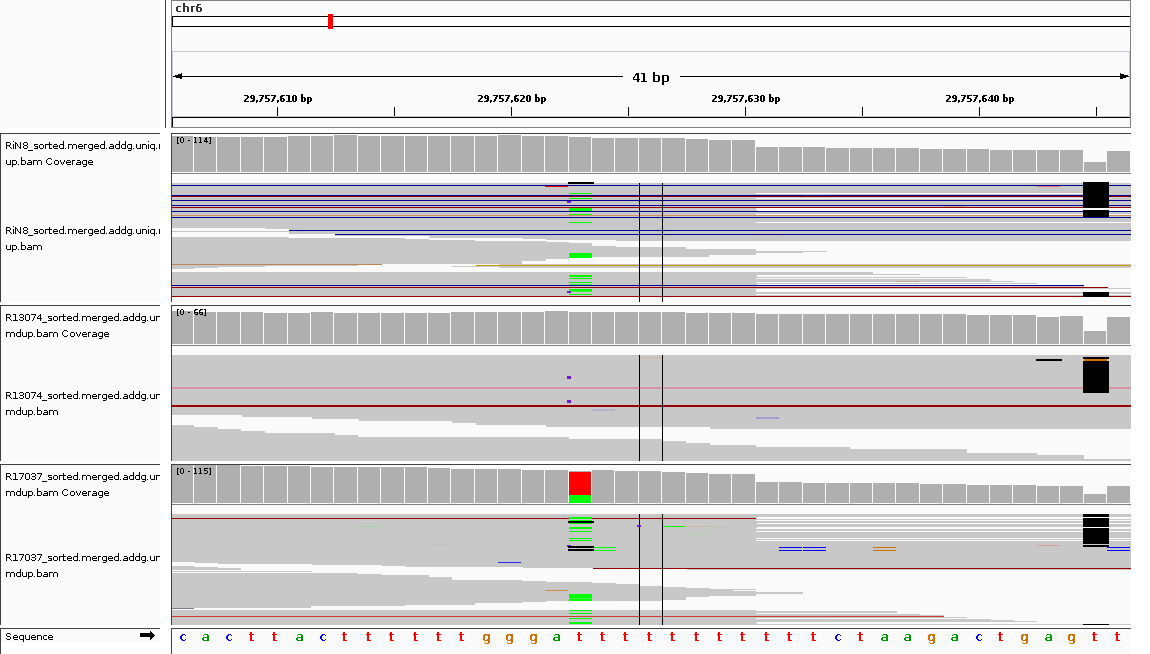

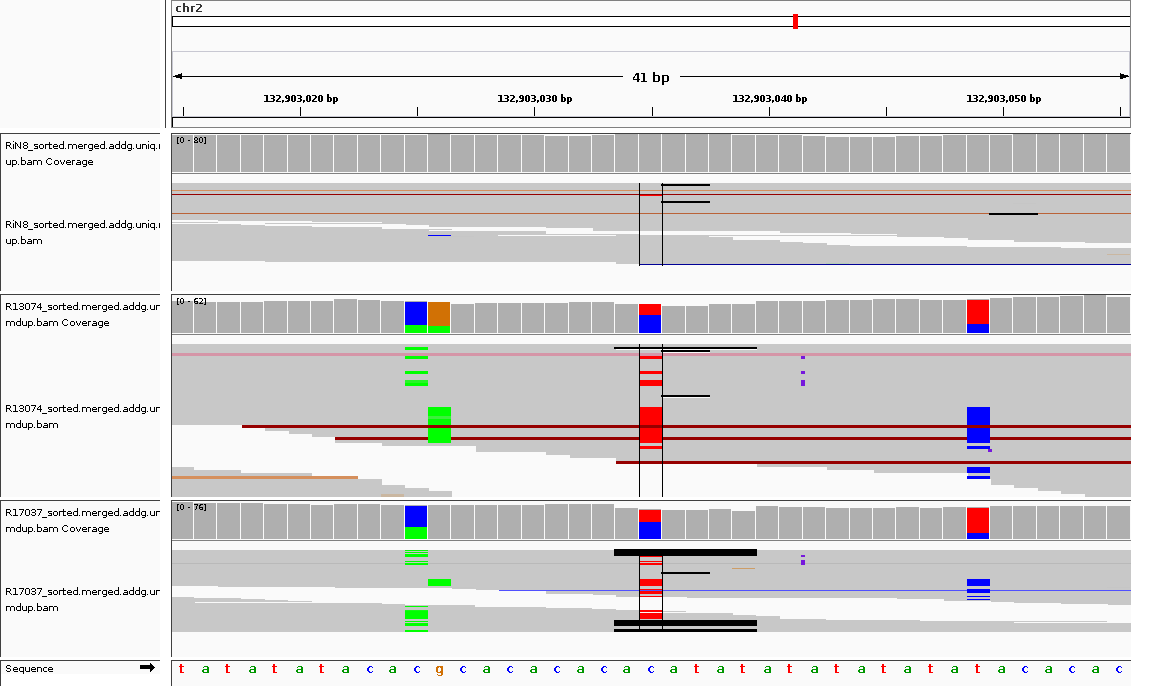

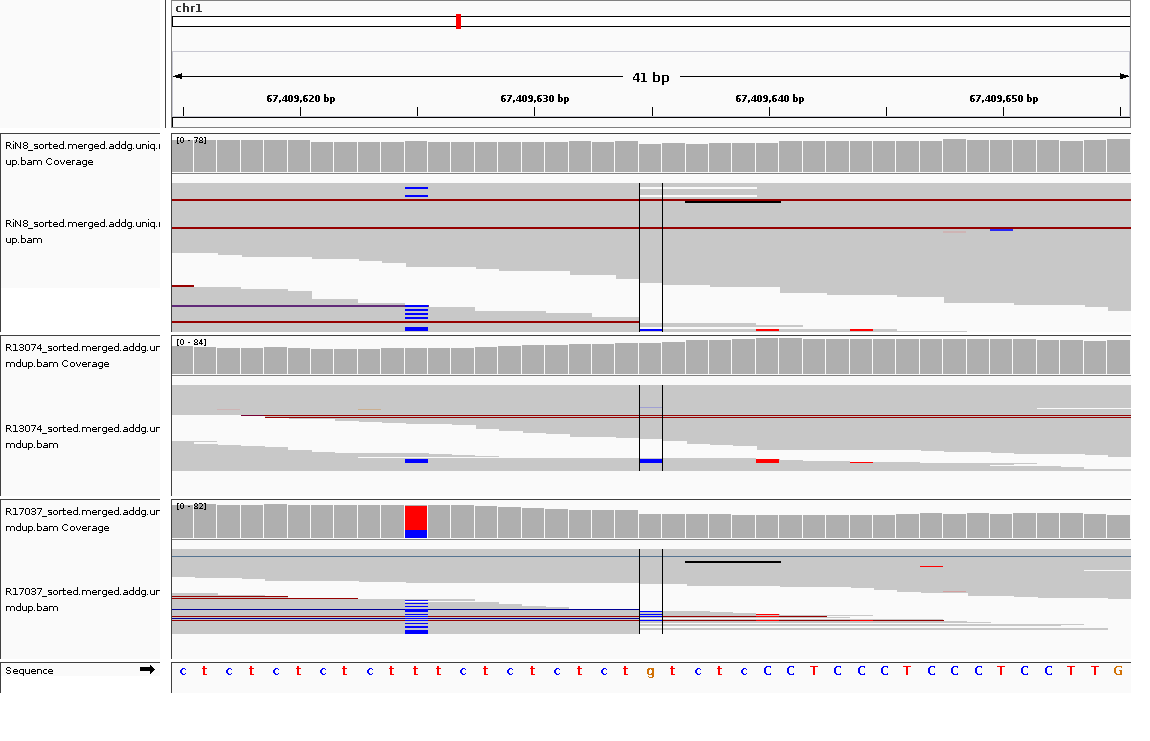

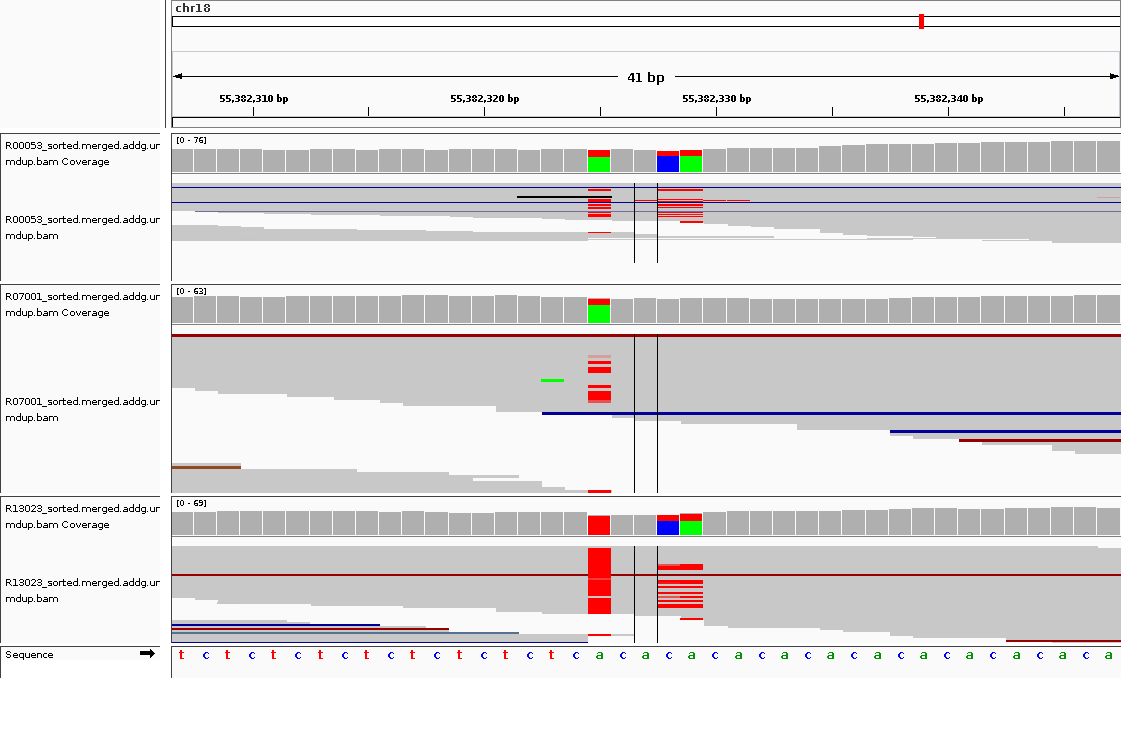

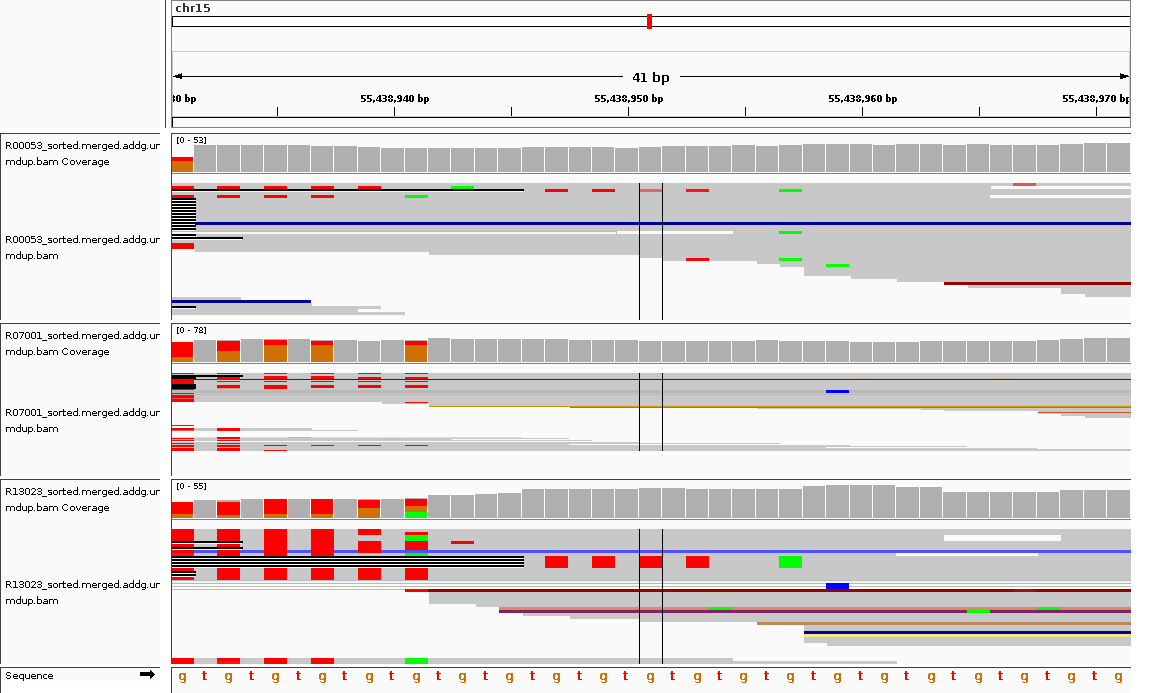

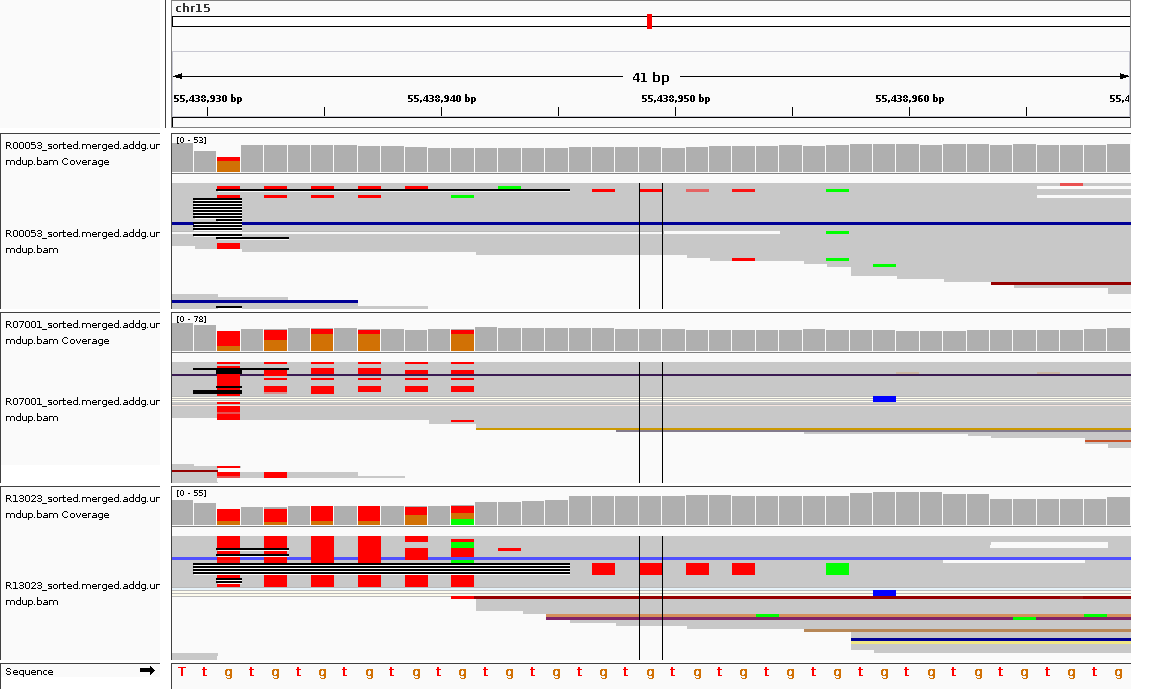

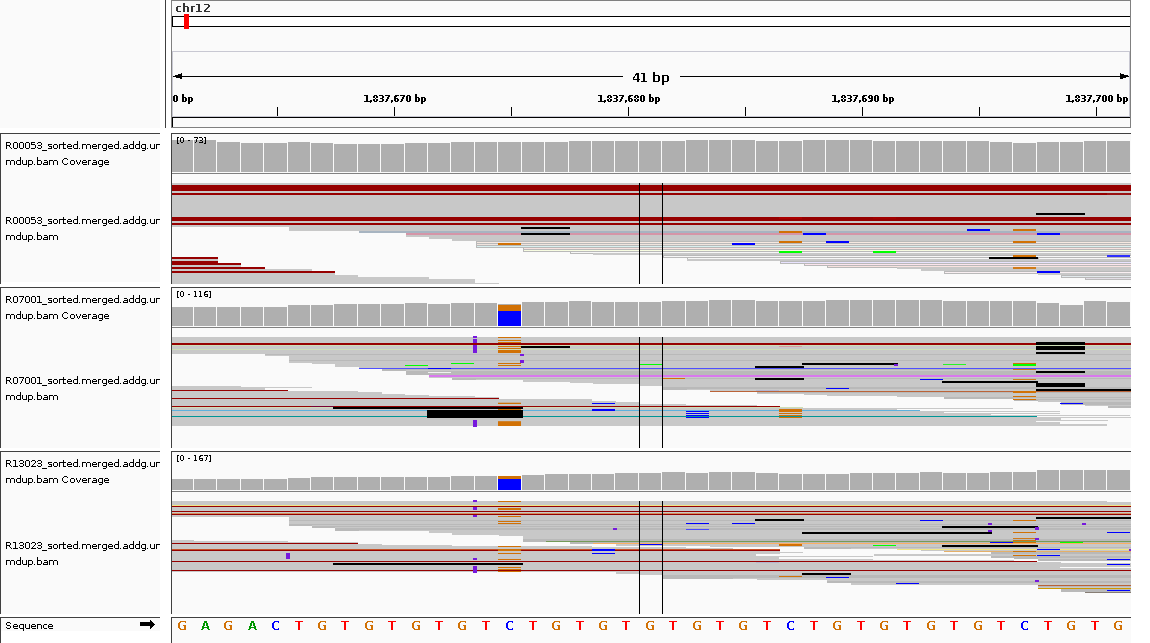

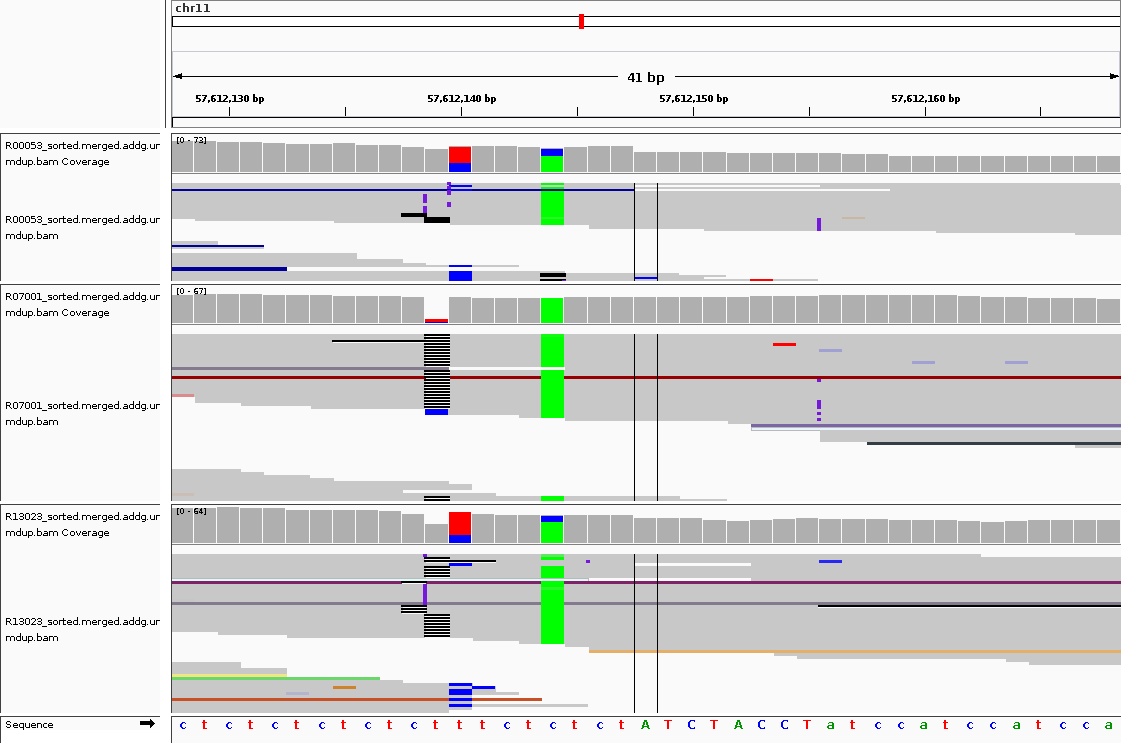

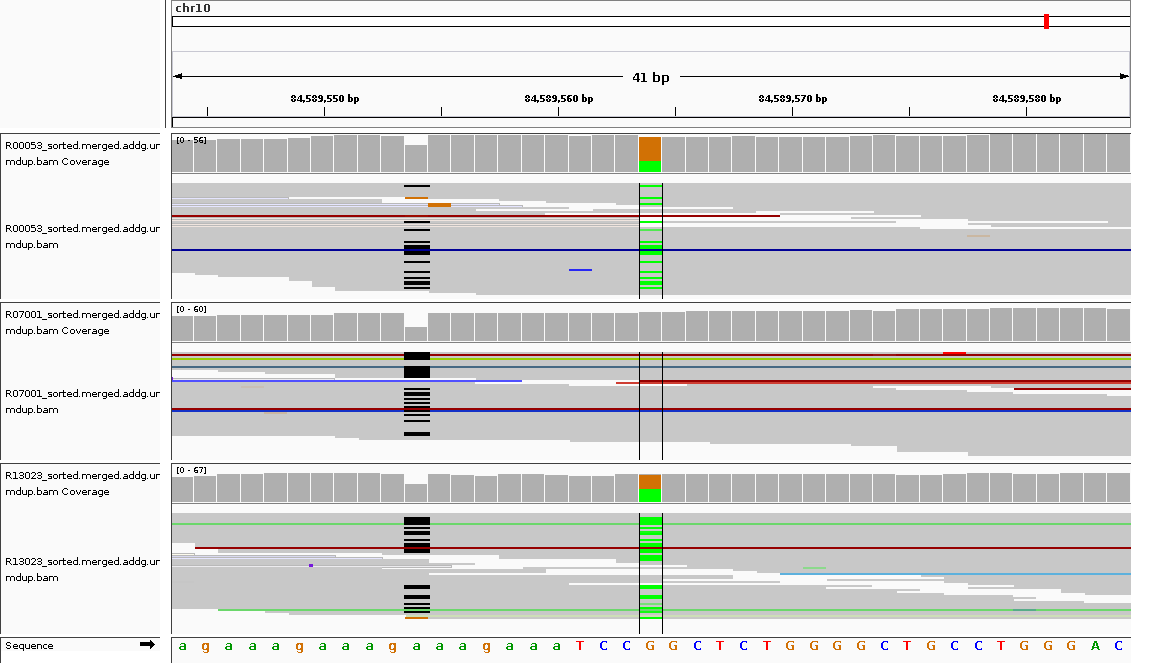

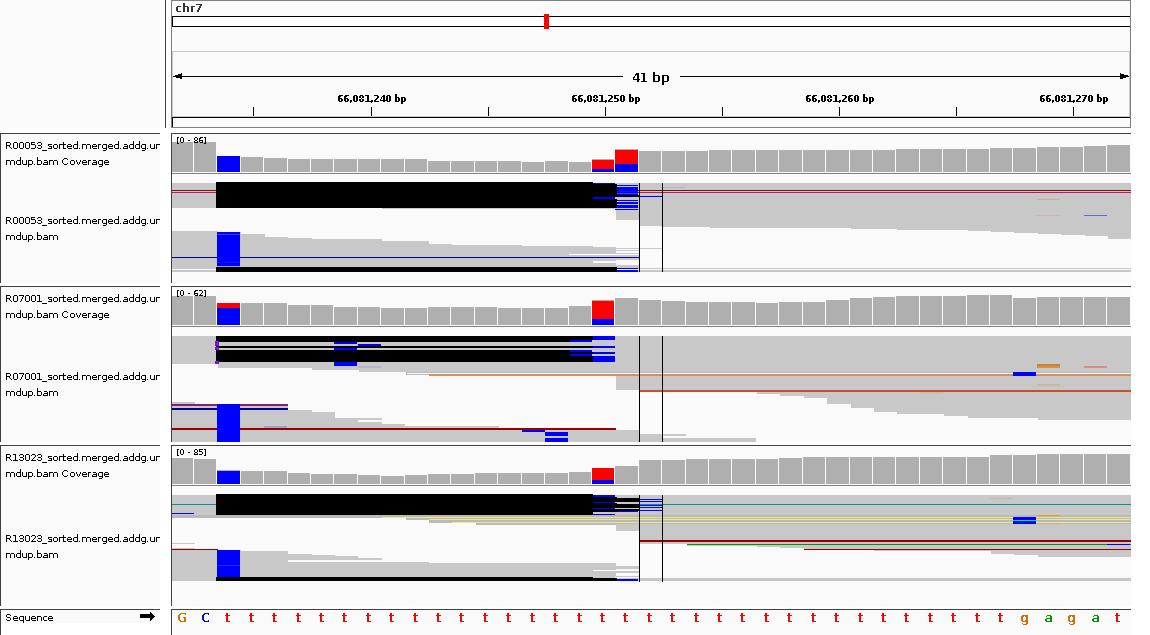

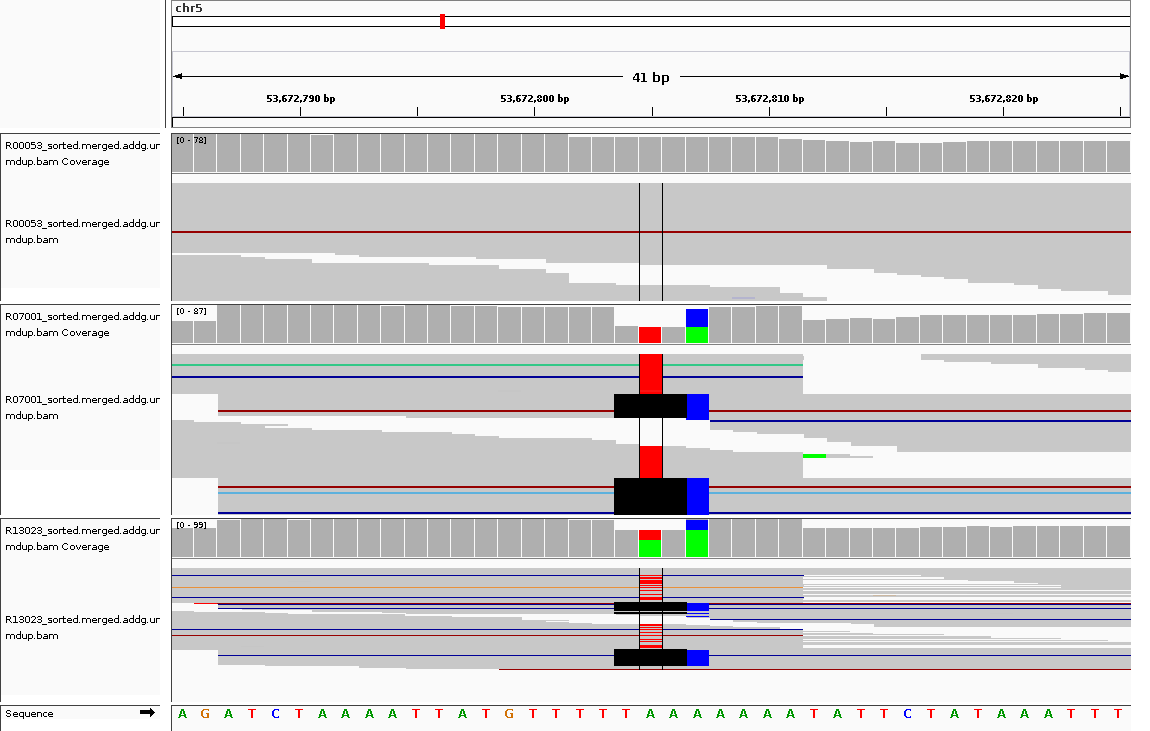

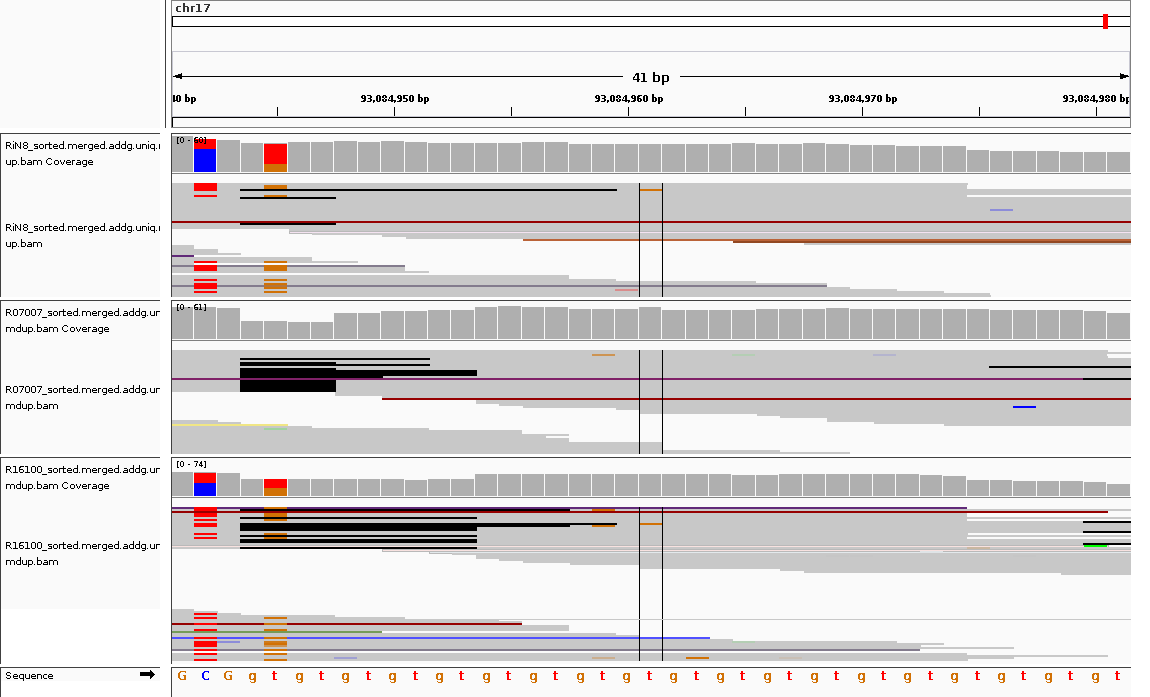

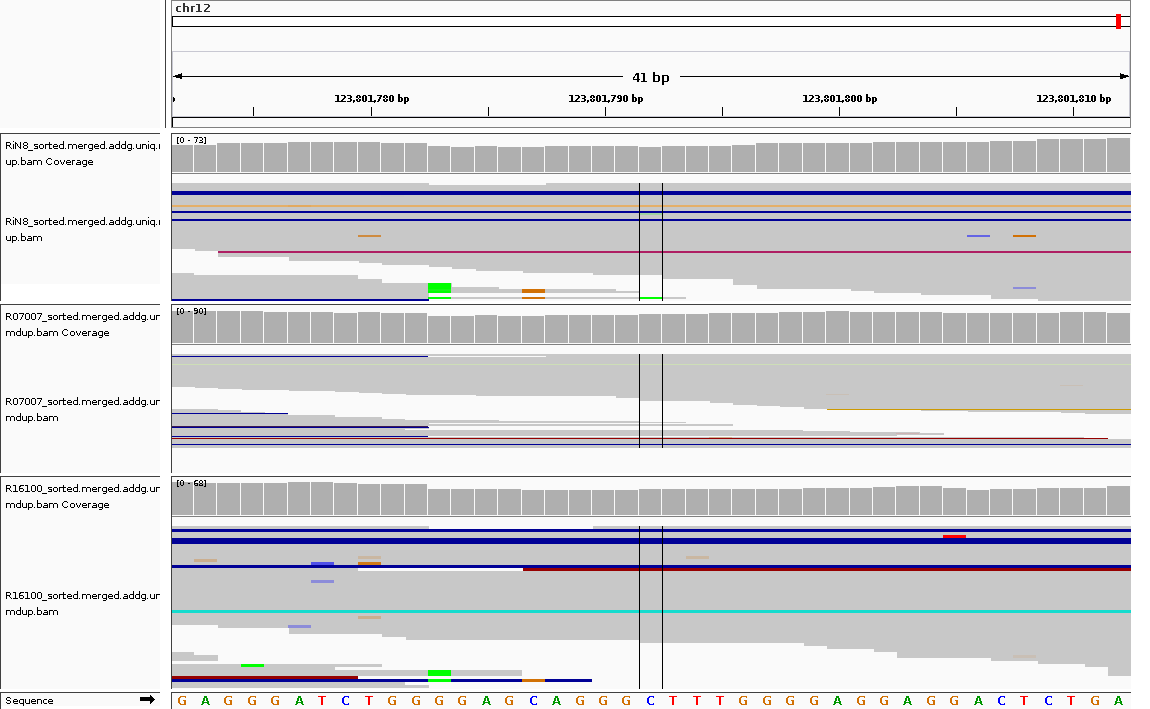

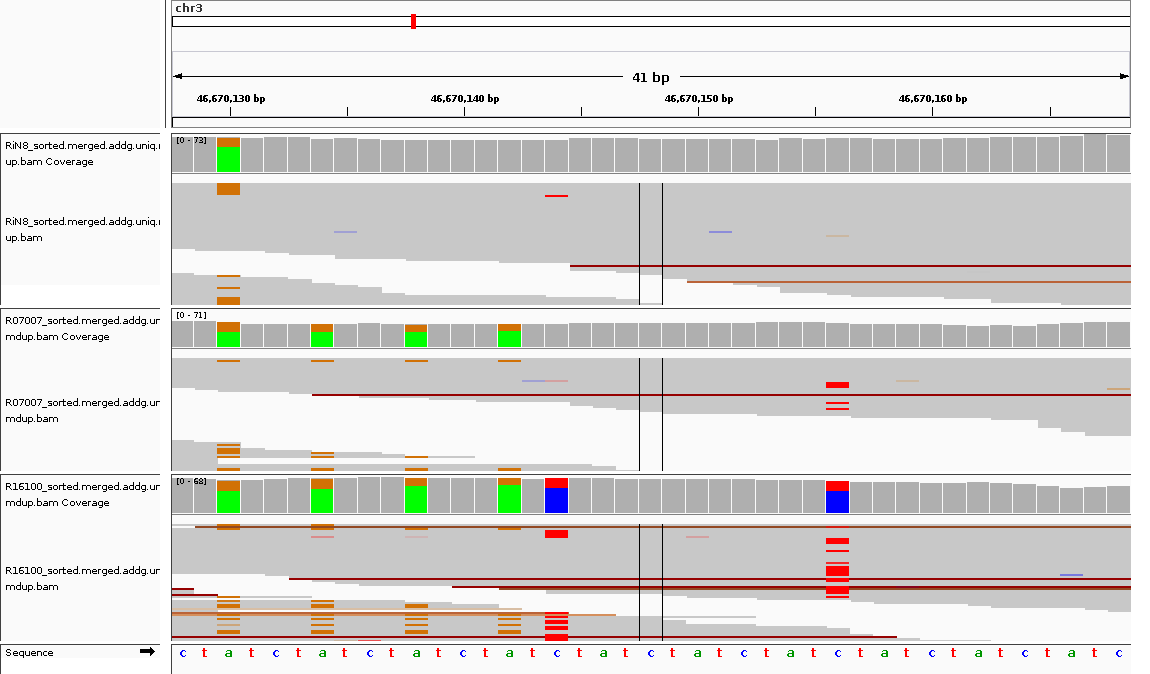

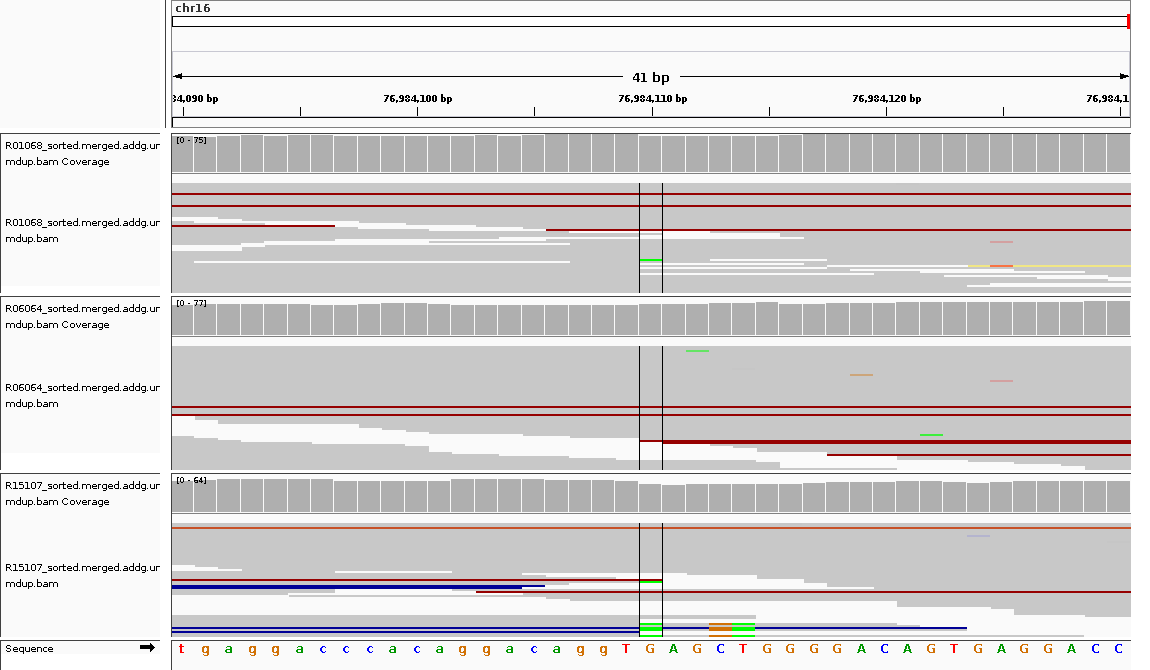

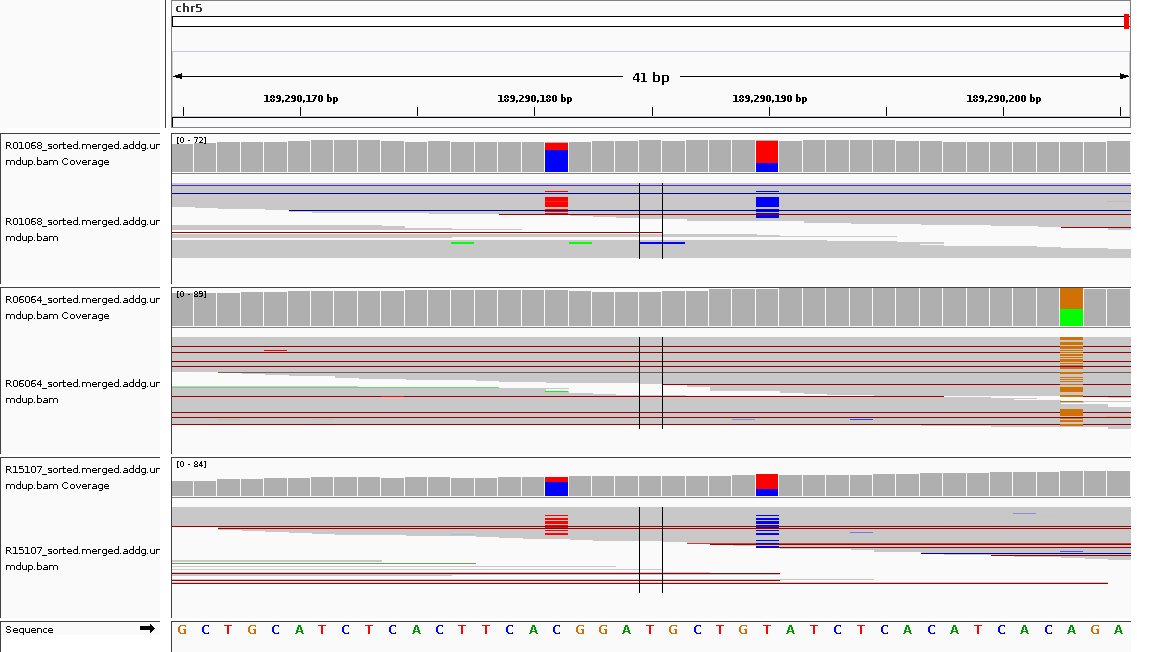

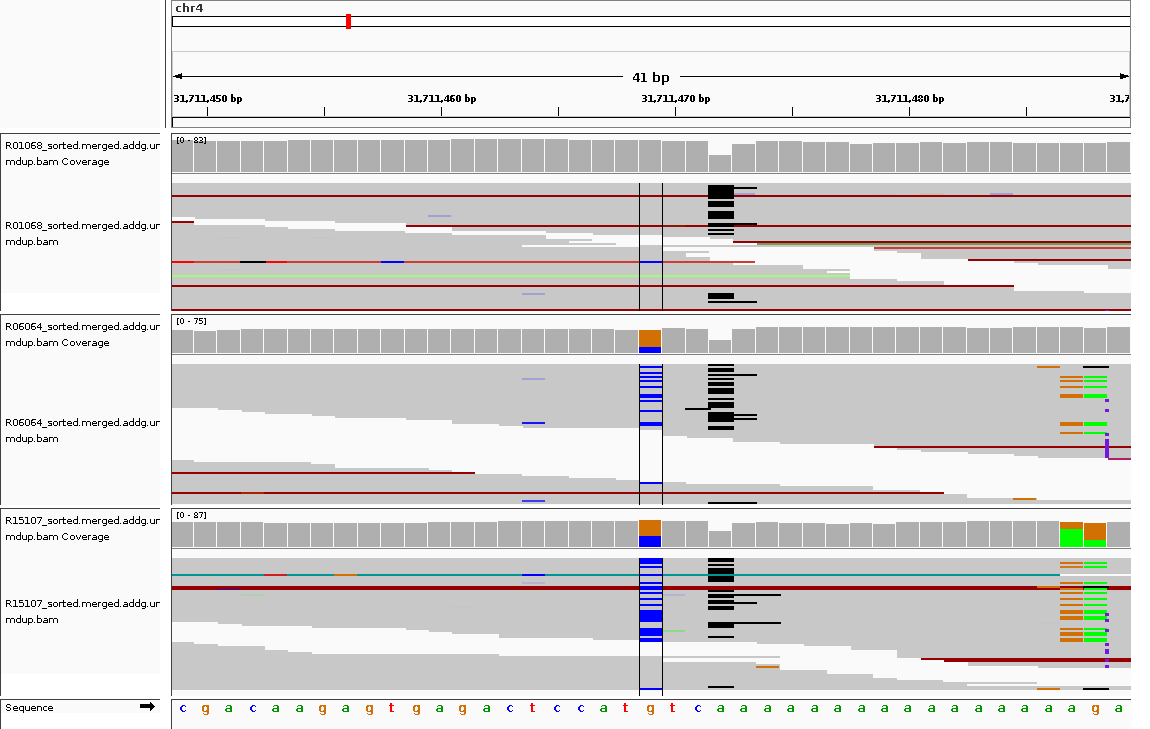

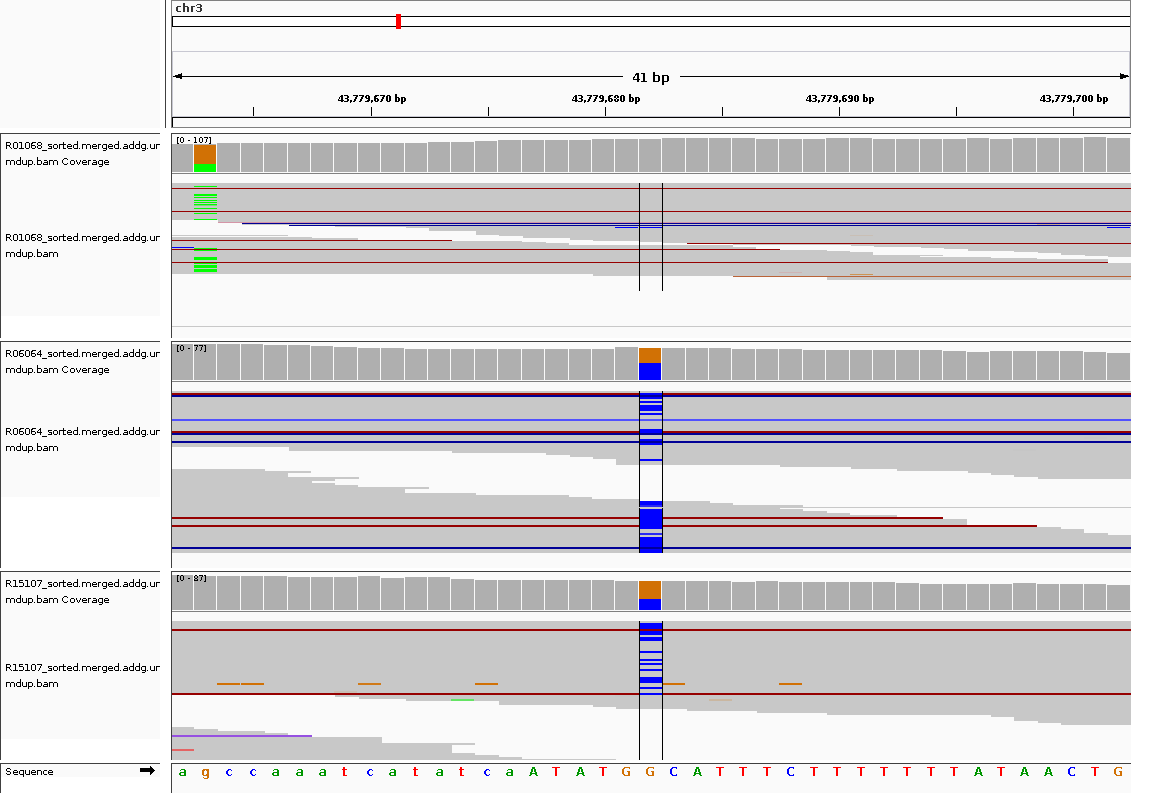

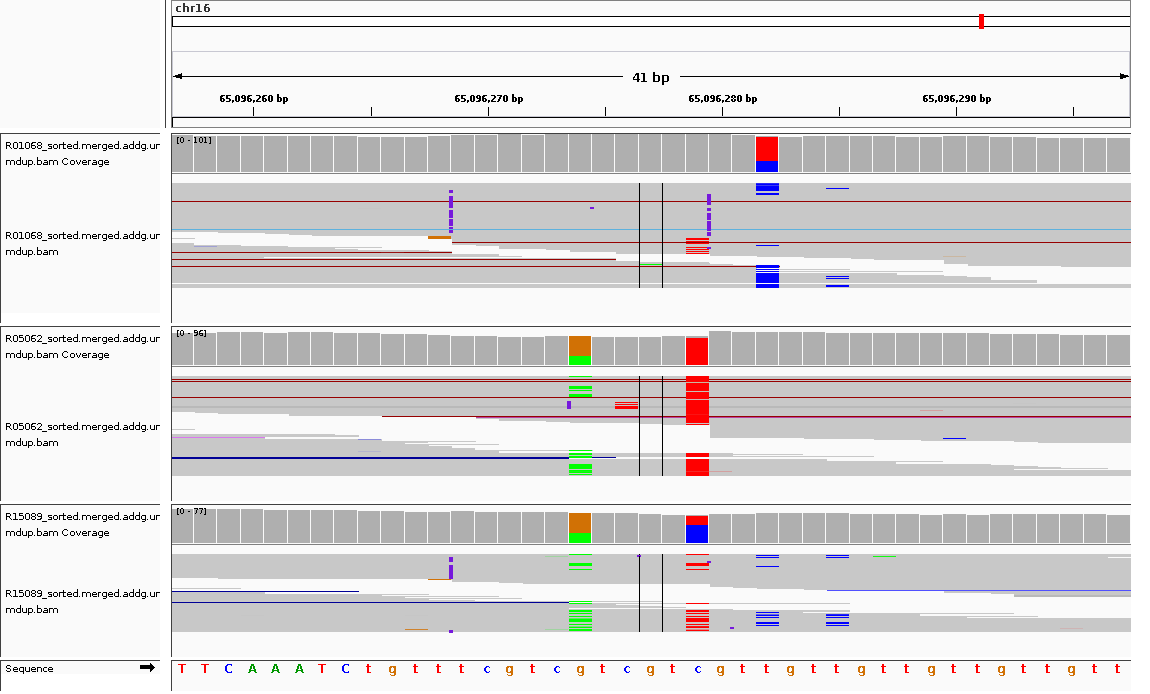

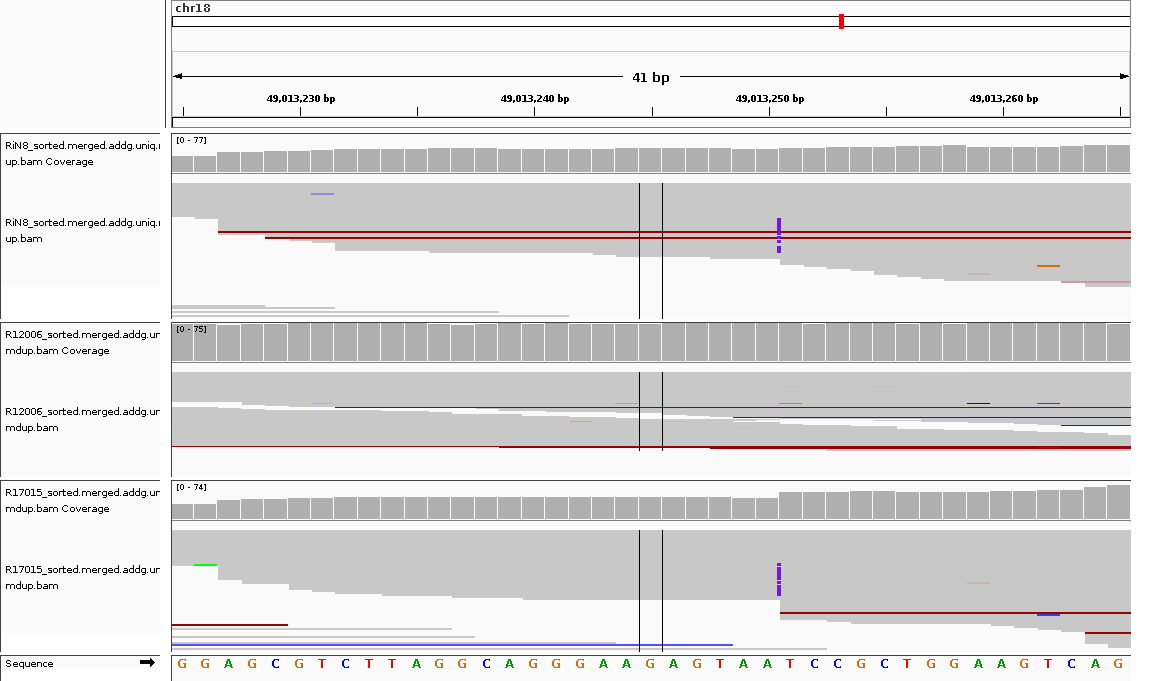

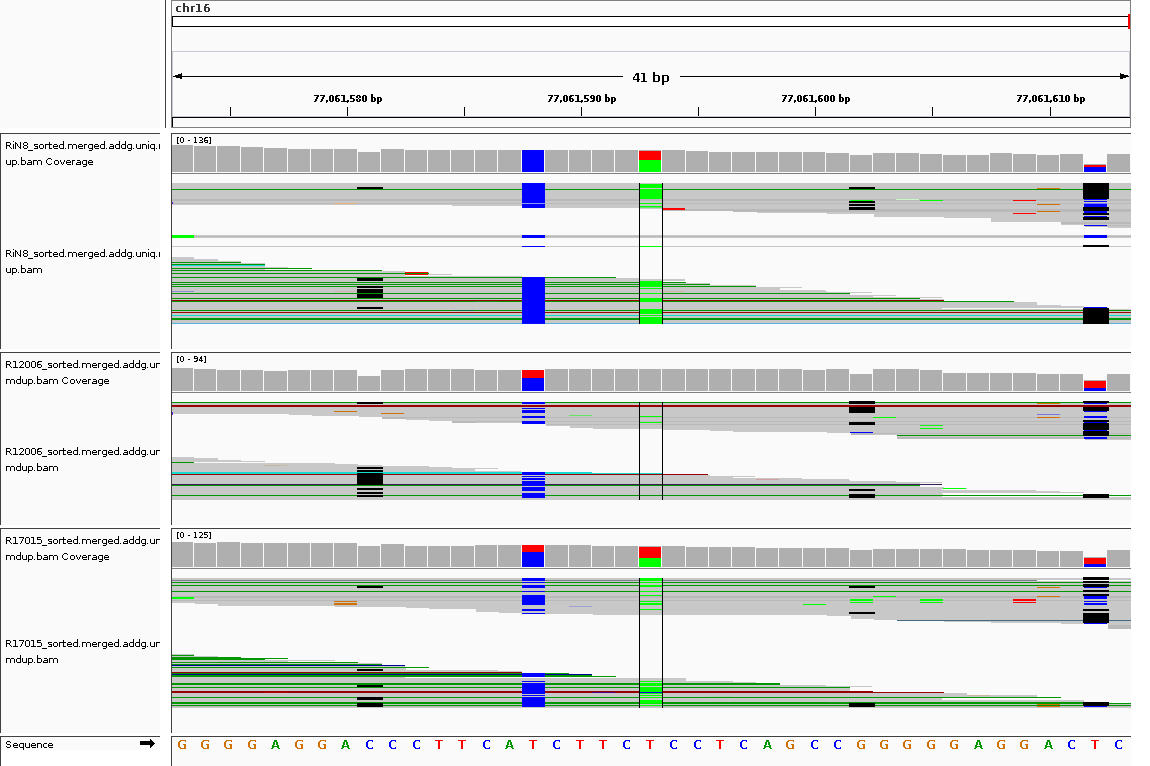

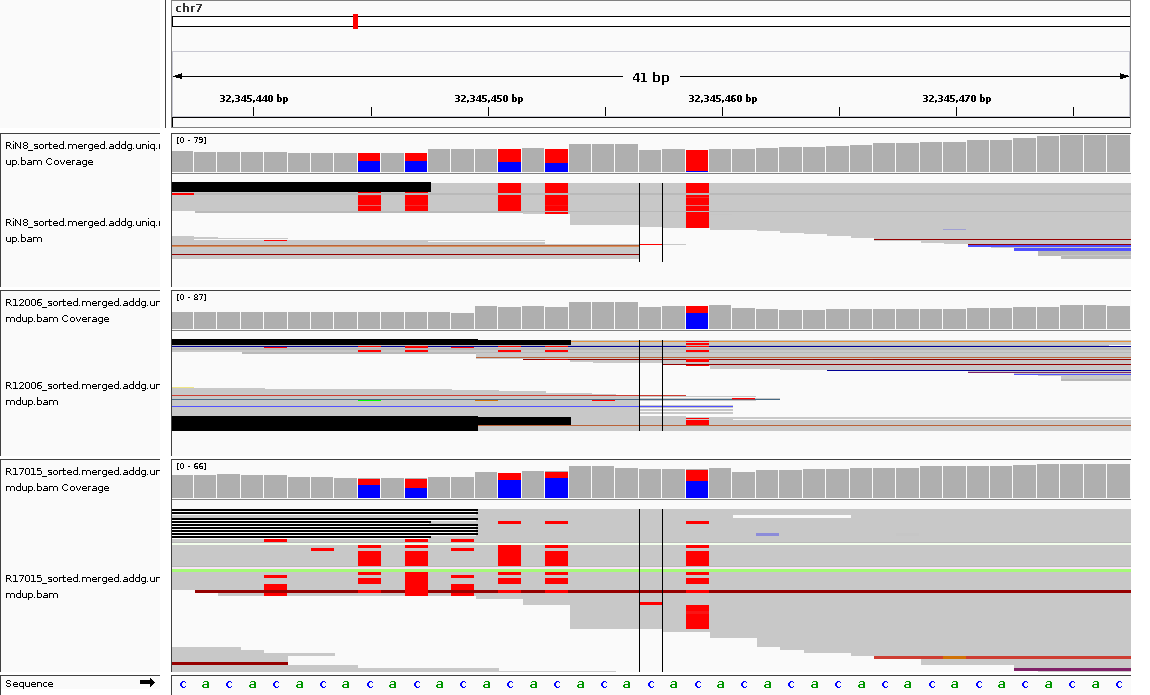

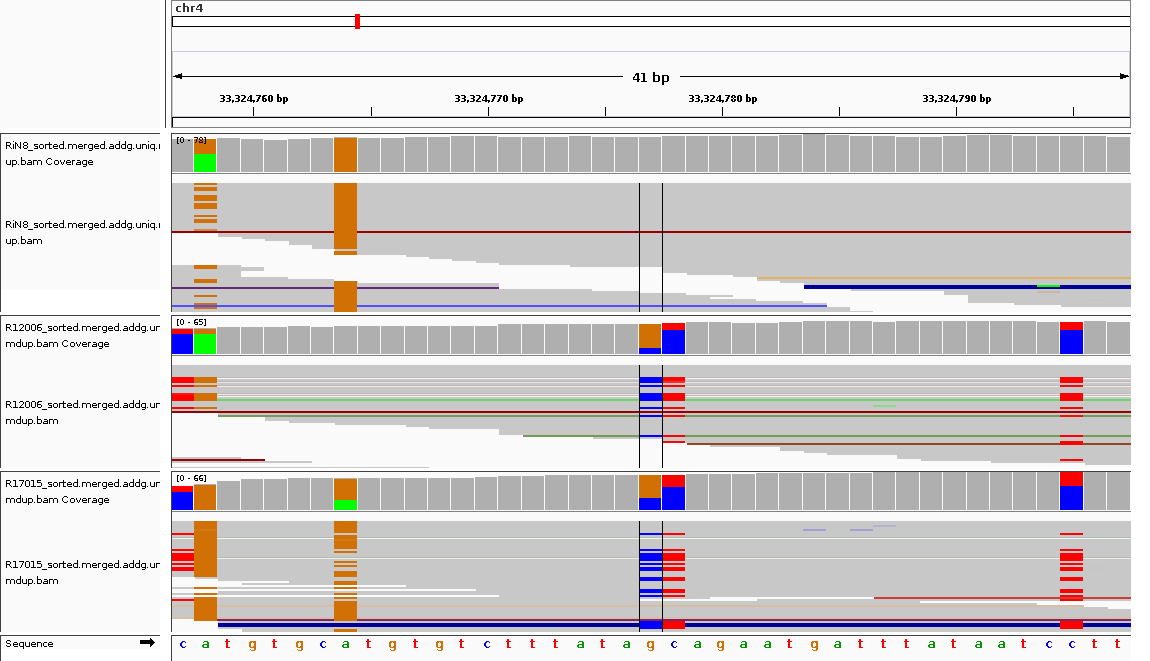

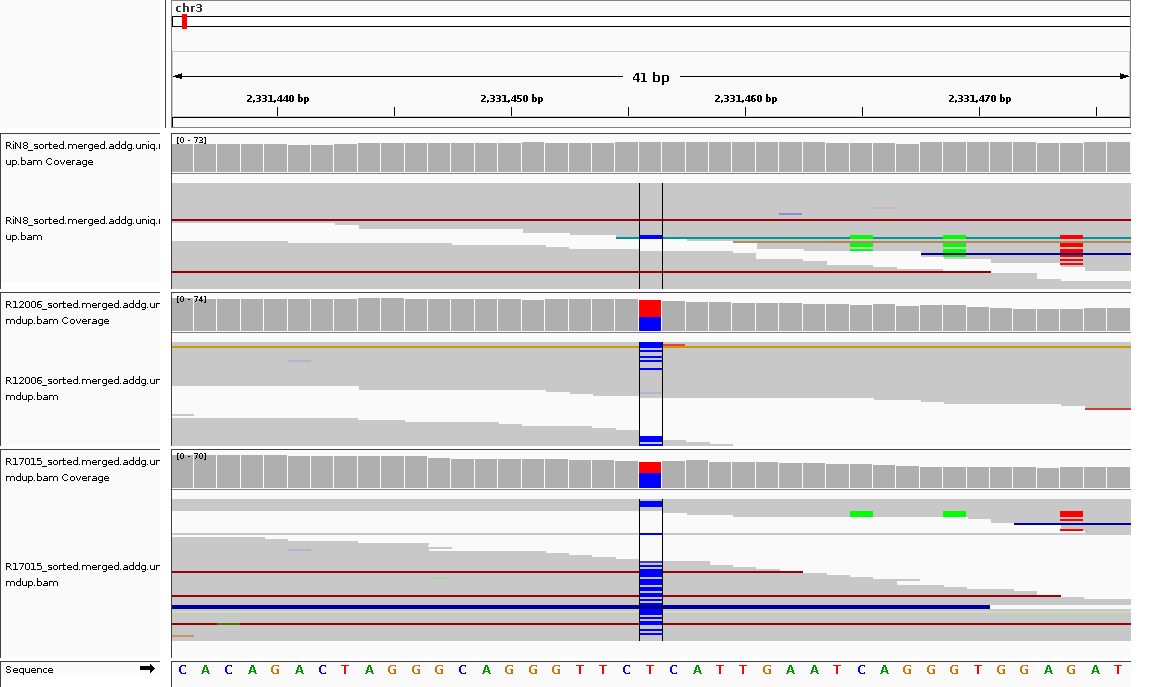

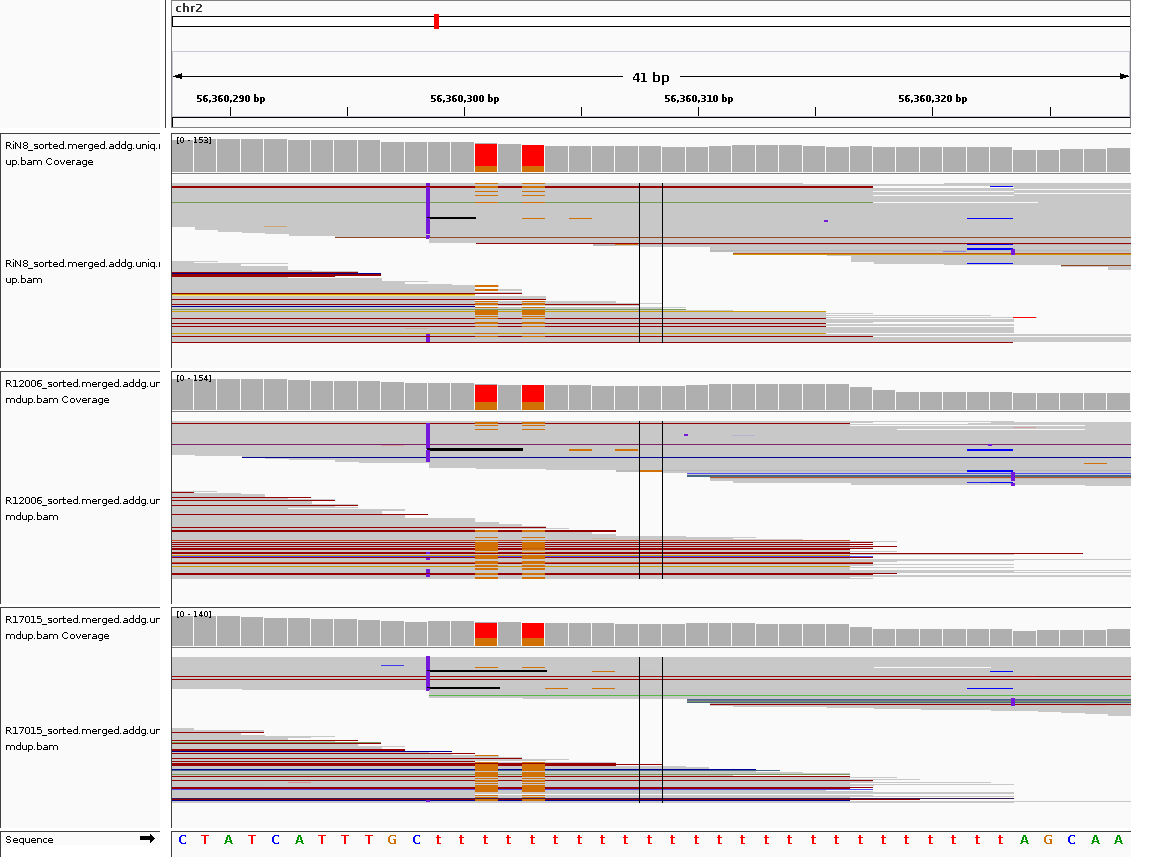

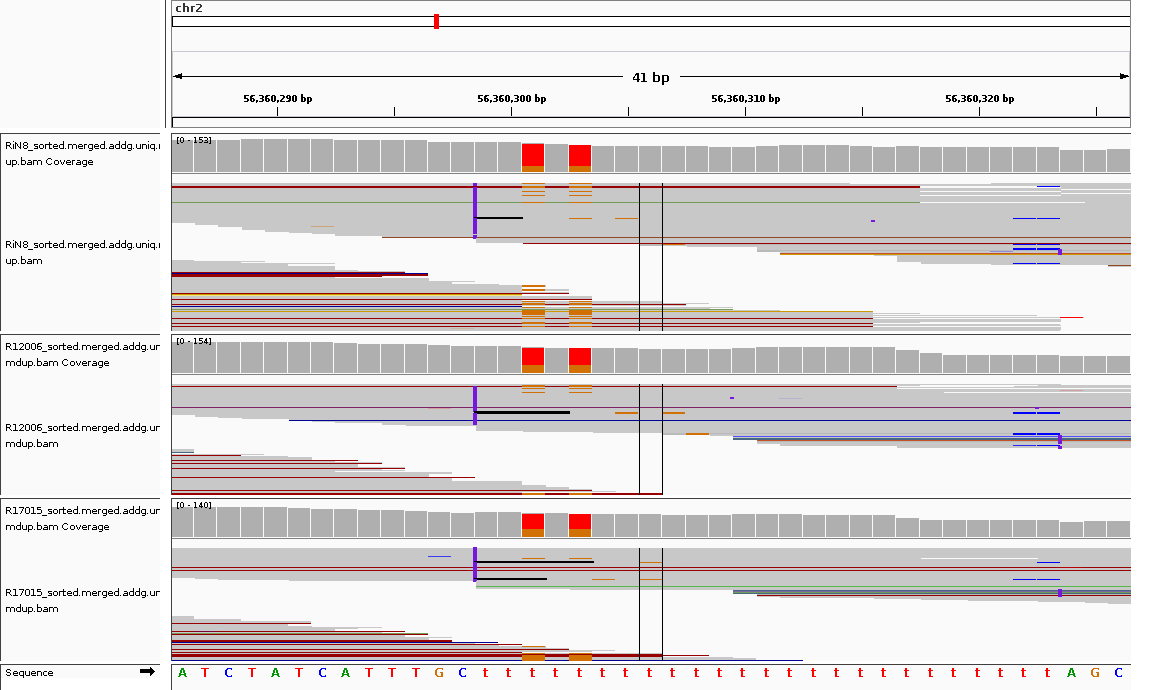

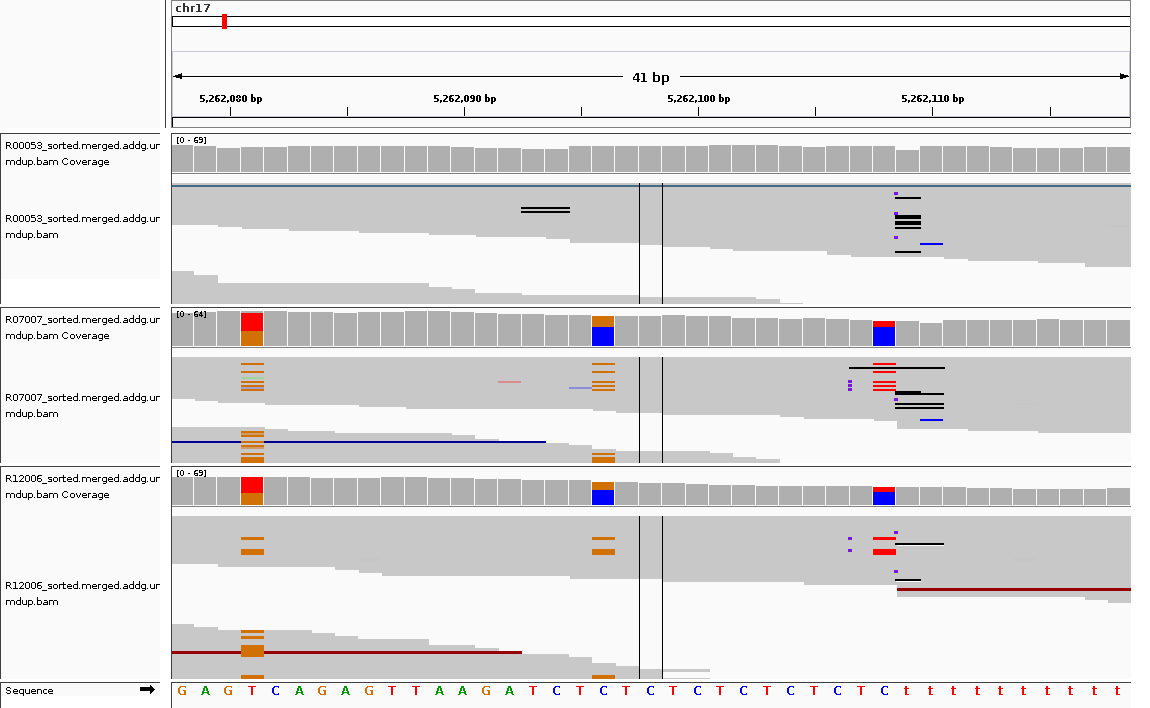

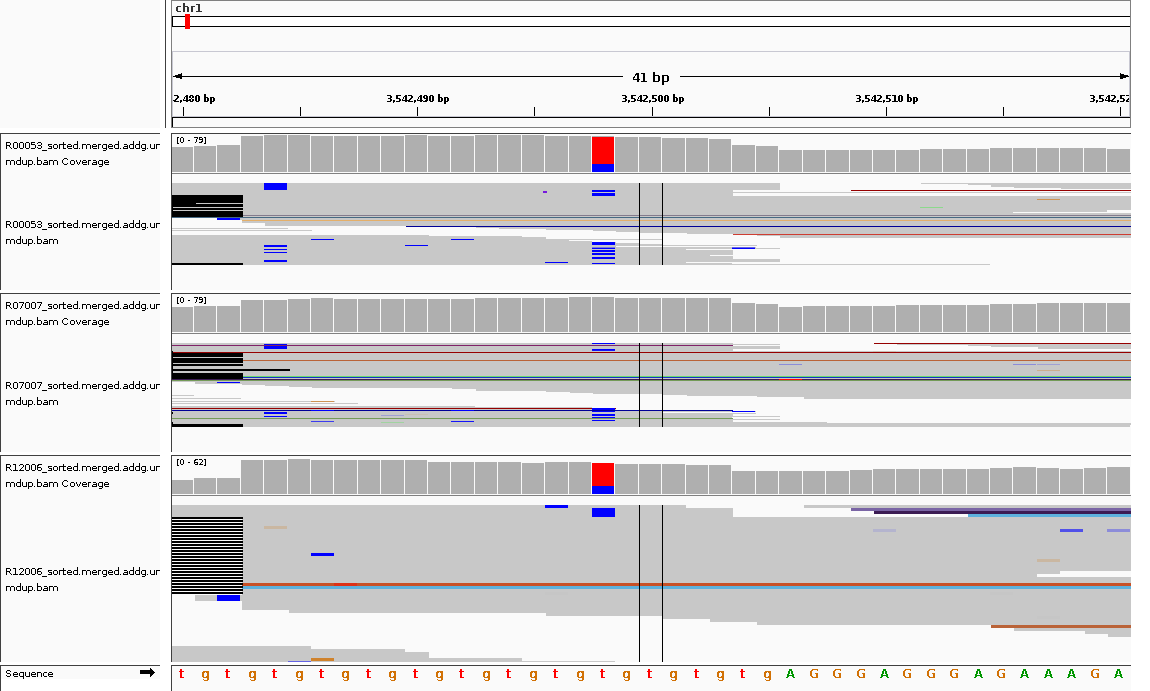

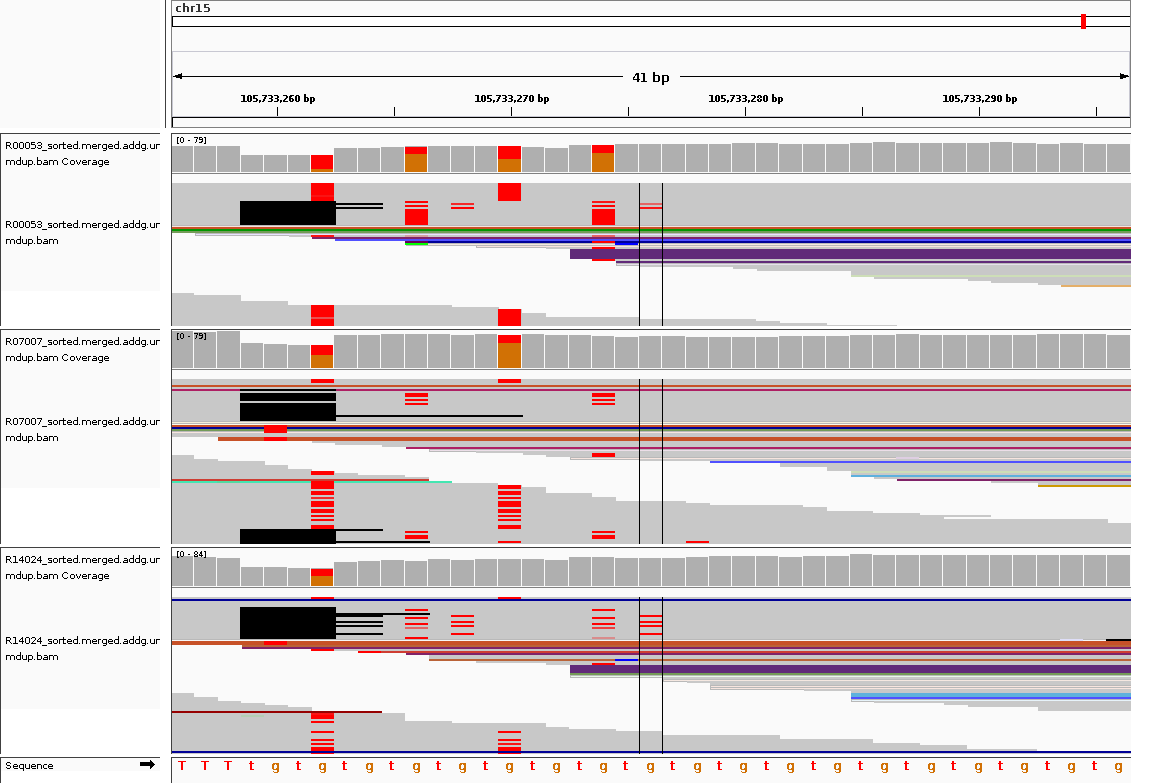

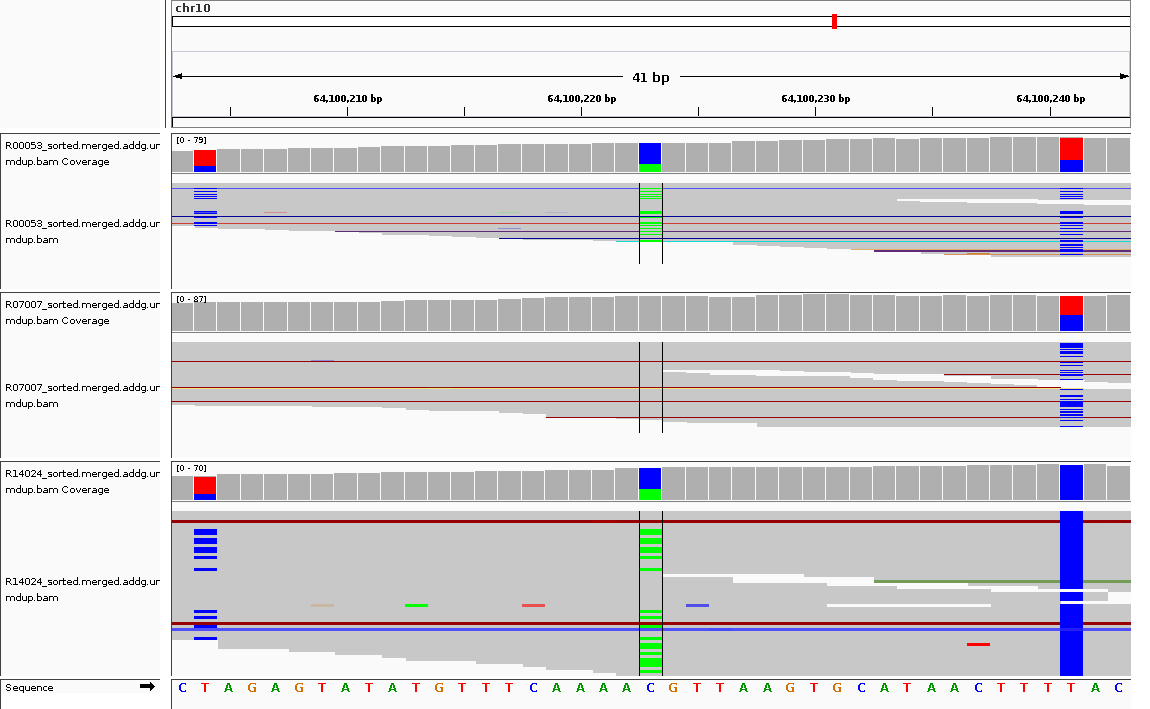

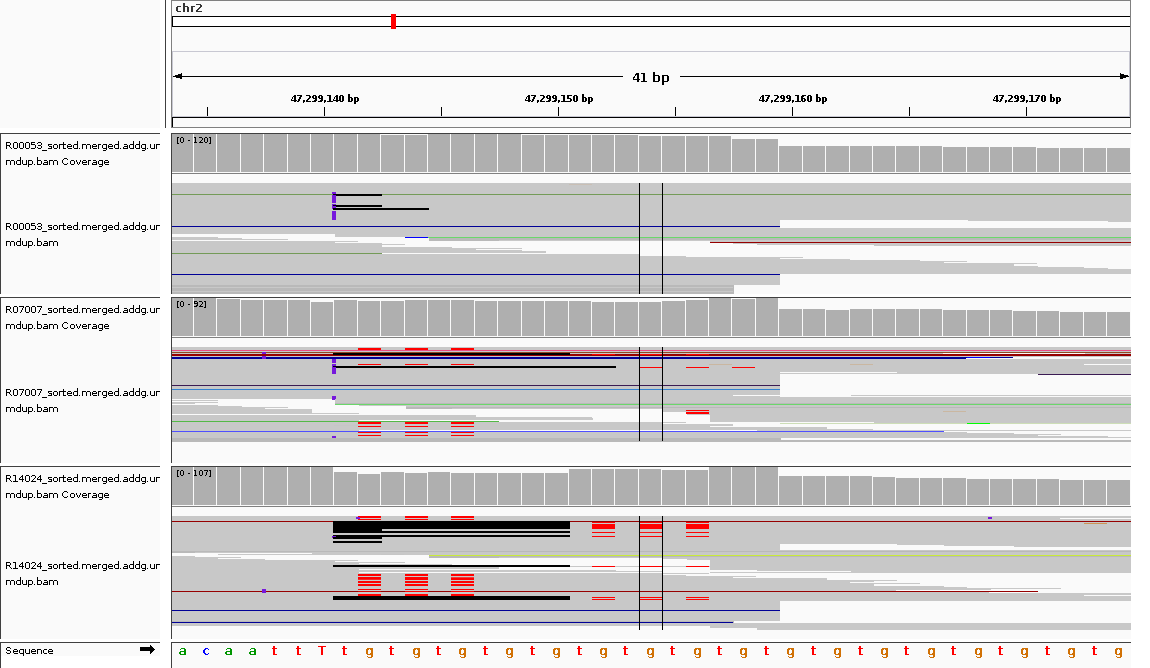

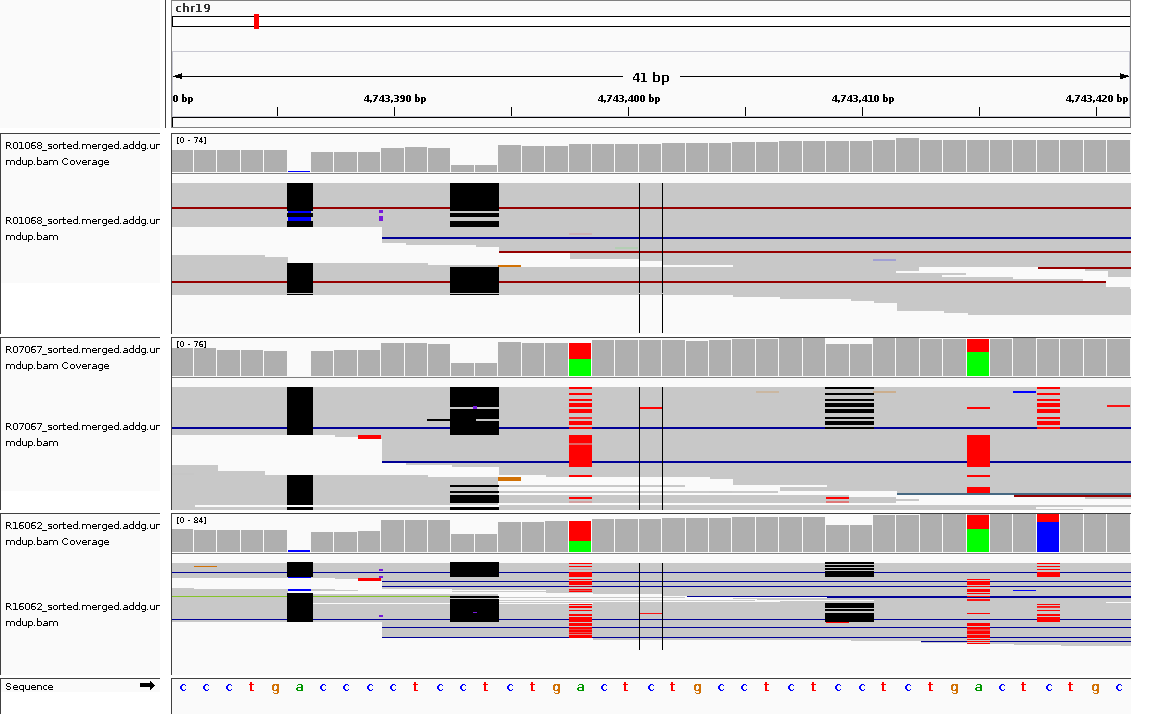

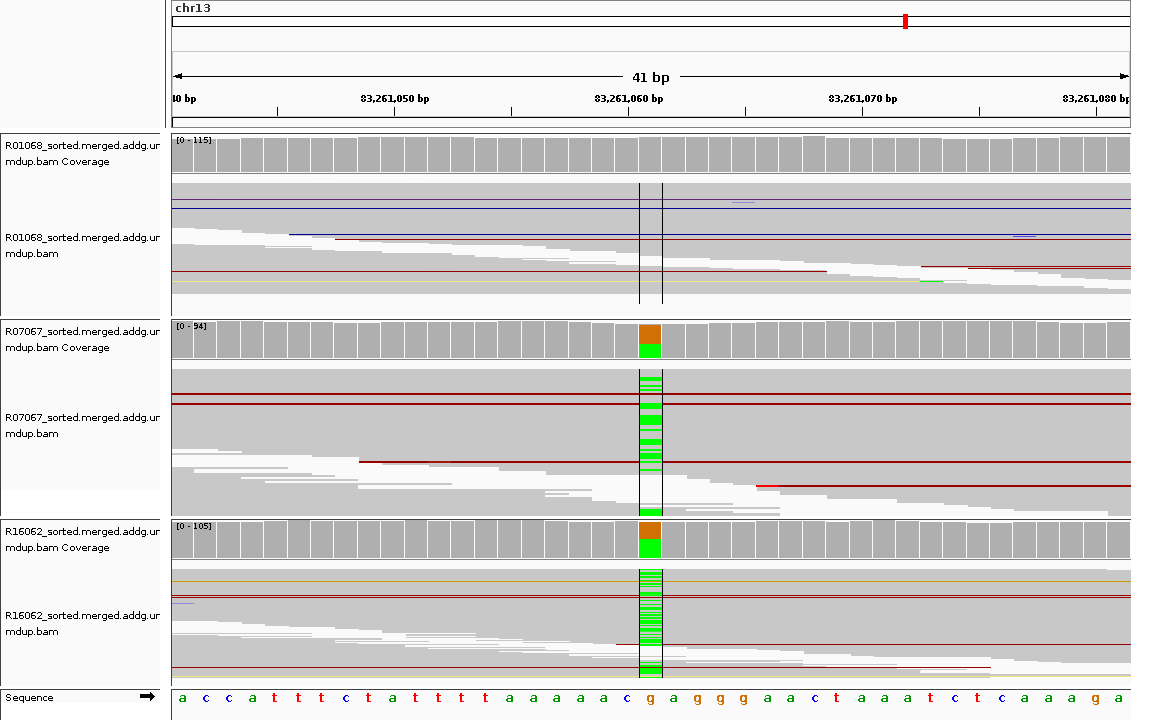

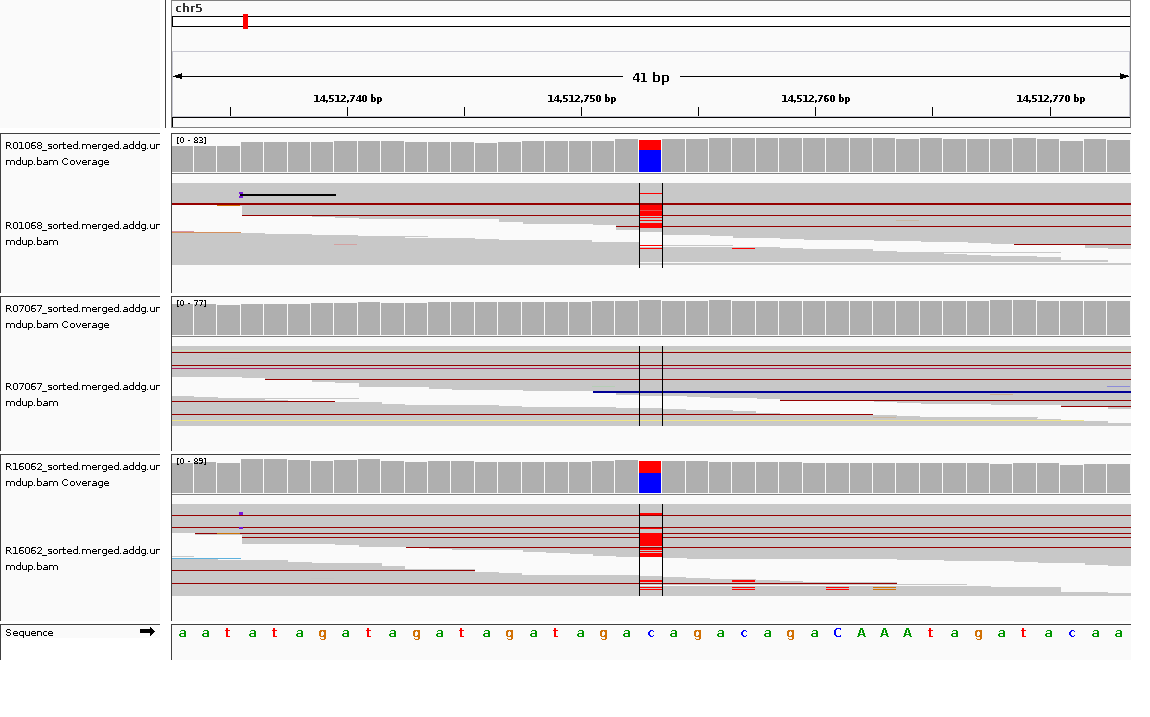

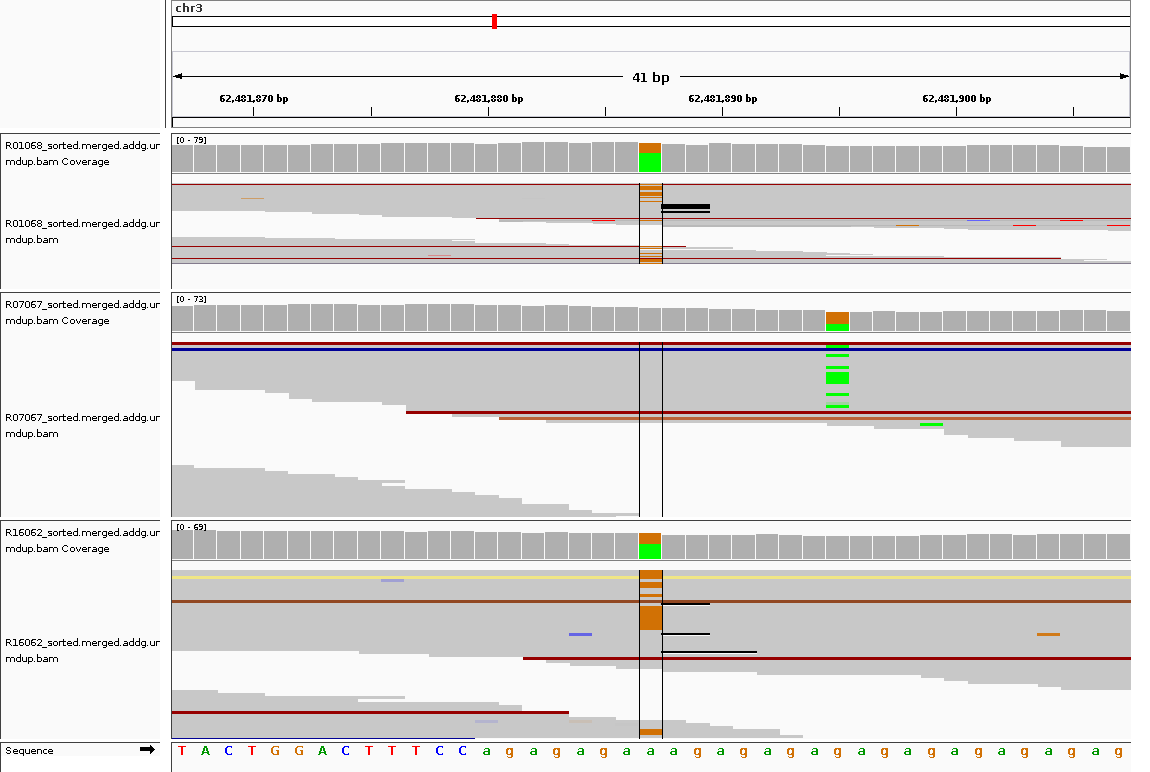

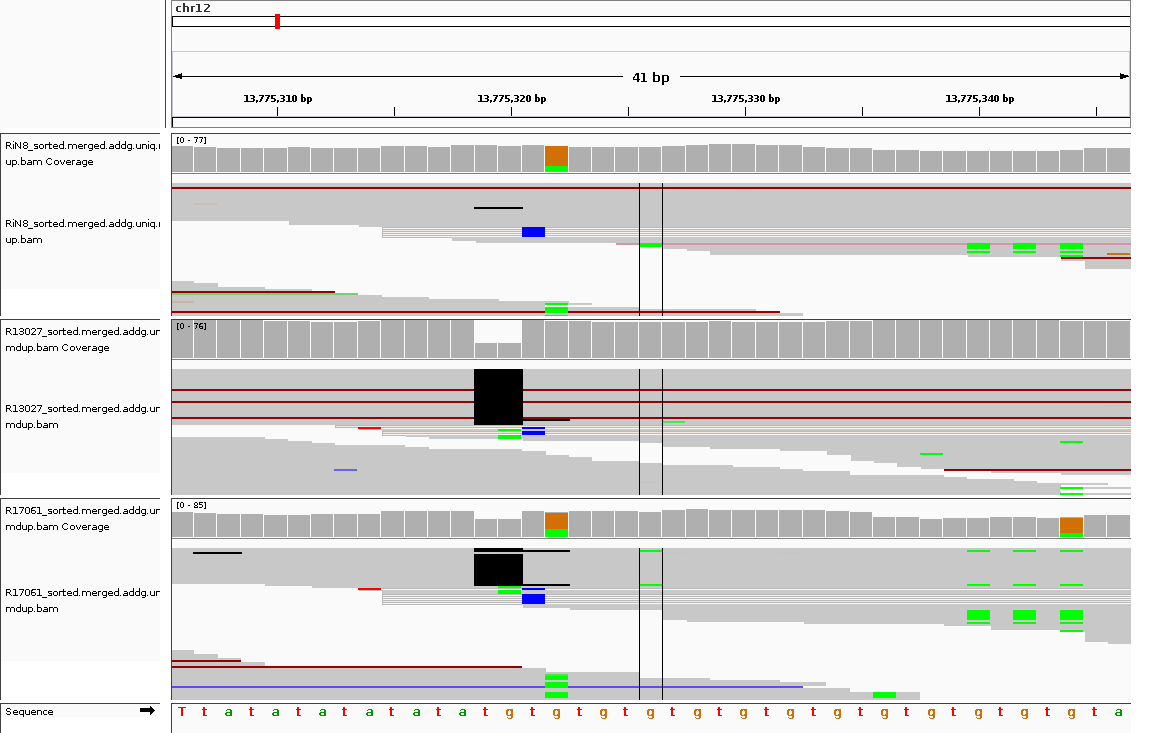

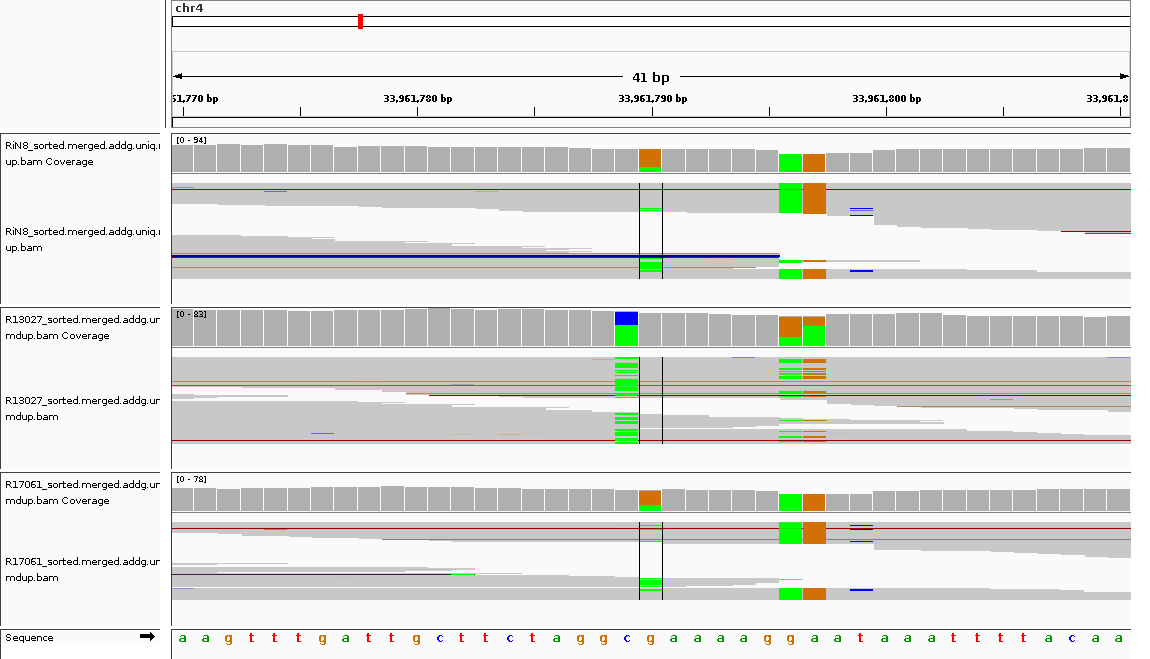

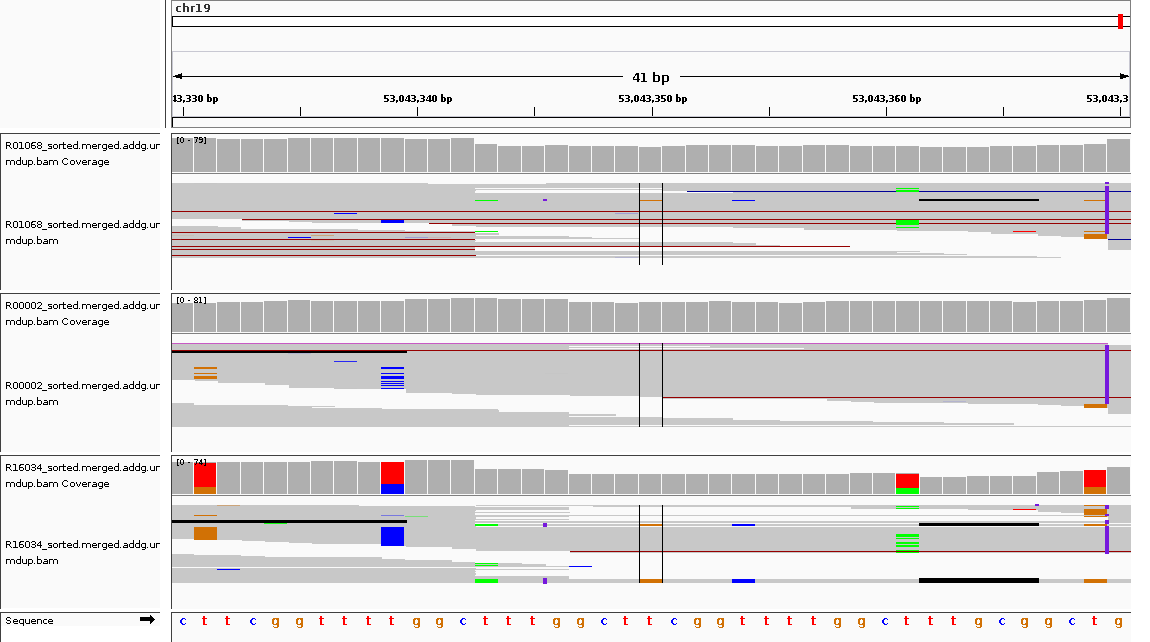

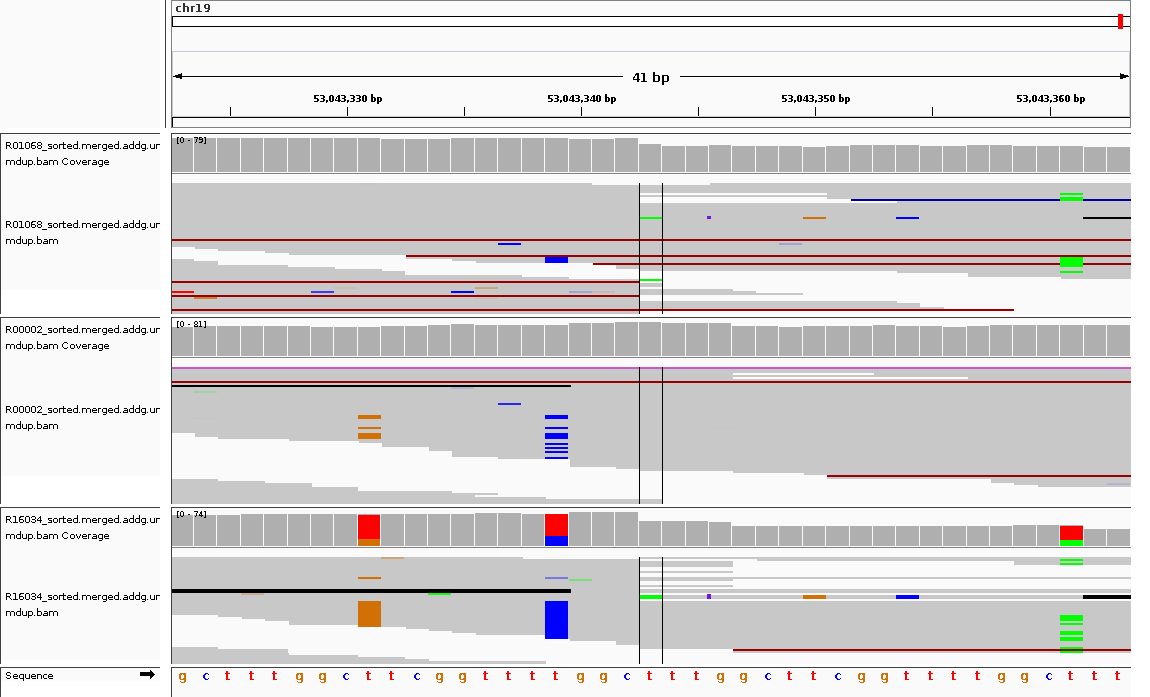

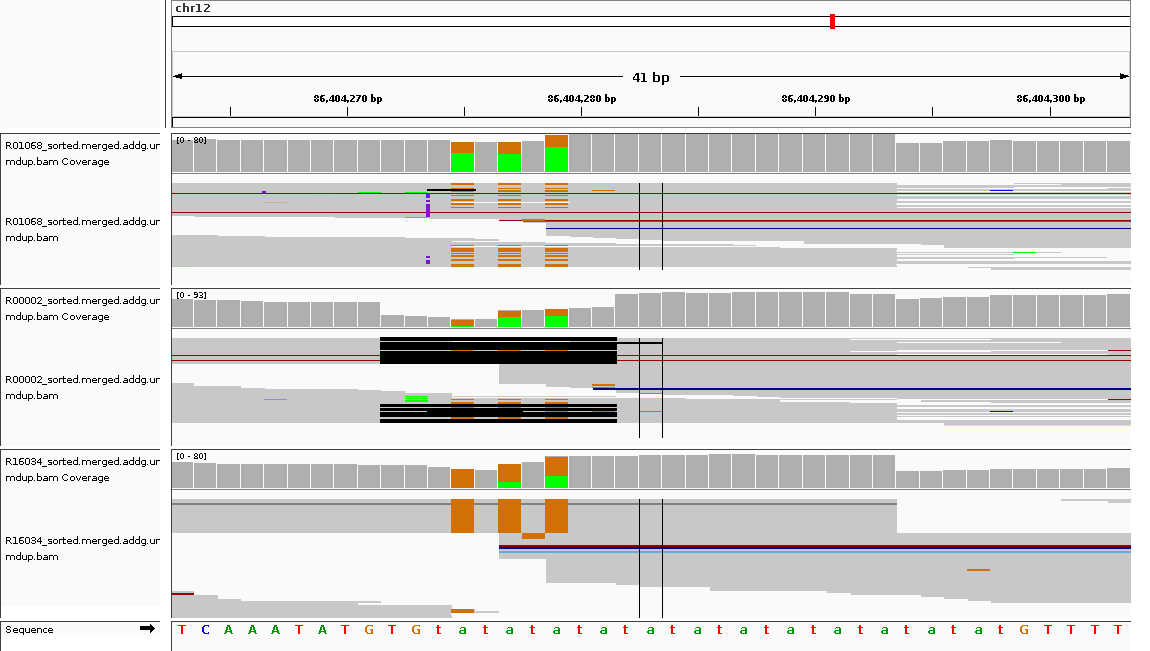

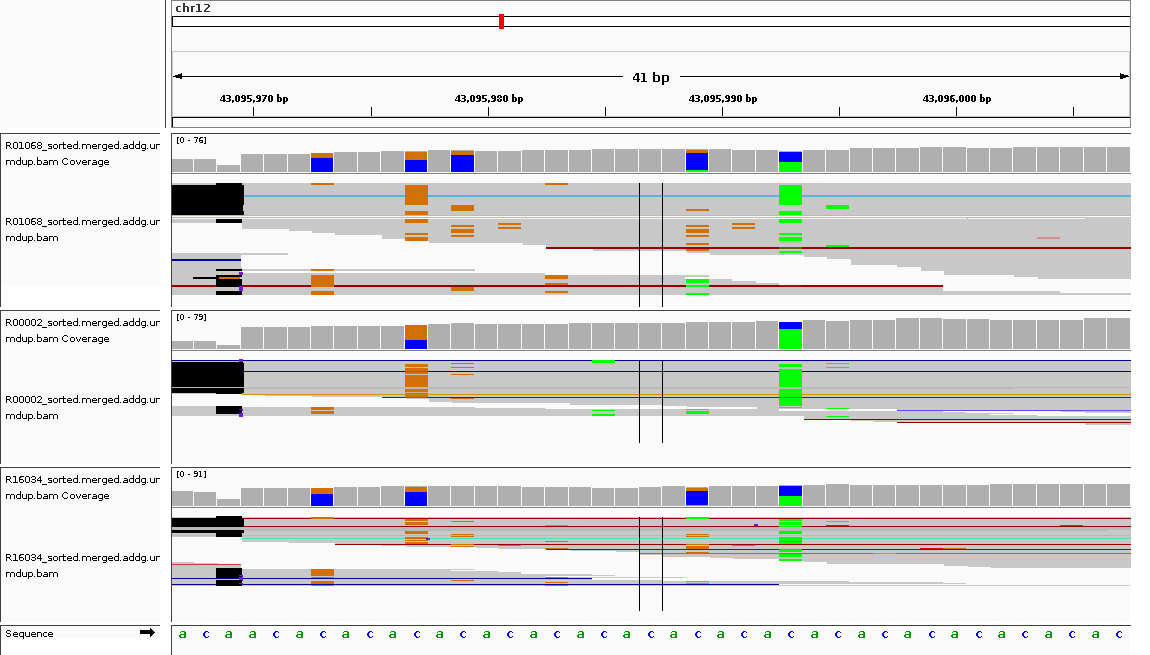

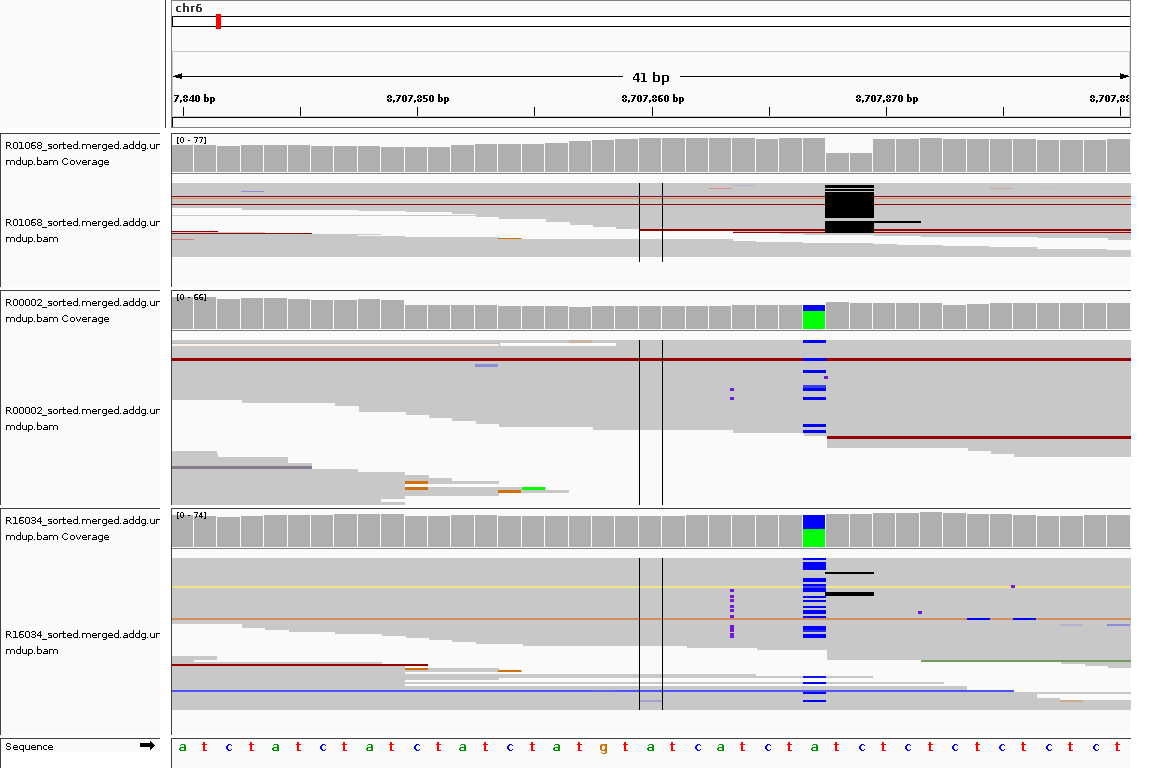

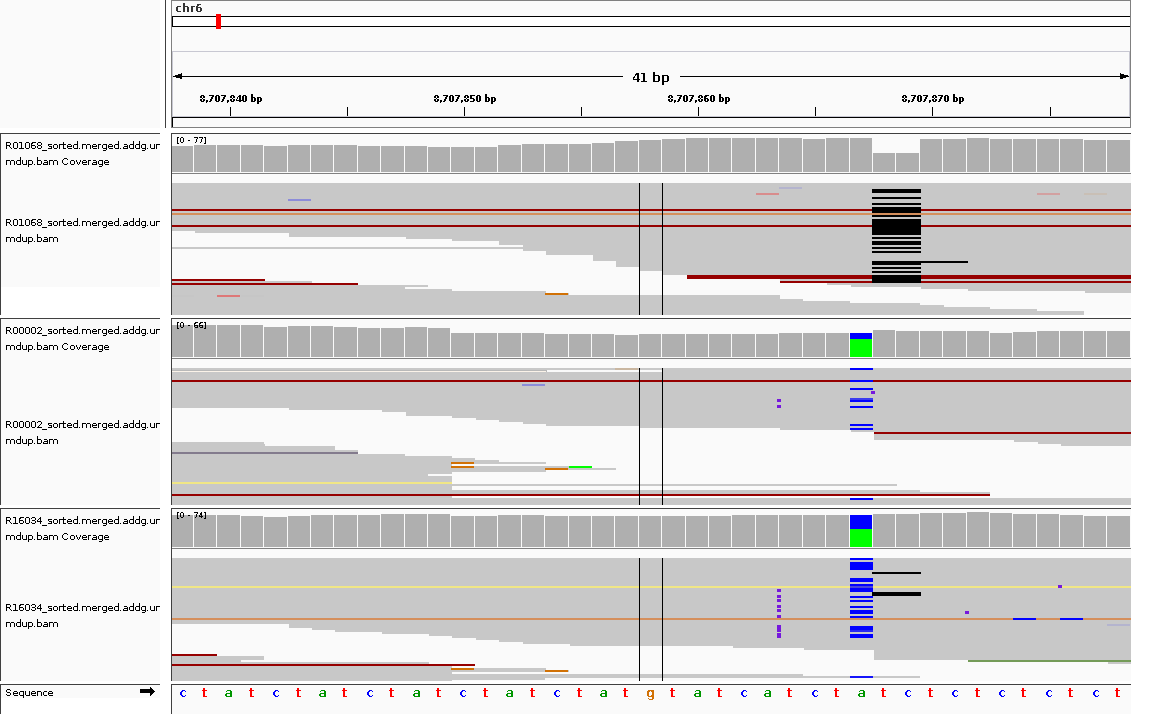

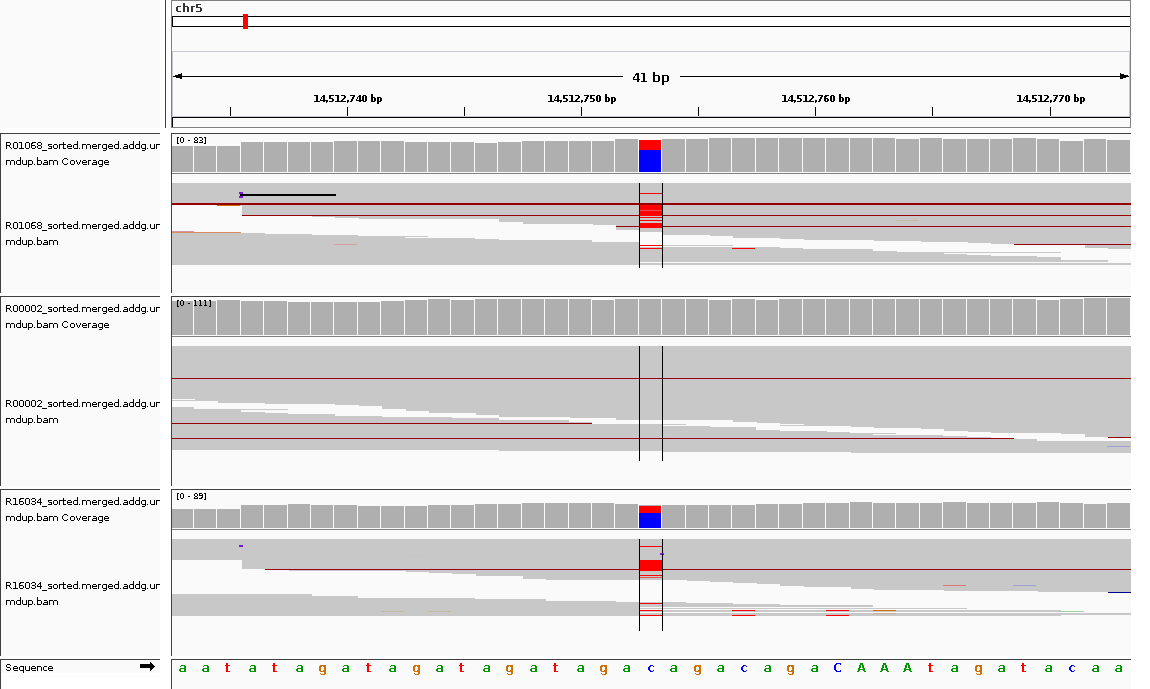

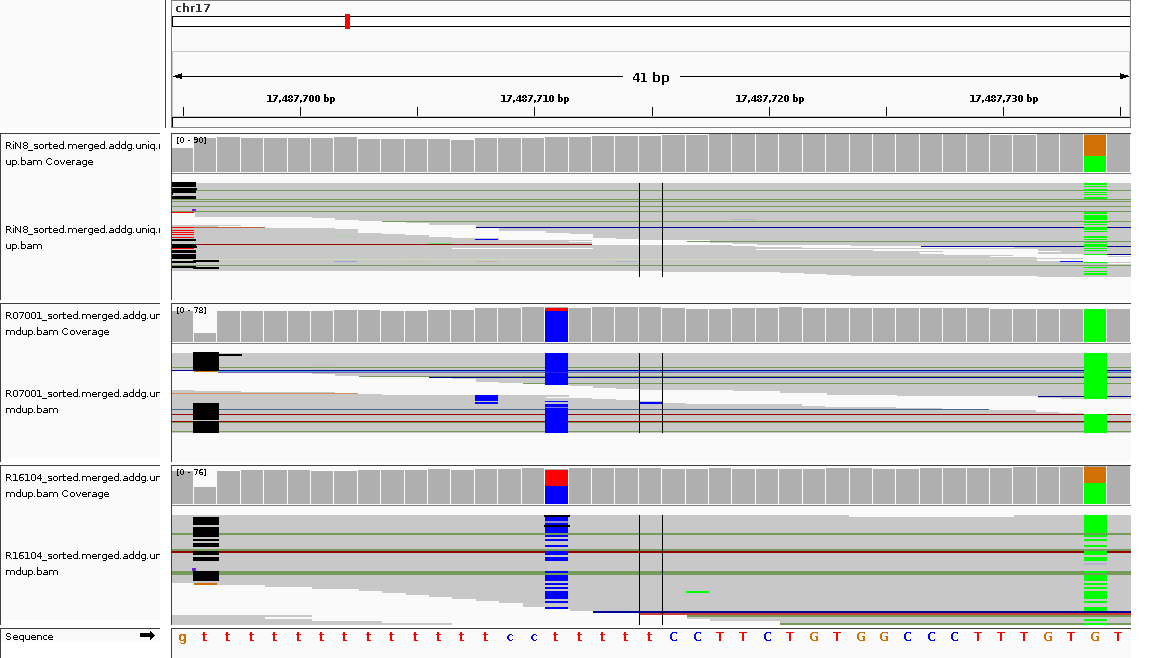

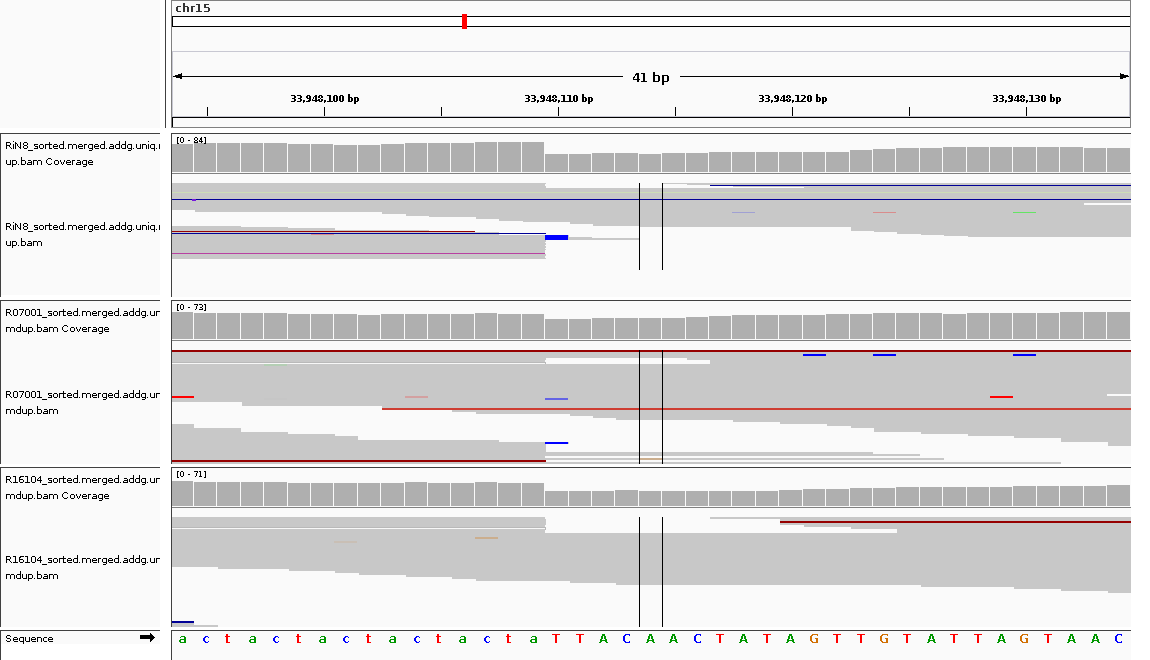

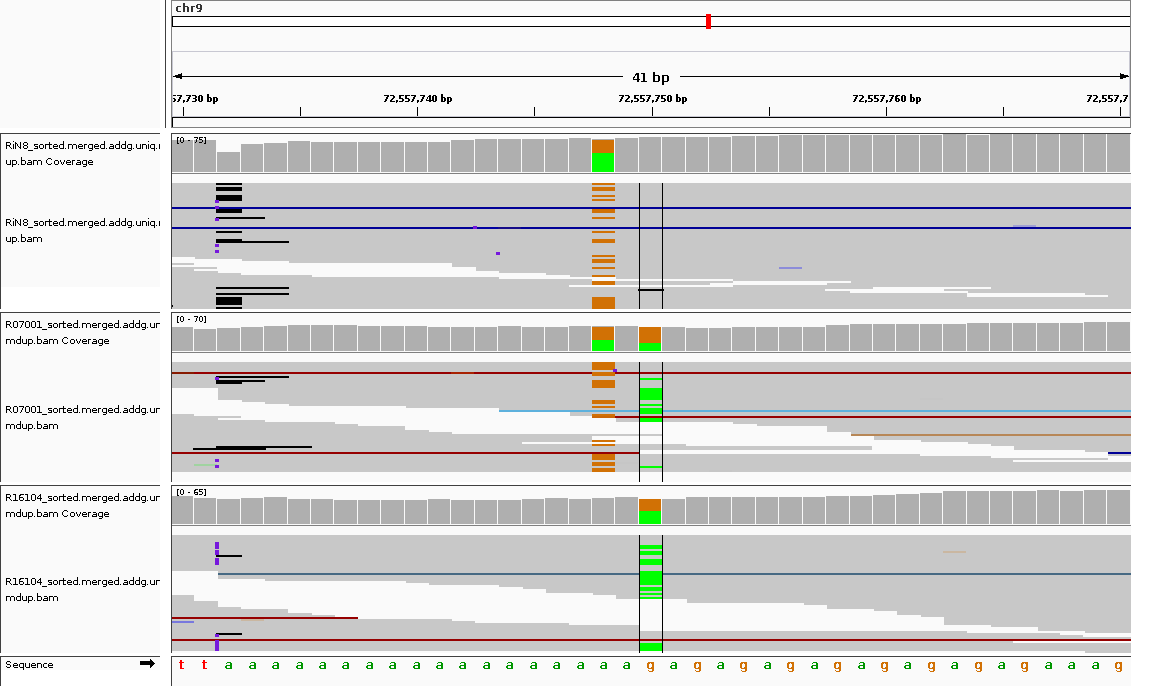

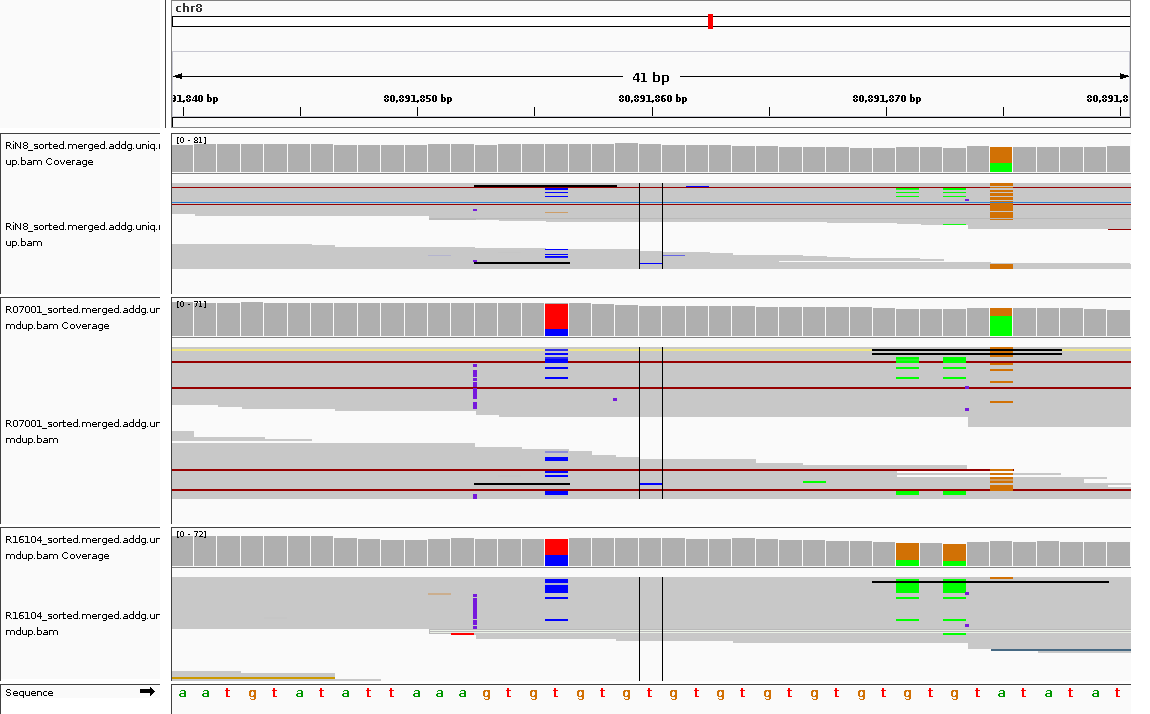

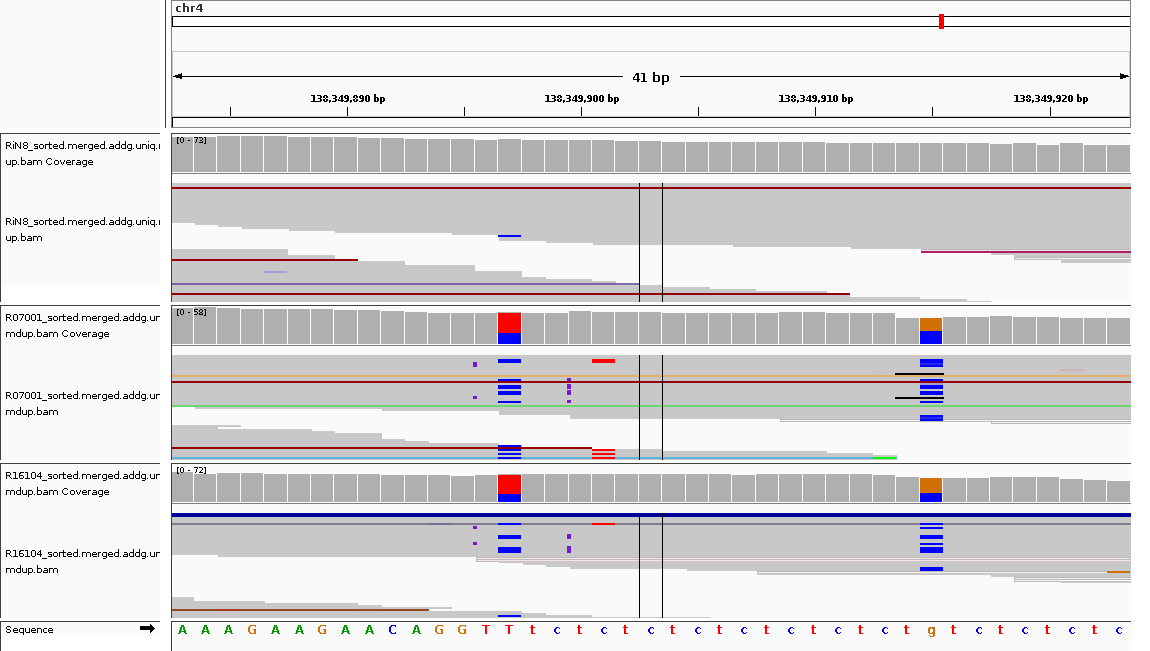

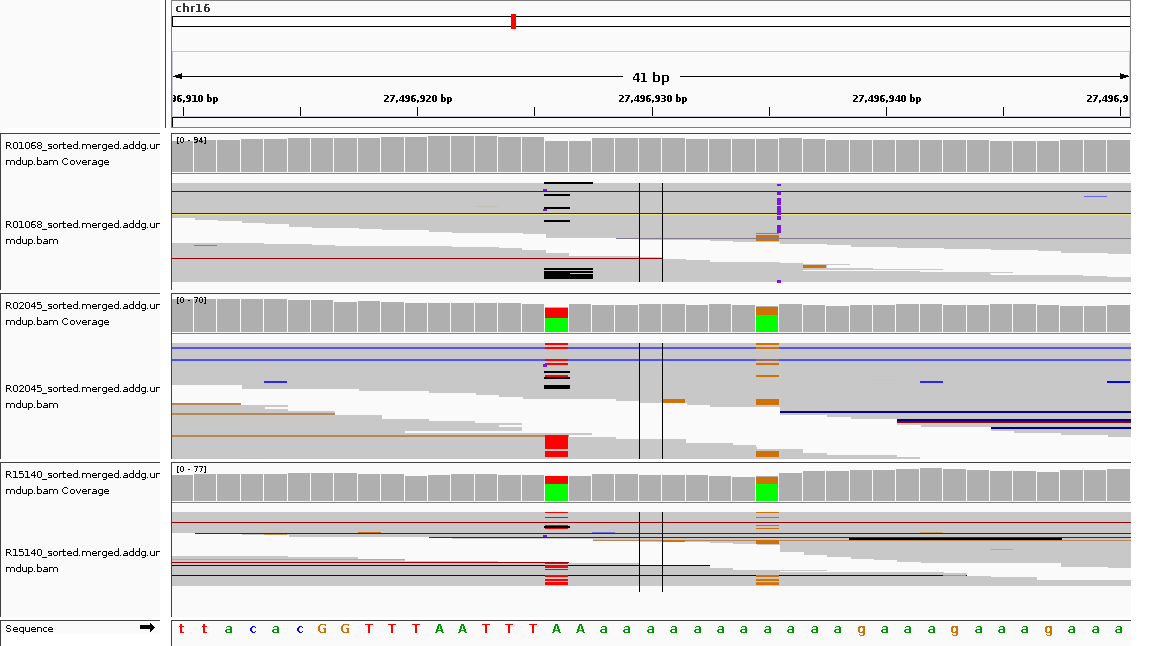

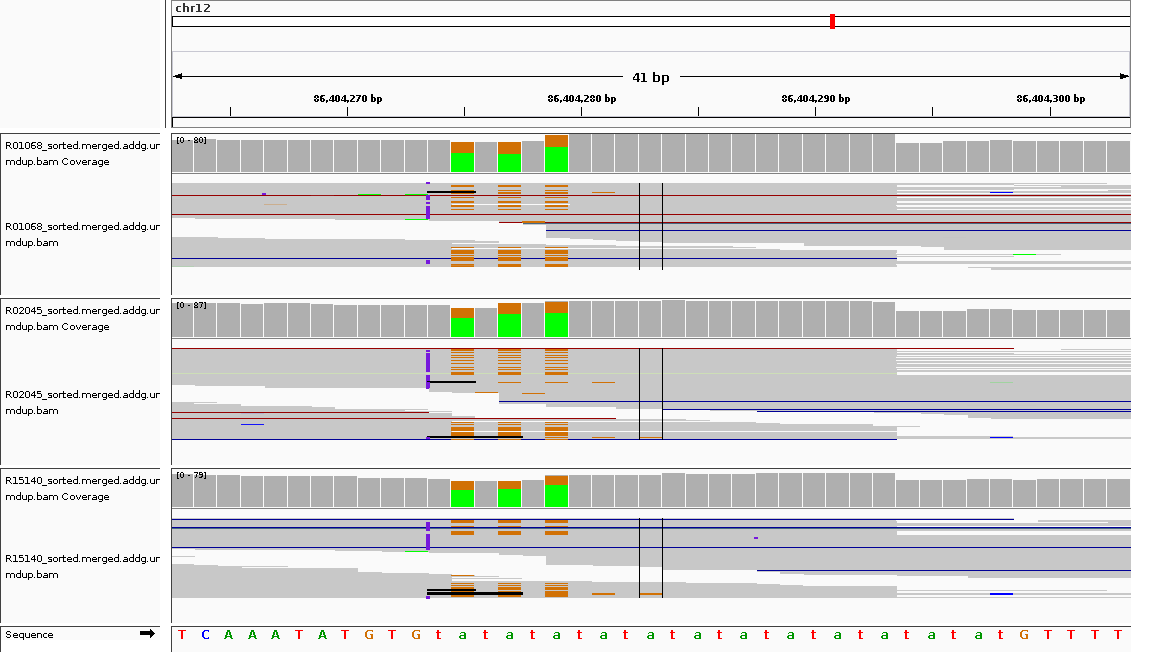

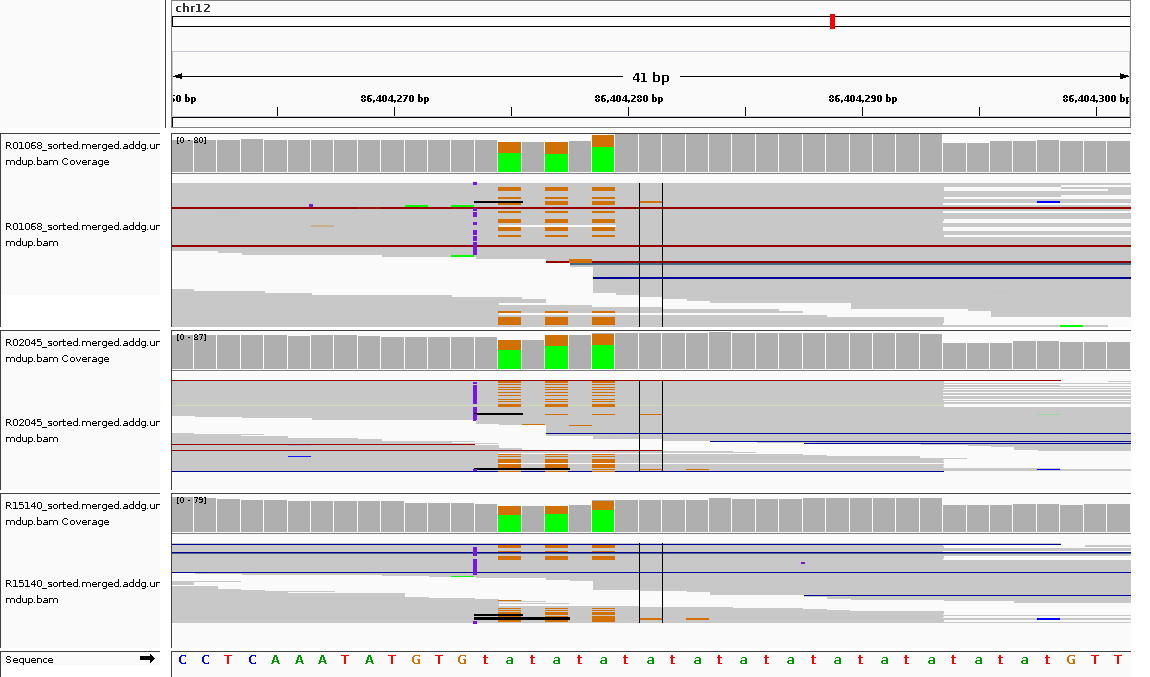

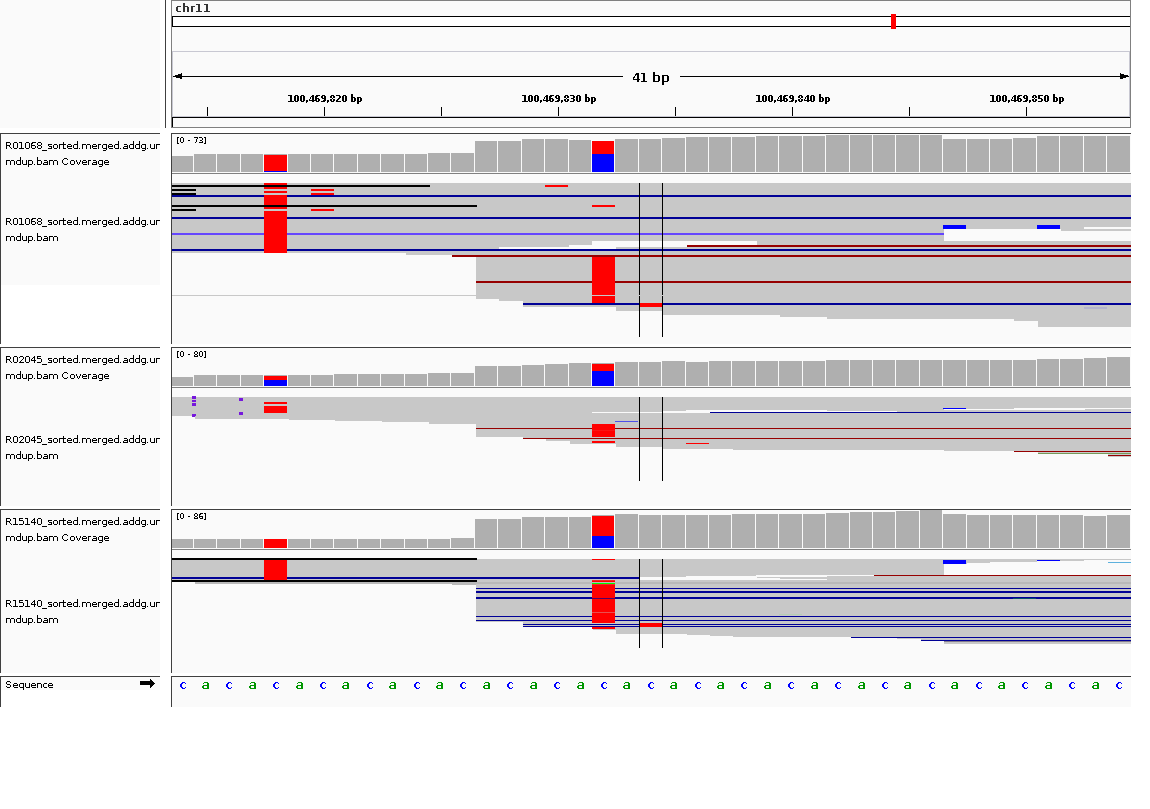

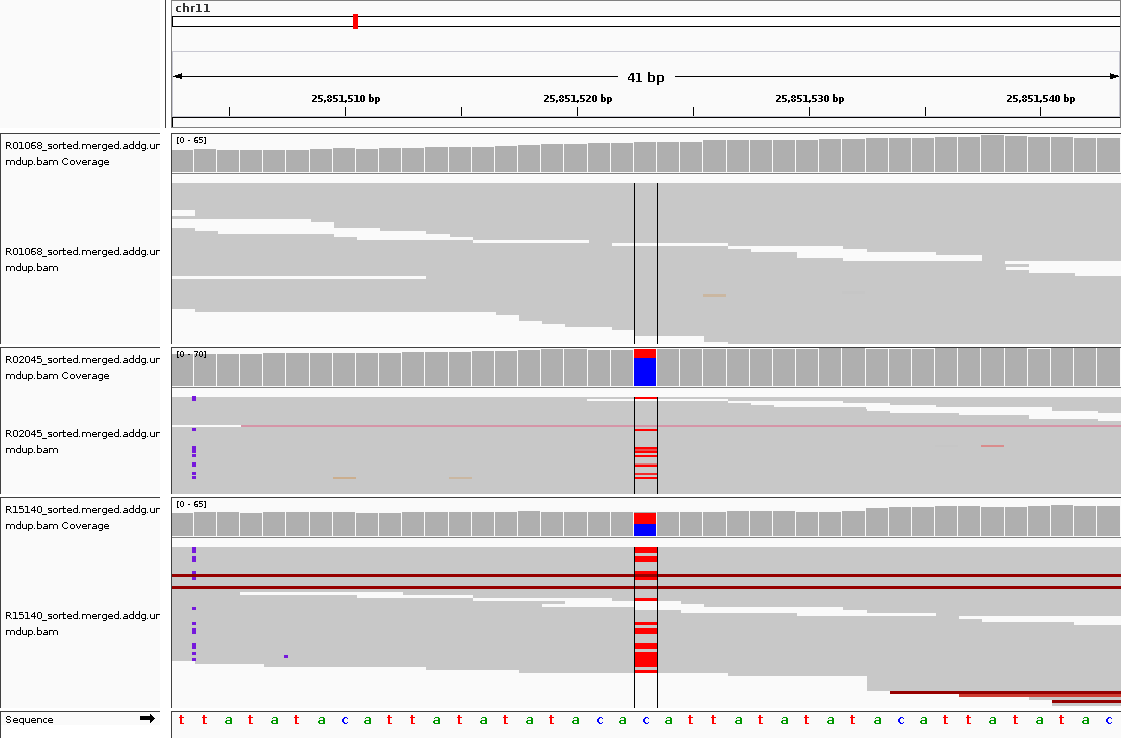

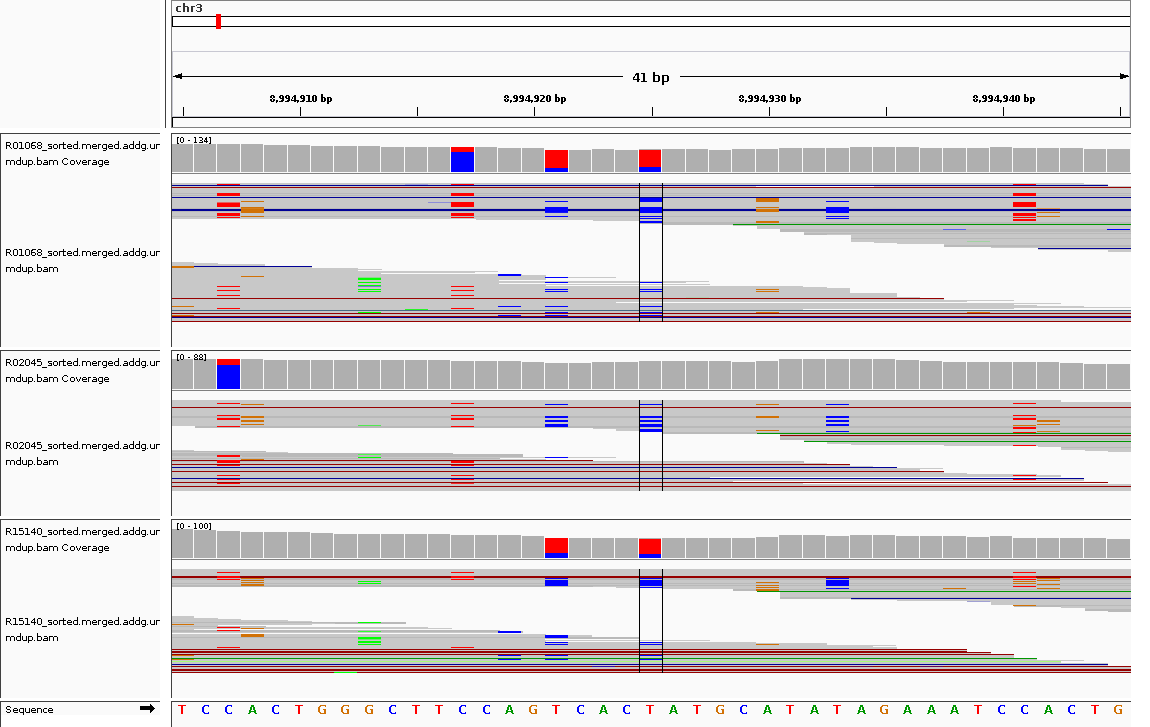

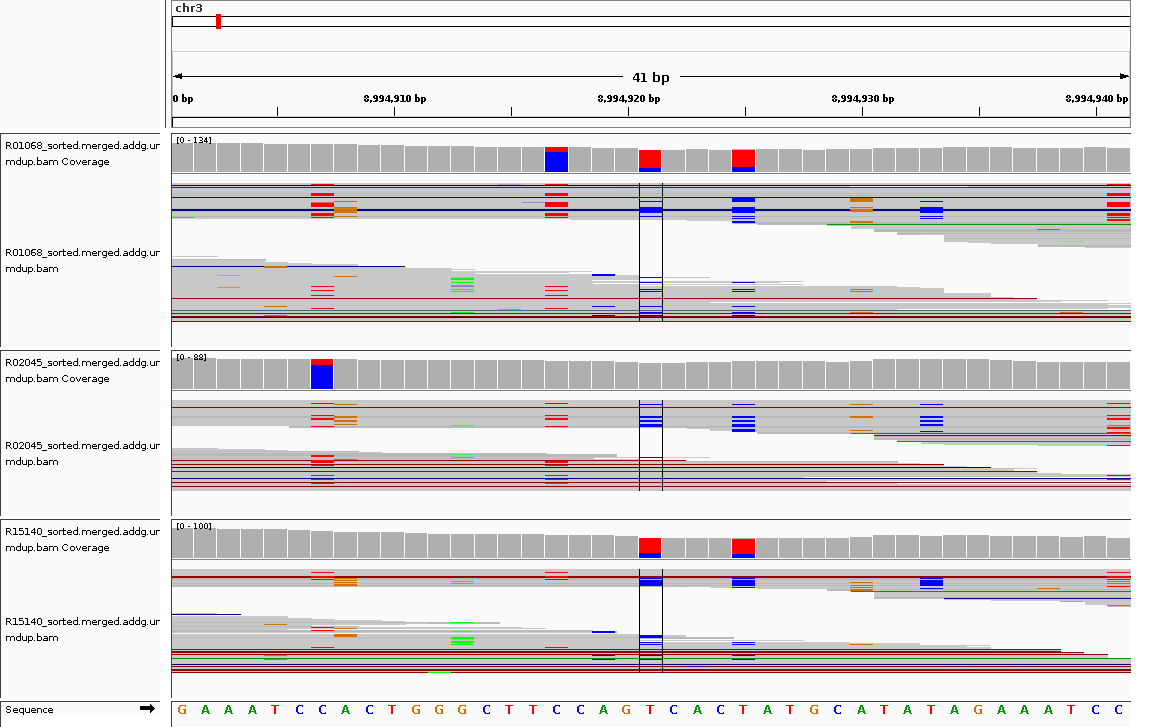

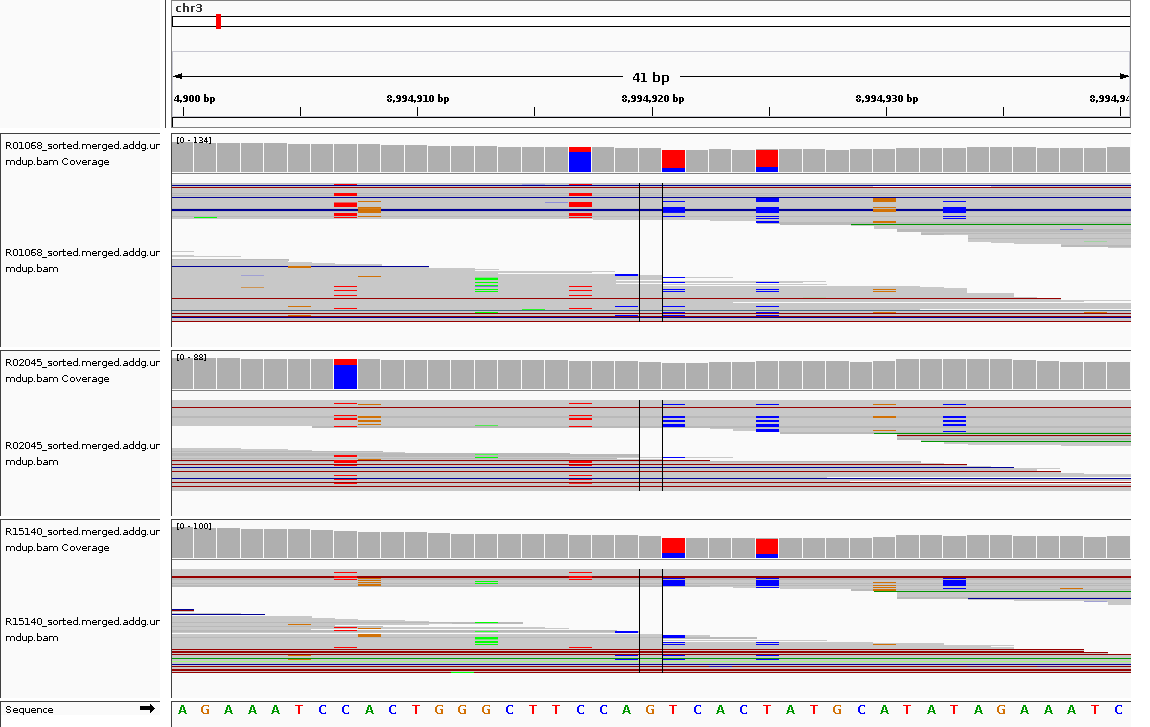

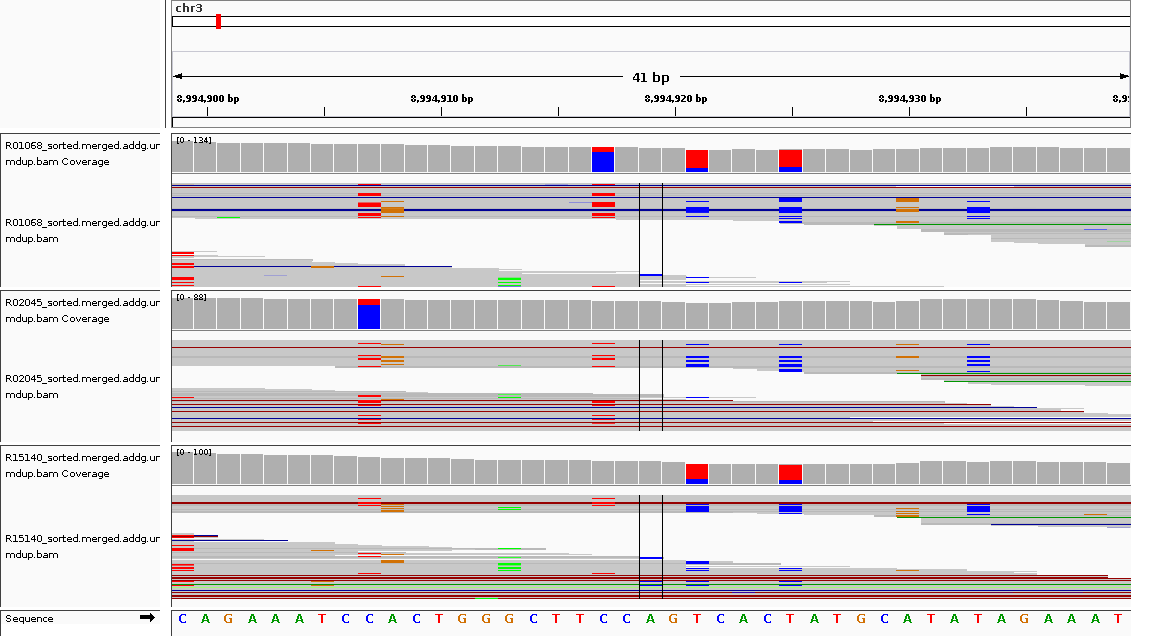

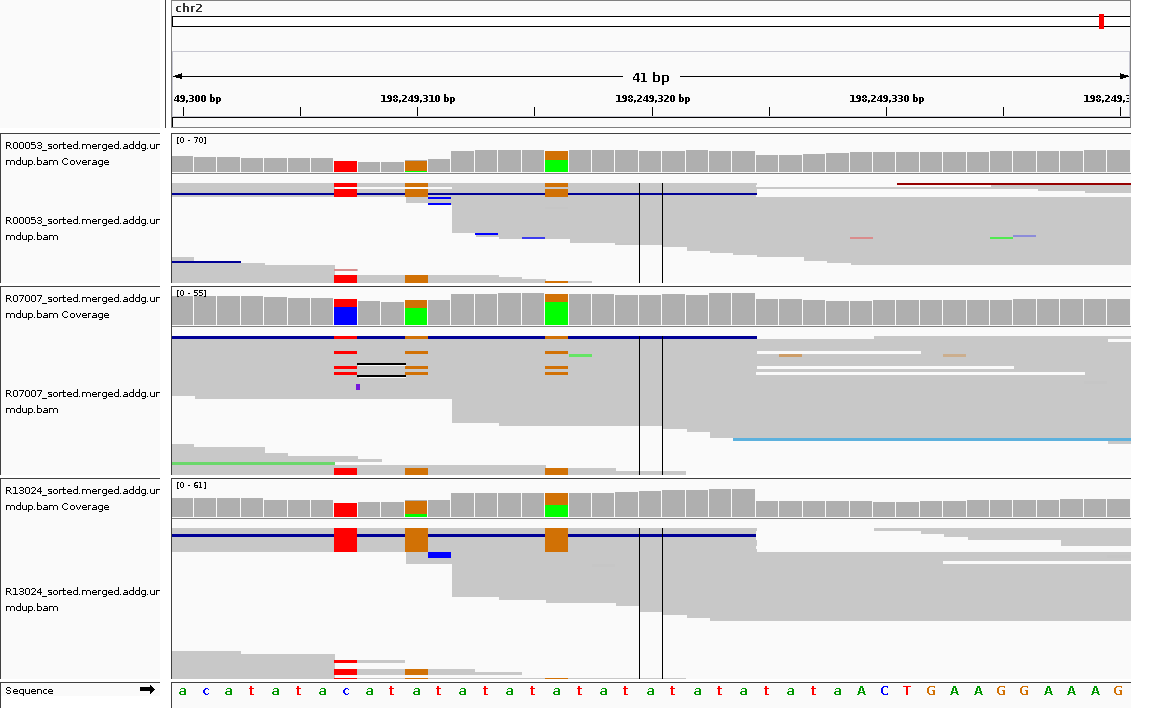

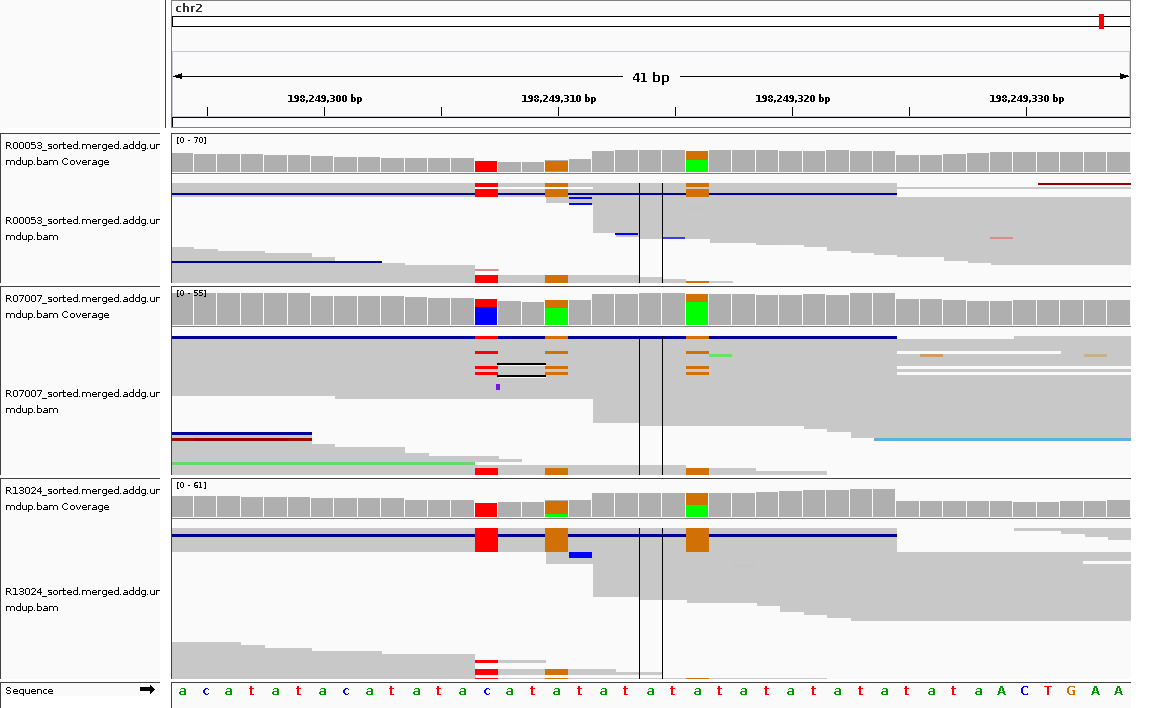

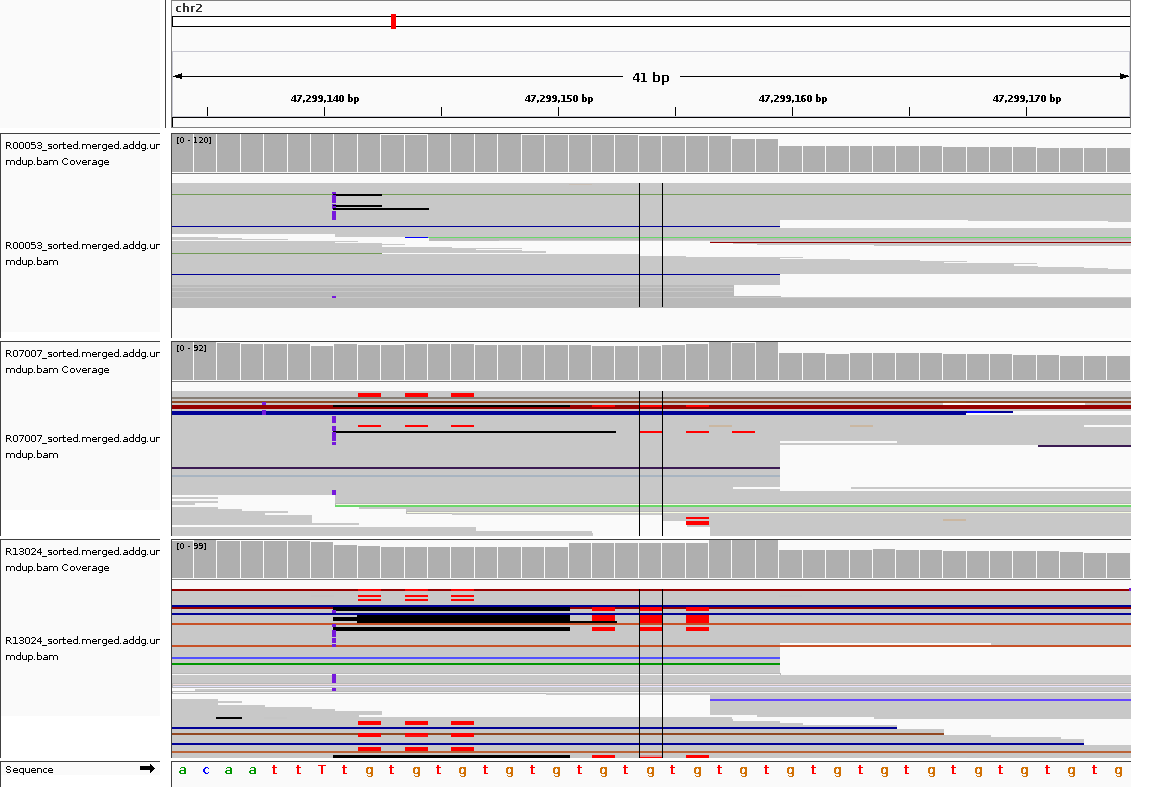

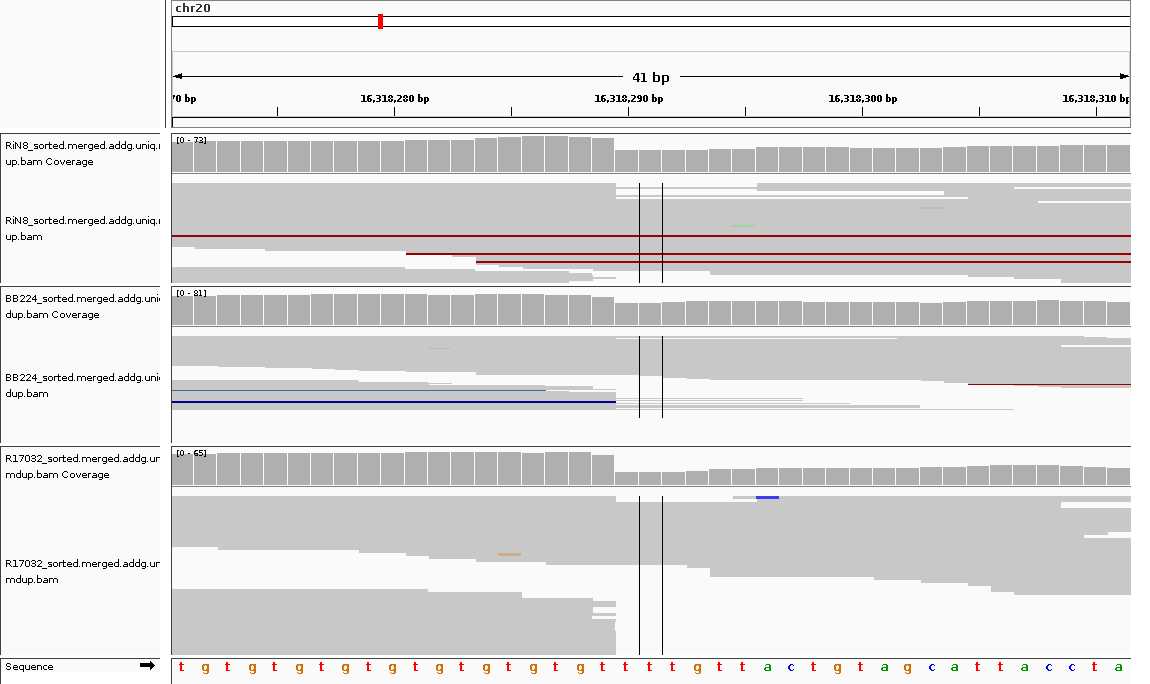

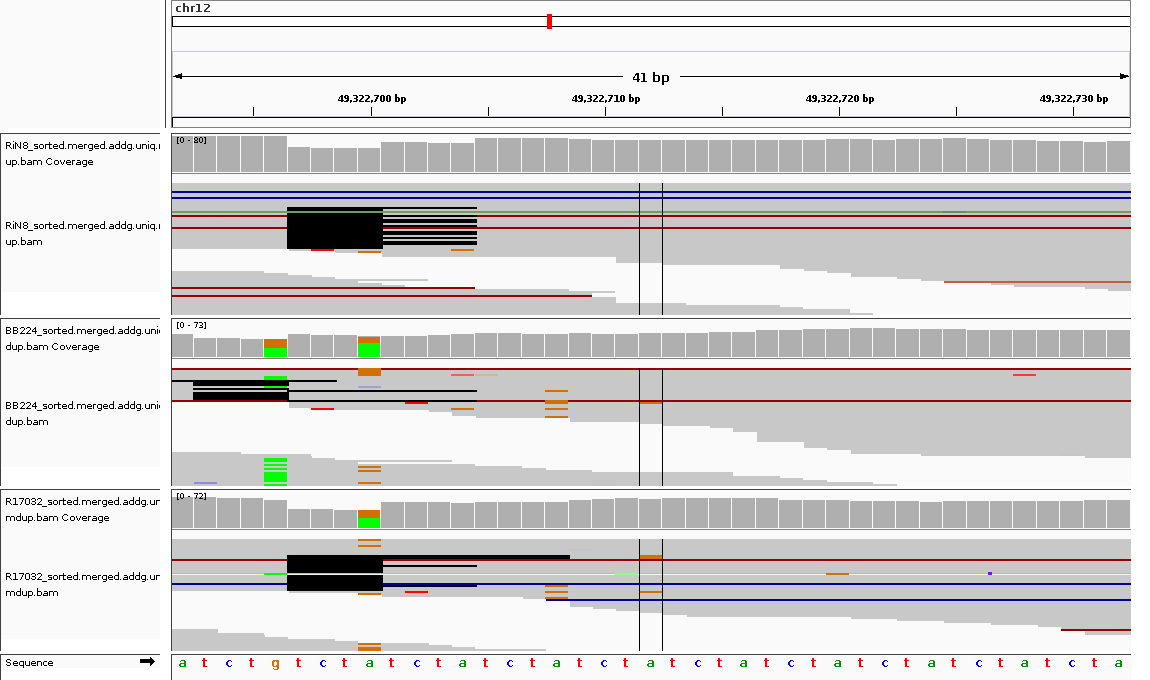

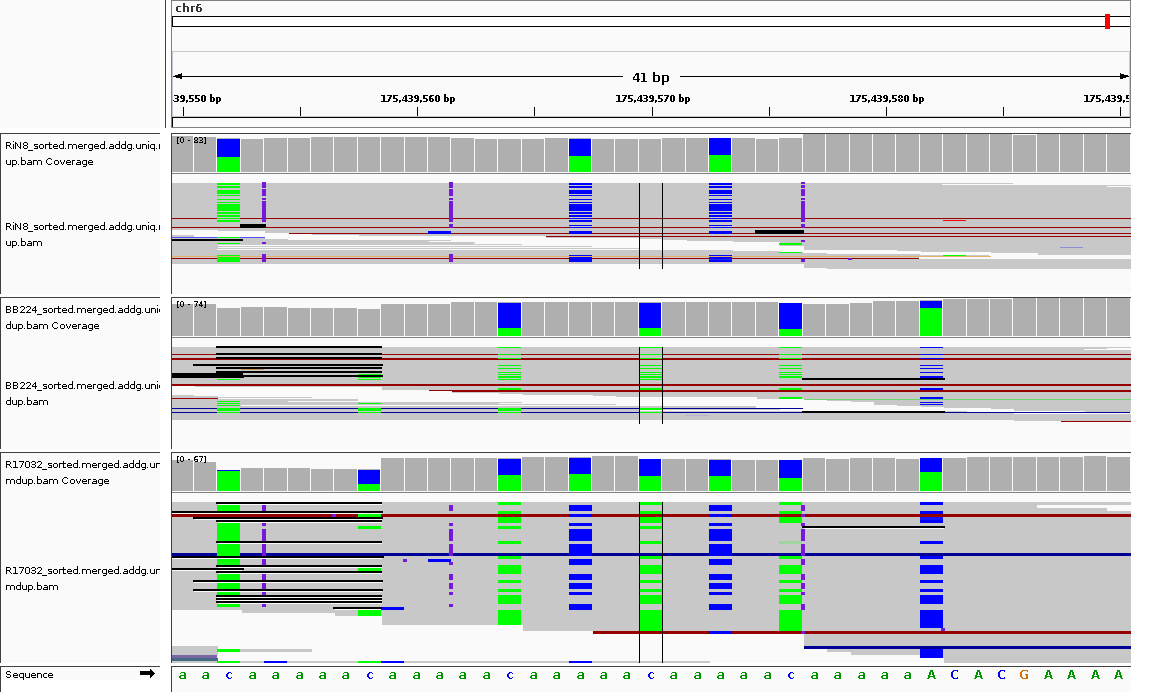

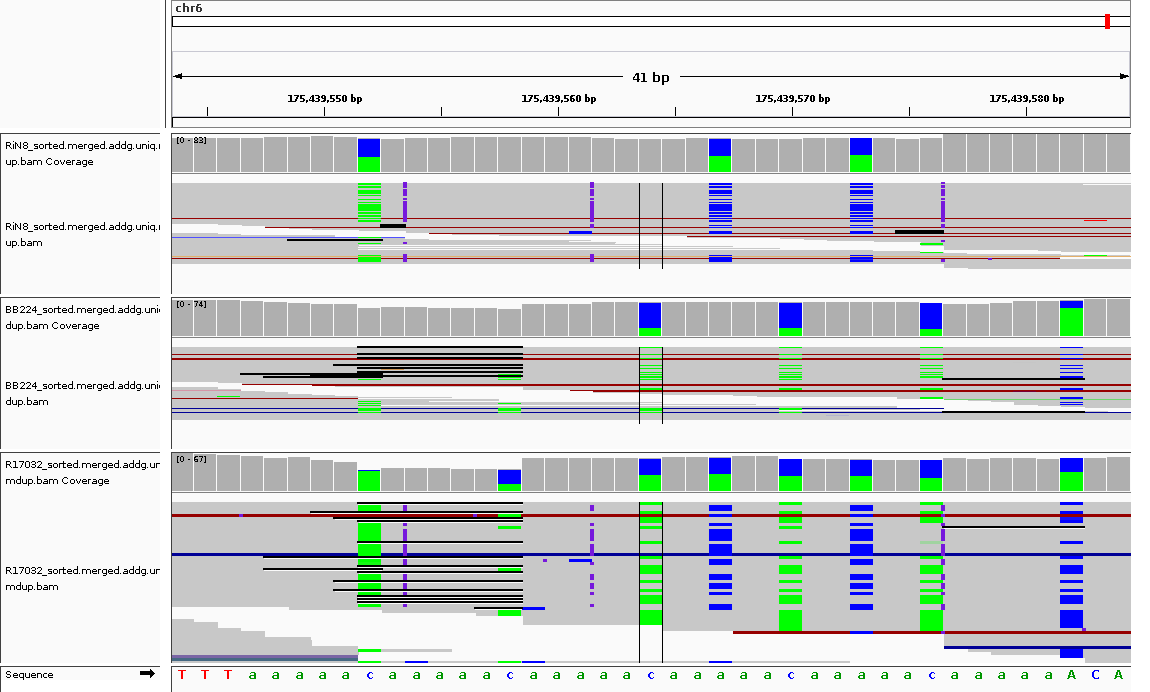

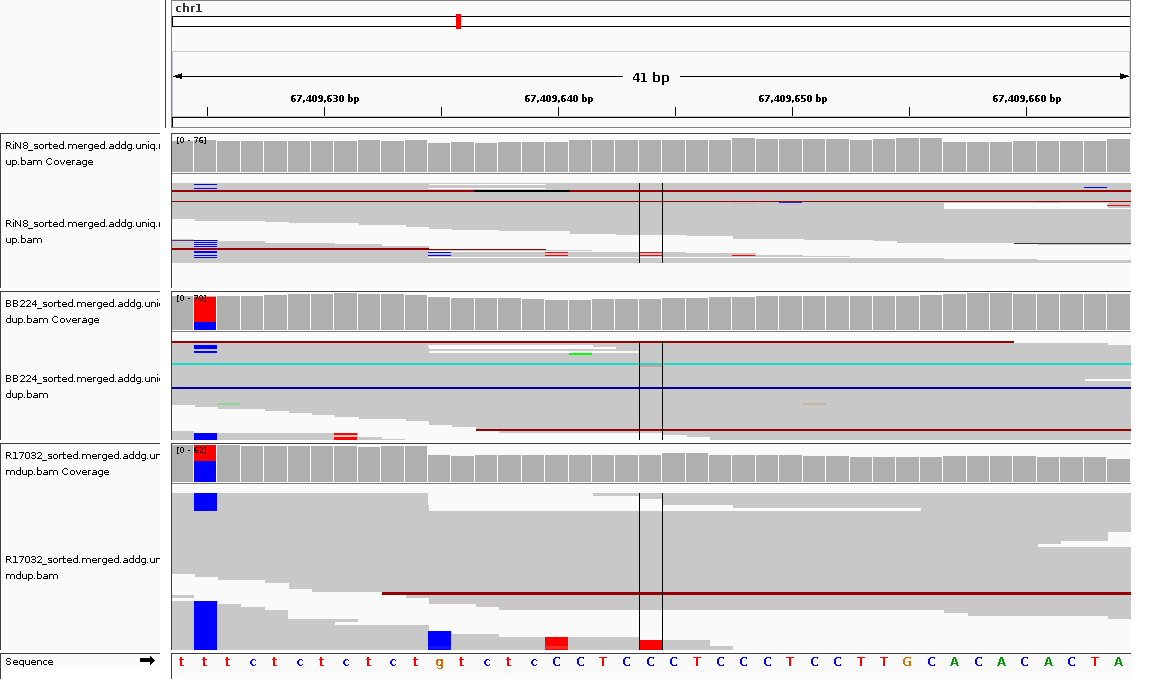

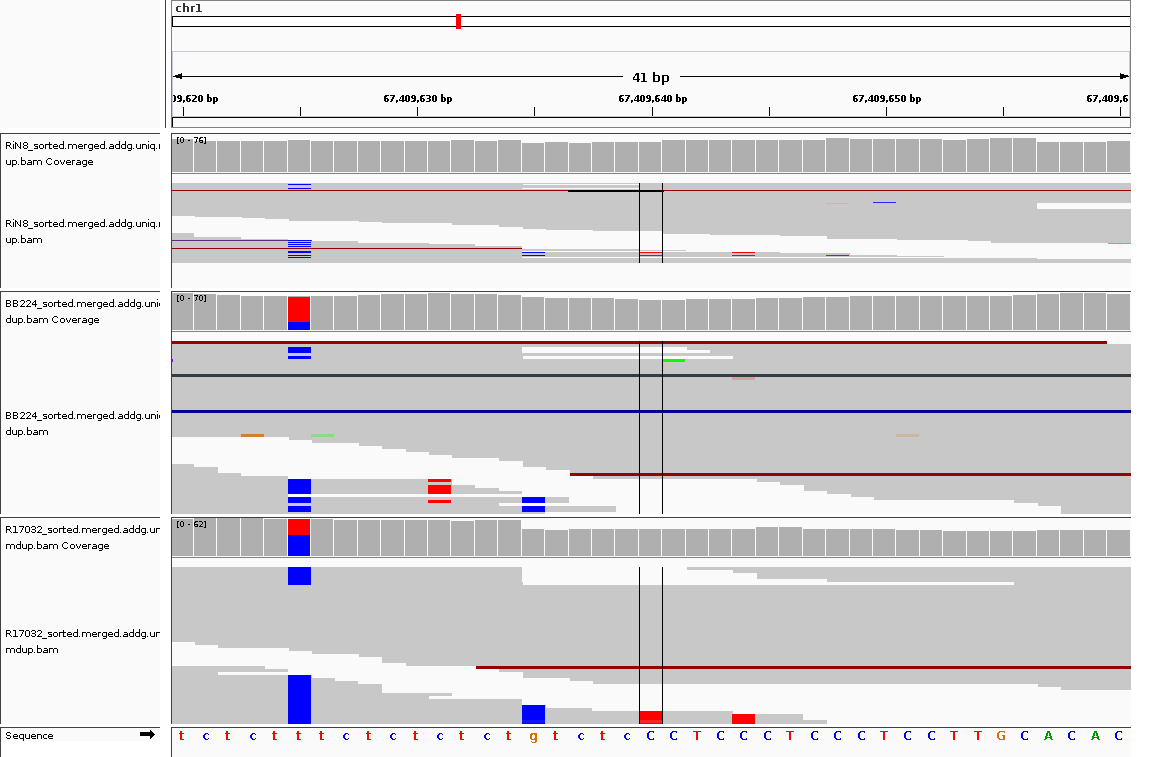

Supplement: giab029_Supplemental_File [file giab029_supplemental_file.docx]
