# Supplementary material for: The germline mutational process in rhesus macaque and its implications for phylogenetic dating
Source: Gigascience. 2021 May 5;10(5):giab029. doi: 10.1093/gigascience/giab029 (PMC8099771; doi:10.1093/gigascience/giab029)
Supplement: giab029_Response_to_Reviewer_Comments_Original_Submission [file giab029_response_to_reviewer_comments_original_submission.pdf]

Author's Response To Reviewer Comments

Close

Answer to the editor:
Although the manuscript is of interest, we are unable to consider it for publication in its current form. Reviewer 1 (Susanne Pfeifer) highlights a number of methodological concerns and also mentions discrepancies with the recent results by Wang et al. The reviewer therefore feels a thorough validation of the pipeline is essential. Reviewer 2 (Jeffrey Rogers) also points out some issues with the analyses, in particular regarding divergence times.

Response: We thank the editor for his consideration of our work.
The comments of Susanne Pfeifer helped us to clarified several points of our analysis. Our method does not differ very much from previously published methods which we hope to have made more clear now. We provide a comparison with a published trio of chimpanzees as a validation of our method. Moreover, we emphasize that the rate we estimated does not differ much from the one of Wang et. al. Indeed if the difference is about 25 % when comparing the per generation rate, the yearly rates (which correct for the parental age) are less than 5 % different from each other.
We found the comments from Jeffrey Rogers highly constructive and we added several changes in the molecular dating part. Especially, we used different pedigree-based estimated rates (from baboons, green monkeys, humans, and chimpanzees) to date different divergence times. As suggested by the reviewer, we also made assumptions on how the rate could have changed over time to conciliate our estimated divergence time with the fossil records.

I am aware that you informed us prior to review that you'd rather defer a full benchmarking and validation to a later publication, as it is part of a larger project, which we understand. However, in light of the reviewers' reports, I feel it is important to show sufficient data and analyses for readers to have confidence in the methods used and to understand differences compared to the Wang et al. study.

Response: We completely agree with the editor that despite our other project on benchmarks and standardization for pedigree-based mutation rate estimation, our rhesus macaque manuscript should by itself provide sufficient data and analyses for the reader to understand (and be confident about) our analysis. We hope that the additional information we have now provided will help the understanding of our method.

Although both reports are rather critical, I am happy to read in reviewer 2's report that the data is important and worthy of publication. Addressing the reviewers' comments will improve the manuscript and may allow a revised version to be published in GigaScience.

Response: We thank the editor for this comment and we hope that our revisions have improved the manuscript.

Reviewer #1:
Using whole genome sequencing data from 33 individuals / 19 trios, Bergeron and colleagues directly estimate the spontaneous mutation rate in rhesus macaque (average rate: 0.77 x 10^-8 per site per generation) which they in turn use to date divergence times across primates.

Overall, this study is highly similar to recent work published by Wang et al. (2020) who estimated the spontaneous mutation rate in rhesus macaque from whole genome sequencing data of 32 individuals. Wang et al. (2020) estimated an average rate of 0.58 x 10^-8 per site per generation. There is a large discrepancy between these two similarly sized datasets which requires an explanation. As expected, the age of the parents is a contributor but, by itself, it is insufficient to explain the observed differences. In fact, it has long been known in the field (and indeed it has been pointed out by the authors themselves here) that differences in the computational pipeline (such as variant calling as well as estimations of

false negative and false positive rates) can have large effects on de novo mutation estimates. The authors implemented their own pipeline which deviates in several aspects from the "best practices" used in similar studies. This is problematic, not only because several of the applied filter criteria can lead to systematic biases, but also because it is hindering a straightforward interpretation of their results. As a consequence, it will be of utmost importance to provide a benchmark using both a well-annotated human dataset as well as the previously published rhesus dataset.

Response: We agree with the point raised by the reviewer on the bias brought by different computational pipelines on the estimated rates. However, we are not aware of any best practices in the field as each group implements its own pipeline. We initiated another project to understand the extent of this methodological effect on estimated rates. A single trio was analyzed by 5 different pipelines and the difference in estimated rates was more than 55 %, stressing the need for standardizing the method to estimate pedigree-based germline mutation rate.
In this study, we found a difference in per generation rate of almost 25 % with Wang et. al. 2020, yet, most of this difference is explained by the parental age. Indeed, when comparing the yearly rate, taking into consideration the parental ages, our estimated yearly rate was only 5 % lower than Wang et. al. We reported this in the discussion (lines), yet, with the reviewer comment, we believe this was not made clear enough in the manuscript and we have now added this information in the results section:
Lines 190-195: “This rate is higher than the 0.58 × 10−8 de novo mutations per site per generation found by Wang et.al. (2020), yet, this difference can be explained by the older age of the parents at the time of reproduction in our study (average 10.4 years old) than in Wang et al (average parental age of 7.5 years). After normalization with the parental age, the estimated yearly rates in these two studies are very close with only 5 % lower in our study.”
To validate our method, we applied our pipeline to a published trio of chimpanzees, from Besenbacher et. al. 2019. For this trio, with Carl as a descendent, the authors found a mutation rate of 1.27 × 10−8 (CI 0.95–1.7 × 10−8), when applying our method, we found a mutation rate of 1.25 × 10−8, less than 2 % difference between the two estimates. We have now added this in the main text:
Lines 147-150: “To validate our method, we applied our pipeline to a published trio of chimpanzee [27], for which the mutation rate was estimated at 1.27 × 10−8 mutations per site per generation (CI 0.95–1.7 × 10−8) and obtained a very similar rate of 1.25 × 10−8 de novo mutations per site per generation.”

Aside from the differences in filter criteria between the studies, the authors claim that their higher sequencing coverage leads to more reliable results. This claim is not supported in the manuscript. Indeed, it is rather surprising given that the reported false positive and false negative rates (10.89% and 4.02%, respectively) are in the range of those reported in previous studies of lower sequencing coverage.

Response: We agree with the reviewer that our claim of a “more accurate” rate due to large coverage was over interpreted. The high coverage of the dataset, compared to other studies, did not reduce the false-negative rate and the false positive rate significantly but allowed us to apply strict and straightforward filters. Strict in the sense that even when using a filter of half the average depth per trio, we end up with a minimum depth of 30X, which with our minimum cutoff of allelic balance filter of 30 % of the reads will leave 9 alternative reads to support the heterozygosity in the offspring. And straightforward, as we could apply the same filters to the denominator instead of using a probabilistic estimation that can be necessary when the coverage is different between individuals in the trio for instance. We have now changed this in the main text by removing the following sentences:
Line 134: “To produce an accurate estimate ...”
Line 384: Such high coverage allowed us to achieve a false-positive rate below 10.89 % and within the regions we deemed callable, we calculated a low false-negative rate of 4.02 %.
Line 397: “… allowed us to gain high confidence estimate …”
And kept only lines 379-382: “Here, we produced sequences at 76X coverage, which allows us to apply conservative filtering processes, while still obtaining high coverage (88 %) of the autosomal genome region when inferring de novo mutations.”

Importantly though, there is also an issue with the way the authors estimate the false positive rate. Specifically, the authors note that "the manual curation may have missed the realignment executed during variant calling". As GATK's HaplotypeCaller locally reassembles genomic regions to determine the haplotype, this is greatly concerning as the authors do not actually visually inspect the final (i.e., reassembled) genomic region using IGV - in other words, they can't actually assess whether a site shows evidence for a de novo mutation or not. This issue becomes apparent in the figures presented in the Supplementary Material. Supplementary Figure 1b highlights a site which has been computationally

classified as a de novo mutation candidate. This site does not show any evidence for an alternative allele in the offspring when, according to the authors own definition ("in the case of a de novo mutation, the number of alternative alleles seen in the offspring should account for ~50 % of the reads"), it should (that is, assuming that the computational pipeline is correctly implemented).

Response: We choose to estimate the false-positive rate from the bam files before realignment. We agree with the reviewer that this choice was not justified enough in the manuscript and we attempt to do so now. We output the bam file from the realignment and explore those files for the 744 candidates. Instead of 81 FP positions, after realignment we found 50 potential FP sites, leading to a false-positive rate of 6.72 % instead of 10.89 %. This would increase the rate of 5 % (to 0.81×10^{-8}). Among those 50 FP candidates, 47 are in common with the non realigned curation. Thus, it is unlikely that using this curation would change our results as only 34 mutations from the previous mutation would be added.
We believe that it is harder to call variants in the realigned regions of the genome and that these regions are more prone to false-positives. In support of this, in one position, we detected a candidate as false-positive before realignment (with no variant in the offspring), after realignment this position looked like a true positive, yet, we validated this mutation in the lab and it happened to be a false-positive. One site is not strong evidence, yet, we decided to keep the before realignment curation method as a conservative choice.
We have now added this in the result section:
Lines 161 - 167: "The manual curation may have missed the realignment executed during variant calling. Doing the manual curation on the realigned reads led to a lower false-positive rate of 6.72 % and a higher per generation rate of 5 % and out of the 50 false-positive candidates, 47 were common to the method before realignment. Thus, in the absence of objective filters and as a conservative choice, we decided to keep these regions in the estimate of mutation rate but corrected the number of mutations for each trio with a false-positive rate (see equation 1 in the Methods section)"

I can't comment on the estimation of the false negative rate, as it is unclear how exactly the simulations were performed (e.g., were sequencing errors incorporated and if so, at which rate and under which model; what proportion of reference vs alternative alleles were simulated, etc.).

Response: The reviewer couldn't comment on our false-negative rate estimation due to an incomplete explanation of the simulation. We understand from the reviewer's comment that we did not clearly explain the way we calculated the false-negative rate. The false-negative rate was estimated as the proportion of callable sites that would be filtered away by the other filters than the one already used for the callability calculation. Thus, we estimated the proportion of callable sites expected to be filtered away by the allelic balance filter as the ratio of true heterozygotes (one parent HomRef and one parent HomAlt) outside the allelic balance filter. We added a correction for the site filters with a known distribution (FS, MQRankSum, and ReadPosRankSum) for which we could infer the expected proportion of good sites that would be filtered away. This method to estimate false-negative rate was used in other studies (Thomas et. al. 2018, Besenbacher et. al. 2019), yet, some studies choose a simulation method (Pfeifer 2017, Wu et al. 2020). Several mutations are simulated on bam files after a second run into the pipeline, the false-negative rate is estimated as the ratio of mutation missed by the pipeline. We did not choose to use this simulation method for our mutation rate estimation, yet, tried it to have an idea of the false-negative rate using this method. To avoid confusion we removed this part about the simulation.

Lastly, it is important to treat non-variant sites (which are used to calculate the denominator) in the exact same way than variant sites. The application of fewer filter criteria to non-variant sites (i.e., the authors state that site filters are being excluded) will result in an overestimate of callability and hence an underestimate of the mutation rate.

Response: We agree with the reviewer that it is crucial to treat non-variant sites (which are used to calculate the denominator) in the same way as variant sites. And the main difference between our mutation rate estimation pipeline and the pipeline used in previous studies is that we take more care to treat variant and non-variants sites equally. In the present study, we produce genotype calls for every single position in the genome - not just the variant sites. This means that we can filter the non-variant sites based on the genotype quality, which is an improvement over previous studies that have had to rely on the sequencing depth as a proxy for the genotype quality at non-variant sites. The few filters that we did not apply to non-variant sites are only filters on quality measurements that are undefined when no alternative alleles are present. And we do adjust the number of non-variants sites with the fraction of heterozygotes variants that hypothetically would be removed by such filters. So we very much agree with the reviewer that treating non-variants sites as equal as possible to variant sites is

essential to calculating the right denominator. And we apologize if we failed to make it sufficiently clear in the text that our method does precisely this. We have tried to make this more clear.
Line 183-186: "As callability is determined using the base-pair resolution vcf file, containing every single site of the genome, all filters used during calling were taken into account during the estimation of callability, except for the site filters and the allelic balance filter, only applicable to variant sites."
Lines 569-574: "We used the BP_RESOLUTION option in GATK to call variants for each position and thus get the exact genotype quality for each site in each individual - also sites that are not polymorphic. So unlike other studies, we do not have to rely on sequencing depth as a proxy for genotype quality at those sites. Instead, we can apply the same genotype quality threshold to the non-polymorphic sites as we do for de novo mutation candidate sites."

Another point that requires additional explanation (though perhaps this might become mute after re-analysis of their data), is the inconsistency of the dates inferred for the human/Old World monkey split with both the fossil record as well as with previously published molecular results. It is also unclear why the authors decided to focus on the model with independent age effects given the previous literature on this topic (was this model the best fit?).

Response: We agreed with the reviewer that the molecular dating was lacking interpretation and we have now modified this last part of our result to date the divergence time of different nodes only using only the macaque rate and the substitution on the macaque branches, and compare this dating with the one using other species rate and branches length (human, chimpanzee, green monkey and baboon). We also estimated a divergence time of the Catarrhini group if allowing a change in mutation rate over time. We tried to justify the use of the model with independent ages in the result section (lines). However, we have now added a comparison of yearly rate using the other model as well and justified our choice:
Lines 299 - 304: "Using the regression estimating the per generation rate given both parental ages, we estimated a yearly rate of $0.7 \times 10{-}9$ mutation per site per year. Yet, as both parental age effects may be confounded in this regression we choose to use the regression yearly rate of the number of mutations given by males and females independently, and the average callability (see equation 2 in the Methods section)."

Availability of data and materials:
Data: BioProject PRJNA588178 does not exist on NCBI.

Response: The BioProject was planned to be released upon publication, but we have now released the BioProject, the BioSample, and the sequences linked to it.

Materials: The authors have made their scripts publicly available via GitHub. As GitHub entries can be altered, I would encourage the authors to deposit their scripts in a permanent repository (e.g., Zenodo).

Response: We thank the reviewer for this useful comment and we have now created a release on GitHub linked to Zenodo: https://zenodo.org/badge/latestdoi/261685907

Reviewer #2: This manuscript reports a novel analysis of de novo mutation rate in rhesus macaques. This species is a major biomedical laboratory animal, and one of the most studied nonhuman primates. Thus, fundamental information about this species, such as the rate of new single nucleotide mutations, is valuable and significant. The authors have investigated de novo mutations across 19 trios (sire, dam, offspring) although several of the offspring share sires, so the 19 trios are not entirely independent. These trios were sequenced to deep (76x average) genome-wide coverage, which constitutes a high-quality dataset. The authors have developed an analytical pipeline that estimates the proportion of the genome that they are surveying for new mutations, the false positive rate for observed parent-offspring differences and the false negative rate (missed true mutations). This pipeline seems to me to be well justified and appropriate. Thus, the estimates of de novo mutation rate presented (0.77 x 10e-8 per site per generation, or 0.62 x 10e-9 per site per year assuming a parental breeding age of 12 for males and 10 for females) are interesting. These results for per generation and per year mutation rates are in my opinion reasonable and valid, worthy of publication.
The authors report two additional observations concerning de novo mutations. They observe that most de novo mutations are passed to offspring by fathers rather than mothers, and that older fathers generate more mutations than younger fathers. Both these observations have been previously reported

for both humans and rhesus macaques. So these findings are not novel, but this report is important because it confirms the one previous report in rhesus macaques and further demonstrates in an independent dataset the generality of the differential paternal mutation effect across different species.

Response: We appreciate this comment from the reviewer.

Major issues:

1) In lines 249 - 258 the authors compare the spectrum of mutation types observed in their study to humans and chimpanzees. These comparisons are fine, but the authors should also compare their data to other published data for rhesus macaques.

Response: We agree with the reviewer and have now added a comparison with the rhesus macaque published by Wang et. al. 2020:
Lines 252-258: "We characterized the type of de novo mutations and found that transition from a strong base to weak base (G > A and C > T) were most common (332/663) and similar to what was already reported for rhesus macaque [29], we found 43 % of those mutations located in CpG sites (Fig. 3a). In total, 23.2 % (154/663) of the de novo mutations were located in CpG sites. This is slightly higher than what has been found in humans, for which 19 % of the de novo mutations are in CpG sites [11], but not significantly (human: X2 = 2.774, df = 1, P = 0.096) and similar to the 24 % reported for rhesus macaque [29]."

2) My most significant concern and criticism of this manuscript relates to the authors' proposed dates of divergence among major primate clades. The authors estimate the divergence of Macaca mulatta from M. fascicularis to be about 3.9 mya, which is slightly but not seriously older than previous estimates that used fossil data to inform estimates of molecular divergence. Somewhat more worrying, but not a substantial problem is the Bergeron et al. estimate for Papio vs. Macaca which is again older than prior published results. Based on my critique below, I have some doubts about this Papio-Macaca divergence date, but the problem is most obvious in earlier evolutionary divergence events.

The major problem related to the Bergeron et al. estimate of the divergence date for Hominoidea vs. Cercopithecoidea. Their estimate of 52.31 mya is far older than previous estimates and seems to be out of the range of potential error in those other results. In order to be correct, the other studies must have made a serious error of some type. Bergeron et al. acknowledge that they are aware that this new estimate conflicts strongly with prior studies. The authors further acknowledge that their direct mutation rate approach to dating divergences can overestimate divergence times due to evolutionary changes in either the mutation rate or generation time, or both (lines 439-441 of manuscript).

One obvious issue is the use of the new macaque mutation rate per year for both hominoids and cercopithecoids. A reader might be tempted to suggest that the best estimate for the human mutation rate should be used for that lineage, rather than the macaque mutation rate. But the human rate per year is slower than the macaque rate, so using the current estimated human rate for the hominoid lineage would slow down accumulation of genomic differences further and hence push the divergence date even farther back in time. This use of human rate for inferences regarding evolution in Hominoidea might seem reasonable, but would only exacerbate the problem that the human-macaque divergence seems to be too old.

I would suggest that one problem with the analysis as presented by Bergeron et al. is the estimated genetic difference between humans and macaques. Their paper cites Moorjani et al. (2016) as the source for the genomic difference, but Moorjani et al. (2016) cites the UCSC browser as it stood in 2014. There are now more recent and more accurate reference genome sequences for both humans and macaques (and other nonhuman primates) that might be used to generate a more defensible estimate of the sequence differences to be used in line 606 of the manuscript. This is unlikely to make a large difference and to solve the dating problem, but best to use the most up-to-date genomic data.

Response: We thank the reviewer for this very constructive comment. We agree that our estimation of the divergence time between human and macaque is somehow problematic. As noted here by the reviewer, using the rate estimated for human exacerbate this problem as the human yearly rate is lower than in macaque, leading to even older estimated divergence time. However, using the chimpanzee rate (of 0.64 x 10-9 mutation per site per year) and its branch length (from Moorjani et. al. 2016) would end up in a divergence time of the catarrhini group ~ 42 Mya. This emphasizes the bias brought by using a terminal branched to infer divergence events that happened < 30 Mya. We also agree with the reviewer

that exploring different (and more recent) genetic divergence is a good alternative to what we have done. Thus, based on the useful comment of the reviewer we have now extended this part and 1. date the divergence time of different nodes only using only the macaque rate and the substitution on the macaque branches, 2. compare this dating with the one using other species rate and branches length (human, chimpanzee, green monkey and baboon), and 3. compare the dating using another, and more recent alignment from Armstrong, et. al. (2020): https://www.nature.com/articles/s41586-020-2876-6.

We changed the last part of the result section, the figure 4 and added a line in the discussion:

Line 465: “Moreover, this time is particularly known to have poor fossil records, and dating of the Catarrhini crown group has been difficult [63]”

Furthermore, I would suggest that, based on the Bergeron results, the common ancestor of hominoids and cercopithecoids may have had a higher mutation rate per year than either extant group. This is plausible since the body size was probably smaller than modern rhesus macaques, and other aspects of demography and biology of the outgroups (platyrrhines and strepsirrhines) suggest significant derived evolution in both cercopithecoids and hominoids relative to the ancestor. So assumptions regarding the use mutation rates estimated from macaque and human pedigree studies may not be valid when trying to infer rates of evolution more than 20 million years in the past.

In order to address this problem of a surprisingly old human-macaque divergence date, I suggest that the authors might explore evolutionary scenarios that calculate the effects of changes in mutation rates, generation time, breeding ages or other parameters on estimated divergence dates. That is, they should attempt to determine whether there is a plausible scenario produced through reasonable assumptions for the relevant parameters that makes the estimated divergence date of cercopithecoids vs. hominoids more recent, i.e. closer to what is concluded based on fossils and other molecular analyses. If there is no plausible scenario available, then Bergeron et al. will have to provide a stronger argument for the severely underestimated age of hominoid-cercopithecoid divergence and the remarkable gap in the fossil record. They would (in my opinion) need to address prior studies by Pozzi et al. (2014, Molec. Phylogenet and Evol. 75: 165-183) and Wilkinson et al (2011, Syst. Biol.60: 16-31) or the reviews of primate paleontology by Harrison or Jablonski and Frost in the authoritative volume Cenozoic Mammals of Africa (2010, Univ. of California Press).

Response: Additionally to using different rate to date divergence time we now also estimated a Cercopithecoidea/Hominoidea divergence time if the mutation rate would have changed before the Cercopithecini/Papionini ancestor:
Lines 352 - 359: “Instead, the mutation rate could have changed over time. As estimating the divergence time of the Papio/Macaca node from both the macaque and the baboon rates conciliate, we could infer that the rate only changed before this divergence event. Back then the mutation could have been higher, for instance, similar to the green monkey 1.1×10^{-9} per site per year [28], leading to a divergence of the Cercopithecoidea/Hominoidea ~ 37.5 Mya and a speciation 29.7 Mya. The yearly mutation of the crown Catarrhini could even have been higher considering the rate estimated in New world monkeys that are smaller primates with shorter generation time (eg. 2.7×10^{-9} per site per year in owl monkeys [19]).”
Line 473-474: “Allowing an increase in mutation rate back in time can reconcile the different methods to estimate divergence time between species.”

If Bergeron et al. wish to defend the conclusion that the date of divergence of hominoids vs. cercopithecoids is more than 60% older than prior studies have estimated (52 mya vs 30-32 mya), then they should make a strong argument that addresses the prior studies at some level of detail. The current manuscript states the conclusion, and provides some general comments, but does not adequately make the case that paleontologists and other geneticists have missed the boat by 20 million years.

Response: We do not wish to defend that and we hope that the modification we provided are more explicit on the issue of using a single rate estimated over one generation when trying to estimate a divergence time < 30 Mya

Minor issues:

Line 111: The sister group of the superfamily Hominoidea is the superfamily Cercopithecoidea, not the family Cercopithecidae. The authors refer to Cercopithecidae in several places through the manuscript. I

suggest that it would be more accurate to refer to Cercopithecoidea.

Response: We thank the reviewer for this comment and have now changed this everywhere we were referring to this group.
Lines 155-6: I believe this line should be corrected to read "…due to the incorrect absence of a call of the variant in the parents or the incorrect presence of a called variant in the offspring…"

Response: We did replace this sentence with the suggested one.
Line 175: Should be "…number of callable…"

Response: We did correct this mistake.
Reviewer: Jeffrey Rogers (Baylor College of Medicine)

Close
