# Supplementary material for: The germline mutational process in rhesus macaque and its implications for phylogenetic dating
Source: Gigascience. 2021 May 5;10(5):giab029. doi: 10.1093/gigascience/giab029 (PMC8099771; doi:10.1093/gigascience/giab029)
Supplement: giab029_Response_to_Reviewer_Comments_Revision_1 [file giab029_response_to_reviewer_comments_revision_1.pdf]

Author's Response To Reviewer Comments

Close

------ Point by point answer to the editor --
Thank you for your patience during the review process of your manuscript. I apologize for the delay, but as you will see from the reports below, the second round of review was unfortunately not straightforward.

Response: We thank the editor for his answer and we understand the delay due to contradictory assessments of our manuscript.

Both previous reviewers have re-assessed the revised version. They disagree with respect to their recommendation. Reviewer 2 supports publication (with some minor revisions), whereas reviewer 1 continues to have major concerns regarding the methods and their validation, in particular with respect to treatment of potential false positives / false negatives.

Response: Reviewer 2 had very constructive comments on the evolutionary implication of our study and we believe answering his comments increased the quality of our manuscript. We are glad that our answers pleased him.
Reviewer 1 has mainly technical concerns. We think that the clarifications asked for are, in general, reasonable and we further detailed the methodological part. Yet, some of the concerns, especially on false-positives / false-negatives, reflect methodological differences applied by different groups. There is not yet a general scientific consensus on which methodological approaches are best. Due to the lack of a set of "best practices", we decided to detail our methodology as much as possible for full transparency. We have tried different filters and methods to reduce false-positives and false-negatives and try to justify our choices as much as possible.

After discussion the situation among the GigaScience editors, we still feel that the data is valuable and of interest for the readers of our journal, and we think the work will be acceptable, in principle.

Response: We appreciate that the GigaScience editor board has reached this decision.

However, before we can proceed, we would be grateful if you could make another effort to address both reviewer's follow-up concerns in a second revised version. With respect to reviewer 1's main concern, I feel it is appropriate to discuss in some more depth the methodological issues and clarify any potential discrepancies highlighted by the referee. Please also add more information regarding the methods for the validation with chimpanzee trio data.

Response: We took into account all the minor comments of Reviewer 2.
Regarding reviewer 1, we have now added methodological details in the manuscript especially on our method to discard false-positives and false-negatives. We also added a part on the chimpanzee analysis in the M&M. Moreover, we took into account the minor comments of the reviewer on methodological clarification, figures, and terminology adjustment. We believe this detailed information has addressed the reviewer's technical questions. However, we incline to avoid detailed comparison on different methods in this study as it is beyond the scope of the current manuscript. We believe we have presented sufficient evidence in this manuscript to support the accuracy of our method for de novo mutation rate estimation. The sooner this method can be published, the earlier the community could be benefited from the extensive comparisons of all existing methods. Therefore, we highly appreciate the editor's decision and have followed your direction to further revise our manuscript.

Please also make sure that all scripts are included in the supporting github repository and that all raw sequencing data is available via the appropriate databases such as NCBI. Other supporting data can also be hosted in our database GigaDB.

Response: We have added the script for our statistical analysis to the Github and Zenodo directories, including our analysis on parental age effect, parental contribution, mutation spectrum, CpG, the

distance between mutations, and random simulation (for this distance analysis), shared between siblings, phylogenetic analysis (Ne, dating, comparison with the literature).
All the sequences used in the study are available.

--- Point by point answer to the reviewers ---

Reviewer #1: DNM detection:
While I agree with the authors that there are no current Best Practices in terms of de novo mutation detection, I was actually referring to the Best Practices in variant calling. Most other de novo mutation studies followed the well-established GATK's Best Practices pipeline and the impact of the authors' modified filter criteria is somewhat unclear. In their rebuttal, the authors state that they now "provide a comparison with a published trio of chimpanzees as a validation of our method", and while indeed mentioned in the main text (lines 147-151), this validation is unfortunately not included in the Materials and Methods section, hindering its evaluation.

Response: In our study, the filter criteria on the variant sites differ from the GATK best practices, yet, we decided to do so based on previous work from Besenbacher et. al. (2019) for the apes' mutation rates and Koch et. al. 2019 on the wolf mutation rate. Our choice was justified in M&M lines 508-514: “These filters were chosen by first, running the pipeline with the site filters recommended by GATK (QD < 2.0; FS > 60.0; MQ < 40.0; MQRankSum < -12.5; ReadPosRankSum < -8.0 ; SOR > 3.0), then, doing a manual curation of the candidates de novo mutations on the Integrative Genome Viewer (IGV). Finally, we identified the common parameters within the apparent false-positive calls and decided to adjust the site filter to remove as many false-positives without losing much true positive calls”.
We agree that our comparison with the chimpanzee trio was not clearly described in the M&M and have now added this information lines 596-607:
“To validate our pipeline we analyzed a trio of chimpanzees with a previously published estimated rate at 1.27 × 10-8 de novo mutations per site per generation [27]. We applied the exact same pipeline and found 54 de novo candidate mutations for this trio, a callable genome of 1,966,477,569 base pairs, and a false-negative rate of 4.6 %. The callability represented only 64 % of the total genome, which was lower than the rhesus macaques callability (~ 88 % of the total genome). This is mainly due to the difference in depth between the parents (~ 35X coverage) and the offspring (~ 45X coverage) in the chimpanzee trio, leading to more filtering when using the average depth of all individuals as a depth filter. When exploring the bam file for manual curation we identified 7 candidates as possible false-positives candidates. Removing these candidates to calculate a rate led to 1.25 × 10-8 de novo mutations per site per generation. On the other hand, keeping those candidates and applying the same false-positive rate as for the macaque trio of $\beta = 0.1089$ led to an estimated rate of 1.28 × 10-8 de novo mutations per site per generation. In either case, our analysis resulted in a similar rate than previously estimated [27].”

False positives:
My main concern regarding the manual/visual validation remains, i.e., given that the authors do not inspect the final, locally reassembled .bam files, they won't be able to discern between true de novo mutations and false positives in their pipeline (see my previous comments). The procedure of analysing intermediate files simply can't work (e.g., looking at the final .bam files can reveal, as mentioned in the rebuttal, that mutations currently classified as a false positive are indeed genuine de novo mutations - and vice versa). The final files used to detect de novo mutations are readily available from their GATK runs so there is really no good reason not to use them. If the authors truly believe that "it is harder to call variants in the realigned regions of the genome and that these regions are more prone to false-positives", why perform the variant calling that way?

Response: We apologize that our rebuttal was not clear enough on this point. Indeed, we have performed the manual curation on the final realigned bam file outputted by GATK during HaplotypeCaller as suggested by the reviewer and compare the results with our actual method.
First, the realignment was performed during variant calling and we agree that this step is important to increase the quality of variant calling and discard potential false-positive variants.
We manually explored the 744 candidate mutations on the bam files before realignment (similarly to what is described in our method) and the 744 candidate mutations on the final realigned bam files. On GATK 4 the realignment step is not separated from the variant calling, yet, it is possible to output the realigned bam files. In the realigned bam files we found 50 potential false-positive candidates. Among those, 47 (94 %) were also detected by the manual curation before the realignment. Thus, applying a manual curation after realignment only detected very few (3) additional false-positive candidates.

In contrast, the manual curation after realignment failed to identify 34 candidates discarded by the manual curation before realignment. One of these 34 candidates was included in our PCR validation in another paper we are preparing now where we have compared the de novo mutation sites identified by different methods. And our PCR validation has confirmed this locus was a true false-positive call with no variant in the offspring. We did not include this result in the current manuscript. But this additional evidence supports that a false-positive site found by the manual curation before realignment was hidden by the manual curation after realignment as the candidate appeared then as a true positive. We thus applied a more conservative filtering strategy in the manual curation as a conservative choice to exclude the false-positive sites as many as possible.
Finally, by performing the manual curation after realignment as suggested, we obtained a lower false-positive rate of 6.72 % compared to 10.89 % before realignment, and a mutation rate only 5 % higher than the one we estimated previously (0.81 × 10-8 instead of 0.77 × 10-8 with 95 % CI: 0.69 × 10−8 - 0.85 × 10−8). Such difference is very minor and within the confidence interval of our estimation, thus, should not change the main results of our study.
We modified the comparison of the two manual curations lines 550-561 to clarify it:
"We compared the manual curation methods on the reads before realignment with a manual curation on the realigned reads outputted by GATK HaplotypeCaller. The manual curation on the realigned reads led to a lower false-positive rate of 6.72 % instead of 10.89 % and a 5 % higher per generation rate than the rate estimated with manual curation before realignment. This difference is rather small and within the confidence interval of our estimated rate. Moreover, 47 out of the 50 false-positive candidates found with the manual curation after realignment was also detected in the manual curation method before realignment. However, the latter had a larger set of potential false-positive candidates. Thus, in the absence of objective filters, we decided to use a conservative strategy and keep all sites but corrected the number of mutations for each trio with a false-positive rate (β = 0.1089) according to the manual curation before alignment (see equation 1). The 81 false-positive candidates were removed for downstream pattern analysis."

False negatives:
Thank you for your clarification. In short, the authors estimate the false negative rate for their site filters, however, there are many other reasons why a genuine de novo mutation might not have been detected as such (e.g., due to mis-mapping of reads by the aligner). These down-stream factors need to be accounted for when estimating the false negative rate - and this can only be achieved by simulation (i.e., when the ground-truth is known).

Response: To make sure that there are no misunderstandings we will first expand our clarification a bit. In order to calculate a mutation rate two things are needed: 1) the number of mutations and 2) the number of sites where a mutation could have been called.
One strategy to do this is to genotype all polymorphic sites and apply strict filters to identify the number of de novo mutations among that set and then identify the callable fraction of the genome by simulating reads with variants from all parts of the genome and see how many of them can be detected using the same filters.
The strategy we use in this article is a bit different. We do not only genotype the polymorphic sites in the genome - we genotype every single site in the genome. This means that we can not only apply strict filters to find a set of sites that we are sure are mutated in each trio. We can also apply the same strict filters to find the total number of sites that we are sure that we can call correctly in that trio. Since mismapping will also impact the depth and the mapping and genotype qualities this filtering will also remove regions with mapping problems. The false-negative rate that we talk about in the manuscript is thus only to account for the filters that only can be applied to polymorphic sites, such as what is the probability that a genuine heterozygous variant fails our "allelic balance filter" because it is seen in less than 30% of the reads.
To validate our strategy we have, as mentioned in the previous version, also run some simulations.
We simulated 552 mutations (between 21 and 36 per trio) with an allelic balance between 40% and 60% using Bamsurgeon. They were simulated on the sites we considered as callable (as it is on those sites that we want to apply a correction for FN). Out of these mutations, 545 were detected as de novo mutations, the remaining 7 simulated mutations were filtered out by the allelic balance filter of 30% to 70% due to the read filters applied by GATK. These simulations show that the regions that we deem to be callable really are callable - since all the mutated sites were correctly called by GATK. And the fact that some of the simulated mutations fail the "read balance filter" is not a problem since we do take this into account when calculating the rate.
We are aware that the simulation method is used in several studies (Jonsson et. al. 2017, Pfeifer 2017, and Wu et. al. 2020), yet, others do not use this method. Similar methods to what we applied were used in Besenbacher et. el. 2019 in which a false-negative rate was estimated from site filters and in Thomas

et. al. 2018 in which a false-negative rate was based on the allelic balance filter. There were also no simulations in Tatsumoto et. al. 2017.

Availability of data and materials: Bioproject PRJNA588178 is incomplete - it only contains data from 33 individuals (in contrast, lines 485-499 mention that "whole blood samples (...) were collected from 53 rhesus macaques.")

Response: We understand from the reviewer's comment that lines 479-480 were misleading. Only 33 individuals were used in this study (the initial 20 additional individuals happened to not be related as expected and were discarded from the analysis). Thus, Bioproject PRJNA588178 is complete and we removed the initial number of individuals from this sentence in M&M.

None of the scripts used for the actual analysis (i.e., once the de novo mutation candidates were identified) or plots are included in the current GitHib directory (in addition, the newly created Zenodo repository is not included in the manuscript). For completeness and reproducibility, these scripts should be added.

Response: We thank the reviewer for this comment and have now added a directory on GitHub with scripts for the analysis including parental age effect, parental contribution, mutation spectrum, CpG, the distance between mutations, and random simulation (for this distance analysis), shared between siblings, phylogenetic analysis (Ne, dating, comparison with the literature).
We have created a new Zenodo repository with these scripts and added the DOI in the manuscript.

Other comments:
lines 91-93: "The mutation rate of baboon (Papio anubis) [30] and grey mouse lemur (Microcebus murinus) [31] have also been estimated in preprinted studies." The mutation rate of baboon has been published in August 2020 (Wu et al. 2020).

Response: We thank the reviewer for pointing out this. Indeed, we already changed the citation for the published version of the paper, yet, did not change this sentence. We have now corrected this.

lines 495-496: "Whole-genome pair-ended sequencing was performed on BGISEQ500 platform, with a read length of 2x100 bp." Could the authors please verify that their sequencing protocol was PCR-free (as PCR errors are problematic for the detection of de novo mutations)?

Response: In this project, we have tried to reduce the PCR cycle in library preparation, yet, they were not PCR-free and had 7 PCR cycles.

lines 501-521: BWA and IGV references are missing. What version of Picard was used?

Response: We have now added the citations for BWA and IGV and added the version of Picard 2.7.1.

lines 526-528: "Mendelian violations were selected using GATK SelectVariant and refined to only keep sites where both parents were homozygote reference (HomRef), and their offspring was heterozygote (Het)." My initial reading was that the authors ran gatk SelectVariant (e.g., "gatk SelectVariants -V variants.vcf --select ''vc.getGenotype("father").isHomRef()' --select ''vc.getGenotype("mother").isHomRef()' --select ''vc.getGenotype("offspring").isHet()'") - however, after reading lines 542-544 (i.e., "2,251,363 were potential Mendelian violations found by GATK (...), 177,227 were filtered Mendelian violations with parents HomRef and offspring Het"), it sounds like the authors might have run a two-step process instead, i.e., first "gatk FindMendelianViolations", then "gatk SelectVariants". Could the authors please clarify?

Response: The reviewer understood our method correctly. We ran 2 different steps: 1. selected all mendelian violations with GATK SelectVariants --mendelian-violation -ped pedigree, then 2. imported the table in R and applied all individual filters: refined mendelian violation as parents HomRef and offspring Het, DP filter, GQ filter, and AB filter. The reason is that we initially intended to explore the candidate mutations other than parent HomRef and offspring Het, for instance, parent HomAlt and offspring HomRef.
We have now clarified this in the M&M:
"Thus, for each trio, we applied the following filters using R:
(a) Mendelian violations were selected using GATK SelectVariant (--mendelian-violation) and refined to only keep sites where both parents were homozygote reference (HomRef), and their offspring was

heterozygote (Het).”

lines 569-572: "We used the BP_RESOLUTION option in GATK to call variants for each position (...). So unlike other studies, we do not have to rely on sequencing depth as a proxy for genotype quality at those sites." Several earlier studies actually used GATK in BP_RESOLUTION (including my own study in green monkeys).

Response: We thank the reviewer for this comment. We did not find any studies that explicitly mentioned the use of the BP_RESOLUTION mode and assumed that the common GVCF mode was used. We apologize for the misinterpretation and have now removed that claim.

lines 608-616: The paragraph concerning "Characterization of de novo mutations" is lacking any detailed information. For example, how was the window size or the number of mutations to simulate chosen? How were the mutations actually simulated (e.g., which software was used, which parameters, etc.)? Where was the annotation of the reference genome obtained from?

Response: We have now added the following details to this section: 1. We chose the window size of 20 kb as in Besenbacher et al. 2016 to compare to human DNMs clustering 2. We simulated the 663 mutations using runif in R. 3. The annotation of the Mmul_8.0.1 reference genome was from Ensembl. Lines 631-637 : “We defined a cluster as a window of 20,000 bp, similarly to Besenbacher et. al. 2016 [37], and qualify how many mutations were clustered together; over all individuals, looking at related individuals, and within individuals. We simulated 663 mutations following a uniform distribution (runif function in R) to compare with our dataset. We investigated the mutations that are shared between related individuals. Finally, we looked at the location of mutations in the coding region using the annotation of the Mmul_8.0.1 reference genome from Ensembl.”

lines 619-622: Could the authors please comment on their rational to use a previously published estimate of the nucleotide diversity from different individuals rather than to estimate theta directly from their own data? Moreover, the usage of the estimate provided by Xue et al. 2016 is problematic here as it contains coding regions (thus subject to purifying selection, and background selection effects).

Response: We did not use our data as individuals were related.

lines 626-627: In their introduction, the authors state that "female macaques generally start reproducing right after maturation" (i.e., around three years old) while "males rarely reproduce in the wild until (...) eight years old" (lines 113-117). Yet, in their analysis, they "assumed an average age of reproduction in the wild at 10 years old for females and 12 years old for males". Could the authors please comment on their choice of reproduction time (3-vs-10 years for females and 8-vs-12 years for males)?

Response: We used an average reproduction time based on maturation time and survival of the species in the wild. These ages are also consistent with a generation time of 11 years used in other studies.

In the manuscript, the authors use "mu" for both the mutation rate per site per generation (e.g., in "Estimation of the mutation rate per site per generation") and for the yearly mutation rate (e.g., "Molecular dating using the new mutation rate"). A superscript might help to clarify the difference for the reader.

Response: We thank the reviewer for this comment and changed μ for μyearly referring to the yearly rate.

Figure 4 is missing a x-axis label and units. Ne and Ts estimates are missing for Cercopithecidae and Papionini. Were the pictures taken by the authors (i.e., no picture credit is provided)?

Response: We thank the reviewer for this comment. We have added the x-axis label and credits for the pictures used in this figure. There is no estimation of Ne or Ts available for the two ancestral nodes mentioned by the reviewer.

Reviewer #2: This is a revised manuscript which I reviewed previously. I believe the authors have responded well to all my major concerns. In addition, I think the authors have also responded

satisfactorily to the concerns and issues raised by the other reviewer.

Response: We thank the reviewer for this comment.

However, I do have a series of minor suggestions for edits relevant to this revised draft. None of these suggestions constitute major concerns and the authors and editors should not consider any of these necessary changes. These are provided as suggestions only. I now recommend that this manuscript be accepted for publication in Gigascience.

Response: We thank the reviewer for this additional input and have taken into account points 1,2,3,4,6,7. We answered to point 5.

1) Line 112: I realize this may be a small quibble, but I think this sentence would be better as "….which is 93% identical to the genome of humans…"
2) Line 119: Again a small change to "….as a member of the closest related outgroup…" since macaques are certainly not the only lineage in that group.
3) Line 148: change to "…for which the prior published mutation rate was estimated at 1.27…"
4) Lines 194-5: change to "…these two studies are very close, with our study only 5% lower."
5) Lines 200-206: I think it might be better to move this statement about the correlations between paternal and maternal age and mutation rates below, after you describe how you phased the mutations and that you observed a larger number of paternal vs maternal mutations. It seems a more logical flow to report paternal and maternal age effects after you describe phasing and distinguishing paternal from maternal mutations.

Response: We kept this order to clearly show that the first parental age regression was on the rate per generation without the phasing, compared to the regression after the phasing now dissociating the parental effect from each other.

6) Line 306: Would this be better as "Given that a precise….
7) Line 403: Would this be better as "However, if we calculate the de nov

Close
