# Supplementary material for: The germline mutational process in rhesus macaque and its implications for phylogenetic dating
Source: Gigascience. 2021 May 5;10(5):giab029. doi: 10.1093/gigascience/giab029 (PMC8099771; doi:10.1093/gigascience/giab029)
Supplement: giab029_Reviewer_1_Report_Original_Submission — Susanne Pfeifer -- 11/23/2020 Reviewed [file giab029_reviewer_1_report_original_submission.pdf]

Reviewer Report

Title: The germline mutational process in rhesus macaque and its implications for phylogenetic dating

Version: Original Submission **Date:** 11/23/2020

Reviewer name: Susanne Pfeifer

Reviewer Comments to Author:

Using whole genome sequencing data from 33 individuals / 19 trios, Bergeron and colleagues directly estimate the spontaneous mutation rate in rhesus macaque (average rate: 0.77 x 10^-8 per site per generation) which they in turn use to date divergence times across primates.
Overall, this study is highly similar to recent work published by Wang et al. (2020) who estimated the spontaneous mutation rate in rhesus macaque from whole genome sequencing data of 32 individuals. Wang et al. (2020) estimated an average rate of 0.58 x 10^-8 per site per generation. There is a large discrepancy between these two similarly sized datasets which requires an explanation. As expected, the age of the parents is a contributor but, by itself, it is insufficient to explain the observed differences. In fact, it has long been known in the field (and indeed it has been pointed out by the authors themselves here) that differences in the computational pipeline (such as variant calling as well as estimations of false negative and false positive rates) can have large effects on de novo mutation estimates. The authors implemented their own pipeline which deviates in several aspects from the "best practices" used in similar studies. This is problematic, not only because several of the applied filter criteria can lead to systematic biases, but also because it is hindering a straightforward interpretation of their results. As a consequence, it will be of utmost importance to provide a benchmark using both a well-annotated human dataset as well as the previously published rhesus dataset.
Aside from the differences in filter criteria between the studies, the authors claim that their higher sequencing coverage leads to more reliable results. This claim is not supported in the manuscript. Indeed, it is rather surprising given that the reported false positive and false negative rates (10.89% and 4.02%, respectively) are in the range of those reported in previous studies of lower sequencing coverage. Importantly though, there is also an issue with the way the authors estimate the false positive rate. Specifically, the authors note that "the manual curation may have missed the realignment executed during variant calling". As GATK's HaplotypeCaller locally reassembles genomic regions to determine the haplotype, this is greatly concerning as the authors do not actually visually inspect the final (i.e., reassembled) genomic region using IGV - in other words, they can't actually assess whether a site shows evidence for a de novo mutation or not. This issue becomes apparent in the figures presented in the Supplementary Material. Supplementary Figure 1b highlights a site which has been computationally classified as a de novo mutation candidate. This site does not show any evidence for an alternative allele in the offspring when, according to the authors own definition ("in the case of a de novo mutation, the number of alternative alleles seen in the offspring should account for ~50 % of the reads"), it should (that is, assuming that the computational pipeline is correctly implemented). I can't comment on the estimation of the false negative rate, as it is unclear how exactly the simulations were performed (e.g., were sequencing errors incorporated and if so, at which rate and under which model;

what proportion of reference vs alternative alleles were simulated, etc.). Lastly, it is important to treat non-variant sites (which are used to calculate the denominator) in the exact same way than variant sites. The application of fewer filter criteria to non-variant sites (i.e., the authors state that site filters are being excluded) will result in an overestimate of callability and hence an underestimate of the mutation rate.

Another point that requires additional explanation (though perhaps this might become mute after re-analysis of their data), is the inconsistency of the dates inferred for the human/Old World monkey split with both the fossil record as well as with previously published molecular results. It is also unclear why the authors decided to focus on the model with independent age effects given the previous literature on this topic (was this model the best fit?).

Availability of data and materials:

Data: BioProject PRJNA588178 does not exist on NCBI.

Materials: The authors have made their scripts publicly available via GitHub. As GitHub entries can be altered, I would encourage the authors to deposit their scripts in a permanent repository (e.g., Zenodo).

Methods

Are the methods appropriate to the aims of the study, are they well described, and are necessary controls included? Choose an item.

Conclusions

Are the conclusions adequately supported by the data shown? Choose an item.

Reporting Standards

Does the manuscript adhere to the journal's guidelines on minimum standards of reporting? Choose an item.

Choose an item.

Statistics

Are you able to assess all statistics in the manuscript, including the appropriateness of statistical tests used? Choose an item.

Quality of Written English

Please indicate the quality of language in the manuscript: Choose an item.

Declaration of Competing Interests

Please complete a declaration of competing interests, considering the following questions:

- Have you in the past five years received reimbursements, fees, funding, or salary from an organisation that may in any way gain or lose financially from the publication of this manuscript, either now or in the future?
- Do you hold any stocks or shares in an organisation that may in any way gain or lose financially from the publication of this manuscript, either now or in the future?
- Do you hold or are you currently applying for any patents relating to the content of the manuscript?
- Have you received reimbursements, fees, funding, or salary from an organization that holds or has applied for patents relating to the content of the manuscript?
- Do you have any other financial competing interests?
- Do you have any non-financial competing interests in relation to this paper?

If you can answer no to all of the above, write 'I declare that I have no competing interests' below. If your reply is yes to any, please give details below.

I declare that I have no competing interests.

I agree to the open peer review policy of the journal. I understand that my name will be included on my report to the authors and, if the manuscript is accepted for publication, my named report including any attachments I upload will be posted on the website along with the authors' responses. I agree for my report to be made available under an Open Access Creative Commons CC-BY license (http://creativecommons.org/licenses/by/4.0/). I understand that any comments which I do not wish to be included in my named report can be included as confidential comments to the editors, which will not be published.

Choose an item.

To further support our reviewers, we have joined with Publons, where you can gain additional credit to further highlight your hard work (see: https://publons.com/journal/530/gigascience). On publication of this paper, your review will be automatically added to Publons, you can then choose whether or not to claim your Publons credit. I understand this statement.

Yes Choose an item.
