# Supplementary material for: The germline mutational process in rhesus macaque and its implications for phylogenetic dating
Source: Gigascience. 2021 May 5;10(5):giab029. doi: 10.1093/gigascience/giab029 (PMC8099771; doi:10.1093/gigascience/giab029)
Supplement: giab029_Reviewer_1_Report_Revision_1 — Susanne Pfeifer -- 2/1/2021 Reviewed [file giab029_reviewer_1_report_revision_1.pdf]

Reviewer Report

Title: The germline mutational process in rhesus macaque and its implications for phylogenetic dating

Version: Revision 1 **Date:** 2/1/2021

Reviewer name: Susanne Pfeifer

Reviewer Comments to Author:

DNM detection:
While I agree with the authors that there are no current Best Practices in terms of de novo mutation detection, I was actually referring to the Best Practices in variant calling. Most other de novo mutation studies followed the well-established GATK's Best Practices pipeline and the impact of the authors' modified filter criteria is somewhat unclear. In their rebuttal, the authors state that they now "provide a comparison with a published trio of chimpanzees as a validation of our method", and while indeed mentioned in the main text (lines 147-151), this validation is unfortunately not included in the Materials and Methods section, hindering its evaluation.
False positives:
My main concern regarding the manual/visual validation remains, i.e., given that the authors do not inspect the final, locally reassembled .bam files, they won't be able to discern between true de novo mutations and false positives in their pipeline (see my previous comments). The procedure of analysing intermediate files simply can't work (e.g., looking at the final .bam files can reveal, as mentioned in the rebuttal, that mutations currently classified as a false positive are indeed genuine de novo mutations - and vice versa). The final files used to detect de novo mutations are readily available from their GATK runs so there is really no good reason not to use them. If the authors truly believe that "it is harder to call variants in the realigned regions of the genome and that these regions are more prone to false-positives", why perform the variant calling that way?
False negatives:
Thank you for your clarification. In short, the authors estimate the false negative rate for their site filters, however, there are many other reasons why a genuine de novo mutation might not have been detected as such (e.g., due to mis-mapping of reads by the aligner). These down-stream factors need to be accounted for when estimating the false negative rate - and this can only be achieved by simulation (i.e., when the ground-truth is known).
Availability of data and materials: Bioproject PRJNA588178 is incomplete - it only contains data from 33 individuals (in contrast, lines 485-499 mention that "whole blood samples (...) were collected from 53 rhesus macaques.")
None of the scripts used for the actual analysis (i.e., once the de novo mutation candidates were identified) or plots are included in the current GitHib directory (in addition, the newly created Zenodo repository is not included in the manuscript). For completeness and reproducibility, these scripts should be added.
Other comments:
lines 91-93: "The mutation rate of baboon (Papio anubis) [30] and grey mouse lemur (Microcebus

murinus) [31] have also been estimated in preprinted studies." The mutation rate of baboon has been published in August 2020 (Wu et al. 2020).
lines 495-496: "Whole-genome pair-ended sequencing was performed on BGISEQ500 platform, with a read length of 2x100 bp." Could the authors please verify that their sequencing protocol was PCR-free (as PCR errors are problematic for the detection of de novo mutations)?
lines 501-521: BWA and IGV references are missing. What version of Picard was used?
lines 526-528: "Mendelian violations were selected using GATK SelectVariant and refined to only keep sites where both parents were homozygote reference (HomRef), and their offspring was heterozygote (Het)." My initial reading was that the authors ran gatk SelectVariant (e.g., "gatk SelectVariants -V variants.vcf --select ''vc.getGenotype("father").isHomRef()' --select ''vc.getGenotype("mother").isHomRef()' --select ''vc.getGenotype("offspring").isHet()'") - however, after reading lines 542-544 (i.e., "2,251,363 were potential Mendelian violations found by GATK (...), 177,227 were filtered Mendelian violations with parents HomRef and offspring Het"), it sounds like the authors might have run a two-step process instead, i.e., first "gatk FindMendelianViolations", then "gatk SelectVariants". Could the authors please clarify?
lines 569-572: "We used the BP_RESOLUTION option in GATK to call variants for each position (...). So unlike other studies, we do not have to rely on sequencing depth as a proxy for genotype quality at those sites." Several earlier studies actually used GATK in BP_RESOLUTION (including my own study in green monkeys).
lines 608-616: The paragraph concerning "Characterization of de novo mutations" is lacking any detailed information. For example, how was the window size or the number of mutations to simulate chosen? How were the mutations actually simulated (e.g., which software was used, which parameters, etc.)? Where was the annotation of the reference genome obtained from?
lines 619-622: Could the authors please comment on their rational to use a previously published estimate of the nucleotide diversity from different individuals rather than to estimate theta directly from their own data? Moreover, the usage of the estimate provided by Xue et al. 2016 is problematic here as it contains coding regions (thus subject to purifying selection, and background selection effects).
lines 626-627: In their introduction, the authors state that "female macaques generally start reproducing right after maturation" (i.e., around three years old) while "males rarely reproduce in the wild until (...) eight years old" (lines 113-117). Yet, in their analysis, they "assumed an average age of reproduction in the wild at 10 years old for females and 12 years old for males". Could the authors please comment on their choice of reproduction time (3-vs-10 years for females and 8-vs-12 years for males)?
In the manuscript, the authors use "mu" for both the mutation rate per site per generation (e.g., in "Estimation of the mutation rate per site per generation") and for the yearly mutation rate (e.g., "Molecular dating using the new mutation rate"). A superscript might help to clarify the difference for the reader.
Figure 4 is missing a x-axis label and units. Ne and Ts estimates are missing for Cercopithecidae and Papionini. Were the pictures taken by the authors (i.e., no picture credit is provided)?

Methods

Are the methods appropriate to the aims of the study, are they well described, and are necessary controls included? Choose an item.

Conclusions

Are the conclusions adequately supported by the data shown? Choose an item.

Reporting Standards

Does the manuscript adhere to the journal's guidelines on minimum standards of reporting? Choose an item.

Choose an item.

Statistics

Are you able to assess all statistics in the manuscript, including the appropriateness of statistical tests used? Choose an item.

Quality of Written English

Please indicate the quality of language in the manuscript: Choose an item.

Declaration of Competing Interests

Please complete a declaration of competing interests, considering the following questions:

- Have you in the past five years received reimbursements, fees, funding, or salary from an organisation that may in any way gain or lose financially from the publication of this manuscript, either now or in the future?
- Do you hold any stocks or shares in an organisation that may in any way gain or lose financially from the publication of this manuscript, either now or in the future?
- Do you hold or are you currently applying for any patents relating to the content of the manuscript?
- Have you received reimbursements, fees, funding, or salary from an organization that holds or has applied for patents relating to the content of the manuscript?
- Do you have any other financial competing interests?
- Do you have any non-financial competing interests in relation to this paper?

If you can answer no to all of the above, write 'I declare that I have no competing interests' below. If your reply is yes to any, please give details below.

I declare that I have no competing interests.

I agree to the open peer review policy of the journal. I understand that my name will be included on my report to the authors and, if the manuscript is accepted for publication, my named report including any attachments I upload will be posted on the website along with the authors' responses. I agree for my report to be made available under an Open Access Creative Commons CC-BY license (http://creativecommons.org/licenses/by/4.0/). I understand that any comments which I do not wish to be included in my named report can be included as confidential comments to the editors, which will not be published.

Choose an item.

To further support our reviewers, we have joined with Publons, where you can gain additional credit to further highlight your hard work (see: https://publons.com/journal/530/gigascience). On publication of this paper, your review will be automatically added to Publons, you can then choose whether or not to claim your Publons credit. I understand this statement.

Yes Choose an item.
