# Supplementary material for: The germline mutational process in rhesus macaque and its implications for phylogenetic dating
Source: Gigascience. 2021 May 5;10(5):giab029. doi: 10.1093/gigascience/giab029 (PMC8099771; doi:10.1093/gigascience/giab029)
Supplement: giab029_Reviewer_2_Report_Original_Submission — Jeffrey Rogers, Ph.D. -- 11/30/2020 Reviewed [file giab029_reviewer_2_report_original_submission.pdf]

Reviewer Report

Title: The germline mutational process in rhesus macaque and its implications for phylogenetic dating

Version: Original Submission **Date:** 11/30/2020

Reviewer name: Jeffrey Rogers, Ph.D.

Reviewer Comments to Author:

This manuscript reports a novel analysis of de novo mutation rate in rhesus macaques. This species is a major biomedical laboratory animal, and one of the most studied nonhuman primates. Thus, fundamental information about this species, such as the rate of new single nucleotide mutations, is valuable and significant. The authors have investigated de novo mutations across 19 trios (sire, dam, offspring) although several of the offspring share sires, so the 19 trios are not entirely independent. These trios were sequenced to deep (76x average) genome-wide coverage, which constitutes a high-quality dataset. The authors have developed an analytical pipeline that estimates the proportion of the genome that they are surveying for new mutations, the false positive rate for observed parent-offspring differences and the false negative rate (missed true mutations). This pipeline seems to me to be well justified and appropriate. Thus, the estimates of de novo mutation rate presented (0.77 x 10e-8 per site per generation, or 0.62 x 10e-9 per site per year assuming a parental breeding age of 12 for males and 10 for females) are interesting. These results for per generation and per year mutation rates are in my opinion reasonable and valid, worthy of publication.

The authors report two additional observations concerning de novo mutations. They observe that most de novo mutations are passed to offspring by fathers rather than mothers, and that older fathers generate more mutations than younger fathers. Both these observations have been previously reported for both humans and rhesus macaques. So these findings are not novel, but this report is important because it confirms the one previous report in rhesus macaques and further demonstrates in an independent dataset the generality of the differential paternal mutation effect across different species.

Major issues:

1) In lines 249 - 258 the authors compare the spectrum of mutation types observed in their study to humans and chimpanzees. These comparisons are fine, but the authors should also compare their data to other published data for rhesus macaques.

2) My most significant concern and criticism of this manuscript relates to the authors' proposed dates of divergence among major primate clades. The authors estimate the divergence of Macaca mulatta from M. fascicularis to be about 3.9 mya, which is slightly but not seriously older than previous estimates that used fossil data to inform estimates of molecular divergence. Somewhat more worrying, but not a substantial problem is the Bergeron et al. estimate for Papio vs. Macaca which is again older than prior published results. Based on my critique below, I have some doubts about this Papio-Macaca divergence date, but the problem is most obvious in earlier evolutionary divergence events.

The major problem related to the Bergeron et al. estimate of the divergence date for Hominoidea vs. Cercopithecoidea. Their estimate of 52.31 mya is far older than previous estimates and seems to be out of the range of potential error in those other results. In order to be correct, the other studies must have

made a serious error of some type. Bergeron et al. acknowledge that they are aware that this new estimate conflicts strongly with prior studies. The authors further acknowledge that their direct mutation rate approach to dating divergences can overestimate divergence times due to evolutionary changes in either the mutation rate or generation time, or both (lines 439-441 of manuscript).

One obvious issue is the use of the new macaque mutation rate per year for both hominoids and cercopithecoids. A reader might be tempted to suggest that the best estimate for the human mutation rate should be used for that lineage, rather than the macaque mutation rate. But the human rate per year is slower than the macaque rate, so using the current estimated human rate for the hominoid lineage would slow down accumulation of genomic differences further and hence push the divergence date even farther back in time. This use of human rate for inferences regarding evolution in Hominoidea might seem reasonable, but would only exacerbate the problem that the human-macaque divergence seems to be too old.

I would suggest that one problem with the analysis as presented by Bergeron et al. is the estimated genetic difference between humans and macaques. Their paper cites Moorjani et al. (2016) as the source for the genomic difference, but Moorjani et al. (2016) cites the UCSC browser as it stood in 2014. There are now more recent and more accurate reference genome sequences for both humans and macaques (and other nonhuman primates) that might be used to generate a more defensible estimate of the sequence differences to be used in line 606 of the manuscript. This is unlikely to make a large difference and to solve the dating problem, but best to use the most up-to-date genomic data.

Furthermore, I would suggest that, based on the Bergeron results, the common ancestor of hominoids and cercopithecoids may have had a higher mutation rate per year than either extant group. This is plausible since the body size was probably smaller than modern rhesus macaques, and other aspects of demography and biology of the outgroups (platyrrhines and strepsirrhines) suggest significant derived evolution in both cercopithecoids and hominoids relative to the ancestor. So assumptions regarding the use mutation rates estimated from macaque and human pedigree studies may not be valid when trying to infer rates of evolution more than 20 million years in the past.

In order to address this problem of a surprisingly old human-macaque divergence date, I suggest that the authors might explore evolutionary scenarios that calculate the effects of changes in mutation rates, generation time, breeding ages or other parameters on estimated divergence dates. That is, they should attempt to determine whether there is a plausible scenario produced through reasonable assumptions for the relevant parameters that makes the estimated divergence date of cercopithecoids vs. hominoids more recent, i.e. closer to what is concluded based on fossils and other molecular analyses. If there is no plausible scenario available, then Bergeron et al. will have to provide a stronger argument for the severely underestimated age of hominoid-cercopithecoid divergence and the remarkable gap in the fossil record. They would (in my opinion) need to address prior studies by Pozzi et al. (2014, Molec. Phylogenet and Evol. 75: 165-183) and Wilkinson et al (2011, Syst. Biol. 60: 16-31) or the reviews of primate paleontology by Harrison or Jablonski and Frost in the authoritative volume Cenozoic Mammals of Africa (2010, Univ. of California Press).

If Bergeron et al. wish to defend the conclusion that the date of divergence of hominoids vs. cercopithecoids is more than 60% older than prior studies have estimated (52 mya vs 30-32 mya), then they should make a strong argument that addresses the prior studies at some level of detail. The current manuscript states the conclusion, and provides some general comments, but does not adequately make

the case that paleontologists and other geneticists have missed the boat by 20 million years.
Minor issues:
Line 111: The sister group of the superfamily Hominoidea is the superfamily Cercopithecoidea, not the family Cercopithecidae. The authors refer to Cercopithecidae in several places through the manuscript. I suggest that it would be more accurate to refer to Cercopithecoidea.
Lines 155-6: I believe this line should be corrected to read "…due to the incorrect absence of a call of the variant in the parents or the incorrect presence of a called variant in the offspring…"
Line 175: Should be "…number of callable…"
Reviewer: Jeffrey Rogers (Baylor College of Medicine)

Methods

Are the methods appropriate to the aims of the study, are they well described, and are necessary controls included? Choose an item.

Conclusions

Are the conclusions adequately supported by the data shown? Choose an item.

Reporting Standards

Does the manuscript adhere to the journal's guidelines on minimum standards of reporting? Choose an item.

Choose an item.

Statistics

Are you able to assess all statistics in the manuscript, including the appropriateness of statistical tests used? Choose an item.

Quality of Written English

Please indicate the quality of language in the manuscript: Choose an item.

Declaration of Competing Interests

Please complete a declaration of competing interests, considering the following questions:

- Have you in the past five years received reimbursements, fees, funding, or salary from an organisation that may in any way gain or lose financially from the publication of this manuscript, either now or in the future?
- Do you hold any stocks or shares in an organisation that may in any way gain or lose financially from the publication of this manuscript, either now or in the future?

- Do you hold or are you currently applying for any patents relating to the content of the manuscript?
- Have you received reimbursements, fees, funding, or salary from an organization that holds or has applied for patents relating to the content of the manuscript?
- Do you have any other financial competing interests?
- Do you have any non-financial competing interests in relation to this paper?

If you can answer no to all of the above, write 'I declare that I have no competing interests' below. If your reply is yes to any, please give details below.

I declare that I have no competing interests.

I agree to the open peer review policy of the journal. I understand that my name will be included on my report to the authors and, if the manuscript is accepted for publication, my named report including any attachments I upload will be posted on the website along with the authors' responses. I agree for my report to be made available under an Open Access Creative Commons CC-BY license (http://creativecommons.org/licenses/by/4.0/). I understand that any comments which I do not wish to be included in my named report can be included as confidential comments to the editors, which will not be published.

Choose an item.

To further support our reviewers, we have joined with Publons, where you can gain additional credit to further highlight your hard work (see: https://publons.com/journal/530/gigascience). On publication of this paper, your review will be automatically added to Publons, you can then choose whether or not to claim your Publons credit. I understand this statement.

Yes Choose an item.
