# Supplementary material for: The germline mutational process in rhesus macaque and its implications for phylogenetic dating
Source: Gigascience. 2021 May 5;10(5):giab029. doi: 10.1093/gigascience/giab029 (PMC8099771; doi:10.1093/gigascience/giab029)
Supplement: giab029_Reviewer_2_Report_Revision_1 — Jeffrey Rogers, Ph.D. -- 1/23/2021 Reviewed [file giab029_reviewer_2_report_revision_1.pdf]

Reviewer Report

Title: The germline mutational process in rhesus macaque and its implications for phylogenetic dating

Version: Revision 1 **Date:** 1/23/2021

Reviewer name: Jeffrey Rogers, Ph.D.

Reviewer Comments to Author:

This is a revised manuscript which I reviewed previously. I believe the authors have responded well to all my major concerns. In addition, I think the authors have also responded satisfactorily to the concerns and issues raised by the other reviewer.

However, I do have a series of minor suggestions for edits relevant to this revised draft. None of these suggestions constitute major concerns and the authors and editors should not consider any of these necessary changes. These are provided as suggestions only. I now recommend that this manuscript be accepted for publication in Gigascience.

1) Line 112: I realize this may be a small quibble, but I think this sentence would be better as "….which is 93% identical to the genome of humans…"

2) Line 119: Again a small change to "….as a member of the closest related outgroup…" since macaques are certainly not the only lineage in that group.

3) Line 148: change to "…for which the prior published mutation rate was estimated at 1.27…"

4) Lines 194-5: change to "…these two studies are very close, with our study only 5% lower."

5) Lines 200-206: I think it might be better to move this statement about the correlations between paternal and maternal age and mutation rates below, after you describe how you phased the mutations and that you observed a larger number of paternal vs maternal mutations. It seems a more logical flow to report paternal and maternal age effects after you describe phasing and distinguishing paternal from maternal mutations.

6) Line 306: Would this be better as "Given that a precise….

7) Line 403: Would this be better as "However, if we calculate the de novo mutation…."

Methods

Are the methods appropriate to the aims of the study, are they well described, and are necessary controls included? Choose an item.

Conclusions

Are the conclusions adequately supported by the data shown? Choose an item.

Reporting Standards

Does the manuscript adhere to the journal's guidelines on [minimum standards of reporting?]() Choose an item.

Choose an item.

Statistics

Are you able to assess all statistics in the manuscript, including the appropriateness of statistical tests used? Choose an item.

Quality of Written English

Please indicate the quality of language in the manuscript: Choose an item.

Declaration of Competing Interests

Please complete a declaration of competing interests, considering the following questions:

- Have you in the past five years received reimbursements, fees, funding, or salary from an organisation that may in any way gain or lose financially from the publication of this manuscript, either now or in the future?
- Do you hold any stocks or shares in an organisation that may in any way gain or lose financially from the publication of this manuscript, either now or in the future?
- Do you hold or are you currently applying for any patents relating to the content of the manuscript?
- Have you received reimbursements, fees, funding, or salary from an organization that holds or has applied for patents relating to the content of the manuscript?
- Do you have any other financial competing interests?
- Do you have any non-financial competing interests in relation to this paper?

If you can answer no to all of the above, write 'I declare that I have no competing interests' below. If your reply is yes to any, please give details below.

I declare that I have no competing interests

I agree to the open peer review policy of the journal. I understand that my name will be included on my report to the authors and, if the manuscript is accepted for publication, my named report including any attachments I upload will be posted on the website along with the authors' responses. I agree for my report to be made available under an Open Access Creative Commons CC-BY license (http://creativecommons.org/licenses/by/4.0/). I understand that any comments which I do not wish to be included in my named report can be included as confidential comments to the editors, which will not be published.

Choose an item.

To further support our reviewers, we have joined with Publons, where you can gain additional credit to further highlight your hard work (see: https://publons.com/journal/530/gigascience). On publication of this paper, your review will be automatically added to Publons, you can then choose whether or not to claim your Publons credit. I understand this statement.

Yes Choose an item.
